# Divergent Synthesis of Homoallylic and Allenic Sulfides, Selenides, and S,C-Sulfonium Ylides by Ruthenium Catalysis

**DOI:** 10.1021/prechem.5c00098

**Published:** 2025-11-14

**Authors:** Zhiyue He, Li Wu, Yanli Ma, Liting Wen, Xueqing Liu, Wenqian Ding, Wanqi Hu, Xiaowei Wu

**Affiliations:** † Drug Discovery and Development Center, 58298 Shanghai Institute of Materia Medica, Chinese Academy of Sciences, Shanghai 201203, China; ‡ Zhongshan Institute for Drug Discovery, Shanghai Institute of Materia Medica, Chinese Academy of Sciences, Zhongshan 528400, China; § School of Chinese Materia Medica, 66478 Nanjing University of Chinese Medicine, Nanjing 210023, China; ∥ 47879 Guangzhou University of Chinese Medicine, Guangdong 510006, China; ⊥ University of Chinese Academy of Sciences, Beijing 100049, China

**Keywords:** ruthenium catalysis, decarbonylation, [2,3]-sigmatropic rearrangement, hypervalent iodine reagents, sulfonium ylides

## Abstract

Compounds bearing benzothiophene and thioether motifs have attracted broad interest because of their prevalence across natural products, advanced materials, agrochemicals, and therapeutic agents. Importantly, many marketed drugs incorporate C–S linkages, underscoring the ongoing need for streamlined methods to construct sulfur-containing molecules. We herein report ruthenium-catalyzed divergent synthesis of homoallylic and allenic sulfides, selenides, and S,C-sulfonium ylides from iodonium ylides. This work features a ruthenium-catalyzed Doyle–Kirmse-type reaction of iodonium ylides, followed by a rare decarbonylation to deliver homoallylic and allenic sulfides/selenides. The method operates under mild conditions with broad substrate scope and good functional group tolerance, while extending the Doyle–Kirmse reaction beyond diazo compounds by employing stable, nonexplosive iodonium ylides. Furthermore, we also describe a ruthenium-catalyzed transylidation strategy that couples *N*-acyl sulfenamides with iodonium ylides to generate a wide range of S,C-sulfonium ylides under mild conditions. This method features a broad substrate scope, good tolerance toward functional groups, and an inexpensive ruthenium catalyst. Moreover, scale-up and subsequent derivatization experiments demonstrate its practicality. Overall, this protocol provides a versatile entry to diverse sulfonium ylides and opens opportunities for their application in synthetic chemistry.

## Graphical abstract

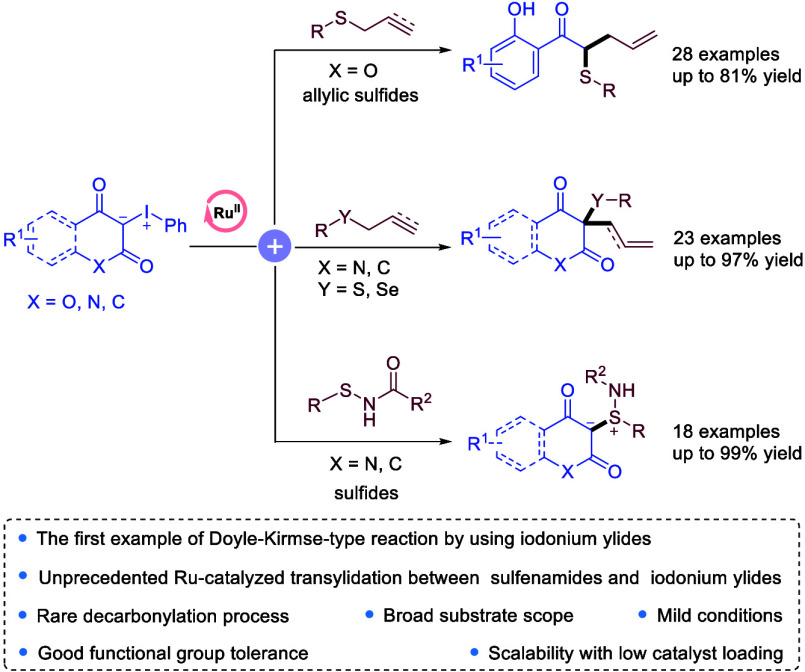

## Introduction

Transition metal-catalyzed carbene transfer reactions have emerged as powerful tools for C–X bond formation and molecular skeleton reorganization.^1–9^ Among these, the Doyle–Kirmse reaction involving diazo compounds and allylic sulfides was originally reported by Kirmse and later refined by Doyle (Figure 1a).^10–13^ In situ generation of metal carbenes is typically achieved through transition metal-catalyzed activation of diazo compounds.^14,15^ Extensive studies have since demonstrated the effectiveness of copper- and rhodium-based catalysts in facilitating this transformation, while more recently, palladium, ruthenium, cobalt, iron, gold, and silver have also been explored as viable catalysts.^10–34^ In addition, several examples of metal-catalyzed Doyle–Kirmse reactions by using *N*-sulfonylhydrazones as diazo precursors have been reported in recent years.^35–37^ The Doyle–Kirmse reaction proceeds via a metal carbene-mediated process involving sulfonium ylide formation and a subsequent [2,3]-sigmatropic rearrangement, making it a valuable strategy for constructing C–C, C–S, and C–Se bonds. This reaction provides an efficient route to homoallylic sulfides, which serve as versatile intermediates in organic synthesis.^14,15,38^ In the Doyle–Kirmse reactions, diazo compounds have traditionally served as carbene precursors. Nevertheless, the instability, toxicity, and potential explosiveness of diazo compounds pose prominent safety concerns. Therefore, the development of novel, safer carbene precursors as alternatives to diazo compounds in the Doyle–Kirmse reactions is highly desirable.

**Figure 1:**
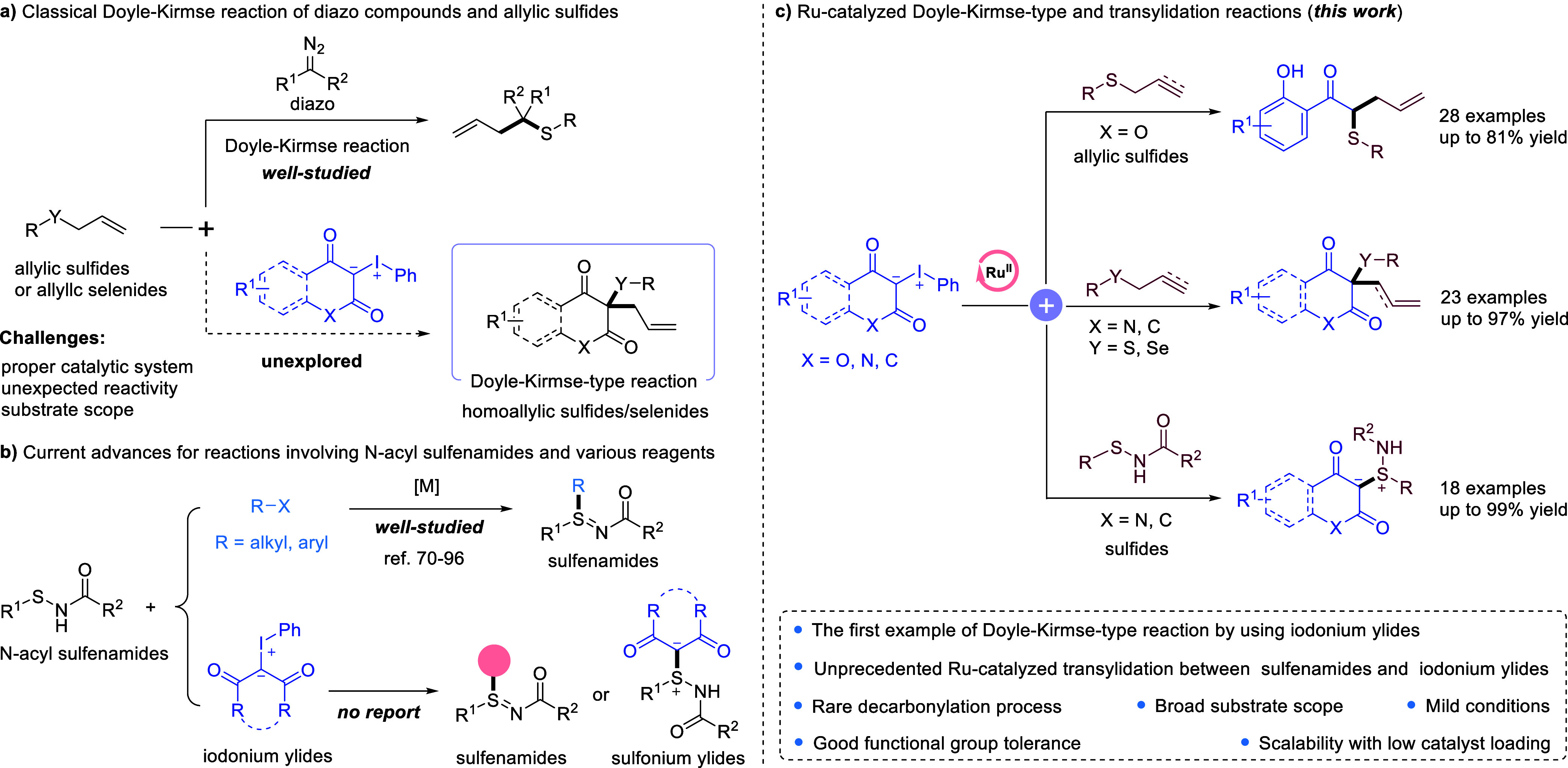
Transition metal-catalyzed Doyle–Kirmse and transylidation reactions.

Furthermore, benzothiophene, thioethers, and related derivatives have drawn significant attention owing to their broad distribution in medicinal agents, materials, and natural products. A lot of marketed pharmaceuticals feature C–S linkages, underscoring the importance of creating reliable methods for assembling sulfur-containing structures.^39^ Within this framework, sulfonium ylides serve as powerful intermediates, offering access to diverse scaffolds and playing key roles in transformations such as aziridination, cyclopropanation, epoxidation, and nucleophilic processes.^40–53^ At the same time, iodonium ylides have emerged as valuable reagents with rich reactivity in metal-mediated reactions, including arylation, cycloadditions, and cyclopropanation.^54–69^ Despite these advances, approaches that exploit iodonium ylides for the preparation of S,C-sulfonium ylides remain poorly developed.

Moreover, extensive studies have focused on the reactions of *N*-acyl sulfenamides with various reagents, including alkyl halides,^70–73^ aryl iodides,^74–76^ arylboronic acids,^77,78^ diazo compounds,^79–82^ aryldiazonium salts,^83^ diaryliodonium salts,^84–87^ alkenyl iodonium salts,^88^ aryl sulfonium salts,^89^ and other reagents^90–96^ for the synthesis of sulfilimines in recent years (Figure 1b). However, the reaction between *N*-acyl sulfonamides and iodonium ylides for the synthesis of sulfilimines or S,C-sulfonium ylides has not yet been explored (Figure 1b). Despite the versatile reactivity of iodonium ylides, the potential of iodonium ylides in Doyle–Kirmse-type and transylidation reactions remains largely underdeveloped. The challenges include (a) identifying an efficient catalytic system to promote the divergent synthesis of homoallylic and allenic sulfides, selenides, and S,C-sulfonium ylides from iodonium ylides; (b) controlling the selective formation of S,C-sulfonium ylides and facilitating the subsequent [2,3]-sigmatropic rearrangement; and (c) expanding the substrate scope to accommodate structurally diverse iodonium ylides and sulfides.

**Table 1:**
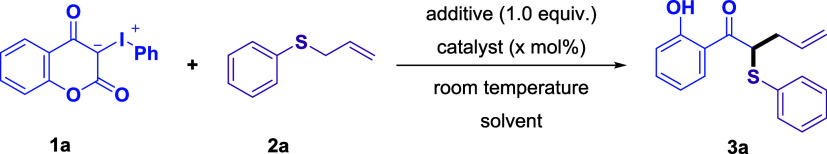
Optimization of Reaction Conditions^a^

entry	catalyst	additive	solvent	yield (%)
1	[RuCl_2_(*p-*cymene)]_2_	-	TFE	69
2	[Cp*RhCl_2_]_2_	-	TFE	58
3	Cp*Co(CO)I_2_	-	TFE	45
4	Rh_2_(OAc)_4_	-	TFE	17
5	Pd(OAc)_2_	-	TFE	trace
6	[RuCl_2_(*p-*cymene)]_2_	-	DCM	62
7	[RuCl_2_(*p-*cymene)]_2_	-	toluene	51
8	[RuCl_2_(*p-*cymene)]_2_	-	EA	48
9	[RuCl_2_(*p-*cymene)]_2_	-	THF	48
10	[RuCl_2_(*p-*cymene)]_2_	-	MeOH	21
11	[RuCl_2_(*p-*cymene)]_2_	-	HFIP	63
12	[RuCl_2_(*p-*cymene)]_2_	NaOAc	TFE	38
13	[RuCl_2_(*p-*cymene)]_2_	Cs_2_CO_3_	TFE	31
14^b^	[RuCl_2_(*p-*cymene)]_2_	-	TFE	70
15^c^	[RuCl_2_(*p-*cymene)]_2_	-	TFE	72
16^d^	[RuCl_2_(*p-*cymene)]_2_	-	TFE	81

a Reaction conditions: **1a** (0.1 mmol), **2a** (0.12 mmol), [RuCl_2_(*p-*cymene)]_2_ (2.5 mol %), under air, TFE (2.0 mL), rt, and 12 h. Isolated yields are reported.

b 5 mol % [RuCl_2_(*p-*cymene)]_2_ was used.

c 0.14 mmol **1a** and 0.1 mmol **2a** were used.

d 0.2 mmol **1a** and 0.1 mmol **2a** were used.

In this study, we report a ruthenium-catalyzed Doyle–Kirmse-type reaction utilizing iodonium ylides, followed by a unique decarbonylation process, enabling the synthesis of novel homoallylic and allenic sulfur- and selenium-containing compounds (Figure 1c). By utilizing stable and nonexplosive iodonium ylides as carbene precursors, this approach mitigates the hazards associated with traditional diazo compounds, offering a safe and reliable strategy for carbon–sulfur, carbon–selenium, and carbon–carbon bond formation. Additionally, we also introduce a previously unreported ruthenium-catalyzed transylidation between *N*-acyl sulfenamides and iodonium ylides, providing efficient access to new S,C-sulfonium ylides under mild conditions (Figure 1c).

## Results and Discussion

### Optimization of Reaction Conditions for Doyle–Kirmse-Type and Decarbonylation Transformations

To optimize the reaction conditions, we initially selected 3-(phenyl-λ^3^-iodaneylidene)­chromane-2,4-dione (**1a**) and allyl­(phenyl)­sulfane (**2a**) as model substrates. First, various transition metal catalysts were evaluated including [RuCl_2_(*p*-cymene)]_2_, [Cp*RhCl_2_]_2_, Cp*Co­(CO)­I_2_, Rh_2_(OAc)_4_, and Pd­(OAc)_2_. Among these, [RuCl_2_(*p*-cymene)]_2_ afforded the highest yield of the desired homoallylic sulfide product (**3a**) via a Doyle–Kirmse-type reaction and a rare decarbonation process, while other catalysts gave inferior or negligible yields (Table 1, entries 1–5). Subsequent solvent screening indicated that dichloromethane, toluene, ethyl acetate, tetrahydrofuran, methanol, and hexafluoro-2-propanol (entries 6–11) failed to significantly enhance the reaction efficiency. In addition, the use of alternative additives such as NaOAc and Cs_2_CO_3_ (entries 12 and 13) resulted in decreased yields. Increasing the catalyst loading to 5 mol % (entry 14) led to only a marginal change in yield. Under stoichiometric optimization conditions (entry 15), the use of 0.14 mmol of **1a** and 0.10 mmol of **2a** afforded a 72% yield. Notably, increasing the amount of **1a** to 0.20 mmol led to a significant improvement in the yield, reaching 81% (entry 16). These results demonstrated that the combination of [RuCl_2_(*p*-cymene)]_2_ and trifluoroethanol under air provided the most efficient conditions for this transformation.

### Substrate Scope for Ru-Catalyzed Doyle–Kirmse-Type and Decarbonylation Transformations

Upon identifying optimal conditions, we proceeded to assess the breadth of the substrate scope (Scheme 1). First, various coumarin-derived iodonium ylides **1** were reacted with allyl­(phenyl)­sulfane **2a**. Substrates bearing halogen substituents (F, Cl, and Br), as well as methyl and methoxy groups at different positions on the coumarin ring, along with a naphthyl-substituted variant, furnished the corresponding products in good yields (**3b**–**3k**). Next, a systematic investigation of allyl sulfide **2** with **1a** revealed notable substituent effects. The transformation displayed good functional group tolerance, accommodating both electron-donating groups (methoxy, methyl, isopropyl) and electron-withdrawing groups (bromo, fluoro, chloro) at various positions on the phenyl ring to deliver products **3l**–**3s** in yields ranging from 47 to 75%. The strongly electron-withdrawing nitro group was also well tolerated, affording product **3t** in 54% yield. When the phenyl group was replaced with a benzyl or methyl group, the desired products (**3w** and **3za**) were obtained in good yields. In contrast, substitution with naphthyl, pyridyl, cyclohexyl, allyl, or propyl groups led to lower yields of the corresponding homoallylic sulfides (**3u**, **3v**, **3x**–**3z**). Additionally, a nonterminal alkene was also tolerated under the reaction conditions, affording **3zb** in good yield.

**Scheme 1:**
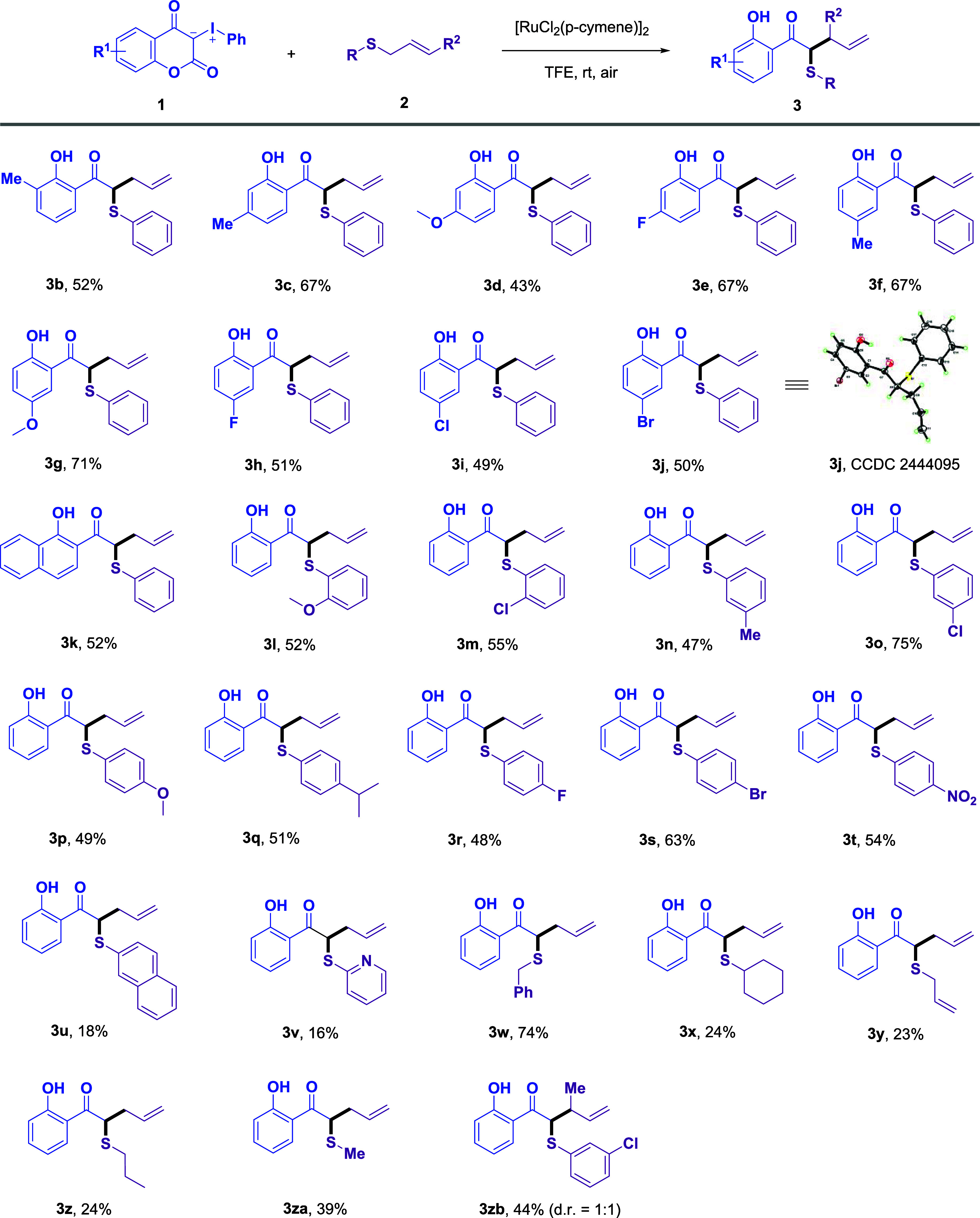
Substrate Scope of Coumarin-Derived Iodonium Ylides and Allyl Sulfides^a^

a Reaction conditions: **1** (0.4 mmol), **2** (0.2 mmol), [RuCl_2_(*p-*cymene)]_2_ (2.5 mol %), under air, TFE (3.0 mL), rt, and 12 h. Isolated yields are reported.

In addition, when 1,3-dicarbonyl-derived iodonium ylides **4** were used in place of coumarin-derived iodonium ylides, the substrates successfully underwent a Doyle–Kirmse-type reaction to afford the corresponding homoallylic sulfides **5** (Scheme 2). In general, a variety of allyl sulfides **2** exhibited good functional group tolerance, furnishing the desired products (**5a**–**5l**) in moderate to excellent yields. Functional groups such as *tert*-butyl, ester, and nitro (**5b**–**5d**) groups on the benzene ring, naphthyl, pyridyl, thienyl, benzyl, cyclohexyl, allyl, and methyl groups (**5e**–**5k**) were well tolerated. Furthermore, the methodology was compatible with a broad range of heterocyclic frameworks, including 4-hydroxy-1-methylquinolinone, pyridine-2,4­(1H,3H)-dione, and various cyclohexane-1,3-dione and cyclopentane-1,3-dione derivatives (**5m**–**5s**). Notably, noncyclic iodonium ylides were also effective under the reaction conditions, delivering products **5t** and **5u** in good yields. Additionally, the transformation showed good reactivity with allyl­(phenyl)­selane, affording product **5v** in 65% yield. The noncyclic iodonium ylide reacted with phenyl­(prop-2-yn-1-yl)­sulfane, providing the corresponding allenyl sulfide **5w** in 54% yield.

**Scheme 2:**
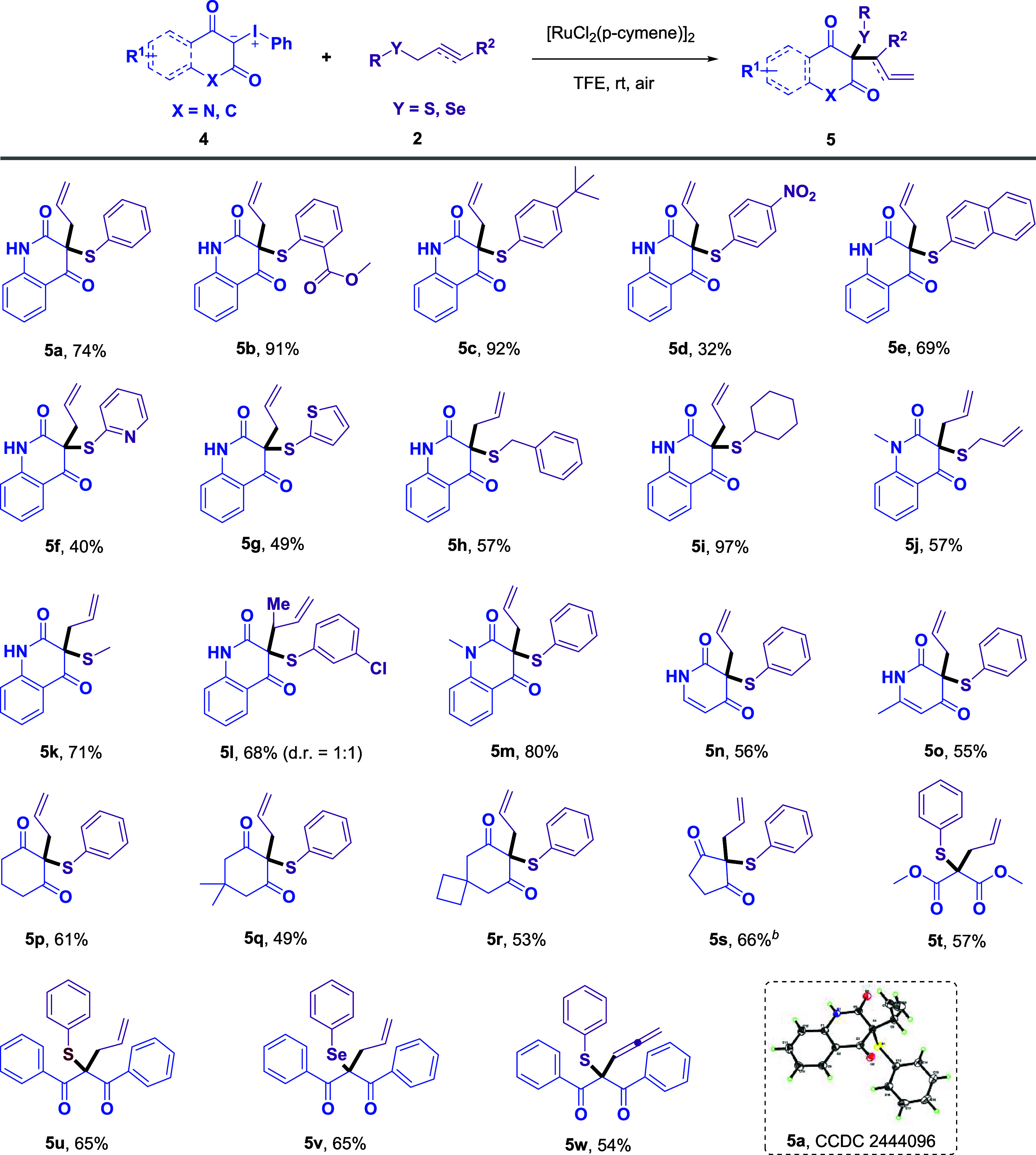
Substrate Scope of 1,3-Dicarbonyl-Derived Iodonium Ylides, Allyl Sulfides, Allyl Selenides, and Alkynyl Sulfides^a^

a Reaction conditions: **4** (0.2 mmol), **2** (0.28 mmol), [RuCl_2_(*p-*cymene)]_2_ (2.5 mol %), under air, TFE (3.0 mL), rt, and 12 h. Isolated yields are reported. ^*b*^0.1 mmol **4** and 0.14 mmol **2** were used.

### Optimization of Reaction Conditions for the Synthesis of S,C-Sulfonium Ylides

Furthermore, the reaction between *N*-pivaloyl sulfenamide **6a** and iodonium ylide **1a** for the synthesis of S,C-sulfonium ylide **7a** was also explored (see Supporting Information, Table S1). Initial optimization revealed that elevating the reaction temperature to 50 °C markedly improved the yield from 30 to 78% (Table S1, entries 1 and 2). Subsequently, a range of solvents, including DCE, EA, dioxane, and toluene, were evaluated (Table S1, entries 3–6). Among them, toluene proved to be the most effective, affording the highest yield of 88%, while the others resulted in significantly lower yields. Furthermore, replacing [RuCl_2_(*p*-cymene)]_2_ with alternative catalysts such as Cp*Co­(CO)­I_2_, [Cp*RhCl_2_]_2_, Rh_2_(esp)_2_, Pd­(OAc)_2_, or Rh_2_(OAc)_2_ led to no reaction (Table S1, entries 7–11).

With optimal conditions in hand, the substrate scope of *N*-acyl sulfenamides and iodonium ylides was explored (Scheme 3). Initially, a variety of heterocycle-derived iodonium ylides, such as coumarin, 4-hydroxynaphthalene-1,2-dione, 4-hydroxy-1-methylquinolinone, 4-hydroxy-2H-pyrido­[1,2-*a*]­pyrimidin-2-one, 4-hydroxy-pyran-2-one, and pyridine-2,4­(1H,3H)-dione, were explored. The corresponding products (**7a**–**7f**) were obtained in good yields, demonstrating good compatibility with a broad range of heterocyclic scaffolds. Additionally, the incorporation of saturated heterocyclic systems (**7g**–**7k**) yielded products in 27–98% yields. Notably, noncyclic iodonium ylide was also tolerated, affording S,C-sulfonium ylides **7l** smoothly. Next, various substituents on *N*-pivaloyl sulfenamides were explored. Both electron-donating and -withdrawing substituents on the phenyl ring were well tolerated (**7m**–**7o**, 73–94% yield). Moreover, the transformation remained effective when the phenyl group was replaced with a thiophene moiety, affording product **7p**. Substitution of the pivaloyl group with an acetyl or benzoyl group also proceeded efficiently, delivering products in good yields (**7q** and **7r**, 78–99%). Overall, these results collectively demonstrate that the ruthenium-catalyzed synthesis of S,C-sulfonium ylides exhibits a broad substrate scope and outstanding functional group tolerance.

**Scheme 3:**
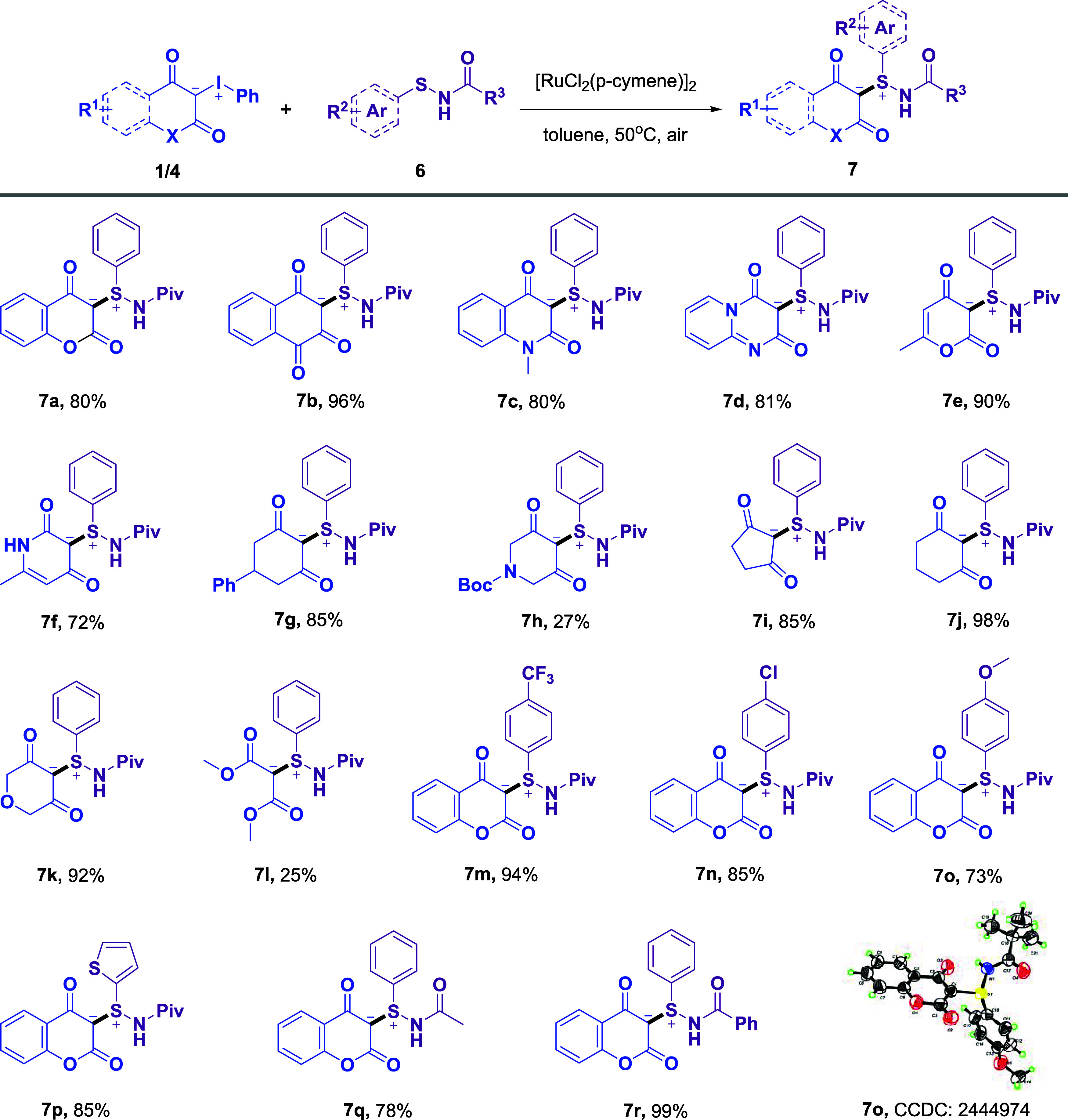
Substrate Scope of *N*-Acyl Sulfenamides and Iodonium Ylides^a^

a Reaction conditions: **1/4** (0.2 mmol), **6** (0.3 mmol), [RuCl_2_(*p-*cymene)]_2_ (3 mol %), under air, toluene (3.0 mL), 50 °C, and 12 h. Isolated yields are reported.

Subsequently, a scale-up synthesis was conducted using 3-(phenyl-λ^3^-iodaneylidene)­chromane-2,4-dione (**1a**, 4.0 mmol) and allyl­(phenyl)­sulfane (**2a**, 2.0 mmol) under standard conditions, yielding product **3a** in 66% yield (Figure 2a). Compound **3a** was treated with acetic anhydride and DMAP in DCM at room temperature provided the ester **8a** in 97% yield. In addition, **3a** was reacted with iodocyclopentane in the presence of Cs_2_CO_3_ in DMF, affording product **8b** in 43% yield. Furthermore, tunable oxidation with *m*-CPBA (3 or 5 equiv) selectively afforded compounds **8c** and **8d**, respectively. These results highlight the functional group transformation potential of compound **3**, enabling the synthesis of structurally complex molecules bearing diverse functionalities.

**Figure 2:**
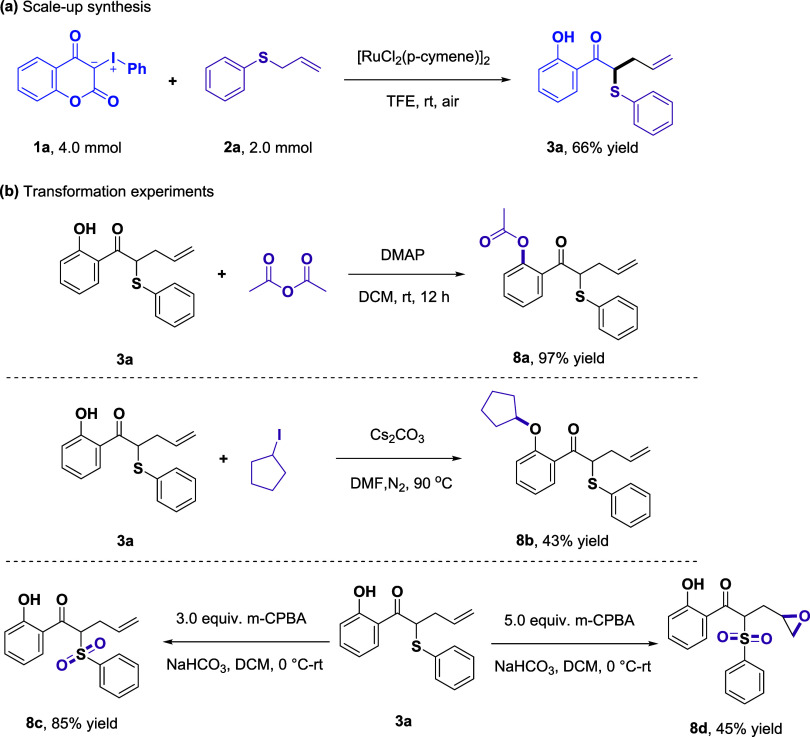
Scale-up synthesis and transformation experiments: (a) scale-up synthesis and (b) transformation experiments.

In addition, the reaction was carried out with the inclusion of radical scavengers, such as TEMPO or BHT, and the desired product **5a** was still obtained in good yields. This observation suggests that a radical-mediated process is unlikely (Figure 3a). Based on preliminary mechanistic studies and relevant literature,^14,52,53^ a plausible catalytic cycle is proposed (Figure 3b). In this mechanism, iodonium ylide **1/4** undergoes decomposition by the ruthenium catalyst to form ruthenium–carbene intermediate **A**. This intermediate subsequently reacts with allyl sulfide **2** or amide sulfides **6** to generate metal-associated zwitterionic intermediate **B**. This intermediate subsequently converts into sulfonium ylide **7**, while simultaneously regenerating the active catalyst. When R is an allyl group, a [2,3]-sigmatropic rearrangement occurs, leading to the formation of compound **5**. When X = O, this rearrangement is followed by a unique decarbonylation process, ultimately yielding homoallylic sulfide **3**.

**Figure 3:**
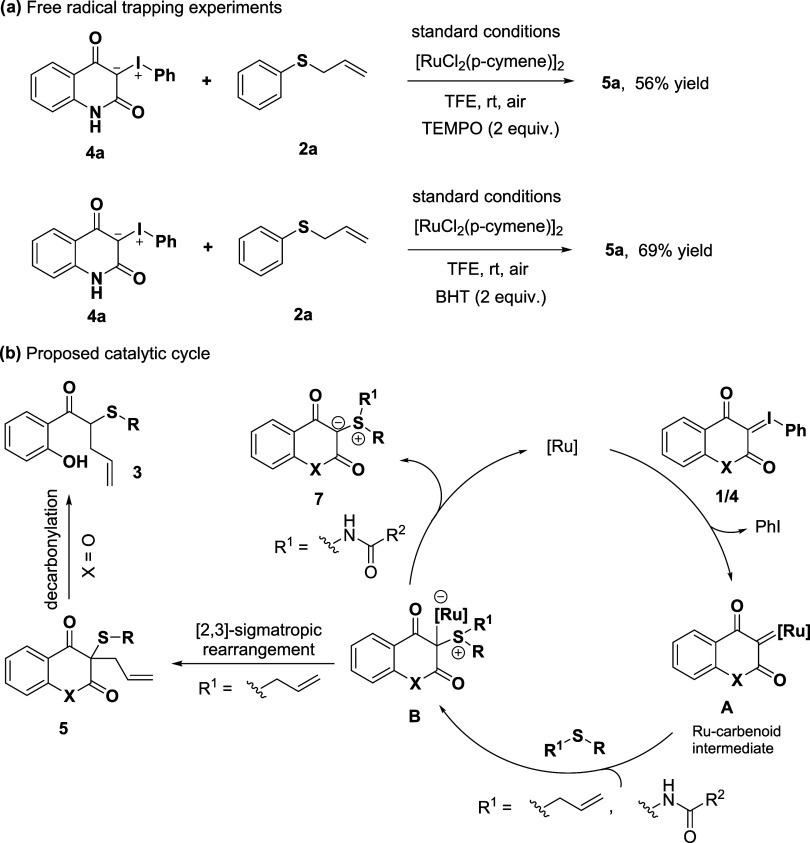
Mechanistic studies: (a) free radical trapping experiments and (b) proposed catalytic cycle.

## Conclusion

In summary, we disclose an unprecedented ruthenium-catalyzed Doyle–Kirmse-type transformation employing iodonium ylides. This strategy proceeds via a rare decarbonylation pathway to deliver structurally novel homoallylic and allenic sulfides and selenides. Moreover, this work also establishes a ruthenium-catalyzed transylidation process that couples *N*-acyl sulfenamides with cyclic iodonium ylides, affording diverse S,C-sulfonium ylides. These methods showcase broad applicability to various substrates, good tolerance for functional groups, mild reaction conditions, and scalability while employing an inexpensive, commercially available ruthenium catalyst. Scale-up experiments and downstream derivatizations underscore the synthetic utility of this method. Importantly, this work extends the Doyle–Kirmse reaction beyond classical diazo compounds, offering a practical and safe alternative for carbon–sulfur, carbon–selenium, and carbon–carbon bond formation, while simultaneously providing a valuable route for the synthesis of diverse sulfonium ylides, with potential applications in organic synthesis. By leveraging the unique reactivities of iodonium ylides, these transformations not only broaden the application scope of hypervalent iodine reagents but also harness ruthenium catalysis and coumarin-derived iodonium ylides to enable both rearrangement and decarbonylation.

## Supplementary Material









## Data Availability

The data underlying this study are available in the published article and its Supporting Information.

## Notes

General reaction procedures, characterization data of products, and NMR spectra of all products (PDF)
X-ray crystallographic data for **3j** (CIF)
X-ray crystallographic data for **5a** (CIF)
X-ray crystallographic data for **7o** (CIF)
