# Supplementary material for: Divergent Synthesis of Homoallylic and Allenic Sulfides, Selenides, and S,C-Sulfonium Ylides by Ruthenium Catalysis
Source: Precis Chem. 2025 Nov 14;4(3):219–28. doi: 10.1021/prechem.5c00098 (PMC13014341; doi:10.1021/prechem.5c00098)
Supplement: Supplementary file 1 [file pc5c00098_si_001.pdf]

Supporting Information

Divergent Synthesis of Homoallylic and Allenic Sulfides, Selenides, and S,C-Sulfonium Ylides by Ruthenium Catalysis

Zhiyue He,[a,b,e,#] Li Wu,[b,#] Yanli Ma,[b,c] Liting Wen,[b,d] Xueqing Liu,[b,d] Wenqian Ding,[b,d] Wanqi Hu,[b,c] and Xiaowei Wu[a,b,c,e]*

[a]*Drug Discovery and Development Center, Shanghai Institute of Materia Medica, Chinese Academy of Sciences, Shanghai 201203, China*

[b]*Zhongshan Institute for Drug Discovery, Shanghai Institute of Materia Medica, Chinese Academy of Sciences, Zhongshan 528400, China*

[c]*School of Chinese Materia Medica, Nanjing University of Chinese Medicine, Nanjing 210023, China*

[d]*Guangzhou University of Chinese Medicine, Guangdong 510006, China*

[e]*University of Chinese Academy of Sciences, Beijing 100049, China*

[#]*These authors contributed equally.*

**Email: X. W. (wuxiaowei@simm.ac.cn)*

I. General Information 2

II. Preparation of Starting Materials 2

III. Optimization of Reaction Conditions 7

IV. General Procedure for synthesizing products **3**, **5** and **7** 8

V. Characterization Data of Products **3** 9

VI. Characterization Data of Products **5** 18

VII. Characterization Data of Products **7** 25

VIII. Scale-up Preparation and Transformation Experiments 30

IX. Characterization Data of Products **8** 32

X. Mechanism Study 34

XI. X-ray Crystallographic Data 36

XII. References 40

XIII. NMR Spectra 41

I. *General Information*

Unless otherwise specified, the chemical reagents were purchased from commercial sources and used directly without purification. Analytical thin-layer chromatography (TLC): HSGF 254 (0.15-0.2 mm thickness). Detection was conducted under UV light at 254 nm. Preparative thin layer chromatography was HSFG 254 (0.4-0.5 mm thickness). ^{1}H, ^{13}C, and ^{19}F NMR spectra were collected on a Brucker 500 MHz or 600 MHz instrument in chloroform-*d*, Methanol-d_4 or DMSO-d_6. Chemical shifts (δ) are expressed as parts per million (ppm). Proton coupling patterns were recorded as singlet (s), broad (br), doublet (d), triplet (t), quartet (q), and multiplet (m). HRMS (high-resolution mass) were measured on a spectrometer with an electrospray ionization (ESI) source. Single crystals of **3j**, **5a,** and **7o** were grown by slow diffusion of methanol/hexane or dichloromethane/hexane (1/9, v/v, 10 mL) in a sample bottle at room temperature. X-ray diffractions of single crystals (**3j**, **5a**, and **7o**) were carried out on a Bruker D8 VENTURE diffractometer using MoKα radiation (λ = 0.71073 Å).

II. *Preparation of Starting Materials*

(1) General procedure A for the preparation of 1a-1j[1]:

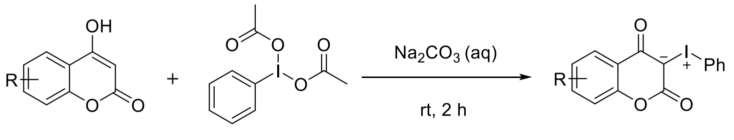

S1 **1**

PhI(OAc)$_2$ (6.2 mmol) was added to 62.0 mL of a saturated sodium carbonate aqueous solution and stirred at room temperature for 30 minutes. Subsequently, 62.0 mL of a saturated sodium carbonate aqueous solution containing 4-hydroxycoumarin compounds (6.2 mmol) was added. The reaction mixture was stirred for an additional 2 hours at room temperature and then cooled in an ice bath. The reaction mixture was filtered, and the resulting solid was washed with water to afford the product.

General procedure for the preparation of 1k[1, 2]

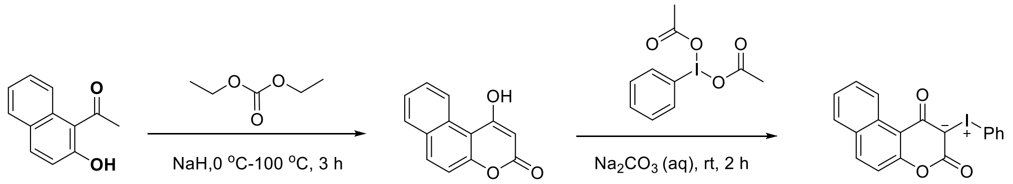

Step 1: A solution of 1-(2-hydroxynaphthalen-1-yl)ethan-1-one in diethylcarbonate (1 g/5 mL) was added dropwise to a suspension of NaH (60% dispersion in mineral oil) in diethylcarbonate (1g/5 mL) at 0 °C. The resulting mixture was heated at 100 °C for 3 h when it was cooled first to rt and then to 0 °C. Water was then carefully added dropwise to quench residual NaH. The remaining diethylcarbonate was extracted into diethyl ether. The aqueous phase was carefully acidified to pH 3 with 2 N HCl (significant foaming occurred). The resulting precipitated solid was collected by filtration, washed sequentially with water followed by petroleum ether.

Step 2: $PhI(OAc)_2$ (2.3 mmol) was added to 47.0 mL of a saturated sodium carbonate aqueous solution and stirred at room temperature for 30 minutes. Subsequently, 47.0 mL of a saturated sodium carbonate aqueous solution containing 4-hydroxycoumarin compounds (2.4 mmol) was added. The reaction mixture was stirred for an additional 2 hours at room temperature and then cooled in an ice bath. The reaction mixture was filtered, and the resulting solid was washed with water to afford the product as a white precipitate.

(2) General procedure B for the preparation of 2b-2n and 2r-2u [3]:

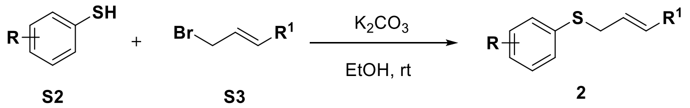

Add allyl bromide (2.14 mmol, 1.0 equiv.), K_2CO_3 (6.42 mmol, 3.0 equiv.) to the benzenethiol (2.57 mmol, 1.2 equiv.) in EtOH at room temperature with constant stirring overnight. H_2O (10 mL) was added and the aqueous layer was extracted with DCM (20 mL × 3). The combined organic phase was dried over anhydrous Na_2SO_4, filtered, and evaporated under reduced pressure. The products are purified by

preparative thin layer chromatography (PE).

General procedure for the preparation of 2w[4]

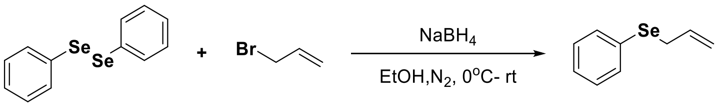

To a suspension of $NaBH_4$ (2.0 equiv.) in EtOH (5 mL) was added a solution of diselenide (1.92 mmol, 1.0 equiv.) in THF (10 mL) dropwise under a nitrogen atmosphere. The mixture was kept under agitation at 0 ºC and allyl bromide (3.84 mmol, 2.0 equiv.) in THF (5 mL) was added. After 25 minutes, the reaction mixture was quenched with water and the organic phase was extracted with diethyl ether. The organic layers were combined, dried over anhydrous Na_2SO_4, and concentrated under vacuum. The product was purified by preparative thin layer chromatography (PE).

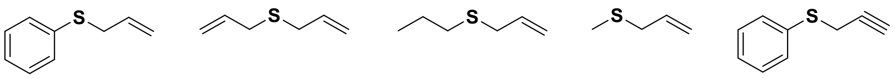

2a 2o 2p 2q 2v

Substrate **2a**, **2o**, **2p**, **2q** and **2v** were commercially available and used directly without purification.

(3) General procedure C for synthesizing compounds 4a-4d[1]:

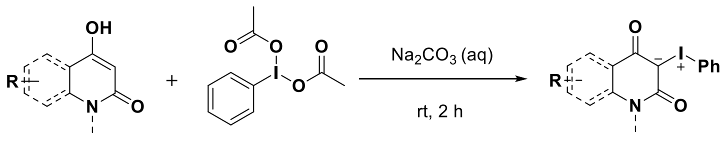

S4 4

$PhI(OAc)_2$ (6.2 mmol) was added to 62.0 mL of a saturated sodium carbonate aqueous solution and stirred at room temperature for 30 minutes. Subsequently, 62.0 mL of a saturated sodium carbonate aqueous solution containing 4-hydroxycoumarin compounds (6.2 mmol) was added. The reaction mixture was stirred for an additional 2 hours at room temperature and then cooled in an ice bath. The reaction mixture was filtered, and the resulting solid was washed with water to afford the product.

General procedure for the preparation of 4e-4h[5]:

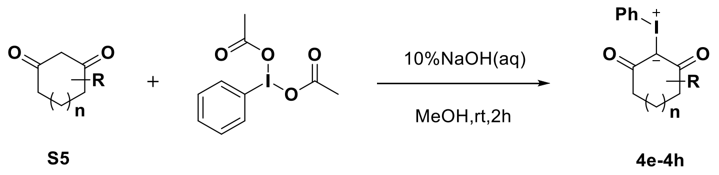

To a solution of cyclic 1,3-dione (14 mmol) in 30 mL MeOH, added at room temperature, 20 mL of a 10% aqueous solution of NaOH, followed by addition of a solution of PhI(OAc)$_2$ (15 mmol) in 40 mL MeOH. The reaction mixture was stirred for 2 h at room temperature and then quenched with ice water. The resulting white precipitate was filtered, and filtrate was extracted with DCM, then washed with water, dried over anhydrous Na_2SO_4, filtered and concentrated in vacuum. The resultant white solid was mixed with the first crop and the mixture was recrystallized from DCM/hexanes.

General procedure for the preparation of 4i-4j[6, 7]:

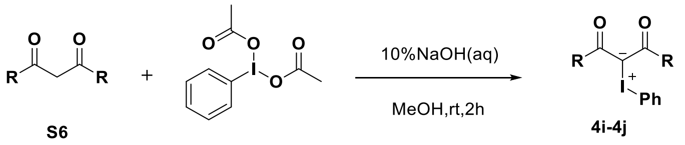

To an oven-dried 100 ml round bottom flask, **S6** (7.2 mmol, 1.15 g, 1.2 equiv.) and MeOH (20 mL) were subjected and kept the solution at room temperature. Then, 20 mL of 10% aqueous solution of KOH solution was added to the reaction mixture and continued stirring for 10 minutes. Further, in another flask, PhI(OAc)$_2$ (6.0 mmol, 1.93g, 1.0 equiv.) was taken and dissolved in MeOH (20 mL), and slowly added this solution to the above reaction mixture via syringe. The reaction mixture was stirred for 2 h at room temperature and then quenched with ice cold water. The resulting white precipitate was filtered, and filtrate was extracted with DCM, then washed with water, dried over anhydrous Na_2SO_4, filtered and concentrated in vacuum. The resultant solid was mixed with the first crop and the mixture was recrystallized from DCM/hexanes.

(4) General procedure for the preparation of 6a-6d[8]:

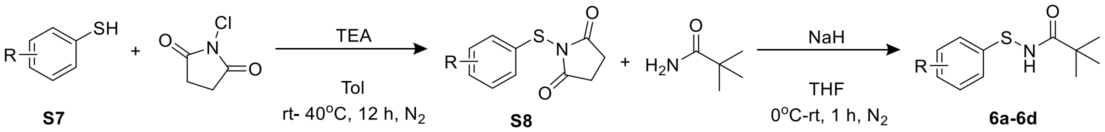

Step 1: To a stirred solution of NCS (3.0 mmol, 1.0 equiv.) in methylene chloride (0.4 M) under nitrogen at room temperature was added the indicated thiol (**S7**) (3.0 mmol, 1.0 equiv.). Then, TEA (6.0 mmol, 2.0 equiv.) was added dropwise. The resulting reaction mixture was brought to 40 °C and stirred for 12 h. After completion, the reaction mixture was diluted with saturated aqueous NH_4Cl solution. The organic layer was separated, and the aqueous layer was extracted with methylene chloride (3x). The combined organic layers were washed with brine, dried over sodium sulfate, and concentrated. The resulting crude residue was purified to afford **S8**.

Step 2: Pivalamide (3.3 mmol, 1.1 equiv.) was added to a flame dried round bottom flask under nitrogen. THF (0.15 M) was added, and the solution was cooled to 0 °C, then NaH (7.5 mmol, 2.5 equiv.) was added, and the solution was stirred for 30 minutes at 0 °C. Then the solution of **S8** (3.0 mmol, 1.0 equiv.) in THF (0.2 M) was added dropwise in the solution over one hour. Upon completion of the addition, the reaction mixture was stirred at room temperature for 1 h, then the reaction mixture was diluted with saturated aqueous ammonium chloride and extracted with ethyl acetate. The combined organic layers were washed with brine, dried over sodium sulfate, and concentrated. The crude residue was purified to afford desired **6a-6d**.

General procedure for the preparation of 6e-6g[8]:

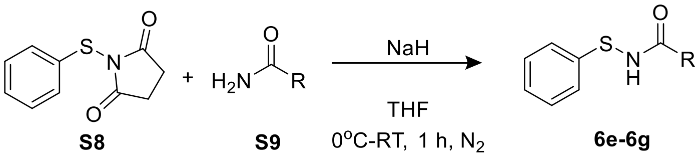

S9 (3.3 mmol, 1.1 equiv.) was added to a flame dried round bottom flask under nitrogen. THF (0.15 M) was added, and the solution was cooled to 0 °C, then NaH (7.5 mmol, 2.5 equiv.) was added, and the solution was stirred for 30 minutes at 0 °C.

Then the solution of **S8** (3.0 mmol, 1.0 equiv.) in THF (0.2 M) was added dropwise in the solution over one hour. Upon completion of the addition, the reaction mixture was stirred at room temperature for 1 h, then the reaction mixture was diluted with saturated aqueous ammonium chloride and extracted with ethyl acetate. The combined organic layers were washed with brine, dried over sodium sulfate, and concentrated. The crude residue was purified to afford **6e-6g**.

III. *Optimization of Reaction Conditions*

Table S1. Optimization of reaction conditions[a]

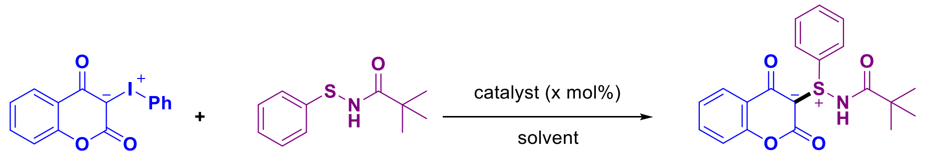

Entry	Catalyst	T (°C)	Solvent	Yield (%)
1	$[RuCl_2(p\text{-cymene})]_2$	rt	TFE	30
2	$[RuCl_2(p\text{-cymene})]_2$	50	TFE	78
3	$[RuCl_2(p\text{-cymene})]_2$	50	DCE	80
4	$[RuCl_2(p\text{-cymene})]_2$	50	EA	75
5	$[RuCl_2(p\text{-cymene})]_2$	50	Dioxane	63
6	$[RuCl_2(p\text{-cymene})]_2$	50	Toluene	88
7	$Cp^*Co(CO)I_2$	50	Toluene	NR
8	$[Cp^*RhCl_2]_2$	50	Toluene	NR
9	$Rh_2(esp)_2$	50	Toluene	NR
10	$Rh_2(OAc)_4$	50	Toluene	NR
11	$Pd(OAc)_2$	50	Toluene	NR

[a]Reaction conditions: **1a** (0.1 mmol), **6a** (0.15 mmol), $[RuCl_2(p\text{-cymene})]_2$ (3 mol%), under air, solvent (2.0 mL), 50 °C, 12 h; isolated yields are reported.

IV. *General Procedure for synthesizing products 3, 5 and 7*

(1) General procedure for synthesizing products 3:

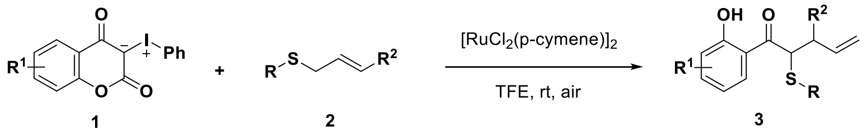

To a mixture of **1** (0.4 mmol), **2** (0.2 mmol) and $[RuCl_2(p\text{-cymene})]_2$ (2.5 mol%) in a 10 mL reaction tube was added TFE (3 mL), then the resulting mixture was stirred at room temperature for 12.0 h. After removing the solvent under vacuum, the residue was purified twice by column chromatography on silica gel (PE/EA = 20/1) to afford product **3**.

(2) General procedure for synthesizing products 5:

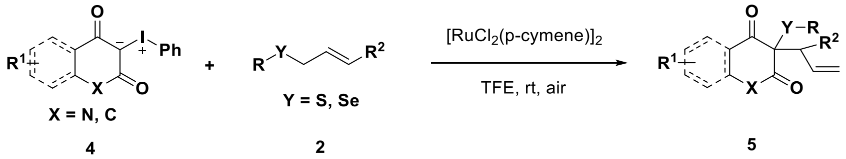

To a mixture of **4** (0.2 mmol), **2** (0.28 mmol) and $[RuCl_2(p\text{-cymene})]_2$ (2.5 mol%) in a 10 mL reaction tube was added TFE (3 mL), the resulting mixture was stirred at room temperature for 12.0 h. After removing the solvent under vacuum, the residue was purified by column chromatography on silica gel (PE/EA = 3/1) to afford **5**.

(3) General procedure for synthesizing products 7:

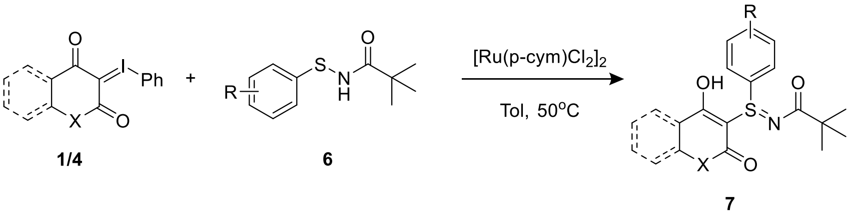

To a mixture of **1/4** (0.2 mmol), **6** (0.3 mmol) and $[RuCl_2(p\text{-cymene})]_2$ (3 mol%) in a 10 mL reaction tube was added Tol (3 mL), then the resulting mixture was stirred at 50 °C for 12.0 h. After removing the solvent under vacuum, the residue was purified by column chromatography on silica gel (PE/EA = 2/1) to afford product **7**.

V. *Characterization Data of Products 3*

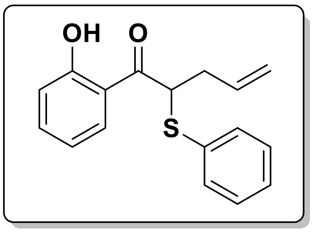

1-(2-hydroxyphenyl)-2-(phenylthio)pent-4-en-1-one (**3a**, column solvent: PE/EA = 20:1, yellow oil, 23 mg, 81%): **^{1}H NMR** (500 MHz, Chloroform-*d*) δ 12.10 (s, 1H), 7.65 (dd, *J* = 8.1, 1.6 Hz, 1H), 7.49 – 7.45 (m, 1H), 7.38 – 7.33 (m, 3H), 7.31 – 7.28 (m, 2H), 7.00 (dd, *J* = 8.4, 1.1 Hz, 1H), 6.85 – 6.82 (m, 1H), 5.91 – 5.82 (m, 1H), 5.16 – 5.09 (m, 2H), 4.55 – 4.52 (m, 1H), 2.78 – 2.72 (m, 1H), 2.63 – 2.57 (m, 1H). **^{13}C NMR** (151 MHz, Methanol-d_4) δ 202.9, 163.8, 137.4, 136.1, 135.9, 132.7, 131.6, 130.1, 130.0, 120.2, 120.0, 119.1, 118.2, 51.5, 35.9. **HRMS (ESI) *m/z*:** calculated for $C_{17}H_{16}O_2S$ [*M* + H]$^+$: 285.0944, found: 285.0947.

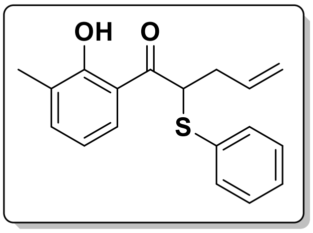

1-(2-hydroxy-3-methylphenyl)-2-(phenylthio)pent-4-en-1-one (**3b**, column solvent: PE/EA = 20:1, yellow oil, 31 mg, 52%): **^{1}H NMR** (500 MHz, Chloroform-*d*) δ 12.43 (s, 1H), 7.51 (d, J = 8.1 Hz, 1H), 7.38 – 7.33 (m, 4H), 7.31 – 7.28 (m, 2H), 6.74 (t, *J* = 7.7 Hz, 1H),5.90 – 5.82 (m, 1H), 5.15 – 5.08 (m, 2H), 4.56 (dd, *J* = 8.1, 6.6 Hz, 1H), 2.75 (dt, *J* = 14.8, 7.4 Hz, 1H), 2.60 (dt, *J* = 14.2, 6.8 Hz, 1H), 2.28 (s, 3H). **^{13}C NMR** (126 MHz, Chloroform-*d*) *δ* 201.6, 161.9, 137.4, 135.3, 134.5, 131.3, 129.2, 129.2, 127.9, 127.5, 118.3, 118.2, 117.6, 50.4, 35.1, 15.8. **HRMS (ESI) *m/z*:** calculated for $C_{14}H_{16}O_2S$ [*M* + H]$^+$: 249.0944, found: 249.0948.

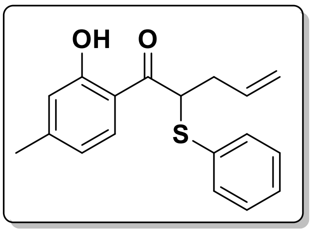

1-(2-hydroxy-4-methylphenyl)-2-(phenylthio)pent-4-en-1-one (**3c**, column solvent: PE/EA = 20:1, yellow oil, 40 mg, 67%): **^{1}H NMR** (500 MHz, Chloroform-*d*) δ 12.14 (s, 1H), 7.53 (d, *J* = 8.3 Hz, 1H), 7.38– 7.32 (m, 3H), 7.31– 7.27 (m, 2H), 6.81 (s, 1H), 6.65 (d, *J* = 8.3, 1H), 5.90– 5.82 (m, 1H), 5.15 – 5.08 (m, 2H), 4.52 – 4.49 (m, 1H), 2.78 – 2.72 (m, 1H), 2.62 – 2.56 (m, 1H), 2.35 (s, 3H). **^{13}C NMR** (126 MHz, Chloroform-*d*) *δ* 200.8, 163.5, 148.4, 135.2, 134.6, 131.3, 129.8, 129.2, 120.3, 118.9, 118.1, 116.1, 50.3, 35.1, 22.1. **HRMS (ESI) *m/z*:** calculated for $C_{14}H_{16}O_2S$ [*M* + H]$^+$: 249.0944, found: 249.0948.

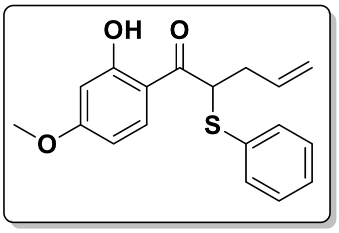

1-(2-hydroxy-4-methoxyphenyl)-2-(phenylthio)pent-4-en-1-one (**3d**, column solvent: PE/EA = 20:1, yellow oil, 27 mg, 43%): **^{1}H NMR** (500 MHz, Chloroform-*d*) δ 12.63 (s, 1H), 7.52 (d, *J* = 9.0 Hz, 1H), 7.38 – 7.37 (m, 2H), 7.34 – 7.31 (m, 1H), 7.30 – 7.26 (m, 2H), 6.43 (d, *J* = 2.4 Hz, 1H), 6.37 (dd, *J* = 9.0, 2.5 Hz, 1H), 5.89 – 5.82 (m, 1H), 5.14 – 5.07 (m, 2H), 4.46 – 4.44 (m, 1H), 3.84 (s, 3H), 2.78 – 2.73 (m, 1H), 2.61 – 2.56 (m, 1H). **^{13}C NMR** (126 MHz, Chloroform-*d***)** δ 199.8, 166.4, 166.4, 135.1, 134.6, 131.6, 131.6, 129.2, 129.1, 118.1, 112.5, 107.9, 101.2, 55.7, 50.5, 35.2. **HRMS (ESI) *m/z*:** calculated for $C_{18}H_{18}O_3S$ [*M* + H]$^+$: 315.1049, found: 315.1054.

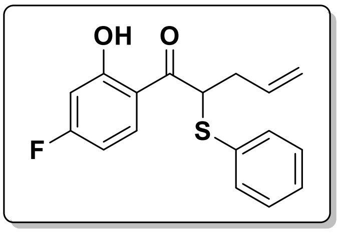

1-(4-fluoro-2-hydroxyphenyl)-2-(phenylthio)pent-4-en-1-one (**3e**, column solvent: PE/EA = 20:1, yellow oil, 41 mg, 67%): **^{1}H NMR** (500 MHz, Chloroform-*d*) δ 12.43 (d, *J* = 1.4 Hz, 1H), 7.62 (dd, *J* = 9.0, 6.3 Hz, 1H), 7.37 – 7.34 (m, 3H), 7.31 – 7.28 (m, 2H), 6.67 (dd, *J* = 10.3, 2.6 Hz, 1H), 6.53 (td, *J* = 8.5, 2.6 Hz, 1H), 5.90 – 5.82 (m, 1H), 5.16 – 5.09 (m, 2H), 4.45 – 4.42 (m, 1H), 2.78 – 2.72 (m, 1H), 2.63 – 2.57 (m, 1H). **^{13}C NMR** (151 MHz, Chloroform-*d***)** δ 200.3, 167.6 (d, *J* = 257.1 Hz), 165.9 (d, *J* = 14.1 Hz), 135.4, 134.4, 132.4 (d, *J* = 11.7 Hz), 131.0, 129.4, 129.3, 118.4, 115.5, 107.3 (d, *J* = 22.4 Hz), 105.3 (d, *J* = 23.5 Hz), 50.7, 34.9. **^{19}F NMR** (565 MHz, Chloroform-*d***)** *δ* -98.9. **HRMS (ESI) *m/z*:** calculated for $C_{17}H_{15}FO_2S$ [*M* + H]$^+$: 303.0850, found: 303.0850.

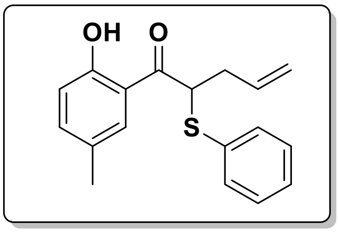

1-(2-hydroxy-5-methylphenyl)-2-(phenylthio)pent-4-en-1-one (**3f**, column solvent: PE/EA = 20:1, yellow oil, 40 mg, 67%): **^{1}H NMR** (500 MHz, DMSO-*d*$_6$) δ 11.24 (s, 1H), 7.57 (d, *J* = 2.2 Hz, 1H), 7.35 – 7.29 (m, 6H), 6.86 (d, *J* = 8.4 Hz, 1H), 5.88 – 5.83 (m, 1H), 5.12 – 5.04 (m, 3H), 2.65 – 2.59 (m, 1H), 2.48 – 2.43 (m, 1H), 2.20 (s, 3H). **^{13}C NMR** (126 MHz, DMSO-*d*$_6$**)** δ 199.5, 158.1, 136.6, 134.9, 133.9, 131.4, 130.5, 129.0, 128.5, 127.8, 119.9, 117.6, 117.5, 50.5, 34.2, 19.9. **HRMS (ESI) *m/z*:** calculated for $C_{18}H_{18}O_2S$ [*M* + H]$^+$: 299.1100, found: 299.1104.

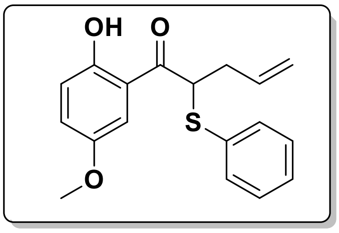

1-(2-hydroxy-5-methoxyphenyl)-2-(phenylthio)pent-4-en-1-one (**3g**, column solvent: PE/EA = 20:1, yellow oil, 45 mg, 71%): **^{1}H NMR** (500 MHz, DMSO-*d*$_6$) δ 10.95 (s, 1H), 7.34 – 7.31 (m, 5H), 7.23 (d, *J* = 3.1 Hz, 1H), 7.12

(dd, J = 8.9, 3.2 Hz, 1H), 6.91 (d, J = 9.0 Hz, 1H), 5.90 – 5.82 (m, 1H), 5.17 (t, J = 7.1 Hz, 1H), 5.12 – 5.05 (m, 2H), 3.67 (s, 3H), 2.66 – 2.60 (m, 1H), 2.47 – 2.43 (m, 1H). **^{13}C NMR** (126 MHz, DMSO-d_6**)** δ 198.7, 154.1, 151.7, 134.9, 133.7, 131.4, 129.1, 128.5, 123.2, 120.5, 118.7, 117.6, 113.1, 55.6, 50.8, 34.2. **HRMS (ESI) *m/z*:** calculated for $C_{18}H_{18}O_3S$ [M + H]$^+$: 315.1049, found: 315.1052.

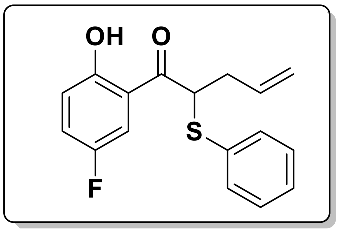

1-(5-fluoro-2-hydroxyphenyl)-2-(phenylthio)pent-4-en-1-one (**3h**, column solvent: PE/EA = 20:1, yellow oil, 31 mg, 51%): **^{1}H NMR** (500 MHz, DMSO-d_6) δ 11.12 (s, 1H), 7.56 (dd, J = 9.6, 3.2 Hz, 1H), 7.37 – 7.28 (m, 6H), 6.98 (dd, J = 9.1, 4.5 Hz, 1H), 5.90 – 5.81 (m, 1H), 5.13 – 5.05 (m, 3H), 2.63 – 2.57 (m, 1H), 2.45 – 2.40 (m, 1H). **^{13}C NMR** (126 MHz, DMSO-d_6**)** δ 197.4, 155.6, 154.1, 134.8, 134.1, 130.8, 129.1, 128.7, 122.4 (d, J = 23.5 Hz), 121.6 (d, J = 6.5 Hz), 119.1 (d, J = 7.4 Hz), 117.6, 115.8 (d, J = 24.2 Hz), 51.2, 33.8. **^{19}F NMR** (471 MHz, DMSO-d_6) δ -124.5. **HRMS (ESI) *m/z*:** calculated for $C_{17}H_{15}FO_2S$ [M + H]$^+$: 303.0850, found: 303.0853.

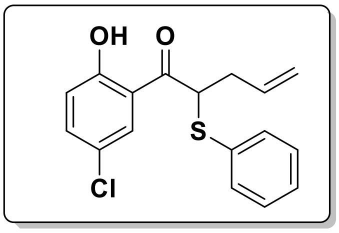

1-(5-chloro-2-hydroxyphenyl)-2-(phenylthio)pent-4-en-1-one (**3i**, column solvent: PE/EA = 20:1, yellow solid, 31 mg, 49%): **^{1}H NMR** (500 MHz, DMSO-d_6) δ 11.32 (s, 1H), 7.72 (d, J = 2.7 Hz, 1H), 7.49 (dd, J = 8.8, 2.7 Hz, 1H), 7.35 – 7.28 (m, 5H), 6.99 (d, J = 8.8 Hz, 1H), 5.90 – 5.82 (m, 1H), 5.12 – 5.06 (m, 3H), 2.63 – 2.57 (m, 1H), 2.45 – 2.39 (m, 1H). **^{13}C NMR** (151 MHz, DMSO-d_6**)** δ 197.0, 157.7, 134.9, 134.6, 134.1, 130.9, 129.7, 129.1, 128.7, 122.9, 122.9, 119.5, 117.6, 51.4, 33.8. **HRMS (ESI) *m/z*:** calculated for $C_{17}H_{15}ClO_2S$ [M + H]$^+$: 319.0554, found: 319.0559.

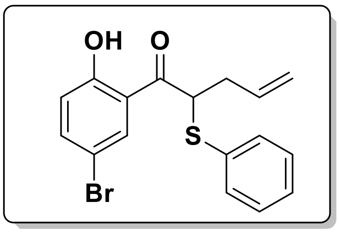

1-(5-chloro-2-hydroxyphenyl)-2-(phenylthio)pent-4-en-1-one (**3j**, column solvent: PE/EA = 20:1, yellow solid, 36 mg, 50%): **^{1}H NMR** (500 MHz, DMSO-d_6) δ 11.33 (s, 1H), 7.82 (d, J = 2.6 Hz, 1H), 7.60 (dd, J = 8.8, 2.6 Hz, 1H), 7.35 – 7.28 (m, 5H), 6.93 (d, J = 8.8 Hz, 1H), 5.90 – 5.82 (m, 1H), 5.12 – 5.05 (m, 3H), 2.63 – 2.57 (m, 1H), 2.45 – 2.39 (m, 1H). **^{13}C NMR** (126 MHz, DMSO-d_6**)** δ 197.0, 158.1, 137.3, 134.9, 134.0, 132.6, 130.9, 129.1, 128.7, 123.5, 119.9, 117.6, 110.4, 51.5, 33.8. **HRMS (ESI) *m/z*:** calculated for $C_{17}H_{15}BrO_2S$ [M + H]$^+$: 363.0049, found: 363.0054.

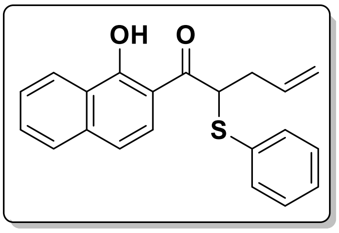

1-(1-hydroxynaphthalen-2-yl)-2-(phenylthio)pent-4-en-1-one (**3k**, column solvent: PE/EA = 20:1, brown solid, 31 mg, 52%): **^{1}H NMR** (500 MHz, Chloroform-d) δ 11.38 (s, 1H), 8.00 (d, *J* = 8.4 Hz, 1H), 7.81 (d, *J* = 9.0 Hz, 1H), 7.72 (dd, *J* = 8.1, 1.3 Hz, 1H), 7.47 – 7.43 (m, 1H), 7.37 – 7.33 (m, 1H), 7.31 – 7.29 (m, 2H), 7.22 – 7.20 (m, 1H), 7.18 – 7.15 (m, 2H), 7.08 (d, *J* = 9.0 Hz, 1H), 5.86 – 5.79 (m, 1H), 5.19 – 5.15 (m, 1H), 5.08 (d, *J* = 10.3 Hz, 1H), 4.91 – 4.88 (m, 1H), 2.97 – 2.91 (m, 1H), 2.73 – 2.67 (m, 1H). **^{13}C NMR** (126 MHz, Chloroform-d**)** δ 203.2, 161.2, 136.8, 134.1, 134.0, 132.0, 131.4, 129.3, 128.9, 128.70, 128.5, 128.1, 124.8, 124.1, 119.3, 118.6, 116.0, 55.1, 36.8. **HRMS (ESI) *m/z*:** calculated for $C_{21}H_{18}O_2S$ [*M* + H]$^+$: 355.1100, found: 355.1104.

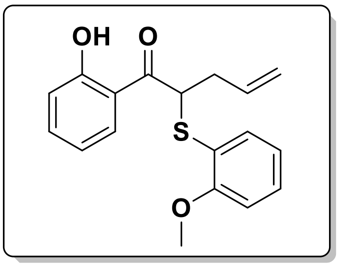

1-(2-hydroxyphenyl)-2-((2-methoxyphenyl)thio)pent-4-en-1-one (**3l**, column solvent: PE/EA = 20:1, yellow oil, 33 mg, 52%): **^{1}H NMR** (600 MHz, Chloroform-d) δ 12.20 (s, 1H), 7.65 (dd, *J* = 8.1, 1.6 Hz, 1H), 7.45 – 7.42 (m, 1H), 7.40 (dd, *J* = 7.6, 1.7 Hz, 1H), 7.34 – 7.31 (m, 1H), 6.98 (dd, *J* = 8.4, 1.2 Hz, 1H), 6.88 – 6.85 (m, 1H), 6.83 – 6.78 (m, 2H), 5.89 – 5.83(m, 1H), 5.13 – 5.10 (m, 1H), 5.07 – 5.05 (m, 1H), 4.60 – 4.58 (m, 1H), 3.69 (s, 3H), 2.79 – 2.73 (m, 1H), 2.62 – 2.57 (m, 1H). **^{13}C NMR** (151 MHz, Chloroform-d**)** δ 202.1, 163.1, 160.3, 137.7, 136.4, 134.8, 131.4, 129.9, 120.9, 119.0, 118.9, 118.8, 118.5, 117.8, 111.1, 55.5, 49.1, 35.0. **HRMS (ESI) *m/z*:** calculated for $C_{18}H_{18}O_3S$ [*M* + H]$^+$: 315.1049, found: 315.1052.

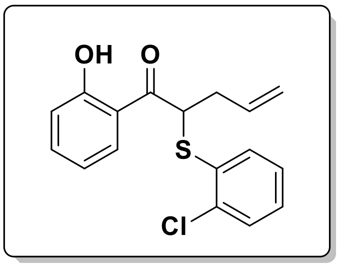

2-((2-chlorophenyl)thio)-1-(2-hydroxyphenyl)pent-4-en-1-one (**3m**, column solvent: PE/EA = 20:1, yellow oil, 36 mg, 55%): **^{1}H NMR** (500 MHz, Chloroform-d) δ 12.13 (s, 1H), 7.51 (dd, *J* = 8.1, 1.6 Hz, 1H), 7.44 – 7.39 (m, 3H), 7.26 – 7.22 (m, 1H), 7.12 (td, *J* = 7.6, 1.4 Hz, 1H), 6.96 (dd, *J* = 8.4, 1.1 Hz, 1H), 6.76 – 6.73 (m, 1H), 5.88 – 5.80 (m, 1H), 5.15 – 5.06 (m, 2H), 4.74 (dd, *J* = 8.5, 5.9 Hz, 1H), 2.88 – 2.81 (m, 1H), 2.70 – 2.64 (m, 1H). **^{13}C NMR** (151 MHz, Chloroform-d**)** δ 201.8, 163.3, 139.0, 136.8, 136.7, 134.1, 131.1, 130.4, 130.4, 129.7, 127.4, 118.9, 118.8, 118.7, 118.4, 49.5, 35.3. **HRMS (ESI) *m/z*:** calculated for $C_{17}H_{15}ClO_2S$ [*M* + H]$^+$: 319.0554, found: 319.0557.

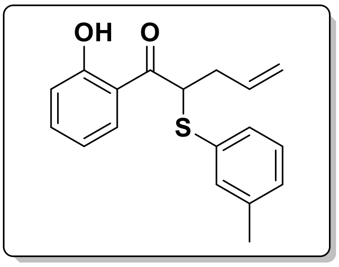

1-(2-hydroxyphenyl)-2-(m-tolylthio)pent-4-en-1-one (**3n**, column solvent: PE/EA = 20:1, yellow oil, 29 mg, 47%): **^{1}H NMR** (500 MHz, Chloroform-d) δ 12.11 (s, 1H), 7.64 (dd, *J* = 8.1, 1.6 Hz, 1H), 7.49 – 7.45 (m, 1H), 7.19 – 7.14 (m, 4H), 7.01 (dd, *J* = 8.4, 1.1 Hz, 1H), 6.85 – 6.82 (m, 1H), 5.91 – 5.83 (m, 1H), 5.16 – 5.09 (m, 2H), 4.53 (dd, *J* = 8.1, 6.5 Hz, 1H), 2.79 – 2.73 (m, 1H), 2.63 – 2.57 (m, 1H), 2.30 (s, 3H). **^{13}C NMR** (151 MHz, Chloroform-d**)** δ 201.4, 163.4, 139.0, 136.6, 136.0, 134.6, 132.3, 130.8, 130.1, 130.0, 129.0, 118.9, 118.8, 118.4, 118.2, 50.4, 35.0, 21.3. **HRMS (ESI)** ***m/z*:** calculated for $C_{18}H_{18}O_2S$ [*M* + H]$^+$: 299.1100, found: 299.1103.

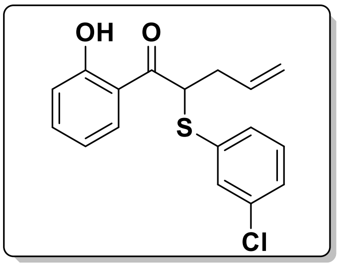

2-((3-chlorophenyl)thio)-1-(2-hydroxyphenyl)pent-4-en-1-one (**3o**, column solvent: PE/EA = 20:1, yellow oil, 48 mg, 75%): **^{1}H NMR** (500 MHz, Chloroform-*d*) δ 12.04 (s, 1H), 7.63 (d, *J* = 8.0 Hz, 1H), 7.50 – 7.47 (m, 1H), 7.38 (s, 1H), 7.33 – 7.29 (m, 1H), 7.24 – 7.20 (m, 2H), 7.01 (d, *J* = 8.4 Hz, 1H), 6.85 (t, *J* = 7.6 Hz, 1H), 5.98 – 5.81 (m, 1H), 5.17 – 5.10 (m, 2H), 4.58 – 4.56 (m, 1H), 2.80 – 2.74 (m, 1H), 2.63 – 2.57 (m, 1H). **^{13}C NMR** (126 MHz, Chloroform-*d***)** δ 201.1, 163.4, 136.9, 134.7, 134.6, 134.1, 133.2, 133.0, 130.2, 129.8, 129.4, 119.0, 119.0, 118.5, 118.2, 50.3, 35.0. **HRMS (ESI)** ***m/z*:** calculated for $C_{17}H_{15}ClO_2S$ [*M* + H]$^+$: 319.0554, found: 319.0559.

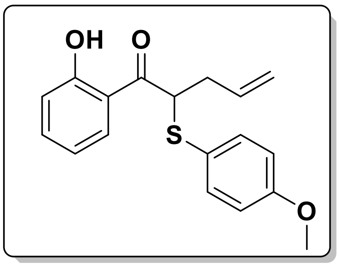

1-(2-hydroxyphenyl)-2-((4-methoxyphenyl)thio)pent-4-en-1-one (**3p**, column solvent: PE/EA = 20:1, yellow oil, 31 mg, 49%): **^{1}H NMR** (500 MHz, Chloroform-*d*) δ 12.11 (s, 1H), 7.68 (dd, *J* = 8.1, 1.6 Hz, 1H), 7.48 – 7.45 (m, 1H), 7.28 – 7.25 (m, 2H), 7.00 (dd, *J* = 8.4, 1.2 Hz, 1H), 6.87 – 6.84 (m, 1H), 6.83 – 6.80 (m, 2H), 5.90 – 5.82 (m, 1H), 5.15 – 5.07 (m, 2H), 4.41 (dd, *J* = 8.0, 6.6 Hz, 1H), 3.79 (s, 3H), 2.71 – 2.64 (m, 1H), 2.57 – 2.51 (m, 1H). **^{13}C NMR** (126 MHz, Chloroform-*d***)** δ 201.2, 163.4, 161.0, 138.0, 136.5, 134.7, 130.0, 120.8, 119.0, 118.8, 118.4, 118.0, 114.7, 55.4, 50.3, 34.4. **HRMS (ESI)** ***m/z*:** calculated for $C_{18}H_{18}O_3S$ [*M* + H]$^+$: 315.1049, found: 315.1053.

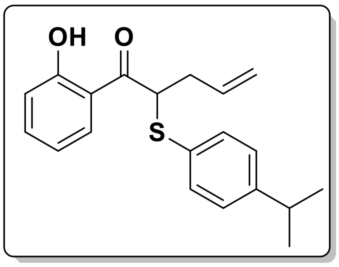

1-(2-hydroxyphenyl)-2-((4-isopropylphenyl)thio)pent-4-en-1-one (**3q**, column solvent: PE/EA = 20:1, yellow oil, 33 mg, 51%): **^{1}H NMR** (600 MHz, DMSO-d_6) δ 11.39 (s, 1H), 7.81 (d, *J* = 9.6 Hz, 1H), 7.50 – 7.47 (m, 1H), 7.22 – 7.19 (m, 4H), 6.96 (d, *J* = 8.3 Hz, 1H), 6.90 (t, *J* = 7.5 Hz, 1H), 5.88 – 5.82 (m, 1H), 5.11 – 5.03 (m, 3H), 2.89 – 2.84 (m, 1H), 2.62 – 2.57 (m, 1H), 2.45 – 2.40 (m, 1H), 1.17 (d, *J* = 6.9 Hz, 6H). **^{13}C NMR** (151 MHz, DMSO-d_6) δ 199.4, 160.0, 149.2, 135.6, 135.0, 134.6, 130.8, 127.6, 127.1, 120.6, 119.2, 117.6, 117.6, 50.6, 34.0, 33.1, 23.7, 23.6. **HRMS (ESI) *m/z*:** calculated for $C_{20}H_{22}O_2S$ [*M* + H]$^+$: 327.1413, found: 327.1417.

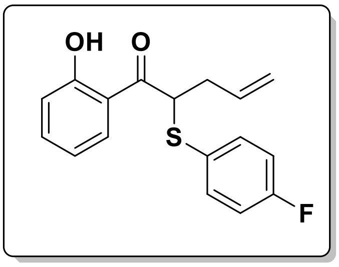

2-((4-fluorophenyl)thio)-1-(2-hydroxyphenyl)pent-4-en-1-one (**3r**, column solvent: PE/EA = 20:1, yellow oil, 28 mg, 48%): **^{1}H NMR** (500 MHz, DMSO-d_6) δ 11.35 (s, 1H), 7.83 (dd, *J* = 8.0, 1.6 Hz, 1H), 7.50 – 7.48 (m, 1H), 7.35 – 7.32 (m, 2H), 7.18 (t, *J* = 8.7 Hz, 2H), 6.96 (d, *J* = 8.3 Hz, 1H), 6.92 (t, *J* = 7.5 Hz, 1H), 5.88 – 5.81 (m, 1H), 5.11 – 5.05 (m, 3H), 2.61 – 2.56 (m, 1H), 2.44 – 2.40 (m, 1H). **^{13}C NMR** (151 MHz, DMSO-d_6) δ 199.1, 162.6 (d, *J* = 246.5 Hz), 159.9, 137.2 (d, *J* = 9.2 Hz), 135.6, 134.9, 130.8, 126.2 (d, *J* = 3.4 Hz), 120.6, 119.2, 117.7, 117.6, 116.2 (d, *J* = 22.2 Hz), 50.8, 33.8. **^{19}F NMR** (471 MHz, DMSO-d_6) *δ* -112.4. **HRMS (ESI) *m/z*:** calculated for $C_{17}H_{15}FO_2S$ [*M* + H]$^+$: 303.0850, found: 303.0851.

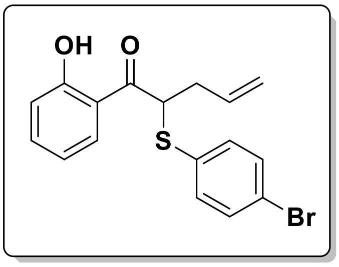

2-((4-bromophenyl)thio)-1-(2-hydroxyphenyl)pent-4-en-1-one (**3s**, column solvent: PE/EA = 20:1, yellow oil, 37 mg, 63%): **^{1}H NMR** (600 MHz, DMSO-d_6) δ 11.31 (s, 1H), 7.83 (dd, *J* = 8.1, 1.6 Hz, 1H), 7.53 – 7.50 (m, 2H), 7.49 – 7.48 (m, 1H), 7.24 (dd, *J* = 8.3, 1.3 Hz, 2H), 6.96 (d, *J* = 8.3 Hz, 1H), 6.92 (t, *J* = 7.5 Hz, 1H), 5.87 – 5.80 (m, 1H), 5.15 (t, *J* = 7.1 Hz, 1H), 5.11 – 5.08 (m, 1H), 5.06 (d, *J* = 10.2 Hz, 1H), 2.64 – 2.60 (m, 1H), 2.47 – 2.42 (m, 1H). **^{13}C NMR** (151 MHz, DMSO-d_6) δ 198.9, 159.8, 135.8, 135.7, 134.7, 132.0, 130.8, 130.8, 122.2, 120.7, 119.3, 117.8, 117.6, 50.8, 34.1. **HRMS (ESI) *m/z*:** calculated for $C_{17}H_{15}BrO_2S$ [*M* + H]$^+$: 363.0049, found: 363.0053.

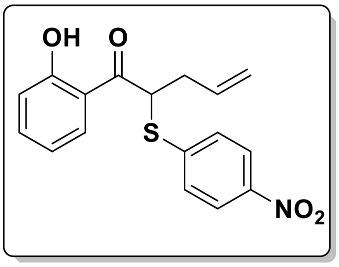

1-(2-hydroxyphenyl)-2-((4-nitrophenyl)thio)pent-4-en-1-one (**3t**, column solvent: PE/EA = 20:1, yellow oil, 36 mg, 54%): **^{1}H NMR** (500 MHz, Chloroform-*d*) δ 11.95 (s, 1H), 8.13 – 8.11 (m, 2H), 7.69 (dd, *J* = 8.2, 1.6 Hz, 1H), 7.53 – 7.49 (m, 1H), 7.48 – 7.45 (m, 2H), 7.02 (dd, *J* = 8.4, 1.2 Hz, 1H), 6.90 – 6.87 (m, 1H), 5.88 – 5.80 (m, 1H), 5.18 – 5.12 (m, 2H), 4.78 (t, *J* = 7.2 Hz, 1H), 2.88 – 2.82 (m, 1H), 2.69 – 2.63 (m, 1H). **^{13}C NMR** (126 MHz, Chloroform-*d*) δ 200.8, 163.6, 147.2, 141.9, 137.3, 133.41, 132.3, 129.5, 124.1, 119.3, 119.3, 119.1, 117.9, 50.0, 35.5. **HRMS (ESI) *m/z*:** calculated for $C_{17}H_{15}NO_4S$ [*M* + H]$^+$: 330.0795, found: 330.0793.

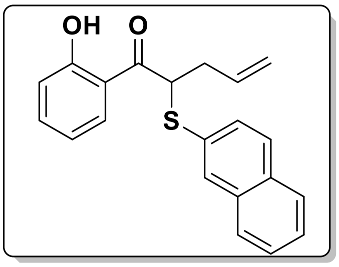

1-(2-hydroxyphenyl)-2-(naphthalen-2-ylthio)pent-4-en-1-one (**3u**, column solvent: PE/EA = 20:1, yellow oil, 12 mg, 18%): **^{1}H NMR** (500 MHz, Chloroform-*d*) δ 12.10 (s, 1H), 7.88 – 7.88 (m, 1H), 7.83 – 7.81 (m, 1H), 7.78 – 7.74 (m, 2H), 7.67 (dd, *J* = 8.1, 1.6 Hz, 1H), 7.52 – 7.49 (m, 2H), 7.47 – 7.44 (m, 1H), 7.41 (dd, *J* = 8.5, 1.9 Hz, 1H), 7.01 (dd, *J* = 8.4, 1.2 Hz, 1H), 6.81 – 6.78 (m, 1H), 5.93 – 5.85 (m, 1H), 5.17 – 5.10 (m, 2H), 4.62 (dd, *J* = 8.1, 6.5 Hz, 1H), 2.83 – 2.76 (m, 1H), 2.69 – 2.63 (m, 1H). **^{13}C NMR** (126 MHz, Chloroform-*d*) δ 201.4, 163.4, 136.7, 135.1, 134.5, 133.6, 133.3, 131.6, 129.9, 128.8, 128.5, 127.9, 127.8, 127.2, 126.8, 119.0, 118.9, 118.4, 118.3, 50.5, 35.1. **HRMS (ESI) *m/z*:** calculated for $C_{21}H_{18}O_2S$ [*M* + H]$^+$: 335.1100, found: 335.1098.

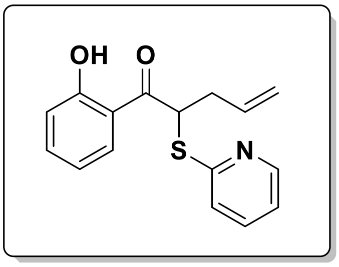

1-(2-hydroxyphenyl)-2-(pyridin-2-ylthio)pent-4-en-1-one (**3v**, column solvent: PE/EA = 20:1, yellow oil, 9 mg, 16%): **^{1}H NMR** (500 MHz, Chloroform-*d*) δ 12.14 (s, 1H), 8.42 – 8.41 (m, 1H), 7.99 (dd, *J* = 8.1, 1.6 Hz, 1H), 7.51 – 7.44 (m, 2H), 7.17 (d, *J* = 8.0 Hz, 1H), 7.04 – 7.01 (m, 1H), 6.99 – 6.97 (m, 1H), 6.85 – 6.82 (m, 1H), 5.90 – 5.79 (m, 2H), 5.17 – 5.13 (m, 1H), 5.05 (dd, *J* = 10.2, 1.7 Hz, 1H), 2.95 – 2.90 (m, 1H), 2.71 – 2.66 (m, 1H). **^{13}C NMR** (126 MHz, Chloroform-*d*) δ 203.5, 163.2, 156.3, 149.4, 136.7, 136.5, 134.2, 130.4, 122.6, 120.3, 119.1, 118.7, 118.6, 118.2, 46.0, 36.3. **HRMS (ESI) *m/z*:** calculated for $C_{16}H_{15}NO_2S$ [*M* + H]$^+$: 286.0896, found: 286.0894.

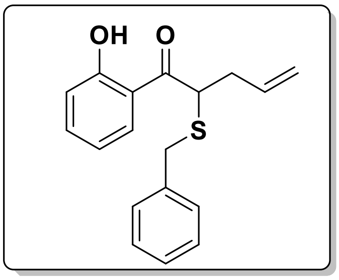

2-(benzylthio)-1-(2-hydroxyphenyl)pent-4-en-1-one (**3w**, column solvent: PE/EA = 20:1, yellow oil, 44 mg, 74%): **^{1}H NMR** (500 MHz, Chloroform-*d*) δ 12.16 (s, 1H), 7.49 – 7.44 (m, 2H), 7.29 – 7.24 (m, 5H), 6.99 (dd, *J* = 8.3, 1.1 Hz, 1H), 6.81 – 6.78 (m, 1H), 5.83 – 5.75 (m, 1H), 5.12 – 5.05 (m, 2H), 4.24 (dd, *J* = 8.2, 6.5 Hz, 1H), 3.82 – 3.69 (m, 2H), 2.88 – 2.82 (m, 1H), 2.63 – 2.56 (m, 1H). **^{13}C NMR** (126 MHz, Chloroform-*d*) δ 201.7, 163.5, 137.0, 136.7, 134.5, 129.8, 129.3, 128.8, 127.5, 119.0, 118.9, 118.1, 118.1, 46.1, 34.9, 34.7. **HRMS (ESI) *m/z*:** calculated for $C_{18}H_{18}O_2S$ [*M* + H]$^+$: 299.1100, found: 299.1102.

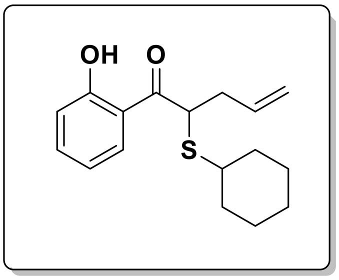

2-(benzylthio)-1-(2-hydroxyphenyl)pent-4-en-1-one (**3x**, column solvent: PE/EA = 20:1, yellow oil, 14 mg, 24%): **^{1}H NMR** (500 MHz, Chloroform-*d*) δ 12.22 (s, 1H), 7.80 (dd, *J* = 8.1, 1.6 Hz, 1H), 7.50 – 7.46 (m, 1H), 7.00 (dd, *J* = 8.4, 1.2 Hz, 1H), 6.92 – 6.88 (m, 1H), 5.86 – 5.78 (m, 1H), 5.14 – 5.04 (m, 2H), 4.33 – 4.30 (m, 1H), 2.91 – 2.85 (m, 1H), 2.80 – 2.74 (m, 1H), 2.63 – 2.57 (m, 1H), 1.94 – 1.82 (m, 2H), 1.74 – 1.54 (m, 4H), 1.37 – 1.25 (m, 4H). **^{13}C NMR** (126 MHz, Chloroform-*d*) δ 202.8, 163.4, 136.7, 134.8, 129.8, 119.0, 119.0, 118.3, 117.9, 46.3, 43.2, 36.2, 34.7, 34.5, 26.2, 26.2, 25.6. **HRMS (ESI) *m/z*:** calculated for $C_{17}H_{22}O_2S$ [*M* + H]$^+$: 291.1413, found: 291.1410.

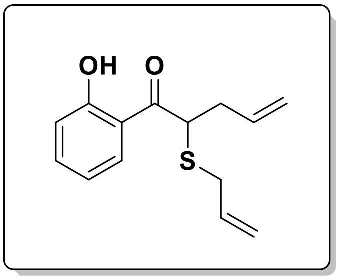

2-(allylthio)-1-(2-hydroxyphenyl)pent-4-en-1-one (**3y**, column solvent: PE/EA = 20:1, yellow oil, 12 mg, 23%): **^{1}H NMR** (500 MHz, Chloroform-*d*) δ 12.18 (s, 1H), 7.76 (dd, J = 8.1, 1.6 Hz, 1H), 7.50 – 7.47 (m, 1H), 7.01 (dd, J = 8.4, 1.2 Hz, 1H), 6.91 – 6.88 (m, 1H), 5.86 – 5.74 (m, 2H), 5.18 – 5.06 (m, 4H), 4.29 (dd, J = 8.2, 6.5 Hz, 1H), 3.26 – 3.13 (m, 2H), 2.89 – 2.82 (m, 1H), 2.63 – 2.57 (m, 1H). **^{13}C NMR** (151 MHz, Chloroform-*d*) δ 202.0, 163.5, 136.8, 134.5, 133.7, 129.8, 119.0, 119.0, 118.4, 118.3, 118.1, 45.8, 35.1, 33.4. **HRMS (ESI) *m/z*:** calculated for $C_{14}H_{16}O_2S$ [*M* + H]$^+$: 249.0944, found: 249.0949.

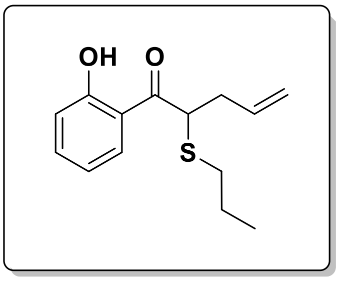

1-(2-hydroxyphenyl)-2-(propylthio)pent-4-en-1-one (**3z**, column solvent: PE/EA = 20:1, yellow oil, 12 mg, 24%): **^{1}H NMR** (500 MHz, Chloroform-*d*) δ 12.20 (s, 1H), 7.79 (dd, *J* = 8.1, 1.6 Hz, 1H), 7.49 – 7.46 (m, 1H), 7.00 (dd, *J* = 8.4, 1.2 Hz, 1H), 6.91 – 6.88 (m, 1H), 5.88 – 5.80 (m, 1H), 5.16 – 5.11 (m, 1H), 5.09 – 5.07 (m, 1H), 4.27 – 4.25 (m, 1H), 2.88 – 2.81 (m, 1H), 2.62 – 2.52 (m, 2H), 2.46 – 2.41 (m, 1H), 1.58 – 1.50 (m, 2H), 0.93 (t, *J* = 7.3 Hz, 3H). **^{13}C NMR** (126 MHz, Chloroform-*d*) δ 201.5, 163.5, 136.6, 134.7, 129.8, 119.0, 119.0, 118.1, 117.9, 45.8, 34.5, 31.3, 22.8, 13.7. **HRMS (ESI) *m/z*:** calculated for $C_{14}H_{18}O_2S$ [*M* + H]$^+$: 251.1100, found: 252.1103.

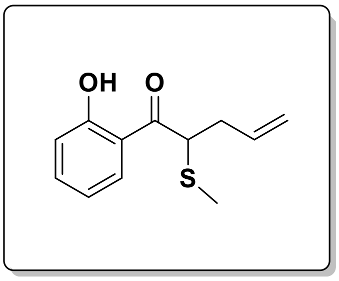

1-(2-hydroxyphenyl)-2-(methylthio)pent-4-en-1-one (**3za**, column solvent: PE/EA = 20:1, yellow oil, 17 mg, 39%): **^{1}H NMR** (500 MHz, Chloroform-*d*) δ 12.18 (s, 1H), 7.77 (dd, *J* = 8.2, 1.7 Hz, 1H), 7.49 – 7.46 (m, 1H), 7.00 (dd, *J* = 8.4, 1.1 Hz, 1H), 6.91 – 6.88 (m, 1H), 5.89 – 5.81 (m, 1H), 5.18 – 5.08 (m, 2H), 4.25 (t, *J* = 7.4 Hz, 1H), 2.85 – 2.78 (m, 1H), 2.60 – 2.54 (m, 1H), 2.02 (s, 3H). **^{13}C NMR** (126 MHz, Chloroform-*d*) δ 200.4, 163.6, 136.6, 134.5, 129.8, 119.0, 119.0, 118.0, 117.9, 45.0, 33.0, 11.5. **HRMS (ESI) *m/z*:** calculated for $C_{12}H_{14}O_2S$ [*M* + H]$^+$: 223.0787, found: 223.0790.

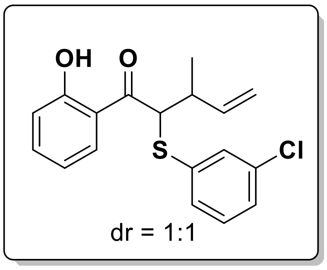

2-((3-chlorophenyl)thio)-1-(2-hydroxyphenyl)-3-methylpent-4-en-1-one (**3zb**, column solvent: PE/EA = 20:1, yellow oil, 28 mg, 44%): **^{1}H NMR** (500 MHz, Chloroform-*d*) δ 12.16 (s, 1H), 12.13 (s, 1H), 7.56 – 7.51 (m, 2H), 7.49 – 7.44 (m, 2H), 7.38 – 7.37 (m, 2H), 7.28 – 7.15 (m, 6H), 7.01 – 6.98 (m, 2H), 6.84 – 6.80 (m, 2H), 6.05 – 5.98 (m, 1H), 5.76 – 5.69 (m, 1H), 5.24 – 5.19 (m, 2H), 5.06 (dd, *J* = 17.2, 1.5 Hz, 1H), 4.97 (d, *J* = 10.4 Hz, 1H), 4.37 (d, *J* = 9.4 Hz, 2H), 2.90 (dq, *J* = 9.1, 6.8 Hz, 2H), 1.39 (d, *J* = 6.8 Hz, 3H), 1.10 (d, *J* = 6.7 Hz, 3H). **^{13}C NMR** (126 MHz, Chloroform-*d*) δ 201.8, 201.7, 163.5, 163.5, 140.1, 139.8, 137.0, 136.8, 134.7, 134.6, 134.4, 134.1, 133.9, 132.2, 132.3, 130.2, 130.1, 129.7, 129.6, 129.0, 119.0, 119.0, 118.9, 118.8, 116.7, 116.4, 57.4, 56.9, 39.1, 38.6, 19.2, 18.0. **HRMS (ESI) *m/z*:** calculated for $C_{18}H_{17}ClO_2S$ [*M* + H]$^+$: 333.0711, found: 333.0708.

VI. *Characterization Data of Products 5*

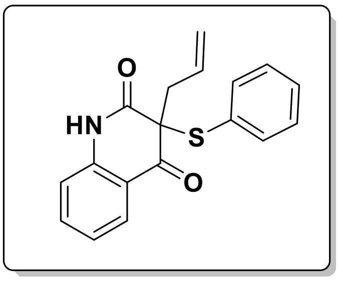

3-allyl-3-(phenylthio)quinoline-2,4(1H,3H)-dione (**5a,** column solvent: PE/EA = 3:1, yellow solid, 46 mg, 74%): **^{1}H NMR** (500 MHz, Chloroform-*d*) δ 10.13 (s, 1H), 7.90 (dd, J = 7.9, 1.5 Hz, 1H), 7.49 – 7.45 (m, 1H), 7.38 – 7.36 (m, 2H), 7.29 – 7.26 (m, 1H), 7.17 – 7.11 (m, 3H), 6.82 (dd, J = 8.1, 0.9 Hz, 1H), 5.69 – 5.61 (m, 1H), 5.18 – 5.02 (m, 1H), 5.03 (dd, J = 10.3, 1.8 Hz, 1H), 3.17 – 3.07 (m, 2H). **^{13}C NMR** (126 MHz, Chloroform-*d*) δ 189.7, 170.3, 139.9, 137.6, 135.9, 132.3, 130.8, 128.8, 128.3, 127.8, 123.8, 120.2, 120.0, 116.4, 63.2, 36.1. **HRMS (ESI) *m/z*:** calculated for $C_{18}H_{15}NO_2S$ [*M* + H]$^+$: 310.0896, found: 310.0901.

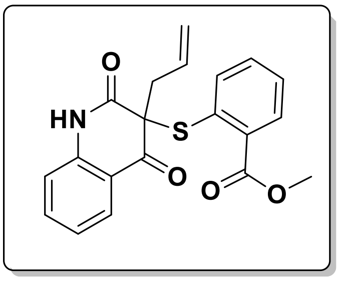

methyl 2-((3-allyl-2,4-dioxo-1,2,3,4-tetrahydroquinolin-3-yl)thio)benzoate (**5b,** column solvent: PE/EA = 3:1, yellow solid, 66 mg, 91%): **^{1}H NMR** (500 MHz, Chloroform-*d*) δ 9.96 (s, 1H), 7.85 (dd, J = 7.9, 1.6 Hz, 1H), 7.58 (dd, J = 7.5, 1.7 Hz, 1H), 7.48 – 7.43 (m, 2H), 7.30 – 7.23 (m, 2H), 7.11 – 7.08 (m, 1H), 6.84 (d, J = 8.1 Hz, 1H), 5.66 – 5.58 (m, 1H), 5.16 – 5.11 (m, 1H), 5.03 – 5.00 (m, 1H), 3.79 (s, 3H), 3.11 (d, J = 7.0 Hz, 2H). **^{13}C NMR** (126 MHz, Chloroform-*d*) δ 190.0, 170.1, 167.4, 140.0, 138.6, 137.6, 136.0, 131.9, 131.0, 130.1, 130.0, 128.6, 128.0, 123.7, 120.4, 119.7, 116.4, 63.4, 52.5, 37.4. **HRMS (ESI) *m/z*:** calculated for $C_{20}H_{17}NO_4S$ [*M* + H]$^+$: 368.0951, found: 368.0950.

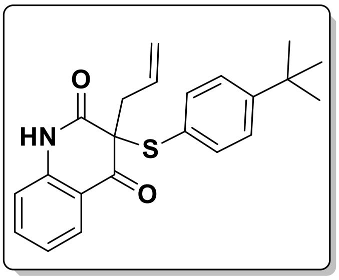

3-allyl-3-((4-(tert-butyl)phenyl)thio)quinoline-2,4(1H,3H)-dione (**5c,** column solvent: PE/EA = 3:1, yellow solid, 68 mg, 92%): **^{1}H NMR** (500 MHz, Chloroform-*d*) δ 10.14 (s, 1H), 7.86 (dd, *J* = 7.8, 1.5 Hz, 1H), 7.43 – 7.39 (m, 1H), 7.31 – 7.28 (m, 2H), 7.16 – 7.14 (m, 2H), 7.07 (td, *J* = 7.5, 1.0 Hz, 1H), 6.78 (dd, *J* = 8.0, 1.0 Hz, 1H), 5.71 – 5.63 (m, 1H), 5.18 – 5.14 (m, 1H), 5.04 – 5.01 (m, 1H), 3.15 – 3.06 (m, 2H), 1.20 (s, 9H). **^{13}C NMR** (126 MHz, Chloroform-*d*) δ 190.1, 170.7, 154.2, 140.0, 137.3, 135.7, 132.4, 127.7, 125.8, 124.6, 123.6, 120.3, 120.0, 116.3, 62.9, 35.8, 34.8, 31.2. **HRMS (ESI) *m/z*:** calculated for $C_{22}H_{23}NO_2S$ [*M* + H]$^+$: 366.1522, found: 366.1523.

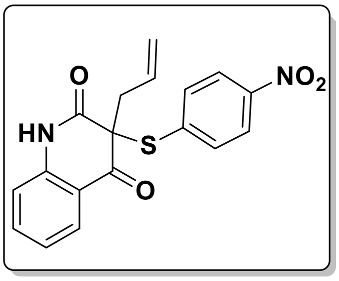

1-methoxy-6,6-dimethyl-3-phenyl-7,8-dihydroquinoline-2,5(1H,6H)-dione (**5d,** column solvent: PE/EA = 3:1, yellow solid, 23 mg, 32%): **^{1}H NMR** (500 MHz, Chloroform-*d*) δ 9.56 (s, 1H), 8.04 – 8.02 (m, 2H), 7.91 (dd, *J* = 7.9, 1.4 Hz, 1H), 7.56 – 7.50 (m, 3H), 7.17 (t, *J* = 7.5 Hz, 1H), 6.82 (d, *J* = 8.0 Hz, 1H), 5.66 – 5.58 (m, 1H), 5.16 (dd, *J* = 17.1, 1.7 Hz, 1H), 5.06 (dd, *J* = 10.3, 1.6 Hz, 1H), 3.16 – 3.06 (m, 2H). **^{13}C NMR** (126 MHz, Chloroform-*d*) δ 189.0, 169.2, 149.0, 139.6, 137.9, 137.0, 136.5, 131.4, 128.2, 124.4, 123.6, 121.0, 119.7, 116.3, 63.7, 36.9. **HRMS (ESI) *m/z*:** calculated for $C_{18}H_{14}N_2O_4S$ [*M* + H]$^+$: 355.0747, found: 355.0744.

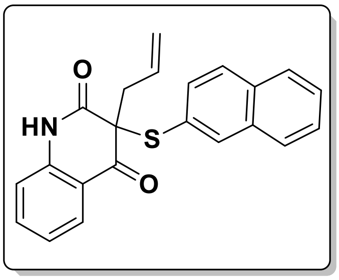

3-allyl-3-(naphthalen-2-ylthio)quinoline-2,4(1H,3H)-dione (**5e,** column solvent: PE/EA = 3:1, yellow solid, 50 mg, 69%): **^{1}H NMR** (500 MHz, Chloroform-*d*) δ 9.90 (s, 1H), 7.93 – 7.91 (m, 2H), 7.69 -7.68 (m, 1H), 7.63 -7.61 (m, 1H), 7.59 -7.58 (m, 1H), 7.46 -7.43 (m, 1H), 7.41 – 7.37 (m, 2H), 7.33 -7.30 (m, 1H), 7.09 – 7.05 (m, 1H), 6.60 (d, *J* = 7.9 Hz, 1H), 5.66 -5.58 (m, 1H), 5.17 -5.13 (m, 1H), 5.01 (dd, *J* = 10.3, 1.9 Hz, 1H), 3.19 – 3.12 (m, 2H). **^{13}C NMR** (126 MHz, Chloroform-*d*) δ 189.7, 170.0, 139.9, 138.4, 135.8, 133.9, 133.2, 133.2, 132.3, 128.3, 128.2, 127.8, 127.8, 127.6, 126.6, 125.6, 123.7, 120.2, 120.0, 116.3, 63.5, 36.1. **HRMS (ESI) *m/z*:** calculated for $C_{22}H_{17}NO_2S$ [*M* + H]$^+$: 360.1053, found: 360.1051.

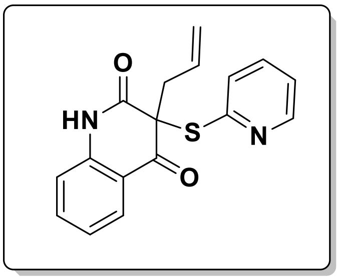

3-allyl-3-(pyridin-2-ylthio)quinoline-2,4(1H,3H)-dione (**5f,** column solvent: PE/EA = 3:1, yellow solid, 25 mg, 40%): **^{1}H NMR** (500 MHz, Chloroform-*d*) δ 9.38 (s, 1H), 7.95 (dd, *J* = 7.8, 1.4 Hz, 1H), 7.65 – 7.64 (m, 1H), 7.51 – 7.48 (m, 1H), 7.41 – 7.38 (m, 1H), 7.18 (d, *J* = 8.1 Hz, 1H), 7.13 – 7.10 (m, 1H), 6.91 (d, *J* = 8.1 Hz, 1H), 6.76 – 6.73 (m, 1H), 5.75 – 5.66 (m, 1H), 5.17 – 5.13 (m, 1H), 5.05 (dd, *J* = 10.1, 1.5 Hz, 1H), 2.90 – 2.82 (m, 2H). **^{13}C NMR** (126 MHz, Chloroform-*d*) δ 192.7, 172.5, 156.2, 148.3, 140.0, 136.4, 135.5, 129.7, 127.8, 123.3, 120.9, 120.8, 120.6, 119.8, 116.1, 66.2, 41.0. **HRMS (ESI) *m/z*:** calculated for $C_{17}H_{14}N_2O_2S$ [*M* + H]$^+$: 311.0849, found: 311.0847.

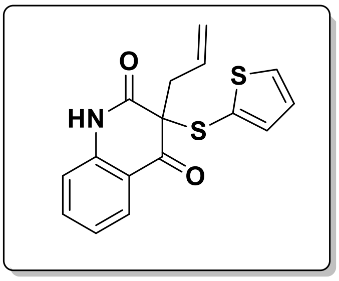

3-allyl-3-(thiophen-2-ylthio)quinoline-2,4(1H,3H)-dione (**5g,** column solvent: PE/EA = 3:1, yellow solid, 31 mg, 49%): **^{1}H NMR** (500 MHz, Chloroform-*d*) δ 9.75 (s, 1H), 7.92 (dd, *J* = 7.8, 1.5 Hz, 1H), 7.52 – 7.40 (m, 1H), 7.41 (dd, *J* = 5.4, 1.2 Hz, 1H), 7.14 (td, *J* = 7.5, 1.0 Hz, 1H), 7.10 (dd, *J* = 3.6, 1.3 Hz, 1H), 6.90 (dd, *J* = 5.4, 3.6 Hz, 1H), 6.87 (dd, *J* = 8.1, 0.9 Hz, 1H), 5.68 – 5.60 (m, 1H)), 5.18 – 5.14 (m, 1H), 5.04 – 5.02 (m, 1H), 3.17 – 3.09 (m, 2H). **^{13}C NMR** (126 MHz, Chloroform-*d*) δ 189.3, 169.5, 140.0, 139.6, 136.0, 134.2, 132.2, 128.1, 128.0, 126.4, 123.9, 120.3, 119.8, 116.4, 64.8, 35.9. **HRMS (ESI) *m/z*:** calculated for $C_{16}H_{13}NO_2S_2$ [*M* + H]$^+$: 316.0460, found: 316.0459.

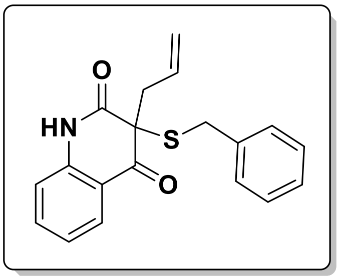

3-allyl-3-(benzylthio)quinoline-2,4(1H,3H)-dione (**5h,** column solvent: PE/EA = 3:1, yellow oil, 37 mg, 57%): **^{1}H NMR** (500 MHz, Chloroform-*d*) δ 10.11 (s, 1H), 7.98 (dd, *J* = 7.9, 1.4 Hz, 1H), 7.55 (td, *J* = 7.7, 1.5 Hz, 1H), 7.22 – 7.15 (m, 6H), 7.03 (d, *J* = 8.0 Hz, 1H), 5.72 – 5.64 (m, 1H), 5.16 (dd, *J* = 17.2, 1.7 Hz, 1H), 5.04 (dd, *J* = 10.3, 1.8 Hz, 1H), 3.92 – 3.89 (m, 1H), 3.84 – 3.82 (m, 1H), 3.20 – 3.12 (m, 2H). **^{13}C NMR** (126 MHz, Chloroform-*d*) δ 188.2, 170.0, 139.9, 136.1, 135.4, 132.2, 129.5, 128.6, 128.2, 127.6, 124.0, 120.1, 119.4, 116.6, 61.0, 36.5, 34.8. **HRMS (ESI) *m/z*:** calculated for $C_{19}H_{17}NO_2S$ [*M* + H]$^+$: 324.1053, found: 324.1052.

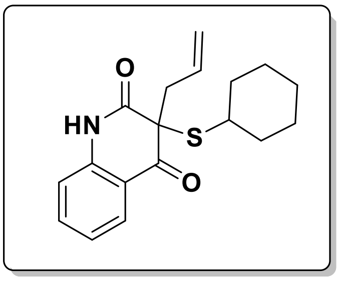

3-allyl-3-(cyclohexylthio)quinoline-2,4(1H,3H)-dione (**5i,** column solvent: PE/EA = 3:1, yellow oil, 62 mg, 97%): **^{1}H NMR** (500 MHz, Chloroform-*d*) δ 10.12 (s, 1H), 7.98 (dd, *J* = 7.8, 1.4 Hz, 1H), 7.55 (td, *J* = 7.7, 1.6 Hz, 1H), 7.16 (td, *J* = 7.7, 1.0 Hz, 1H), 7.06 (d, *J* = 7.9 Hz, 1H), 5.69 – 5.61 (m, 1H), 5.16 – 5.12 (m, 1H), 5.01 (dd, *J* = 10.3, 1.9 Hz, 1H), 3.19 – 3.11 (m, 2H), 3.01 – 2.95 (m, 1H), 1.91 – 1.86 (m, 1H), 1.80 – 1.76 (m, 1H), 1.66 – 1.58 (m, 2H), 1.50 – 1.44 (m, 1H), 1.39 – 1.22 (m, 4H), 1.17 – 1.10 (m, 1H). **^{13}C NMR** (126 MHz, Chloroform-*d*) δ 189.2, 170.5, 139.8, 135.9, 132.5, 128.1, 124.0, 120.0, 119.5, 116.6, 60.8, 43.5, 37.0, 35.5, 35.5, 35.0, 26.3, 25.3. **HRMS (ESI) *m/z*:** calculated for $C_{18}H_{21}NO_2S$ [*M* + H]$^+$: 316.1366, found: 316.1365.

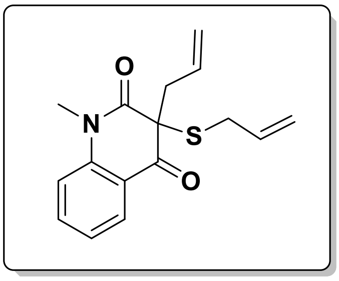

3-allyl-3-(allylthio)-1-methylquinoline-2,4(1H,3H)-dione (**5j,** column solvent: PE/EA = 3:1, yellow oil, 33 mg, 57%): **^{1}H NMR** (500 MHz, Chloroform-*d*) δ 8.06 (dd, *J* = 7.8, 1.7 Hz, 1H), 7.64 – 7.61 (m, 1H), 7.20 (t, *J* = 7.5 Hz, 1H), 7.15 (d, *J* = 8.4 Hz, 1H), 5.72 – 5.58 (m, 2H), 5.12 (d, *J* = 17.0 Hz, 2H), 5.04 – 4.99 (m, 2H), 3.48 (s, 3H), 3.31 – 3.19 (m, 2H), 3.12 (d, *J* = 6.9 Hz, 2H). **^{13}C NMR** (126 MHz, Chloroform-*d*) δ 188.6, 167.7, 142.3, 136.0, 132.6, 132.4, 128.6, 123.4, 120.6, 119.9, 119.1, 114.9, 60.6, 36.8, 33.2, 30.2. **HRMS (ESI)** ***m/z*:** calculated for $C_{16}H_{13}NO_2S$ [*M* + H]$^+$: 288.1053, found: 288.1052.

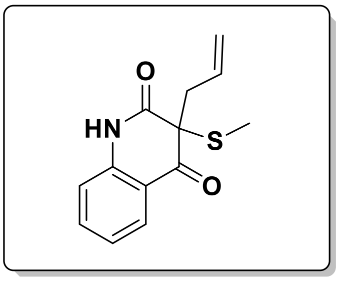

3-allyl-3-(methylthio)quinoline-2,4(1H,3H)-dione (**5k,** column solvent: PE/EA = 3:1, yellow solid, 35 mg, 71%): **^{1}H NMR** (500 MHz, Chloroform-*d*) δ 10.13 (s, 1H), 8.00 (dd, *J* = 7.9, 1.5 Hz, 1H), 7.58 – 7.54 (m, 1H), 7.19 – 7.14 (m, 1H), 7.06 (d, *J* = 8.1 Hz, 1H), 5.72 – 5.64 (m, 1H), 5.17 – 5.13 (m, 1H), 5.03 (dd, *J* = 10.3, 1.8 Hz, 1H), 3.12 – 3.04 (m, 2H), 2.15 (s, 3H). **^{13}C NMR** (126 MHz, Chloroform-*d*) δ 188.3, 170.2, 139.9, 136.0, 132.4, 128.2, 124.0, 120.0, 119.4, 116.5, 59.3, 34.9, 13.0. **HRMS (ESI)** ***m/z*:** calculated for $C_{13}H_{13}NO_2S$ [*M* + H]$^+$: 248.0740, found: 248.0739.

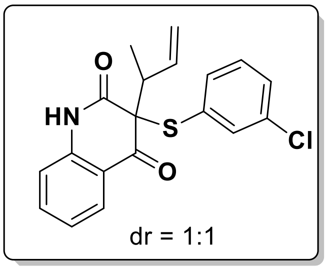

2-allyl-3-(methylthio)quinoline-2,4(1H,3H)-dione (**5l,** column solvent: PE/EA = 3:1, yellow solid, 49 mg, 68%): **^{1}H NMR** (500 MHz, Chloroform-*d*) δ 10.09 (s, 1H), 9.99 (s, 1H), 7.87 (dd, *J* = 7.9, 1.5 Hz, 1H), 7.82 (dd, *J* = 7.8, 1.5 Hz, 1H), 7.47 – 7.43 (m, 2H), 7.34 (t, *J* = 1.9 Hz, 1H), 7.29 (t, *J* = 1.9 Hz, 1H), 7.23 (dt, *J* = 7.8, 1.4 Hz, 1H), 7.20 (dt, *J* = 7.7, 1.4 Hz, 1H), 7.14 – 7.06 (m, 4H), 7.00 – 6.95 (m, 2H), 6.79 (dd, *J* = 8.1, 1.0 Hz, 1H), 6.73 (dd, *J* = 8.0, 1.0 Hz, 1H), 6.37 – 6.30 (m, 1H), 6.26 – 6.19 (m, 1H), 5.27 – 5.15 (m, 4H), 3.51 – 3.44 (m, 2H), 1.33 (d, *J* = 7.0 Hz, 3H), 1.23 (d, *J* = 7.0 Hz, 3H). **^{13}C NMR** (126 MHz, Chloroform-*d*) δ 191.7, 191.6, 171.3, 171.0, 139.8, 139.7, 138.9, 138.2, 136.5, 136.3, 136.2, 134.8, 134.2, 134.1, 131.1, 130.9, 130.3, 130.3, 129.6, 129.6, 127.6, 127.6, 124.0, 123.9, 120.6, 120.3, 118.0, 117.6, 116.1, 116.1, 66.8, 66.8, 43.4, 43.4, 17.5, 17.4. **HRMS (ESI)** ***m/z*:** calculated for $C_{19}H_{16}ClNO_2S$ [*M* + H]$^+$: 358.0663, found: 358.0660.

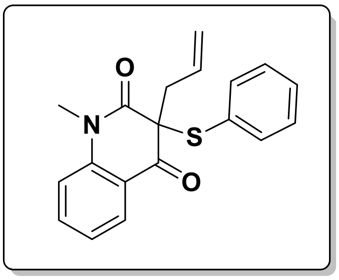

3-allyl-1-methyl-3-(phenylthio)quinoline-2,4(1H,3H)-dione (**5m,** column solvent: PE/EA = 3:1, yellow solid, 52 mg, 80%): **^{1}H NMR** (500 MHz, Chloroform-*d*) δ 7.98 (dd, *J* = 7.8, 1.7 Hz, 1H),7.49 – 7.46 (m, 1H), 7.30 – 7.27 (m, 1H), 7.24 – 7.22 (m, 2H), 7.15 – 7.12 (m, 3H), 6.78 (d, *J* = 8.4 Hz, 1H), 5.69 – 5.61 (m, 1H), 5.18 – 5.14 (m, 1H), 5.03 – 5.00 (m, 1H), 3.21 (s, 3H), 3.19 – 3.04 (m, 2H). **^{13}C NMR** (126 MHz, Chloroform-*d*) δ 189.9, 167.6, 142.2, 137.4, 135.7, 132.5, 130.6, 128.7, 128.4, 128.0, 123.2, 121.6, 120.0, 114.5, 62.9, 36.4, 29.8. **HRMS (ESI) *m/z*:** calculated for $C_{19}H_{17}NO_2S$ [*M* + H]$^+$: 324.1053, found: 324.1060.

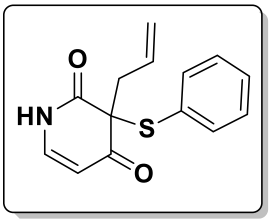

3-allyl-3-(phenylthio)pyridine-2,4(1H,3H)-dione (**5n,** column solvent: PE/EA = 3:1, yellow oil, 29 mg, 56%): **^{1}H NMR** (500 MHz, Chloroform-*d*) δ 10.86 – 10.67 (m, 1H), 7.51 – 7.46 (m, 1H), 7.43 – 7.37 (m, 4H), 7.25 (dd, *J* = 8.0, 5.4 Hz, 1H), 5.49 – 5.42 (m, 1H), 5.38 (d, *J* = 8.0 Hz, 1H), 4.99 – 4.96 (m, 1H), 4.92 – 4.88 (m, 1H), 2.83 – 2.70 (m, 2H). **^{13}C NMR** (126 MHz, Chloroform-*d*) δ 190.5, 170.8, 143.5, 137.2, 132.4, 130.6, 129.0, 127.9, 119.3, 105.0, 63.7, 36.1. **HRMS (ESI) *m/z*:** calculated for $C_{14}H_{13}NO_2S$ [*M* + H]$^+$: 260.0740, found: 260.0745.

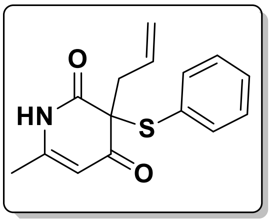

3-allyl-6-methyl-3-(phenylthio)pyridine-2,4(1H,3H)-dione (**5o,** column solvent: PE/EA = 3:1, yellow soild, 30 mg, 55%): **^{1}H NMR** (500 MHz, Chloroform-*d*) δ 8.95 (s, 1H), 7.52 – 7.49 (m, 2H), 7.43 – 7.40 (m, 1H), 7.33 – 7.29 (m, 2H), 5.60 – 5.52 (m, 1H), 5.34 (s, 1H), 5.09 – 5.01 (m, 2H), 3.04 – 3.00 (m, 1H), 2.88 – 2.84 (m, 1H), 1.85 (d, J = 1.0 Hz, 3H). **^{13}C NMR** (126 MHz, Chloroform-*d*) δ 191.0, 173.1, 152.7, 137.7, 132.1, 130.7, 128.9, 128.7, 120.0, 106.5, 62.4, 36.4, 20.1. **HRMS (ESI) *m/z*:** calculated for $C_{15}H_{15}NO_2S$ [*M* + H]$^+$: 274.0896, found: 274.0900.

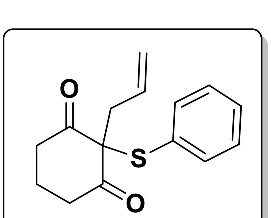

2-allyl-2-(phenylthio)cyclohexane-1,3-dione (**5p,** column solvent: PE/EA = 3:1, yellow solid, 32 mg, 61%): **^{1}H NMR** (500 MHz, Chloroform-*d*) δ 7.45 – 7.41 (m, 3H), 7.37 – 7.34 (m, 2H), 5.53 – 5.45 (m, 1H), 5.05 – 4.97 (m, 2H), 2.96 – 2.90 (m, 2H), 2.71 (d, *J* = 7.1 Hz, 2H), 2.51 – 2.45 (m, 2H), 2.22 – 2.14 (m, 1H), 1.83 – 1.74 (m, 1H). **^{13}C NMR** (126 MHz, Chloroform-*d*) δ 202.7, 137.5, 132.7, 130.8, 129.4, 128.4, 119.9, 69.8, 38.5, 35.8, 17.1. **HRMS (ESI) *m/z*:**

calculated for $C_{15}H_{16}O_2S$ [M + H]$^+$: 261.0944, found: 261.0947.

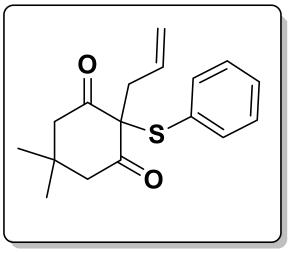

2-allyl-5,5-dimethyl-2-(phenylthio)cyclohexane-1,3-dione (**5q,** column solvent: PE/EA = 3:1, yellow oil, 29 mg, 49%): **^{1}H NMR** (500 MHz, Chloroform-*d*) δ 7.46 – 7.42 (m, 1H), 7.41 – 7.39 (m, 2H), 7.37 – 7.34 (m, 2H), 5.57 – 5.49 (m, 1H), 5.11 – 5.06 (m, 1H), 4.99 (dd, J = 10.2, 2.1 Hz, 1H), 3.13 (d, J = 14.7 Hz, 2H), 2.65 (d, J = 7.1 Hz, 2H), 2.39 – 2.35 (m, 2H), 1.13 (s, 3H), 0.80 (s, 3H). **^{13}C NMR** (126 MHz, Chloroform-*d*) δ 202.6, 137.4, 132.9, 130.9, 129.4, 128.4, 120.2, 68.7, 51.3, 34.8, 31.1, 30.8, 26.4. **HRMS (ESI) *m/z*:** calculated for $C_{17}H_{20}O_2S$ [M + H]$^+$: 289.1257, found: 289.1260.

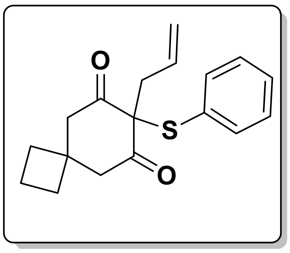

7-allyl-7-(phenylthio)spiro[3.5]nonane-6,8-dione (**5r,** column solvent: PE/EA = 3:1, yellow oil, 32 mg, 53%): **^{1}H NMR** (500 MHz, Chloroform-*d*) δ 7.45 – 7.42 (m, 1H), 7.40 – 7.38 (m, 2H), 7.36 – 7.33 (m, 2H), 5.46 – 5.38 (m, 1H), 5.04 – 5.00 (m, 1H), 4.95 – 4.93 (m, 1H), 3.17 – 3.13 (m, 2H), 2.77 – 2.74 (m, 2H), 2.65 – 2.64 (m, 2H), 2.00 – 1.96 (m, 2H), 1.95 – 1.89 (m, 2H), 1.69 – 1.66 (m, 2H). **^{13}C NMR** (126 MHz, Chloroform-*d*) δ 202.4, 137.4, 132.9, 130.8, 129.4, 128.4, 119.8, 69.0, 50.3, 36.2, 35.2, 33.2, 30.5, 15.2. **HRMS (ESI) *m/z*:** calculated for $C_{18}H_{20}O_2S$ [M + H]$^+$: 301.1257, found: 301.1255.

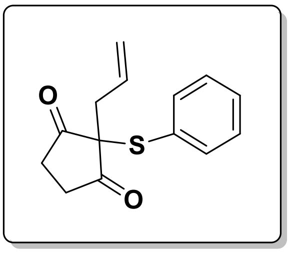

2-allyl-2-(phenylthio)cyclopentane-1,3-dione (**5s,** column solvent: PE/EA = 3:1, yellow oil, 17 mg, 66%): **^{1}H NMR** (500 MHz, Chloroform-*d*) δ 7.46 – 7.43 (m, 3H), 7.37 – 7.34 (m, 2H), 5.57 – 5.48 (m, 1H), 5.08 – 5.02 (m, 2H), 2.79 – 2.74 (m, 2H), 2.68 (d, J = 7.2 Hz, 2H), 2.54 – 2.49 (m, 2H). **^{13}C NMR** (126 MHz, Chloroform-*d*) δ 208.2, 137.3, 131.3, 130.8, 129.4, 128.1, 120.6, 62.5, 35.5, 35.4. **HRMS (ESI) *m/z*:** calculated for $C_{14}H_{14}O_2S$ [M + H]$^+$: 247.0787, found: 247.0793.

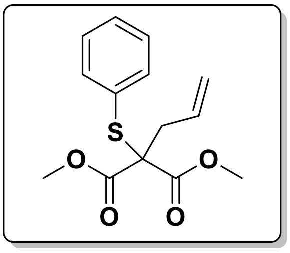

dimethyl 2-allyl-2-(phenylthio)malonate (**5t,** column solvent: PE/EA = 20:1, yellow oil, 32 mg, 57%): **^{1}H NMR** (500 MHz, Chloroform-*d*) δ 7.50 – 7.49 (m, 2H), 7.41 – 7.38 (m, 1H), 7.34 – 7.31 (m, 2H), 5.96 – 5.88 (m, 1H), 5.18 – 5.13 (m, 2H), 3.71 (s, 6H), 2.74 (d, J = 7.0 Hz, 2H). **^{13}C NMR** (126

MHz, Chloroform-*d*) δ 168.6, 137.2, 131.9, 130.2, 129.5, 129.1, 119.7, 65.0, 53.1, 38.5. **HRMS (ESI) *m/z*:** calculated for $C_{14}H_{16}O_4S$ [*M* + H]$^+$: 281.0842, found: 281.0846.

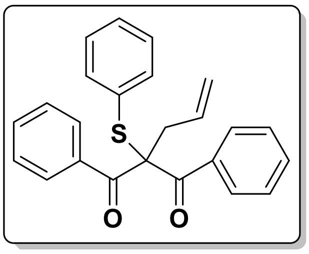

2-allyl-1,3-diphenyl-2-(phenylthio)propane-1,3-dione (**5u,** column solvent: PE/EA = 20:1, yellow solid, 49 mg, 65%): **^{1}H NMR** (500 MHz, Chloroform-*d*) δ 7.94 (d, *J* = 7.8 Hz, 4H), 7.45 – 7.42 (m, 2H), 7.39 – 7.36 (m, 1H), 7.34 – 7.31 (m, 4H), 7.28 – 7.25 (m, 4H), 6.08 – 6.00 (m, 1H), 5.06 (d, *J* = 10.2 Hz, 1H), 4.78 (dd, *J* = 17.1, 1.9 Hz, 1H), 2.93 (d, *J* = 6.9 Hz, 2H). **^{13}C NMR** (126 MHz, Chloroform-*d*) δ 193.8, 137.7, 135.8, 133.4, 131.6, 130.2, 129.7, 129.3, 129.1, 128.7, 119.4, 75.5, 38.8. **HRMS (ESI) *m/z*:** calculated for $C_{24}H_{20}O_2S$ [*M* + H]$^+$: 373.1257, found: 373.1263.

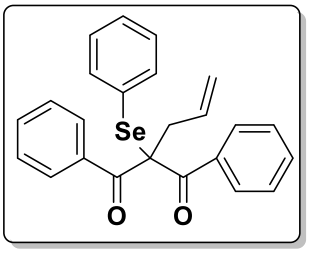

1,3-diphenyl-2-(phenylthio)-2-(propa-1,2-dien-1-yl)propane-1,3-dione (**5v,** column solvent: PE/EA = 20:1, yellow oil, 49 mg, 65%): **^{1}H NMR** (500 MHz, Chloroform-*d*) δ 7.59 – 7.57 (m, 2H), 7.44 – 7.43 (m, 2H), 7.27 – 7.25 (m, 3H), 7.22 – 7.19 (m, 1H), 7.16 – 7.12 (m, 3H), 7.08 – 7.00 (m, 4H), 5.04 – 4.98 (m, 1H), 3.45 (dd, *J* = 15.2, 9.9 Hz, 1H), 3.38 (dd, *J* = 12.6, 5.5 Hz, 1H), 3.24 – 3.20 (m, 1H), 3.12 (dd, *J* = 15.2, 7.4 Hz, 1H). **^{13}C NMR** (126 MHz, Chloroform-*d*) δ 193.4, 165.4, 139.0, 133.3, 131.2, 130.1, 130.0, 129.4, 129.3, 129.3, 129.0, 127.7, 127.6, 127.5, 111.7, 81.1, 38.6, 32.7. **HRMS (ESI) *m/z*:** calculated for $C_{24}H_{20}O_2Se$ [*M* + H]$^+$: 421.0701, found: 421.0700.

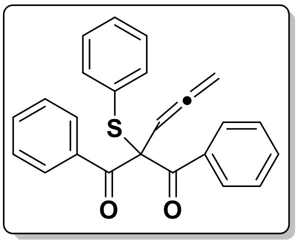

1,3-diphenyl-2-(phenylthio)-2-(propa-1,2-dien-1-yl)propane-1,3-dione (**5w,** column solvent: PE/EA = 20:1, yellow solid, 40 mg, 54%): **^{1}H NMR** (500 MHz, Chloroform-*d*) δ 7.91 – 7.89 (m, 4H), 7.39 – 7.35 (m, 5H), 7.29 – 7.25 (m, 6H), 6.17 (t, *J* = 6.8 Hz, 1H), 4.72 (d, *J* = 6.8 Hz, 1H). **^{13}C NMR** (126 MHz, Chloroform-*d*) δ 208.9, 191.7, 137.8, 135.4, 133.2, 130.0, 129.9, 129.5, 128.9, 128.5, 91.4, 79.2, 73.3. **HRMS (ESI) *m/z*:** calculated for $C_{24}H_{18}O_2S$ [*M* + H]$^+$: 371.1100, found: 371.1101.

VII. *Characterization Data of Products 7*

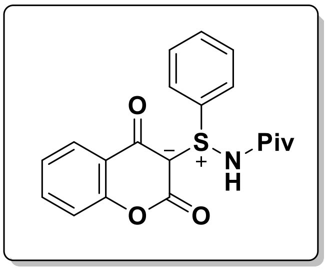

(*Z*)-*N*-((2,4-dioxochroman-3-ylidene)(4-methoxyphenyl)-λ^4-sulfaneyl)pivalamide (**7a**, column solvent: PE/EA = 2/1, yellow solid, 59 mg, 80%): **^{1}H NMR** (500 MHz, $CDCl_3$) *δ* 12.90 (br, 1H), 8.02 (d, *J* = 7.6 Hz, 1H), 7.87 (d, 2H), 7.63 – 7.51 (m, 2H), 7.54 – 7.46 (m, 2H), 7.31 – 7.23 (m, 2H), 1.27 (s, 9H). **^{13}C NMR** (151 MHz, $CDCl_3$) *δ* 178.4, 177.7, 162.4, 154.6, 134.6, 133.9, 133.6, 130.5, 127.7, 125.4, 124.1, 121.2, 117.3, 81.0, 40.3, 27.2. **HRMS (ESI-MS) *m/z:*** calculated for $C_{20}H_{20}NO_4S$ [*M* + H]$^+$: 370.1108; Found: 370.1112.

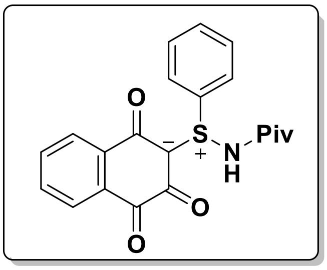

(*Z*)-*N*-(phenyl(1,3,4-trioxo-3,4-dihydronaphthalen-2(1*H*)-ylidene)-λ^4-sulfaneyl)pivalamide (**7b**, column solvent: PE/EA = 2/1, yellow solid, 73 mg, 96%): **^{1}H NMR** (500 MHz, $CDCl_3$) *δ* 12.97 (br, 1H), 8.14 – 8.12 (m, 1H), 8.10 – 8.07 (m, 1H), 7.93 – 7.88 (m, 2H), 7.74 – 7.69 (m, 1H), 7.68 – 7.63 (m, 1H), 7.61 – 7.56 (m, 1H), 7.55 – 7.49 (m, 2H), 1.27 (s, 9H). **^{13}C NMR** (126 MHz, $CDCl_3$) *δ* 181.8, 179.3, 178.8, 173.3, 134.8, 134.8, 133.8, 133.3, 133.0, 131.9, 130.5, 128.1, 127.9, 126.5, 40.3, 27.1. **HRMS (ESI-MS) *m/z:*** calculated for $C_{21}H_{20}NO_4S$ [*M* + H]$^+$: 382.1108; Found: 382.1110.

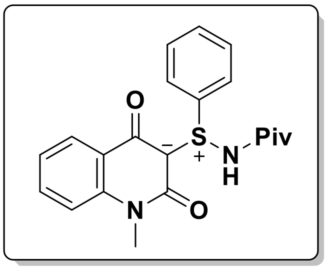

(*Z*)-*N*-((1-methyl-2,4-dioxo-1,4-dihydroquinolin-3(2*H*)-ylidene)(phenyl)-λ^4-sulfaneyl)pivalamide (**7c**, column solvent: PE/EA = 8/1, yellow solid, 53 mg, 80%): **^{1}H NMR** (500 MHz, $CDCl_3$) *δ* 8.27 – 8.22 (m, 1H), 7.96 – 7.92 (m, 2H), 7.63 – 7.58 (m, 1H), 7.56 – 7.51 (m, 1H), 7.48 (t, J = 7.4 Hz, 2H), 7.29 – 7.23 (m, 1H), 3.56 (s, 3H), 1.28 (s, 8H) **^{13}C NMR** (126 MHz, $CDCl_3$) *δ* 175.5, 141.5, 136.4, 133.2, 133.0, 130.2, 128.1, 125.9, 122.0, 121.9, 114.5, 40.2, 29.0, 27.4. **HRMS (ESI-MS) *m/z:*** calculated for $C_{21}H_{22}N_2O_3S$ [*M* + H]$^+$: 383.1424; Found: 383.1427.

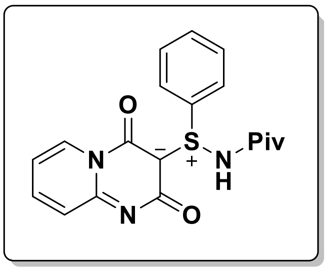

(*E*)-*N*-((2,4-dioxo-2H-pyrido[1,2-a]pyrimidin-3(4*H*)-ylidene)(phenyl)-λ^4-sulfaneyl)pivalamide (**7d**, column solvent: PE/EA = 2/1, yellow solid, 60 mg, 81%): **^{1}H NMR** (600 MHz, $CDCl_3$) *δ* 8.61 (d, *J* = 7.0 Hz, 1H), 7.90 (d, *J* = 7.6

Hz, 2H), 7.62 – 7.56 (m, 1H), 7.55 – 7.48 (m, 1H), 7.46 (t, *J* = 7.7 Hz, 2H), 7.28 (d, *J* = 9.0 Hz, 1H), 6.83 (t, 1H), 1.23 (s, 9H). **^{13}C NMR** (151 MHz, $CDCl_3$) δ 180.8, 170.4, 157.4, 152.9, 138.7, 135.3, 133.3, 130.2, 127.7, 127.4, 124.6, 113.0, 81.7, 40.1, 27.2. **HRMS (ESI-MS)** ***m/z:*** calculated for $C_{19}H_{20}N_3O_3S$ [*M* + H]$^+$: 370.1220; Found: 370.1224.

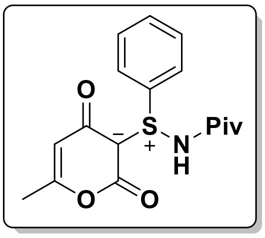

(*Z*)-*N*-((6-methyl-2,4-dioxo-2H-pyran-3(4*H*)-ylidene)(phenyl)-λ^4-sulfaneyl)pivalamide (**7e**, column solvent: PE/EA = 2/1, yellow solid, 60 mg, 90%): **^{1}H NMR** (500 MHz, $CDCl_3$) δ 13.17 (br, 1H), 7.86 – 7.77 (m, 2H), 7.61 – 7.53 (m, 1H), 7.53 – 7.45 (m, 2H), 5.73 (s, 1H), 2.13 (s, 3H), 1.23 (s, 9H). **^{13}C NMR** (151 MHz, $CDCl_3$) δ 180.8, 178.6, 164.1, 163.1, 134.8, 133.5, 130.4, 127.7, 108.0, 80.1, 40.2, 27.1, 20.0. **HRMS (ESI-MS)** ***m/z:*** calculated for $C_{17}H_{20}NO_4S$ [*M* + H]$^+$: 334.1108; Found: 334.1111.

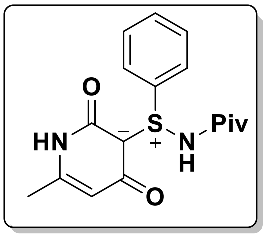

(*E*)-*N*-((6-methyl-2,4-dioxo-1,4-dihydropyridin-3(2*H*)-ylidene) (phenyl)-λ^4-sulfaneyl)pivalamide (**7f**, column solvent: PE/EA = 2/1, white solid, 48 mg, 72%): **^{1}H NMR** (600 MHz, $CDCl_3$) δ 11.94 (br, 1H), 7.82 (d, *J* = 7.2 Hz, 2H), 7.51 – 7.45 (m, 1H), 7.46 – 7.40 (m, 2H), 5.69 (s, 1H), 2.11 (s, 3H), 1.21 (s, 9H). **^{13}C NMR** (151 MHz, $CDCl_3$) δ 183.3, 177.2, 163.8, 149.7, 137.0, 132.6, 129.8, 127.6, 104.9, 90.0, 40.0, 27.7, 19.2. **HRMS (ESI-MS)** ***m/z:*** calculated for $C_{17}H_{21}N_2O_3S$ [*M* + H]$^+$: 333.1267; Found: 333.1269.

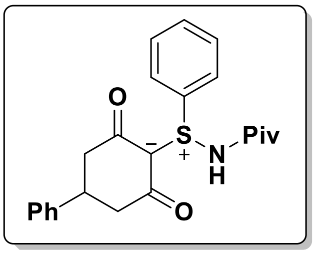

N-((2,6-dioxo-4-phenylcyclohexylidene)(phenyl)-λ^4-sulfaneyl)pivalamide (**7g** column solvent: PE/EA = 8/1, yellow solid, 67 mg, 85%): **^{1}H NMR** (500 MHz, $CDCl_3$) δ 13.14 (br, 1H), 7.82 – 7.79 (m, 2H), 7.60 – 7.57 (m, 1H), 7.55 – 7.51 (m, 2H), 7.38 – 7.34 (m, 2H), 7.31 – 7.28 (m, 1H), 7.27 (d, J = 7.9 Hz, 2H), 3.49 (d, J = 8.0 Hz, 1H), 2.84 – 2.76 (m, 4H), 1.29 (s, 9H). **^{13}C NMR** (151 MHz, $CDCl_3$) δ 192.5, 178.7, 142.7, 135.8, 133.0, 130.2, 128.9, 127.7, 127.0, 126.8, 92.4, 44.7, 44.1, 40.2, 38.5, 27.2. **HRMS (ESI-MS)** ***m/z:*** calculated for $C_{23}H_{25}NO_3S$ [*M* + H]$^+$: 396.1628; Found: 396.1631.

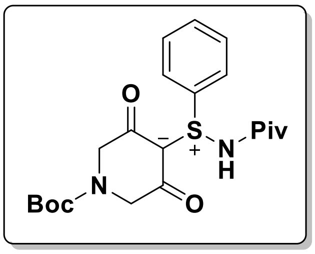

tert-butyl 3,5-dioxo-4-(phenyl(pivalamido)-λ^4-sulfaneylidene)piperidine-1-carboxylate (**7h** column solvent: PE/EA = 2/1, white

solid, 23 mg, 27%): **^{1}H NMR** (600 MHz, $CDCl_3$) δ 12.45 (s, 1H), 7.80 – 7.75 (m, 2H), 7.60 – 7.54 (m, 1H), 7.55 – 7.47 (m, 2H), 4.18 (s, 4H), 1.46 (s, 9H), 1.24 (s, 9H). **^{13}C NMR** (151 MHz, $CDCl_3$) δ 178.5, 154.1, 135.0, 133.4, 130.4, 127.8, 91.4, 81.3, 40.3, 28.4, 27.2. **HRMS (ESI-MS) *m/z:*** calculated for $C_{21}H_{29}N_2O_5S$ [M + H]$^+$: 421.1792; Found: 421.1796.

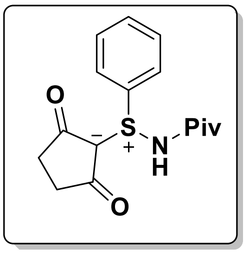

N-((2,5-dioxocyclopentylidene)(phenyl)-λ^4-sulfaneyl)pivalamide (**7i** column solvent: PE/EA = 8/1, yellow solid, 52 mg, 85%): **^{1}H NMR** (500 MHz, $CDCl_3$) δ 7.83 – 7.78 (m, 2H), 7.61 – 7.55 (m, 1H), 7.54 – 7.46 (m, 2H), 2.57 (d, J = 2.5 Hz, 4H), 1.22 (t, J = 2.3 Hz, 9H). **^{13}C NMR** (151 MHz, $CDCl_3$) δ 200.0, 178.3, 135.3, 133.8, 130.5, 127.9, 86.6, 40.2, 33.6, 27.1. **HRMS (ESI-MS) *m/z:*** calculated for $C_{21}H_{29}N_2O_5S$ [M + H]$^+$: 306.1158; Found: 306.1160.

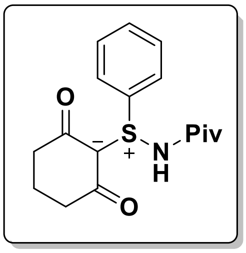

N-((2,6-dioxocyclohexylidene)(phenyl)-λ^4-sulfaneyl)pivalamide (**7j** column solvent: PE/EA = 8/1, yellow solid, 63 mg, 98%): **^{1}H NMR** (500 MHz, $CDCl_3$) δ 13.16 (s, 1H), 7.75 (dt, J = 7.9, 1.1 Hz, 2H), 7.54 – 7.49 (m, 1H), 7.48 – 7.44 (m, 2H), 2.49 – 2.42 (m, 3H), 2.03 – 1.97 (m, 2H), 1.21 (d, J = 1.0 Hz, 9H). **^{13}C NMR** (151 MHz, $CDCl_3$) δ 193.8, 178.7, 135.9, 132.9, 130.1, 127.6, 92.6, 40.1, 37.2, 27.1, 20.8. **HRMS (ESI-MS) *m/z:*** calculated for $C_{17}H_{21}NO_3S$ [M + H]$^+$: 320.1315; Found: 320.1317.

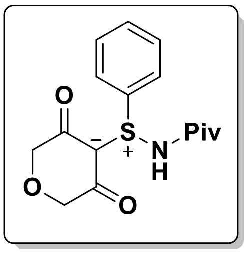

(*E*)-*N*-((5-hydroxy-3-oxo-3,6-dihydro-2*H*-pyran-4-yl)(phenyl)-λ^4-sulfaneylidene)pivalamide (**7k** column solvent: PE/EA = 8/1, yellow solid, 59 mg, 92%): **^{1}H NMR** (500 MHz, $CDCl_3$) δ 12.37 (br, 1H), 7.80 – 7.75 (m, 2H), 7.59 – 7.55 (m, 1H), 7.55 – 7.49 (m, 2H), 4.22 (s, 4H), 1.24 (s, 9H). **^{13}C NMR** (151 MHz, $CDCl_3$) δ 190.0, 178.4, 134.9, 133.4, 130.4, 127.7, 89.0, 72.1, 40.2, 27.1. **HRMS (ESI-MS) *m/z:*** calculated for $C_{16}H_{19}NO_4S$ [M + H]$^+$: 322.1108; Found: 322.1110.

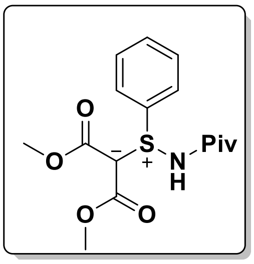

dimethyl 2-(phenyl(pivalamido)-λ^4-sulfaneylidene)malonate: (**7l** column solvent: PE/EA = 8/1, yellow oil, 17 mg, 25%): **^{1}H NMR** (500 MHz, $CDCl_3$) δ 10.14 (s, 1H), 7.57 – 7.44 (m, 5H), 3.73 (s, 6H), 1.28 (s, 9H). **^{13}C NMR** (151 MHz, $CDCl_3$) δ 177.6, 135.1, 131.4, 129. 8, 125.9, 63.4, 53.0, 51.5, 40.2, 27.1. **HRMS**

(ESI-MS) ***m/z:*** calculated for $C_{16}H_{21}NO_5S$ [*M* + H]$^+$: 340.1213; Found: 340.1212.

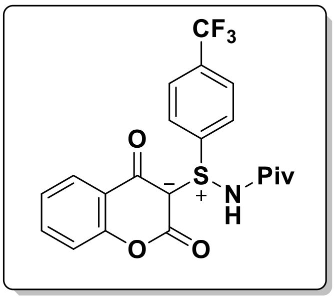

(*Z*)-*N*-((2,4-dioxochroman-3-ylidene)(4-(trifluoromethyl)phenyl)-λ^4-sulfaneyl)pivalamide (**7m** column solvent: PE/EA = 2/1, yellow solid, 82 mg, 94%): **^{1}H NMR** (500 MHz, $CDCl_3$) *δ* 12.99 (br, 1H), 8.04 – 7.95 (m, 3H), 7.78 (d, J = 8.4 Hz, 2H), 7.60 – 7.54 (m, 1H), 7.31 – 7.26 (m, 2H), 1.30 (s, 9H). **^{13}C NMR** (126 MHz, $CDCl_3$) *δ* 178.6, 177.8, 162.3, 154.6, 138.7, 135.1 (q, J = 33.4 Hz), 134.2, 128.0, 127.4 (q, J = 3.5 Hz), 125.4, 124.3, 126.5 – 119.7 (m), 120.9, 117.3, 80.2, 40.4, 27.1. **^{19}F NMR** (471 MHz, $CDCl_3$) δ -63.3. **HRMS (ESI-MS)** ***m/z:*** calculated for $C_{21}H_{19}F_3NO_4S$ [*M* + H]$^+$: 438.0981; Found: 438.0985.

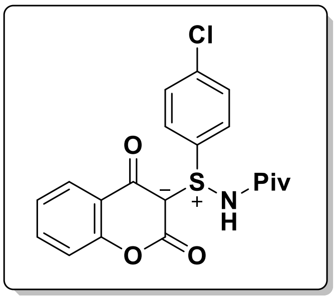

(*Z*)-*N*-((4-chlorophenyl)(2,4-dioxochroman-3-ylidene)-λ^4-sulfaneyl)pivalamide (**7n** column solvent: PE/EA = 2/1, white solid, 69 mg, 85%): **^{1}H NMR** (500 MHz, $CDCl_3$) *δ* 13.02 (br, 1H), 8.02 (dd, *J* = 7.8, 1.7 Hz, 1H), 7.84 (d, *J* = 8.7 Hz, 2H), 7.60 – 7.53 (m, 1H), 7.49 (d, *J* = 8.7 Hz, 2H), 7.32 – 7.23 (m, 2H), 1.28 (s, 9H). **^{13}C NMR** (126 MHz, $CDCl_3$) *δ* 178.6, 177.8, 162.3, 154.6, 140.4, 134.0, 133.1, 130.8, 129.2, 125.4, 124.2, 121.0, 117.3, 80.7, 40.3, 27.1. **HRMS (ESI-MS)** ***m/z:*** calculated for $C_{20}H_{19}ClNO_4S$ [*M* + H]$^+$: 404.0718; Found: 404.0725.

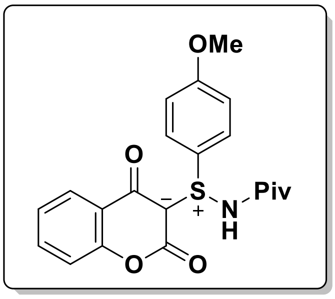

(*Z*)-*N*-((2,4-dioxochroman-3-ylidene)(4-methoxyphenyl)-λ^4-sulfaneyl)pivalamide (**7o** column solvent: PE/EA = 2/1, yellow solid, 58 mg, 73%): **^{1}H NMR** (500 MHz, $CDCl_3$) *δ* 13.04 (br, 1H), 8.04 (dd, *J* = 7.9, 1.7 Hz, 1H), 7.90 – 7.84 (m, 2H), 7.55 (td, *J* = 8.5, 1.7 Hz, 1H), 7.31 – 7.20 (m, 2H), 7.02 – 6.94 (m, 2H), 3.82 (s, 3H), 1.25 (s, 9H). **^{13}C NMR** (126 MHz, $CDCl_3$) *δ* 178.5, 177.6, 164.0, 162.4, 154.5, 133.8, 130.6, 129.1, 128.3, 125.4, 125.2, 124.1, 121.3, 117.2, 115.8, 82.0, 55.9, 40.3, 27.2. **HRMS (ESI-MS)** ***m/z:*** calculated for $C_{21}H_{22}NO_5S$ [*M* + H]$^+$: 400.1213; Found: 400.1209.

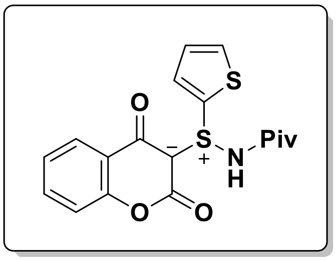

(Z)-N-((2,4-dioxochroman-3-ylidene)(thiophen-2-yl)-λ^4-sulfaneyl)pivalamide (**7p** column solvent: PE/EA = 2/1, yellow solid, 68 mg, 85%): **^{1}H NMR** (500 MHz, $CDCl_3$) δ 12.63 (s, 1H), 8.09 – 8.04 (m, 1H), 7.88 – 7.83 (m, 1H), 7.71 – 7.67 (m, 1H), 7.60 – 7.55 (m, 1H), 7.32 – 7.26 (m, 2H), 7.12 – 7.08 (m, 1H), 1.25 (s, 9H). **^{13}C NMR** (126 MHz, $CDCl_3$) δ 178.2, 154.6, 136.4, 135.4, 134.0, 132.8, 128.0, 125.6, 124.2, 121.1, 117.3, 83.2, 40.3, 27.1. **HRMS (ESI-MS) *m/z:*** calculated for $C_{18}H_{18}NO_4S_2$ $[M + H]^+$: 376.0672; Found: 376.0675.

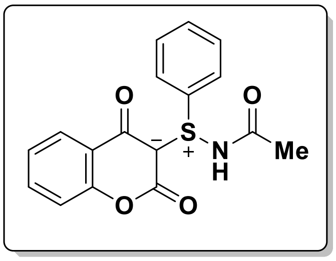

(Z)-N-((2,4-dioxochroman-3-ylidene)(phenyl)-λ^4-sulfaneyl)acetamide (**7q** column solvent: PE/EA = 2/1, white solid, 51 mg, 78%): **^{1}H NMR** (600 MHz, $CDCl_3$) δ 12.14 (br, 1H), 8.01 (dd, J = 7.9, 1.7 Hz, 1H), 7.87 (d, J = 7.3 Hz, 2H), 7.63 – 7.54 (m, 2H), 7.56 – 7.51 (m, 2H), 7.31 – 7.26 (m, 2H), 2.26 (s, 3H). **^{13}C NMR** (151 MHz, $CDCl_3$) δ 177.6, 169.5, 162.3, 154.6, 134.0, 133.6, 130.5, 127.8, 125.4, 124.2, 121.1, 117.4, 80.9, 23.1. **HRMS (ESI-MS) *m/z:*** calculated for $C_{17}H_{14}NO_4S$ $[M + H]^+$: 328.0638; Found: 328.0641.

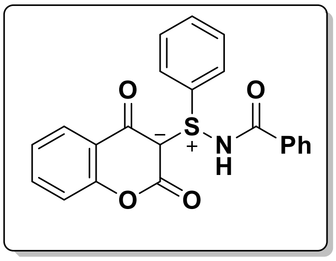

(Z)-N-((2,4-dioxochroman-3-ylidene)(phenyl)-λ^4-sulfaneyl)benzamide (**7r** column solvent: PE/EA = 2/1, yellow solid, 77 mg, 99%): **^{1}H NMR** (600 MHz, $CDCl_3$) δ 13.84 (s, 1H), 8.11 – 8.07 (m, 1H), 8.03 – 7.95 (m, 4H), 7.66 – 7.61 (m, 1H), 7.59 (p, J = 3.2 Hz, 2H), 7.55 – 7.50 (m, 4H), 7.33 – 7.28 (m, 2H). **^{13}C NMR** (151 MHz, $CDCl_3$) δ 177.6, 166.2, 162.4, 154.7, 134.6, 134.1, 134.0, 133.8, 130.5, 129.2, 128.6, 128.0, 125.5, 124.2, 121.1, 117.4, 81.2. **HRMS (ESI-MS) *m/z:*** calculated for $C_{22}H_{16}NO_4S$ $[M + H]^+$: 390.0795; Found: 390.0799.

VIII. *Scale-up Preparation and Transformation Experiments*

(1) Scale-up preparation

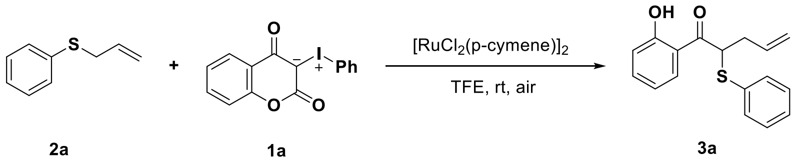

In a 100 mL reaction tube, the mixture of **1a** (300.5 mg, 2.0 mmol, 1.0 equiv.), **2a** (1.46 g, 4.0 mmol, 2.0 equiv.) and [RuCl$_2$(p-cymene)]$_2$ (30.6 mg, 0.05 mmol, 2.5 mol%) was added to TFE (15 mL). Then the resulting mixture was stirred at room temperature for 12.0 h. When the reaction was finished, the product was purified by preparative thin layer chromatography (PE/EA = 20/1, v/v). The product **3a** was obtained as a yellow oil (379 mg, 66% yield).

(2) Transformation of products

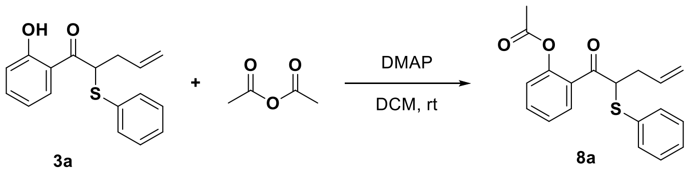

In a 10 mL reaction tube, acetic anhydride (57 μL, 6.0 equiv.) and 4-(Dimethylamino) pyridine (1 mg, 0.1 equiv.) were added to a solution of **3a** (28.4 mg, 0.1 mmol, 1.0 equiv.) in dry DCM (2.0 mL) at room temperature for 12.0 h. When the reaction was finished, the product was purified by preparative thin layer chromatography (PE/EA = 20/1, v/v). Product **8a** was obtained as a yellow oil (33 mg, 97% yield).

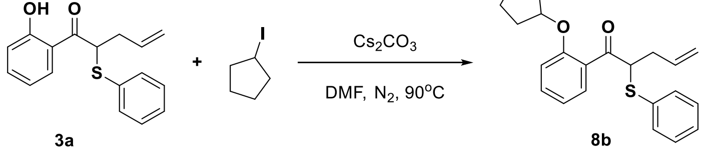

In a 10 mL reaction tube, compound **3a** (28.4 mg, 0.1 mmol, 1.0 equiv.) iodocyclopentane (39.2 mg, 0.2 mol, 2.0 equiv.), and Cs_2CO_3 (65.2 mg, 0.2 mmol, 2.0

equiv.) were dissolved in DMF (3 mL). Then the resulting mixture was stirred and refluxed at 90 ^{o}C for 12.0 h. When the reaction was finished, the product was separated by thin layer chromatography plates (PE/EA = 20/1), product **8b** was obtained as a yellow oil (15 mg, 43% yield).

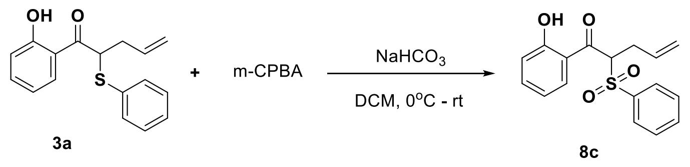

In a 10 mL reaction tube, compound **3a** (28.4 mg, 0.1 mmol, 1.0 equiv.) m-CPBA (51.8 mg, 0.3 mol, 3.0 equiv.), and $NaHCO_3$ (8.4 mg, 0.1 mmol, 1.0 equiv.) were dissolved in DCM (3 mL). Then the resulting mixture was stirred at room temperature for 3.0 h. When the reaction was finished, the product was separated by thin layer chromatography plates (PE/EA = 10/1), product **8c** was obtained as a white solid (27 mg, 85% yield).

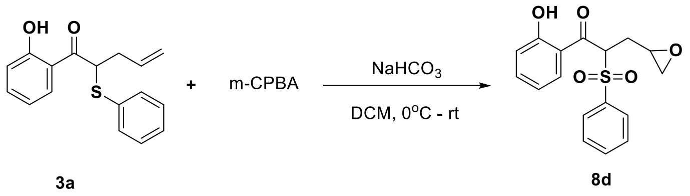

In a 10 mL reaction tube, compound **3a** (28.4 mg, 0.1 mmol, 1.0 equiv.) m-CPBA (86.2 mg, 0.5 mol, 5.0 equiv.), and $NaHCO_3$ (8.4 mg, 0.1 mmol, 1.0 equiv.) were dissolved in DCM (3 mL). Then the resulting mixture was stirred at room temperature for 48.0 h. When the reaction was finished, the product was separated by thin layer chromatography plates (DCM/MeOH = 40/1), product **8d** was obtained as a yellow solid (15 mg, 45% yield).

IX. *Characterization Data of Products 8*

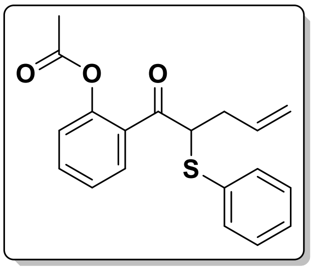

2-(2-(phenylthio)pent-4-enoyl)phenyl acetate (**8a**, column solvent: PE/EA = 20:1, yellow oil, 33 mg, 97%): **^{1}H NMR** (500 MHz, Chloroform-*d*) δ 7.70 (dd, *J* = 7.8, 1.7 Hz, 1H), 7.53 – 7.50 (m, 1H), 7.37 – 7.35 (m, 2H), 7.31 – 7.29 (m, 1H), 7.28 – 7.25 (m, 3H), 7.14 (dd, *J* = 8.1, 1.2 Hz, 1H), 5.92 – 5.84 (m, 1H), 5.16 – 5.09 (m, 2H), 4.35 (t, *J* = 7.3 Hz, 1H), 2.73 – 2.67 (m, 1H), 2.54 – 2.48 (m, 1H), 2.29 (s, 3H). **^{13}C NMR** (126 MHz, Chloroform-*d*) δ 194.8, 169.4, 149.4, 135.2, 134.8, 133.1, 131.1, 130.5, 129.9, 129.0, 128.9, 126.1, 124.3, 118.0, 53.7, 34.8, 21.2. **HRMS (ESI) *m/z*:** calculated for $C_{19}H_{18}O_3S$ [*M* + H]$^-$: 327.1049, found: 327.1048.

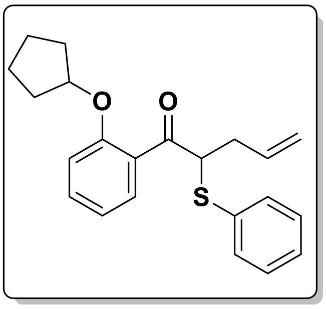

1-(2-(cyclopentyloxy)phenyl)-2-(phenylthio)pent-4-en-1-one (**8b**, column solvent: PE/EA = 20:1, yellow oil, 15 mg, 43%): **^{1}H NMR** (500 MHz, Chloroform-*d*) δ 7.68 (dd, *J* = 7.6, 1.9 Hz, 1H), 7.41 – 7.37 (m, 1H), 7.28 – 7.19 (m, 5H), 6.99 – 6.96 (m, 1H), 6.86 (d, *J* = 8.3 Hz, 1H), 5.97 – 5.89 (m, 1H), 5.15 – 5.08 (m, 2H), 4.85 (t, *J* = 7.2 Hz, 1H), 4.86 – 4.78 (m, 1H), 2.75 – 2.69 (m, 1H), 2.53 – 2.46 (m, 1H), 1.94 – 1.81 (m, 4H), 1.79 – 1.72 (m, 2H), 1.67 – 1.60 (m, 2H). **^{13}C NMR** (151 MHz, Chloroform-*d*) δ 198.0, 156.5, 135.4, 134.6, 133.2, 132.2, 131.8, 128.8, 128.4, 128.0, 120.4, 117.3, 113.2, 80.1, 55.1, 34.4, 33.0, 32.9, 24.2, 24.2. **HRMS (ESI) *m/z*:** calculated for $C_{22}H_{24}O_2S$ [*M* + H]$^-$: 353.1570, found: 353.1568.

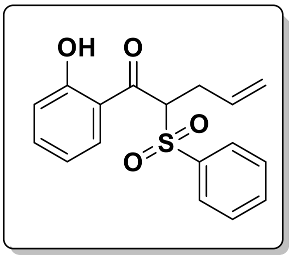

1-(2-hydroxyphenyl)-2-(phenylsulfonyl)pent-4-en-1-one (**8c**, column solvent: PE/EA = 10:1, white solid, 27 mg, 85%): **^{1}H NMR** (600 MHz, Chloroform-*d*) δ 11.77 (s, 1H), 7.79 (dd, *J* = 8.3, 1.4 Hz, 2H), 7.74 (dd, *J* = 8.2, 1.6 Hz, 1H), 7.66 – 7.63 (m, 1H), 7.53 – 7.48 (m, 3H), 6.97 (dd, *J* = 8.5, 1.2 Hz, 1H), 6.91 – 6.89 (m, 1H), 5.62 – 5.55 (m, 1H), 5.15 (dd, *J* = 10.5, 4.1 Hz, 1H), 5.08 – 5.04 (m, 1H), 5.00 (dd, *J* = 10.2, 1.4 Hz, 1H), 2.91 – 2.82 (m, 2H). **^{13}C NMR** (151 MHz, Chloroform-*d*) δ 196.8, 163.2, 137.8, 136.3, 134.6, 131.7, 131.0, 129.8, 129.2, 120.4, 119.5, 119.4, 118.8, 68.9, 32.0. **HRMS (ESI) *m/z*:** calculated for $C_{17}H_{16}O_4S$ [*M* + H]$^-$: 317.0842, found: 317.0842.

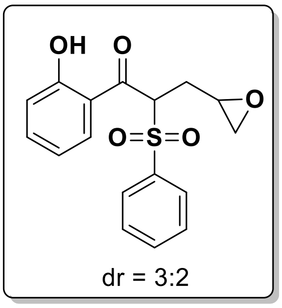

1-(2-hydroxyphenyl)-3-(oxiran-2-yl)-2-(phenylsulfonyl)propan-1-one (**8d**, DCM/MeOH = 40:1, yellow solid, 15 mg, 45%): **^{1}H NMR** (500 MHz, Chloroform-*d*) δ 11.74 (s, 3H), 11.66 (s, 2H), 7.78 – 7.75 (m, 9H), 7.75 – 7.72 (m, 6H), 7.66 – 7.62 (m, 5H), 7.53 – 7.48 (m, 15H), 6.99 – 6.96 (m, 4H), 6.92 – 6.87 (m, 6H), 5.27 – 5.23 (m, 5H), 3.10 – 3.06 (m, 2H), 2.81 – 2.77 (m, 3H), 2.73 – 2.71 (m, 5H), 2.66 – 2.59 (m, 5H), 2.54 (dd, *J* = 4.9, 2.5 Hz, 3H), 2.42 (dd, *J* = 4.7, 2.6 Hz, 2H), 2.31 – 2.25 (m, 2H), 2.17 – 2.13 (m, 3H). **^{13}C NMR** (126 MHz, Chloroform-*d*) δ 196.7, 196.6, 163.3, 138.1, 137.9, 136.2, 136.2, 134.7, 131.1, 130.8, 129.7, 129.7, 129.3, 129.3, 120.1, 119.7, 119.6, 119.6, 118.9, 118.8, 66.8, 66.6, 49.6, 48.8, 47.9, 47.6, 30.8, 30.6. **HRMS (ESI) *m/z*:** calculated for $C_{17}H_{16}O_5S$ [M + H]$^-$: 333.0791, found: 333.0789.

X. *Mechanism Study*

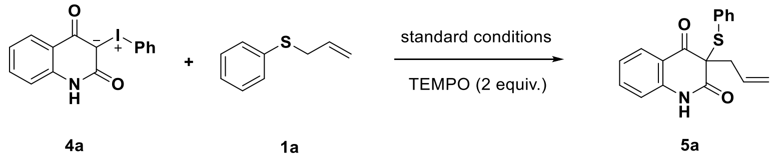

To a mixture of **4a** (0.2 mmol), **1a** (0.28 mmol), TEMPO (0.4 mmol) and $[RuCl_2(p\text{-cymene})]_2$ (2.5 mol%) in a 10 mL reaction tube was added TFE (2 mL), then the resulting mixture was stirred at room temperature for 12.0 h. After removing the solvent under vacuum, the residue was purified by column chromatography on silica gel (PE/EA = 3:1) to afford product **5a** (yellow solid, 56%).

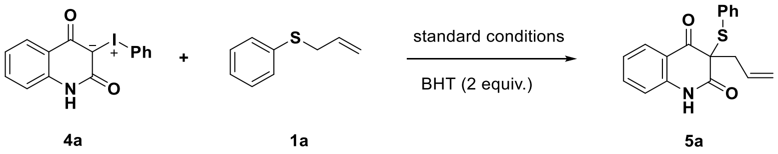

To a mixture of **4a** (0.2 mmol), **1a** (0.28 mmol), BHT (0.4 mmol) and $[RuCl_2(p\text{-cymene})]_2$ (2.5 mol%) in a 10 mL reaction tube was added TFE (2 mL), then the resulting mixture was stirred at room temperature for 12.0 h. After removing the solvent under vacuum, the residue was purified by column chromatography on silica gel (PE/EA = 3:1) to afford product **5a** (yellow solid, 69%).

Investigation of Intermediate 5

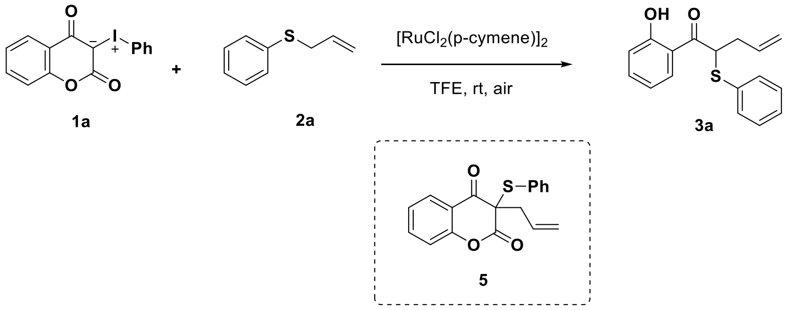

To a mixture of **1a** (0.4 mmol), **2a** (0.2 mmol) and $[RuCl_2(\text{p-cymene})]_2$ (2.5 mol%) in a 10 mL reaction tube was added TFE (3 mL), then the resulting mixture was stirred at room temperature for 12.0 h. Following a brief reaction period, the crude

mixture was purified by preparative TLC (PE/EA = 20/1, v/v), yielding a mixture. LC-MS analysis revealed a prominent signal for the molecular weight of intermediate **5** and a minor signal for the target molecular weight (**3a**).

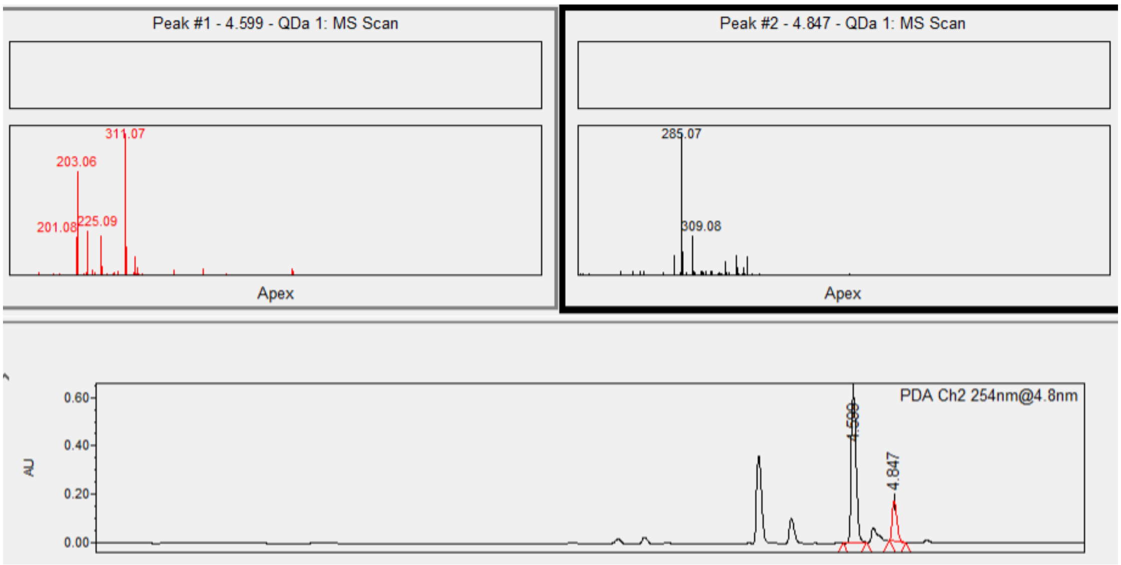

XI. *X-ray Crystallographic Data*

The Single Crystal Structure of 3j

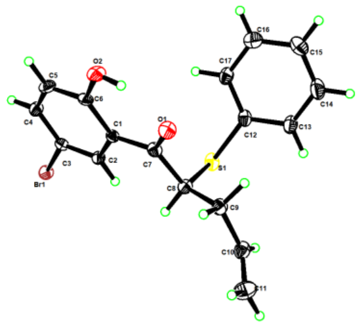

Crystal structure of **3j (Deposition Data: CCDC 2444095)**, showing an ellipsoid contour probability level of 50%.

Table S2. Crystal data and structure refinement for compound 3j.

Identification code	**3j**
Empirical formula	$C_{17}H_{15}BrO_2S$
Formula weight	363.26
Temperature/K	170
Crystal system	triclinic
Space group	P-1
a/Å	7.0105(4)
b/Å	7.6775(4)
c/Å	15.0372(9)
α/°	82.337(2)
β/°	87.723(2)
γ/°	78.647(2)
Volume/Å^3	786.35(8)
Z	2
ρ_{calc}g/cm^3	1.534
μ/mm^{-1}	2.747
F (000)	368.0
Crystal size/mm^3	0.15 × 0.06 × 0.05
Radiation	MoKα (λ = 0.71073)
2Θ range for data collection/°	5.456 to 52.792

Index ranges	$-8 \leq h \leq 8$, $-9 \leq k \leq 9$, $-18 \leq l \leq 18$
Reflections collected	9221
Independent reflections	3214 [R_{int} = 0.0566, R_{sigma} = 0.0637]
Data/restraints/parameters	3214/0/192
Goodness-of-fit on F^2	1.062
Final R indexes [I>=2σ (I)]	R_1 = 0.0382, wR_2 = 0.0881
Final R indexes [all data]	R_1 = 0.0471, wR_2 = 0.0915
Largest diff. peak/hole / e $Å^{-3}$	0.48/-0.74

The Single Crystal Structure of 5a

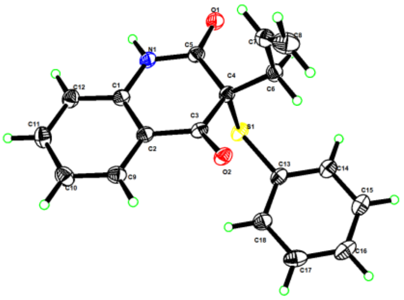

Crystal structure of **5a (Deposition Data: CCDC 2444096)**, showing an ellipsoid contour probability level of 50%.

Table S3. Crystal data and structure refinement for compound 5a.

Identification cod	**5a**
Empirical formula	$C_{18}H_{15}NO_2S$
Formula weight	309.37
Temperature/K	173
Crystal system	monoclinic
Space group	P21/n
a/Å	7.8830(3)
b/Å	18.5727(7)
c/Å	10.4373(3)
α/°	90

β/°	95.3060(10)
γ/°	90
Volume/Å^3	1521.56(9)
Z	4
ρ_{calc}g/cm^3	1.351
μ/mm^{-1}	0.219
F (000)	648.0
Crystal size/mm^3	0.15 × 0.08 × 0.05
Radiation	MoKα (λ = 0.71073)
2Θ range for data collection/°	4.386 to 52.092
Index ranges	$-9 \le h \le 9$, $-22 \le k \le 22$, $-12 \le l \le 11$
Reflections collected	12800
Independent reflections	2930 [R_{int} = 0.0692, R_{sigma} = 0.0507]
Data/restraints/parameters	2930/0/203
Goodness-of-fit on F^2	1.048
Final R indexes [I>=2σ (I)]	R_1 = 0.0427, wR_2 = 0.1046
Final R indexes [all data]	R_1 = 0.0582, wR_2 = 0.1144
Largest diff. peak/hole / e Å^{-3}	0.19/-0.23

The Single Crystal Structure of 7o

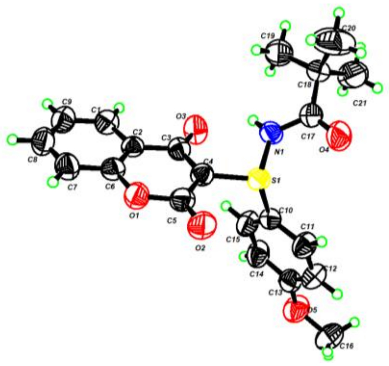

Crystal structure of **7o (Deposition Data: CCDC 2444974)**, showing an ellipsoid contour probability level of 50%.

Table S5. Crystal data and structure refinement for compound 7o.

Identification cod	**7o**
Empirical formula	$C_{21}H_{21}NO_5S$
Formula weight	399.45
Temperature/K	300.00
Crystal system	orthorhombic
Space group	Pbca
a/Å	15.997(2)
b/Å	12.827(2)
c/Å	19.115(2)
α/°	90
β/°	90
γ/°	90
Volume/Å^3	3922.4(10)
Z	8
ρ_{calc}g/cm^3	1.353
μ/mm^{-1}	0.198
F(000)	1680.0
Crystal size/mm^3	0.16 × 0.15 × 0.12
Radiation	MoKα (λ = 0.71073)
2Θ range for data collection/°	4.594 to 52.758
Index ranges	$-19 \le h \le 18$, $-15 \le k \le 16$, $-23 \le l \le 23$
Reflections collected	19622
Independent reflections	3987 [R_{int} = 0.1084, R_{sigma} = 0.0758]
Data/restraints/parameters	3987/0/258
Goodness-of-fit on F^2	1.014
Final R indexes [I>=2σ (I)]	R_1 = 0.0548, wR_2 = 0.1319
Final R indexes [all data]	R_1 = 0.1052, wR_2 = 0.1711
Largest diff. peak/hole / e Å^{-3}	0.29/-0.26

XII. *References*

[1] Liang M, He M, Zhong Z, Wan B, Du Q, Mai S. Catalytic and Base-free Suzuki-type α-Arylation of Cyclic 1,3-Dicarbonyls via a Cyclic Iodonium Ylide Strategy. *Angew. Chem. Int. Ed.* **2024,** *63*, e202400741.

[2] Nolan K, Doncaster J, Dunstan M, Scott K, Frenkel A, Siegel D, Ross D, Barnes J, Levy C, Leys D, Whitehead R, Stratford I, Bryce R. Synthesis and biological evaluation of coumarin-based inhibitors of NAD(P)H: quinone oxidoreductase-1 (NQO1). *J. Med. Chem.* **2009,** *52*, 7142-56.

[3] Ying H, Zhi L, Xiao G, Jiao L, Can L, Qian W, Yong W. Rhodium-catalyzed Doyle-Kirmse rearrangement reactions of sulfoxoniun ylides. *Chinese Chemical Letters*. **2024,** *35*, 389-392.

[4] Jana S, Koenigs RM. Rhodium-Catalyzed Carbene Transfer Reactions for Sigmatropic Rearrangement Reactions of Selenium Ylides. *Org. Lett.* **2019,** *21*, 3653-3657.

[5] Liu X, Shi S, Ding W, et al. Accessing 7,8-Dihydroquinoline-2,5-diones via Rh-Catalyzed Olefinic C-H Activation/[4+2] Cyclization. *Org. Lett.* **2024,** *26*, 5136-5140.

[6] Ren, J.; Yang, L.; Pi, C.; Cui, X.; Wu, Y. Rhodium(III)-Catalyzed Divergent C−H Functionalization of N-Aryl Amidines with Iodonium Ylides: Access to Carbazolones and Zwitterionic Salts. *Adv. Synth. Catal.* **2023,** *365*, 1817–1823.

[7] Sen S, Barman D, Khan H, Das R, Maiti D. Cu(II)-Catalyzed Multicomponent Reaction of Pyridine Derivatives/Isoquinolines with Iodonium Ylide and 1,4-Quinones Using Mechanochemistry. *J. Org. Chem.* **2022**, *87*, 12164-12174.

[8] Yuan Y, Han Y, Zhang ZK, Sun S, Wu K, Yang J, Zhang J. Enantioselective Arylation of Sulfenamides to Access Sulfilimines Enabled by Palladium Catalysis. *Angew. Chem. Int. Ed.* **2024**, *63*, e202409541.

XIII. *NMR Spectra*

1-(2-hydroxyphenyl)-2-(phenylthio)pent-4-en-1-one (3a)

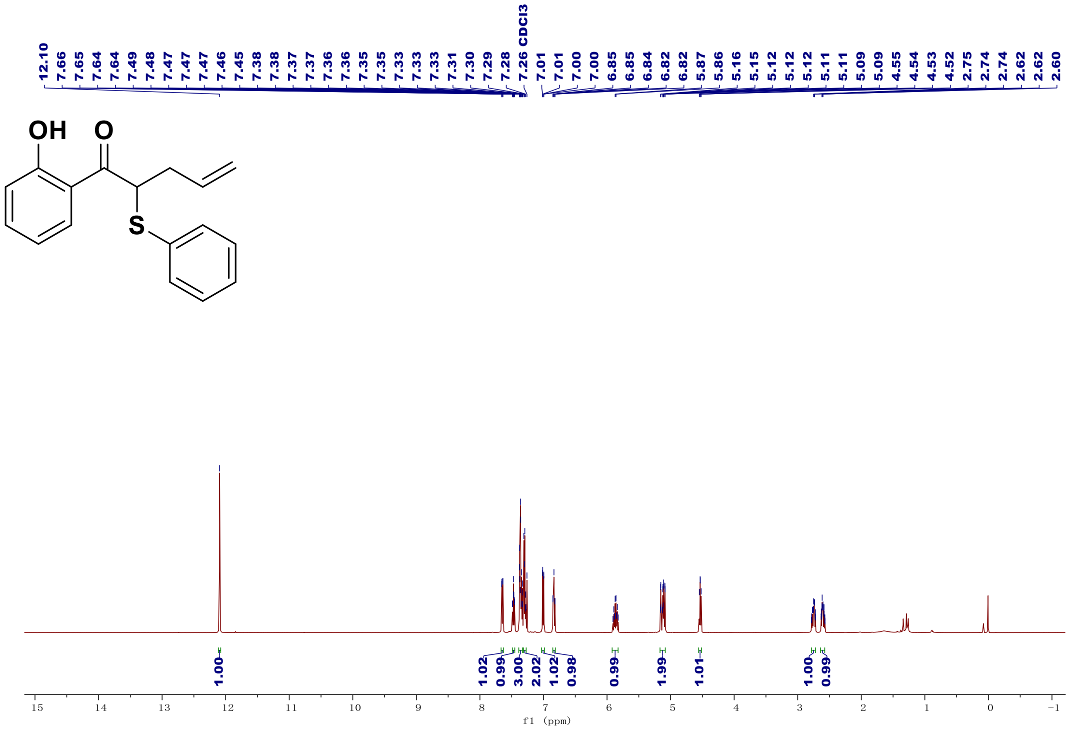

^{1}H NMR spectrum for product **3a**

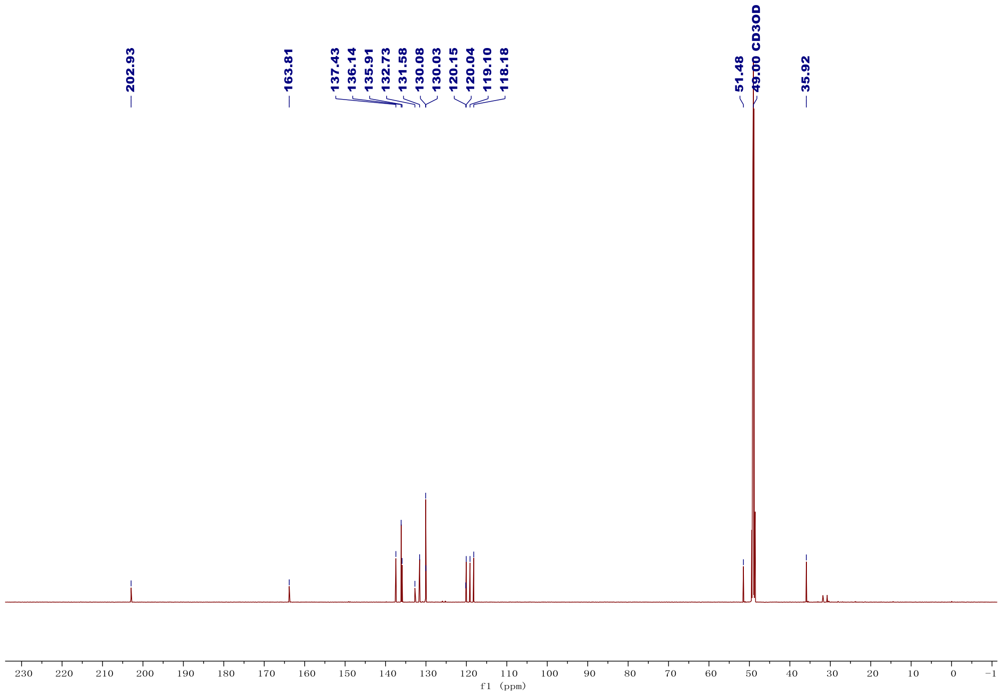

^{13}C NMR spectrum for product **3a**

1-(2-hydroxy-3-methylphenyl)-2-(phenylthio)pent-4-en-1-one (3b)

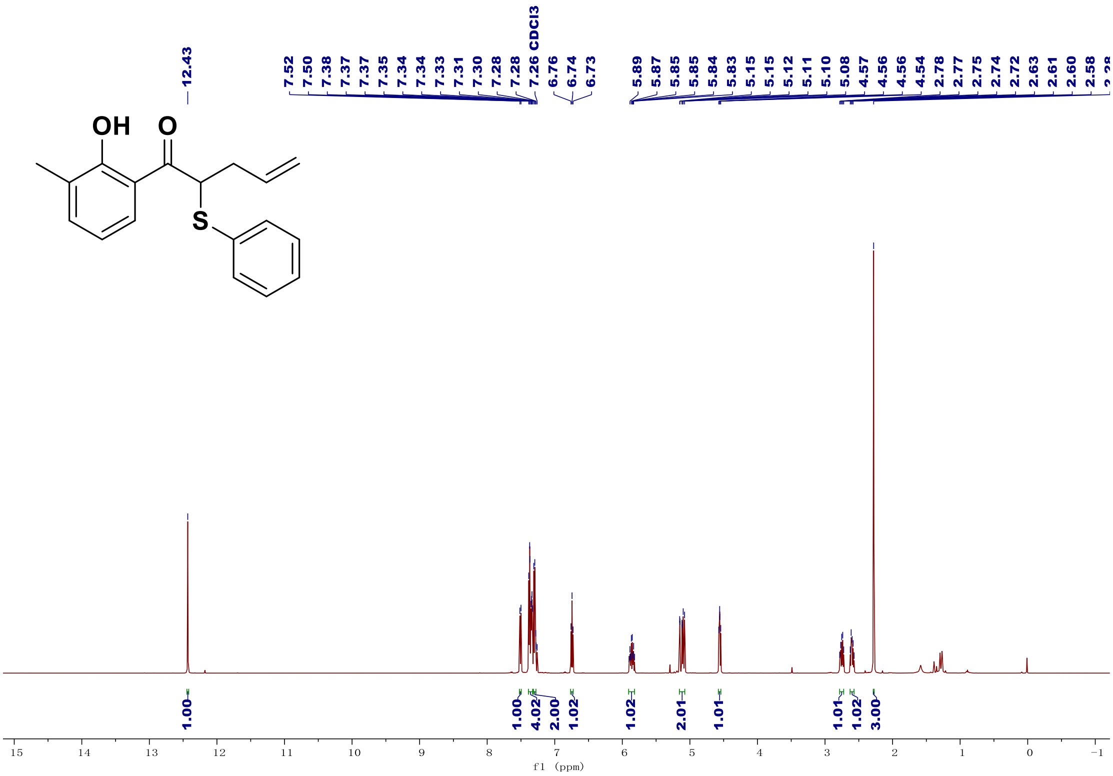

^{1}H NMR spectrum for product **3b**

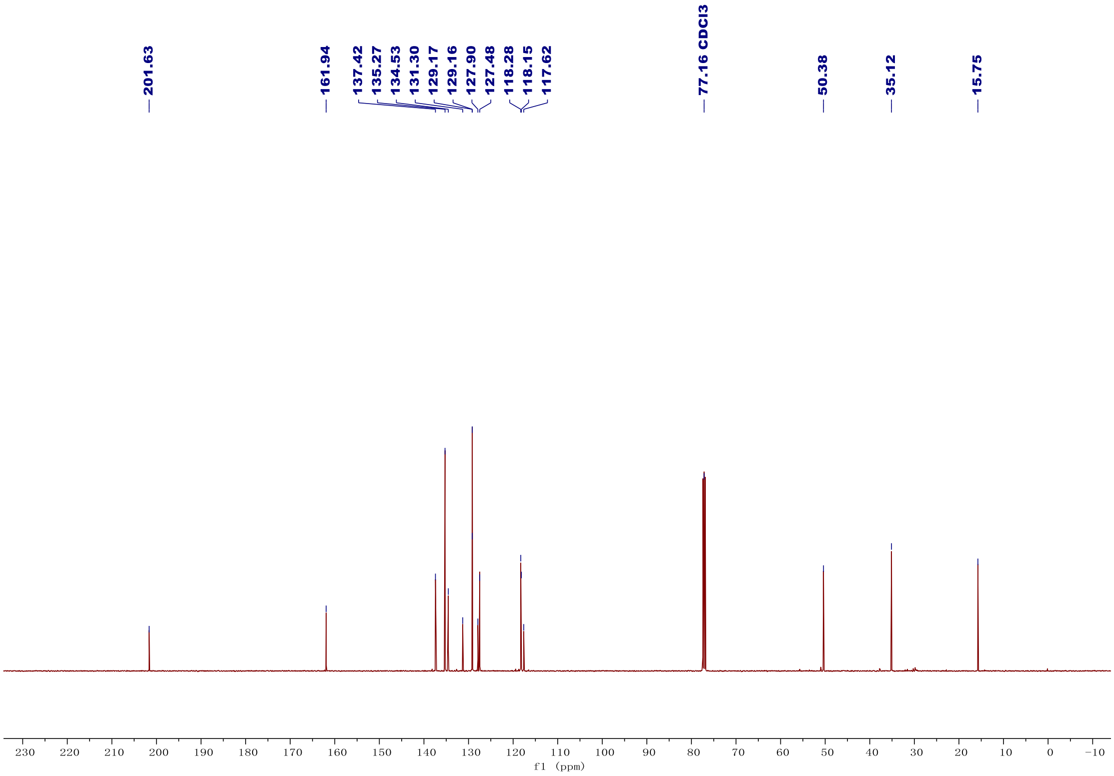

^{13}C NMR spectrum for product **3b**

1-(2-hydroxy-4-methylphenyl)-2-(phenylthio)pent-4-en-1-one (3c)

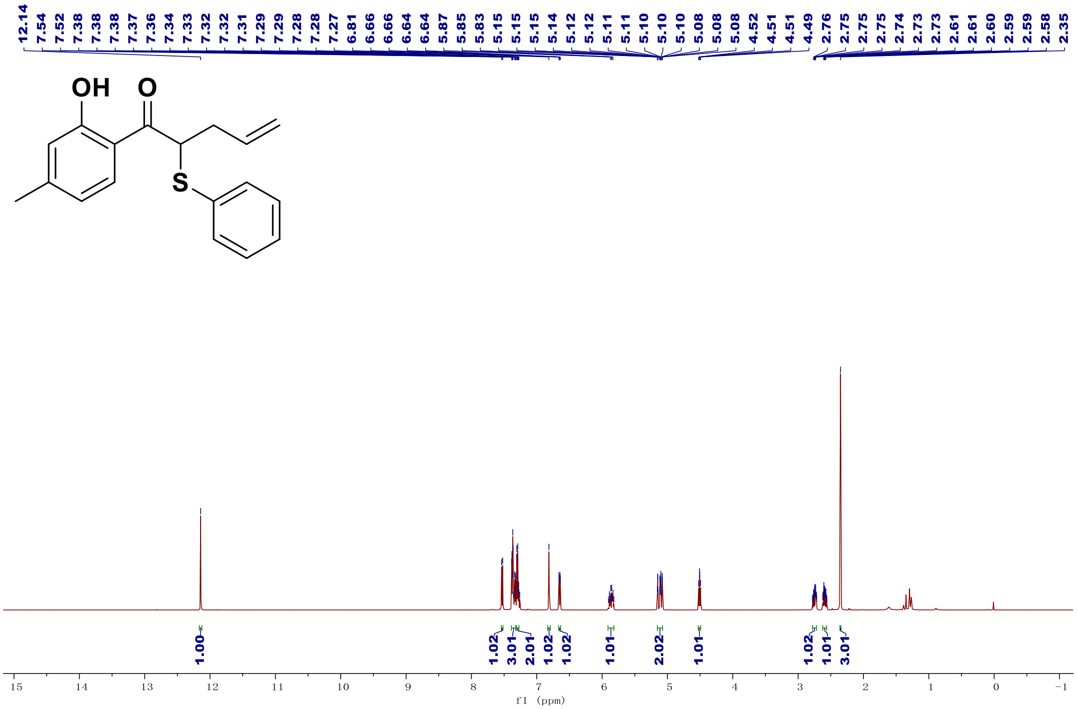

^{1}H NMR spectrum for product **3c**

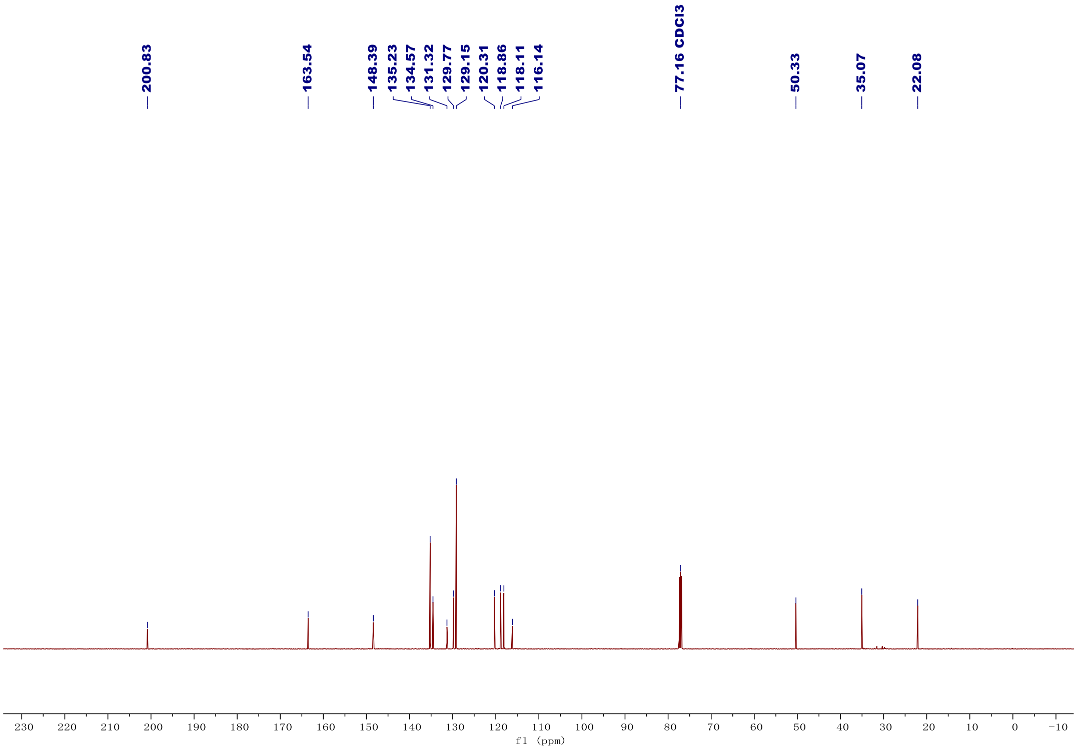

^{13}C NMR spectrum for product **3c**

1-(2-hydroxy-4-methoxyphenyl)-2-(phenylthio)pent-4-en-1-one (3d)

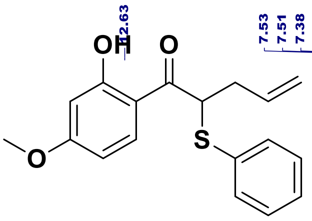

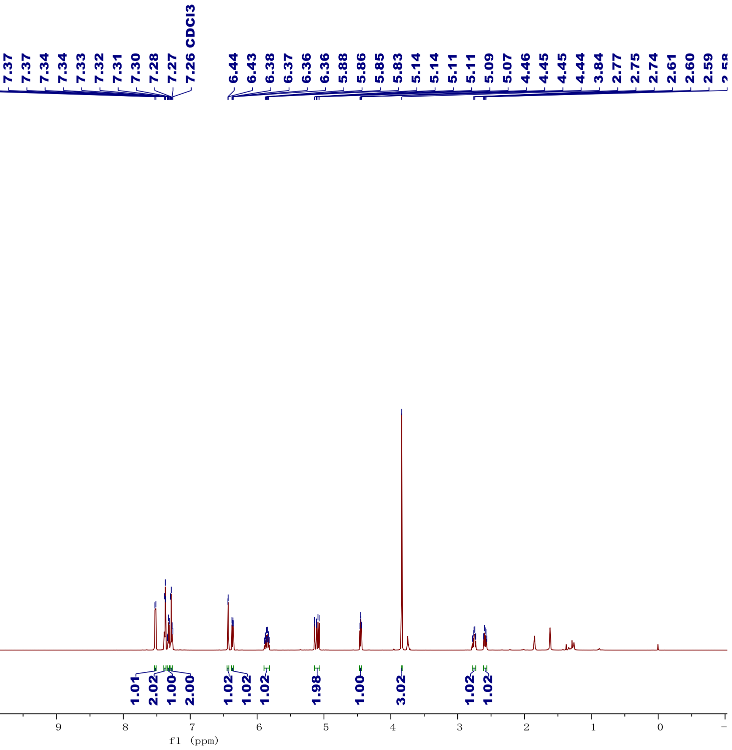

^{1}H NMR spectrum for product **3d**

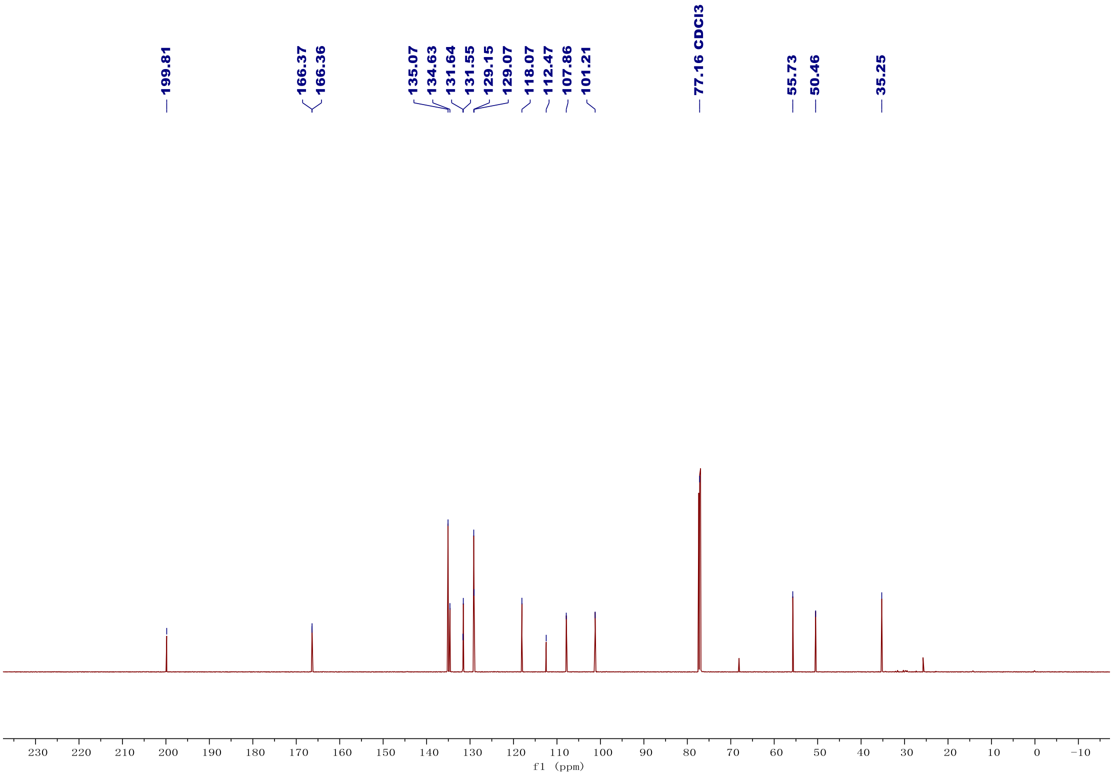

^{13}C NMR spectrum for product **3d**

1-(4-fluoro-2-hydroxyphenyl)-2-(phenylthio)pent-4-en-1-one (3e)

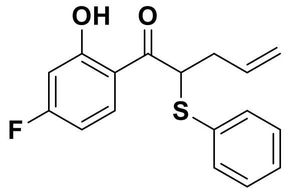

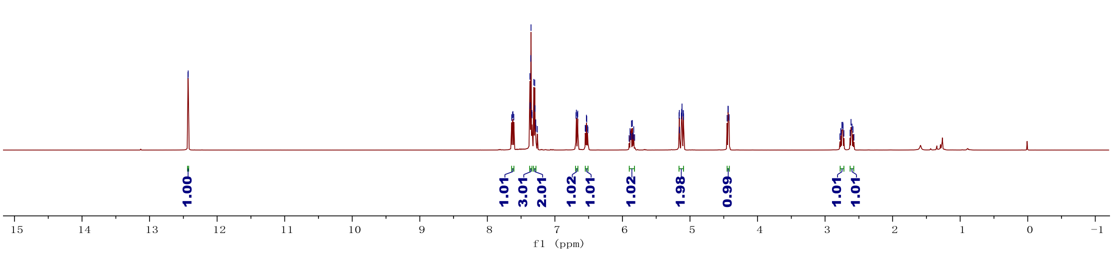

^{1}H NMR spectrum for product **3e**

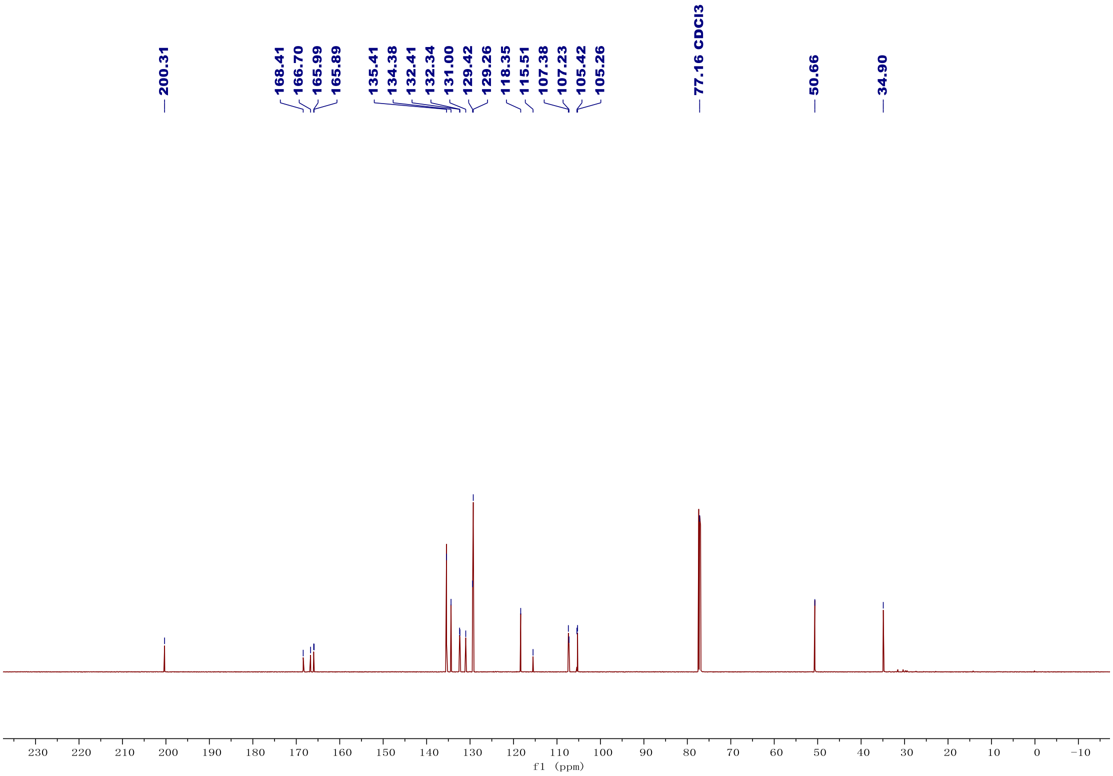

^{13}C NMR spectrum for product **3e**

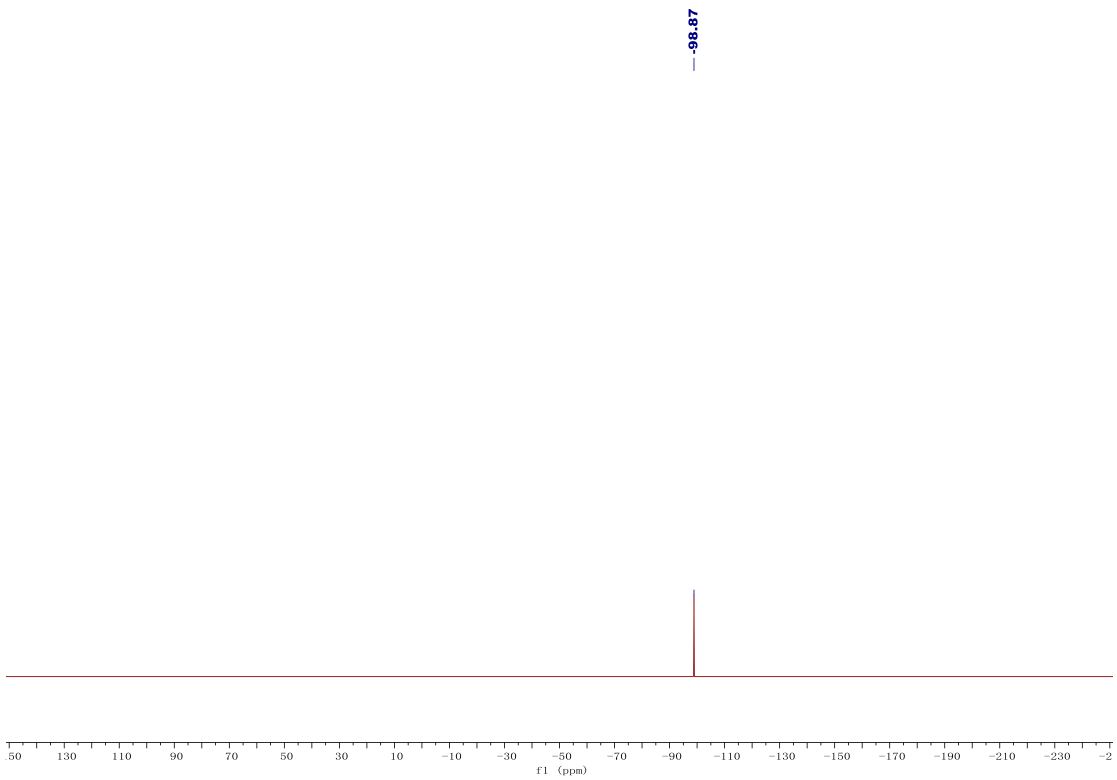

^{19}F NMR spectrum for product **3e**

1-(2-hydroxy-5-methylphenyl)-2-(phenylthio)pent-4-en-1-one (3f)

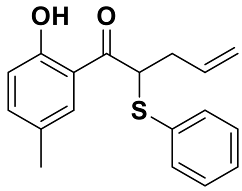

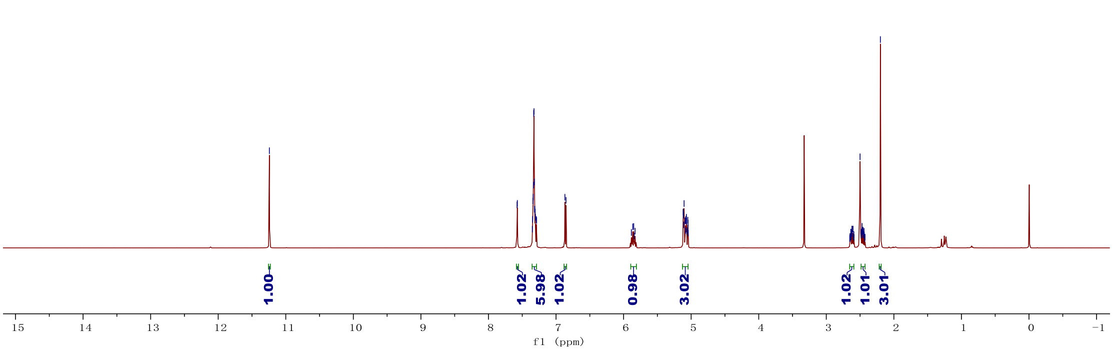

^{1}H NMR spectrum for product **3f**

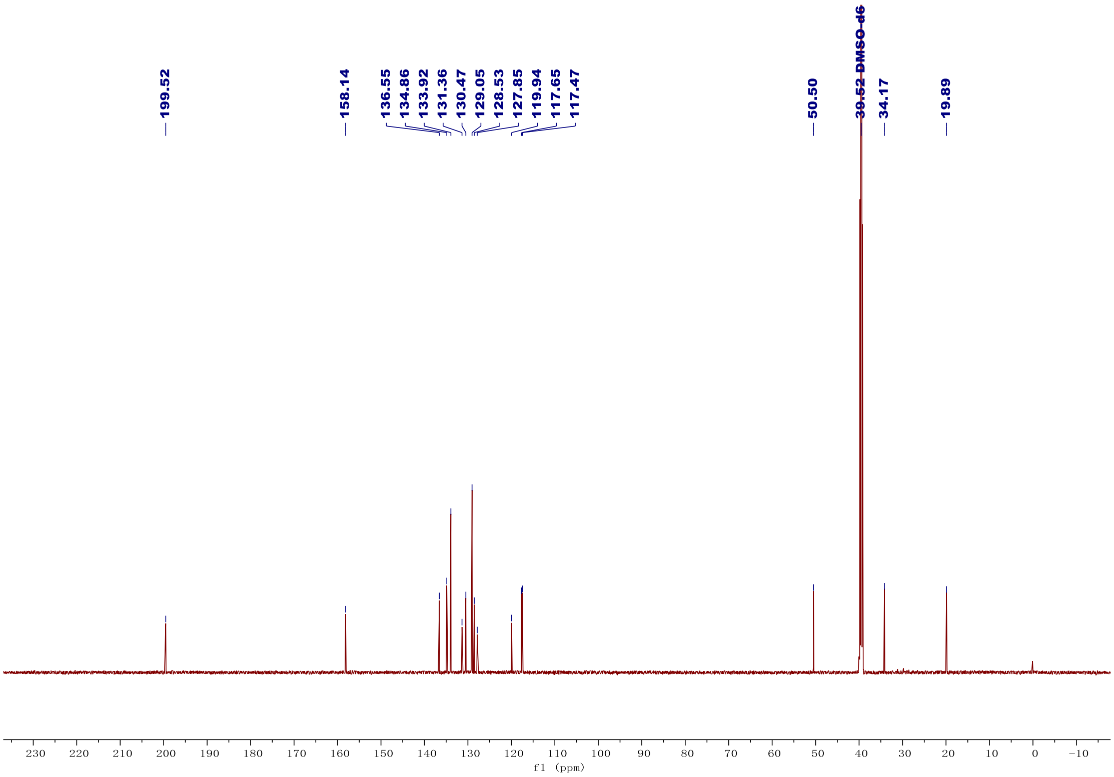

^{13}C NMR spectrum for product **3f**

1-(2-hydroxy-5-methoxyphenyl)-2-(phenylthio)pent-4-en-1-one (3g)

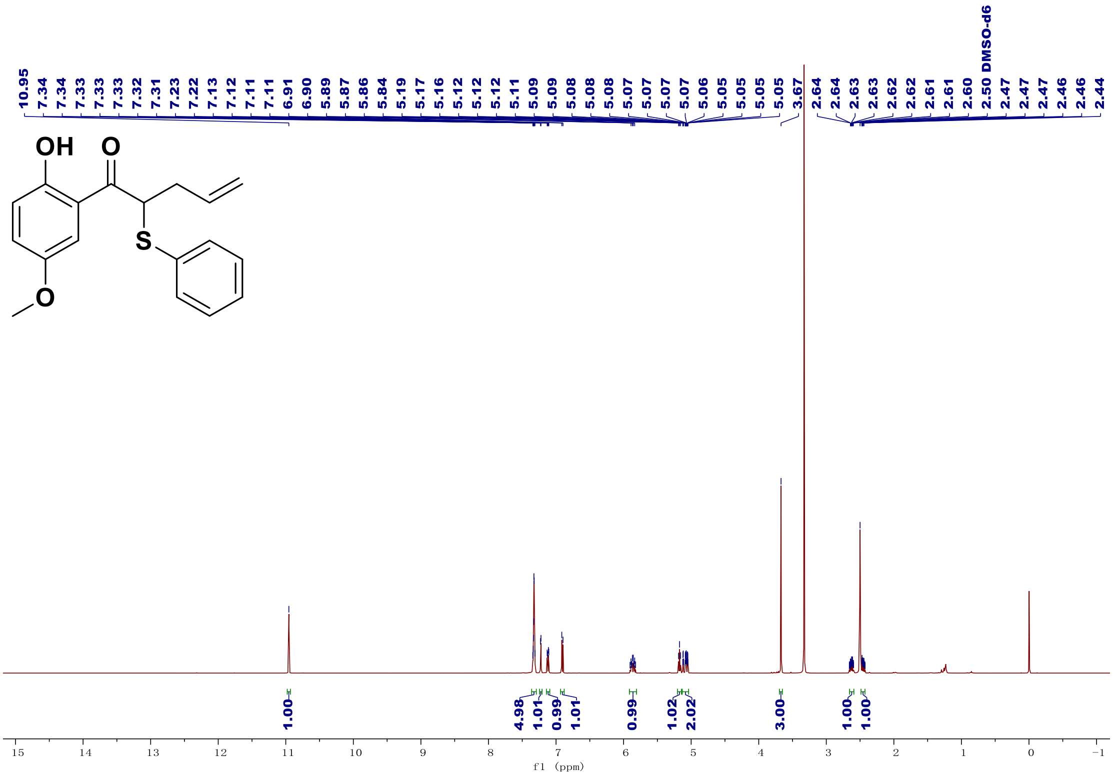

^{1}H NMR spectrum for product **3*g***

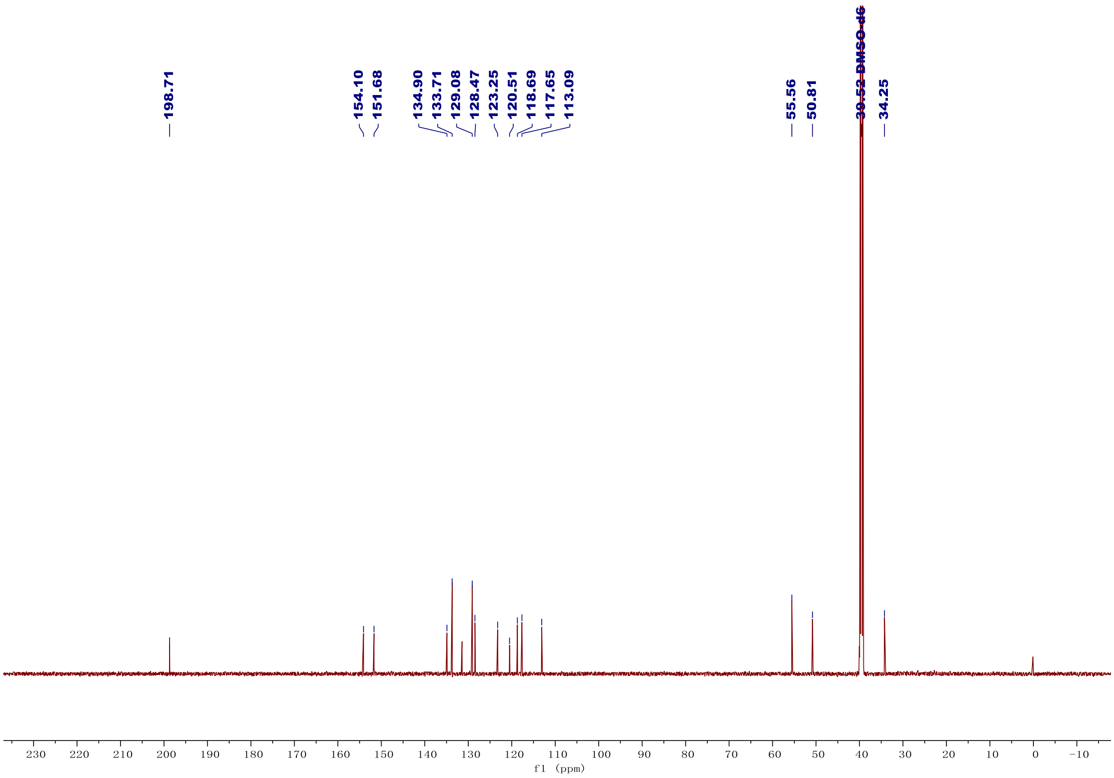

^{13}C NMR spectrum for product **3g**

1-(5-fluoro-2-hydroxyphenyl)-2-(phenylthio)pent-4-en-1-one (3h)

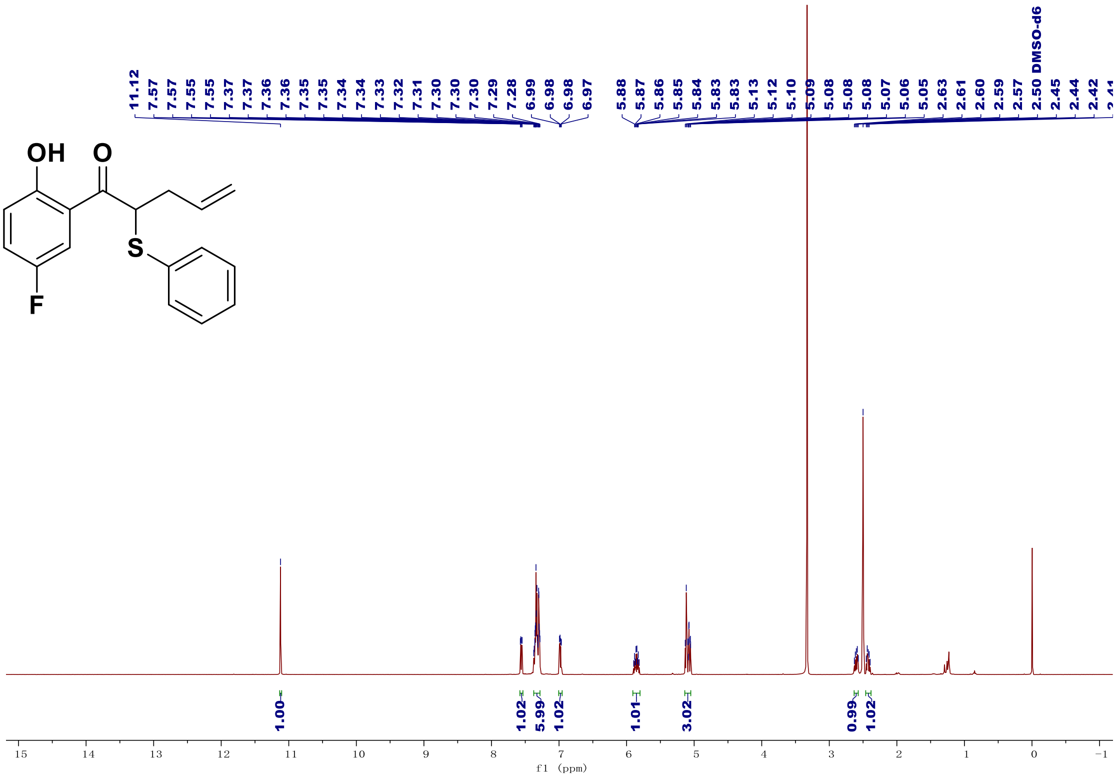

^{1}H NMR spectrum for product **3h**

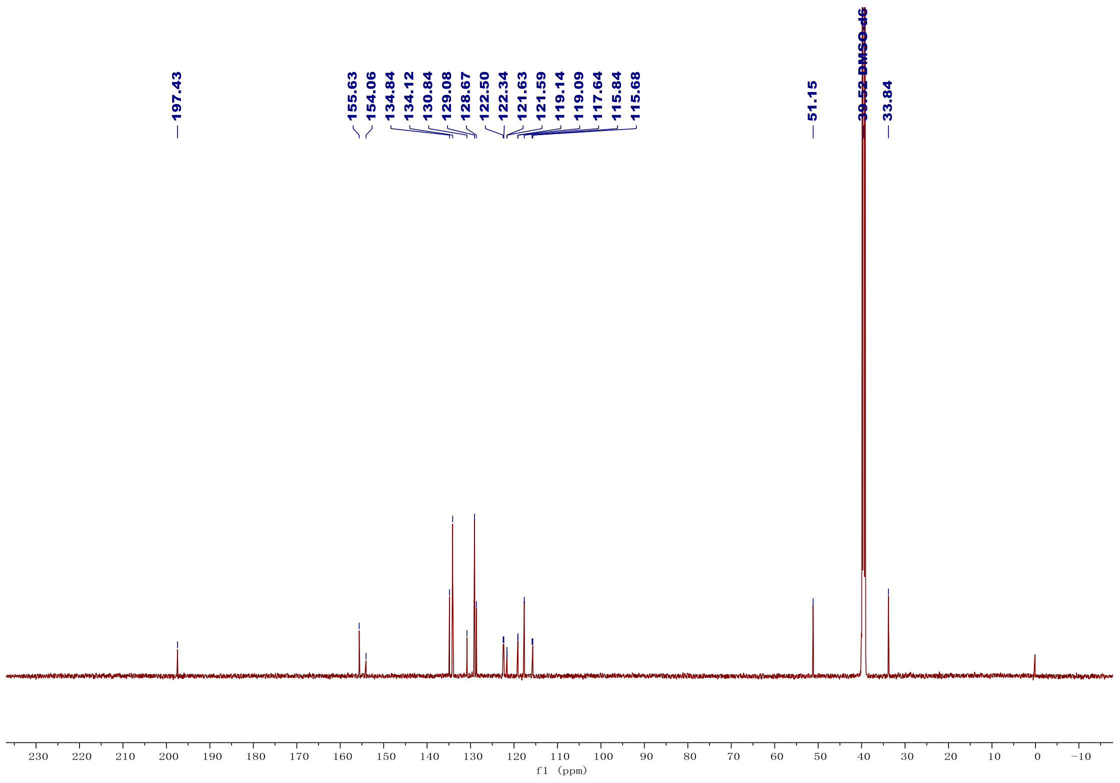

^{13}C NMR spectrum for product **3h**

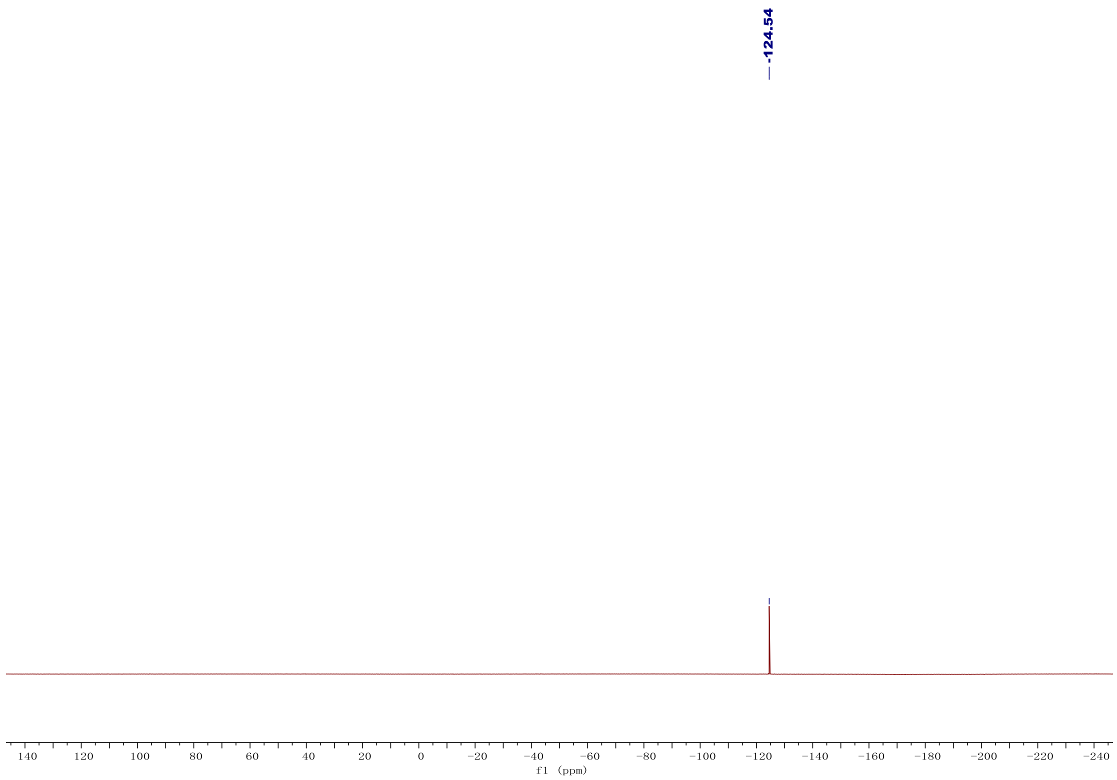

^{19}F NMR spectrum for product **3h**

1-(5-chloro-2-hydroxyphenyl)-2-(phenylthio)pent-4-en-1-one (3i)

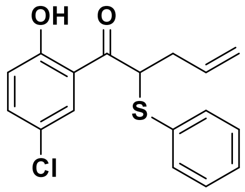

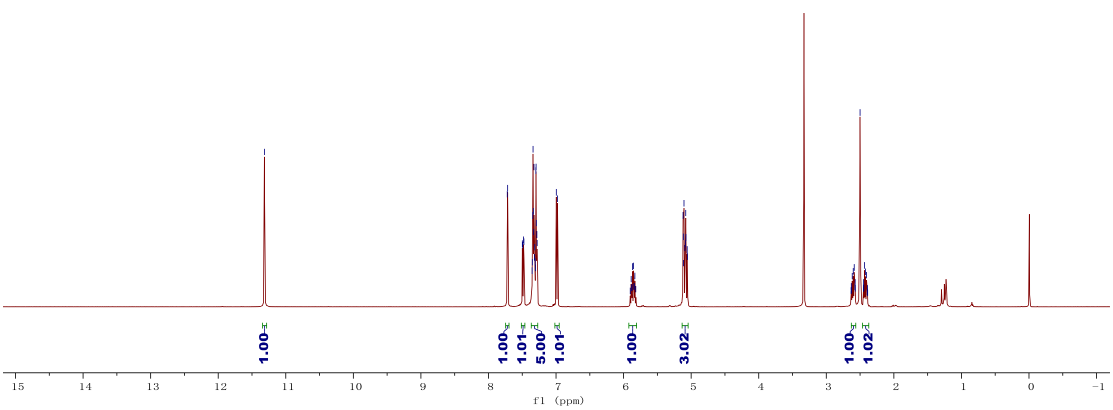

^{1}H NMR spectrum for product **3i**

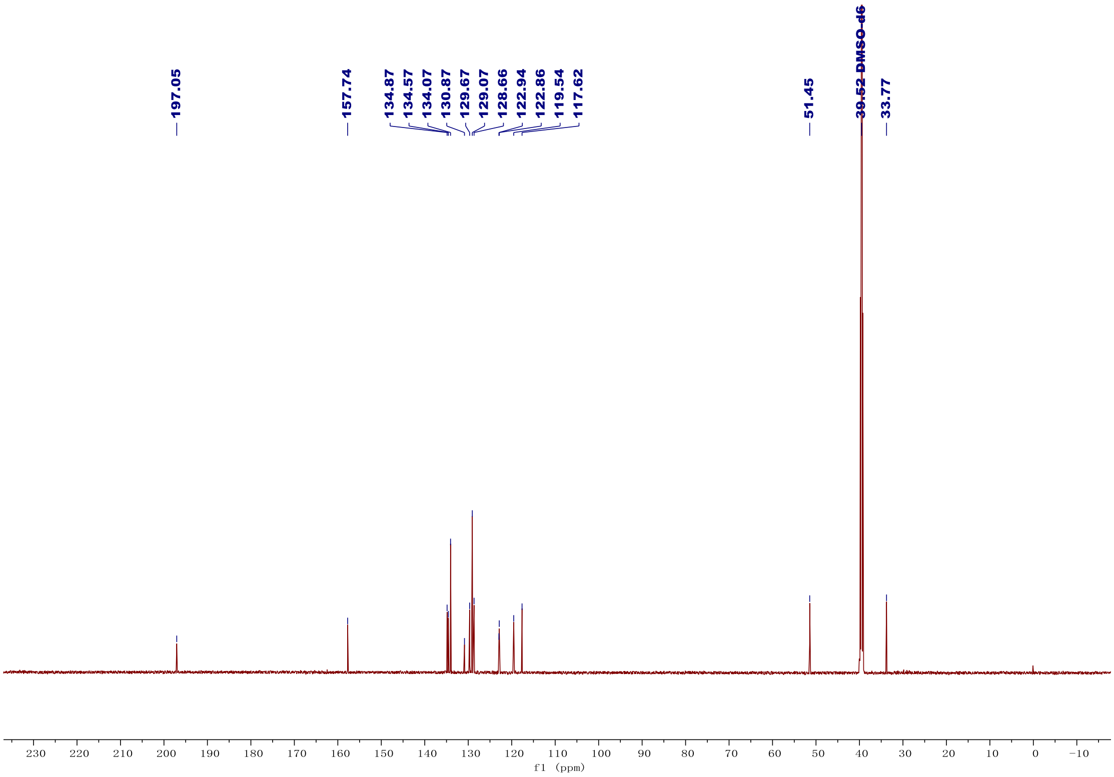

^{13}C NMR spectrum for product **3i**

1-(5-bromo-2-hydroxyphenyl)-2-(phenylthio)pent-4-en-1-one (3j)

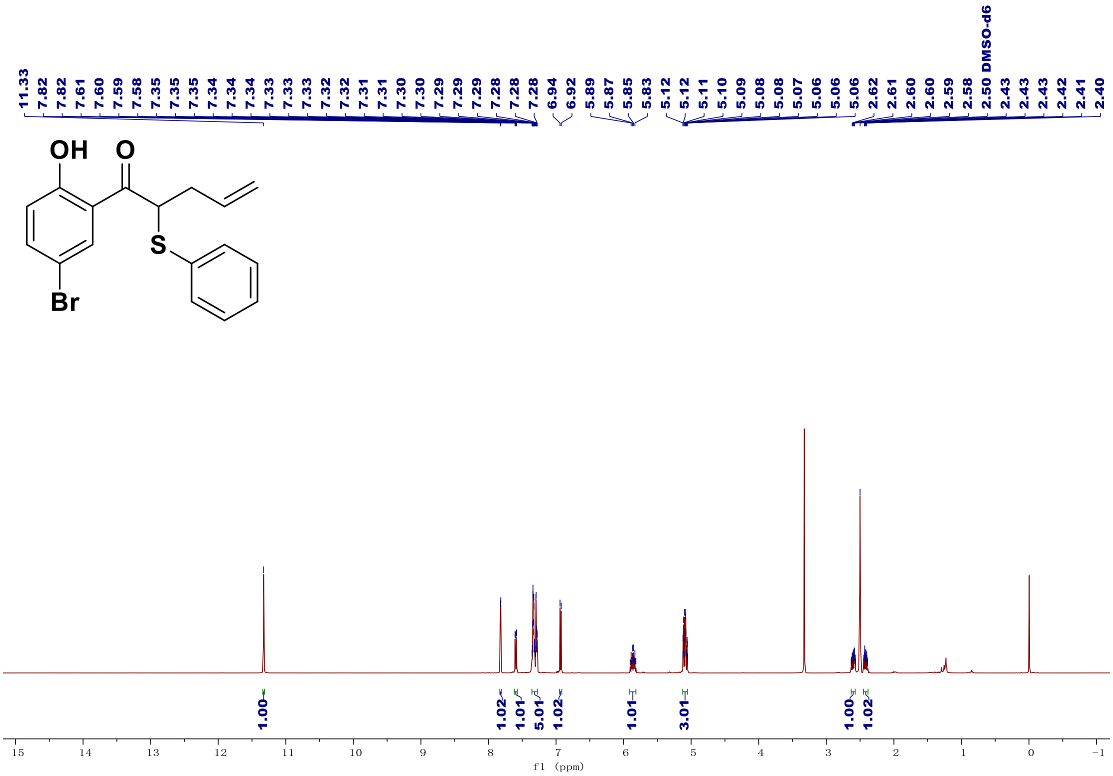

^{1}H NMR spectrum for product **3j**

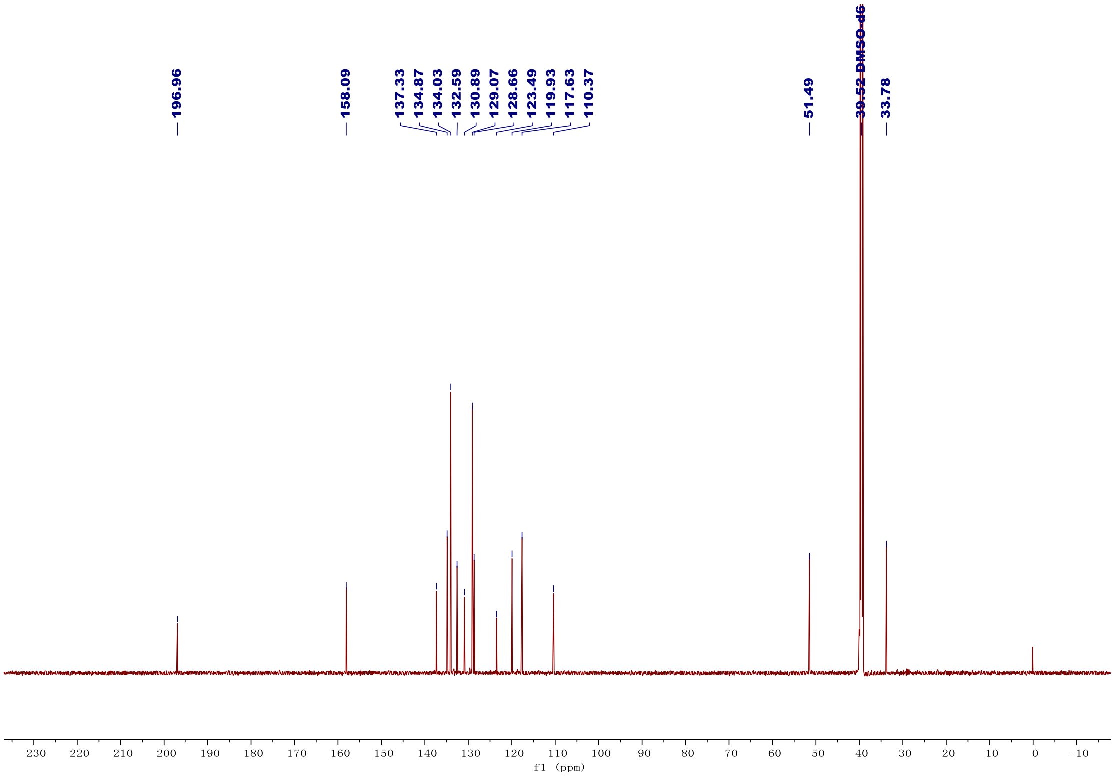

^{13}C NMR spectrum for product **3j**

1-(1-hydroxynaphthalen-2-yl)-2-(phenylthio)pent-4-en-1-one (3k)

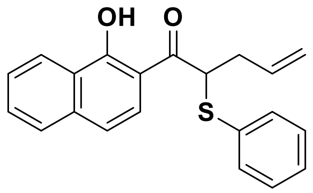

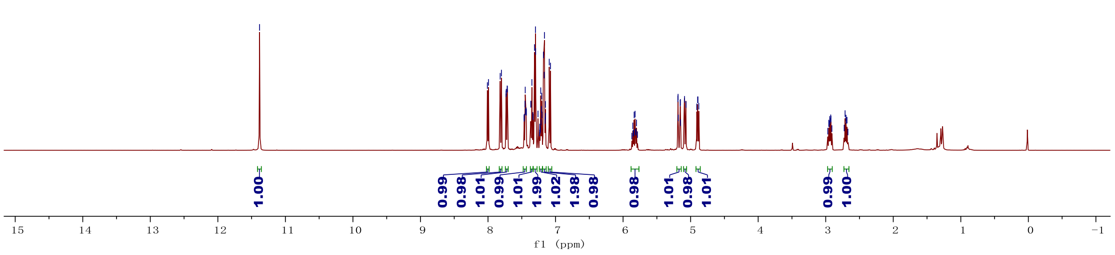

^{1}H NMR spectrum for product **3k**

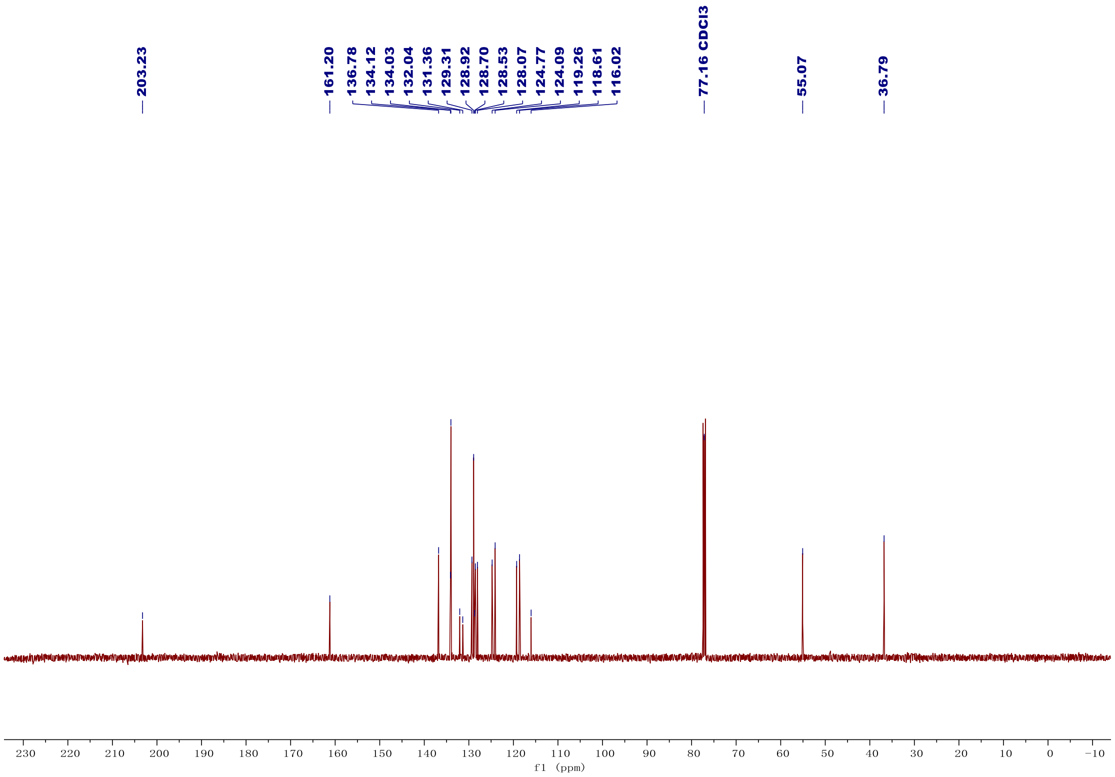

^{13}C NMR spectrum for product **3k**

1-(2-hydroxyphenyl)-2-((2-methoxyphenyl)thio)pent-4-en-1-one (3l)

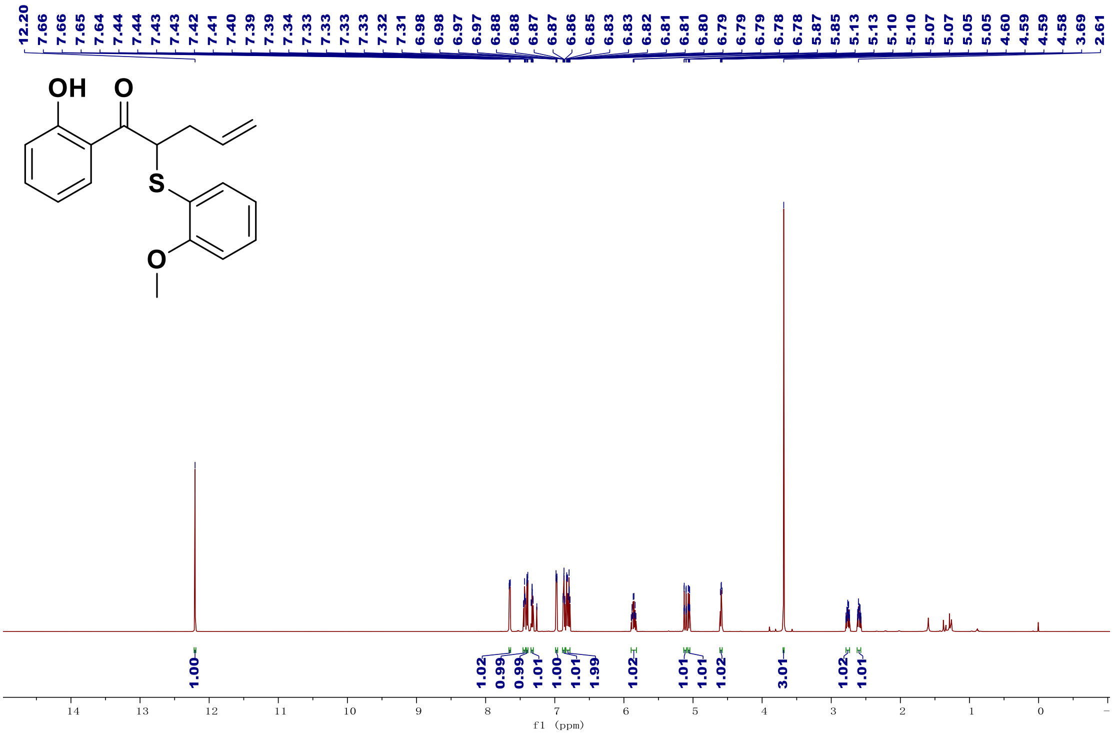

^{1}H NMR spectrum for product **3l**

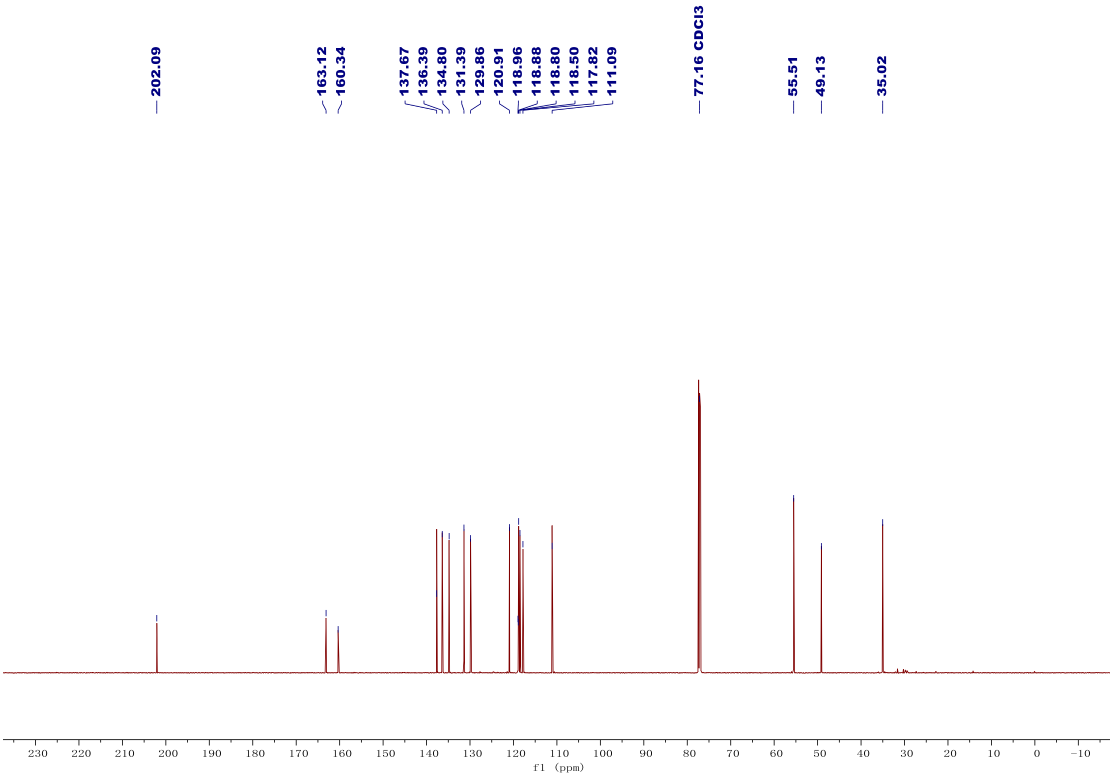

^{13}C NMR spectrum for product **3l**

2-((2-chlorophenyl)thio)-1-(2-hydroxyphenyl)pent-4-en-1-one (3m)

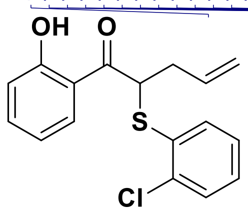

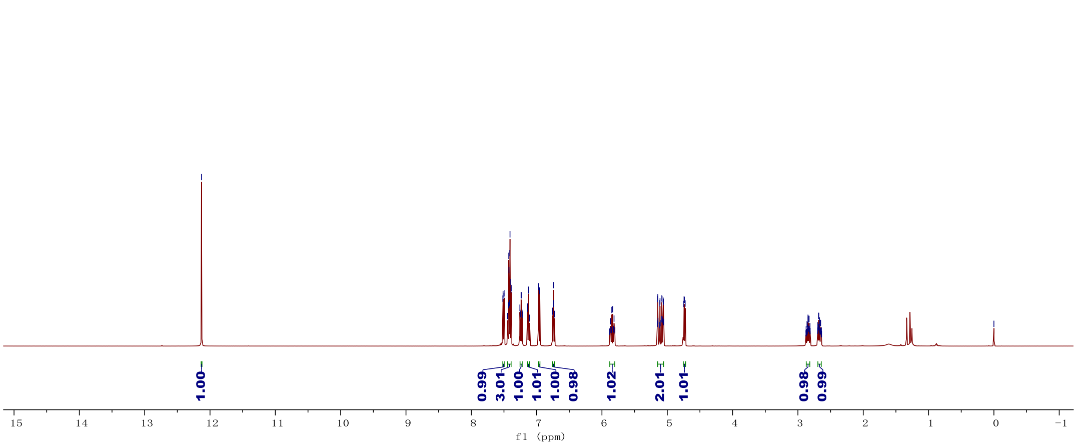

^{1}H NMR spectrum for product **3m**

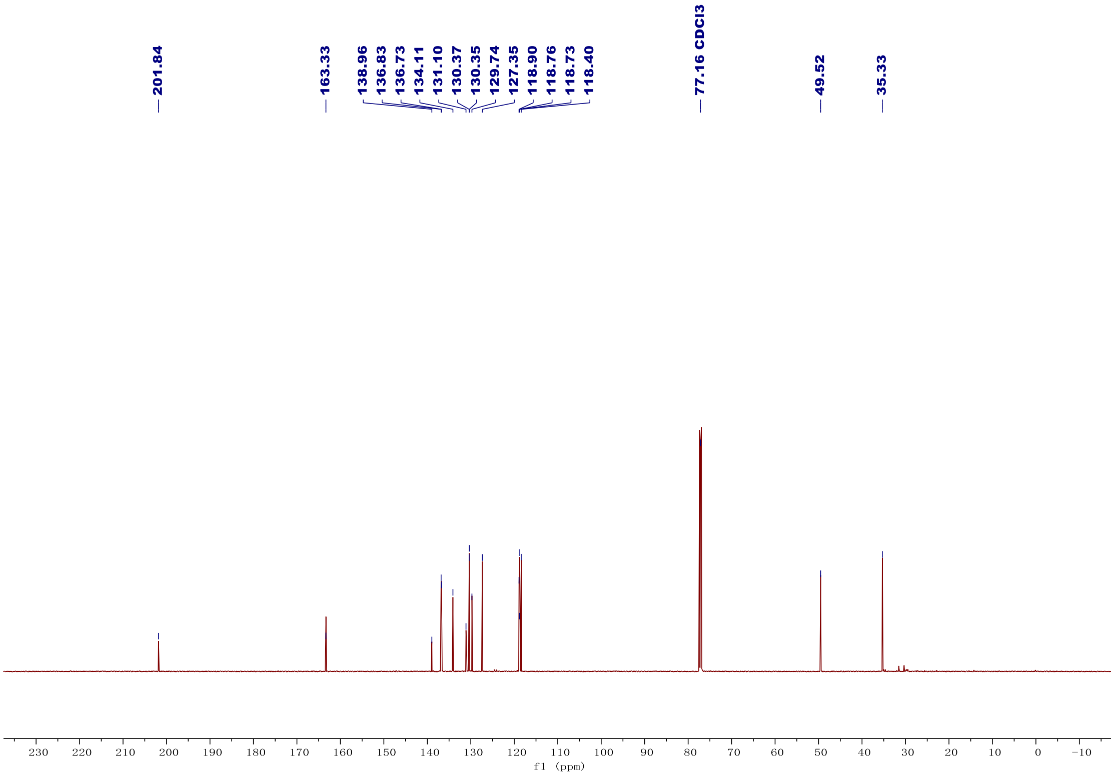

^{13}C NMR spectrum for product **3m**

1-(2-hydroxyphenyl)-2-(m-tolylthio)pent-4-en-1-one (3n)

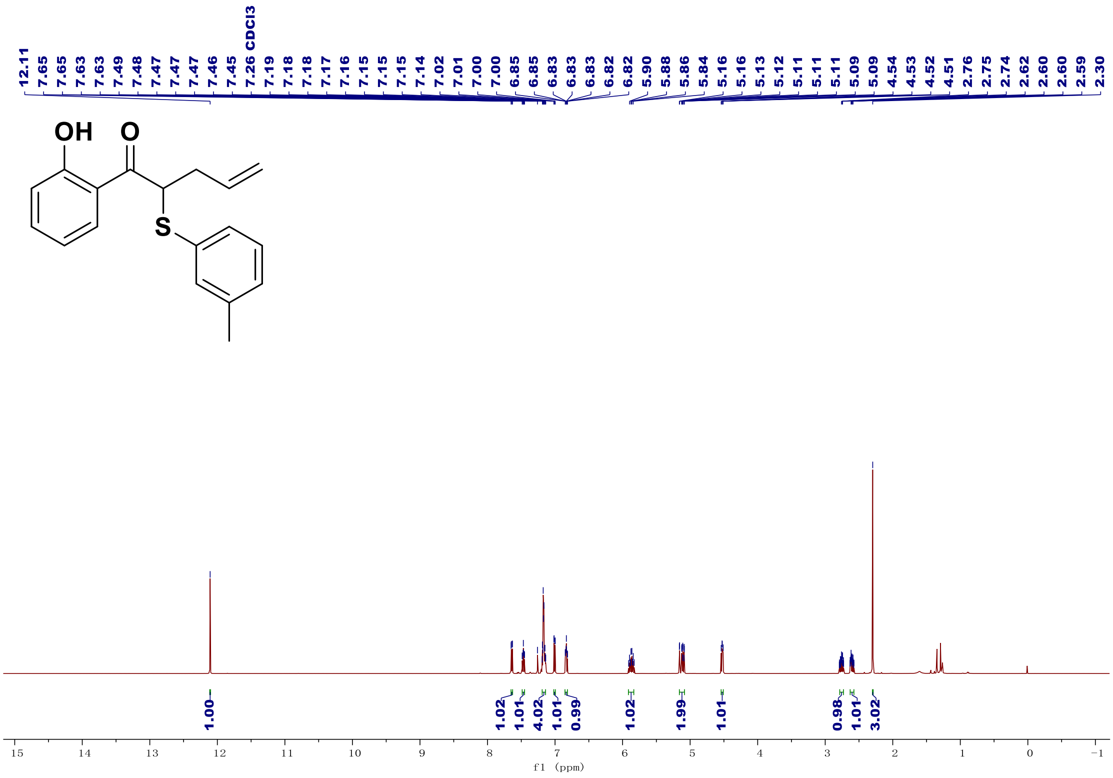

^{1}H NMR spectrum for product **3n**

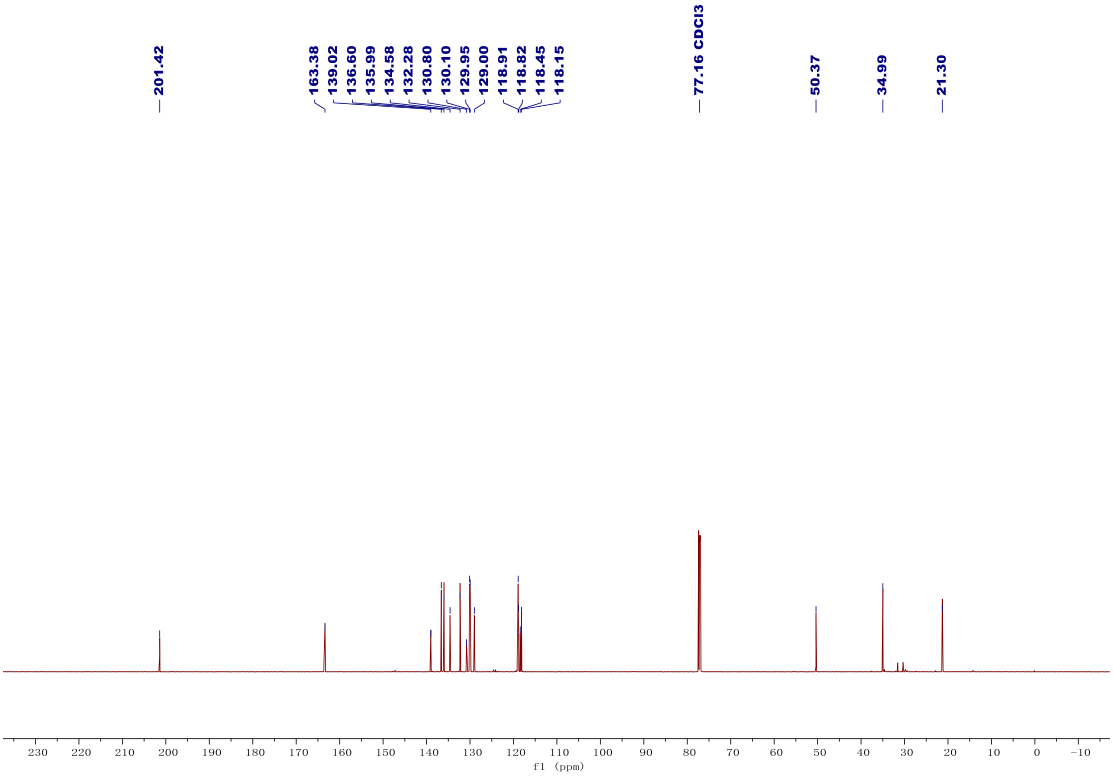

^{13}C NMR spectrum for product **3n**

2-((3-chlorophenyl)thio)-1-(2-hydroxyphenyl)pent-4-en-1-one (3o)

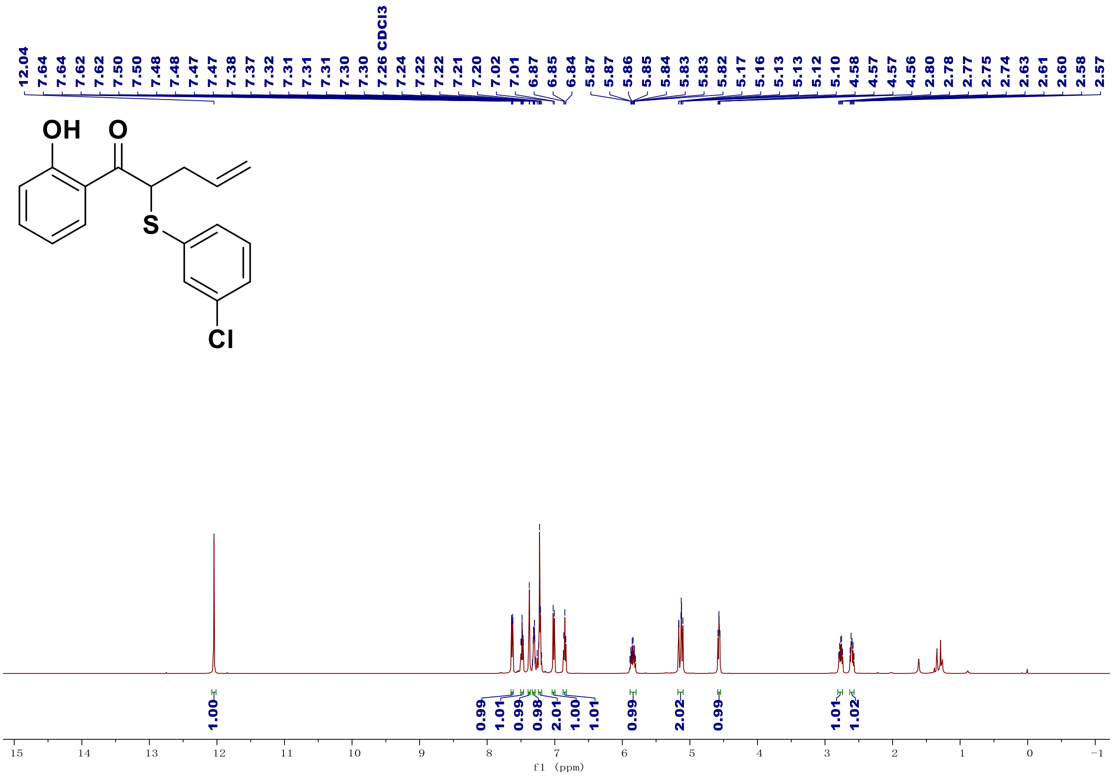

^{1}H NMR spectrum for product **3o**

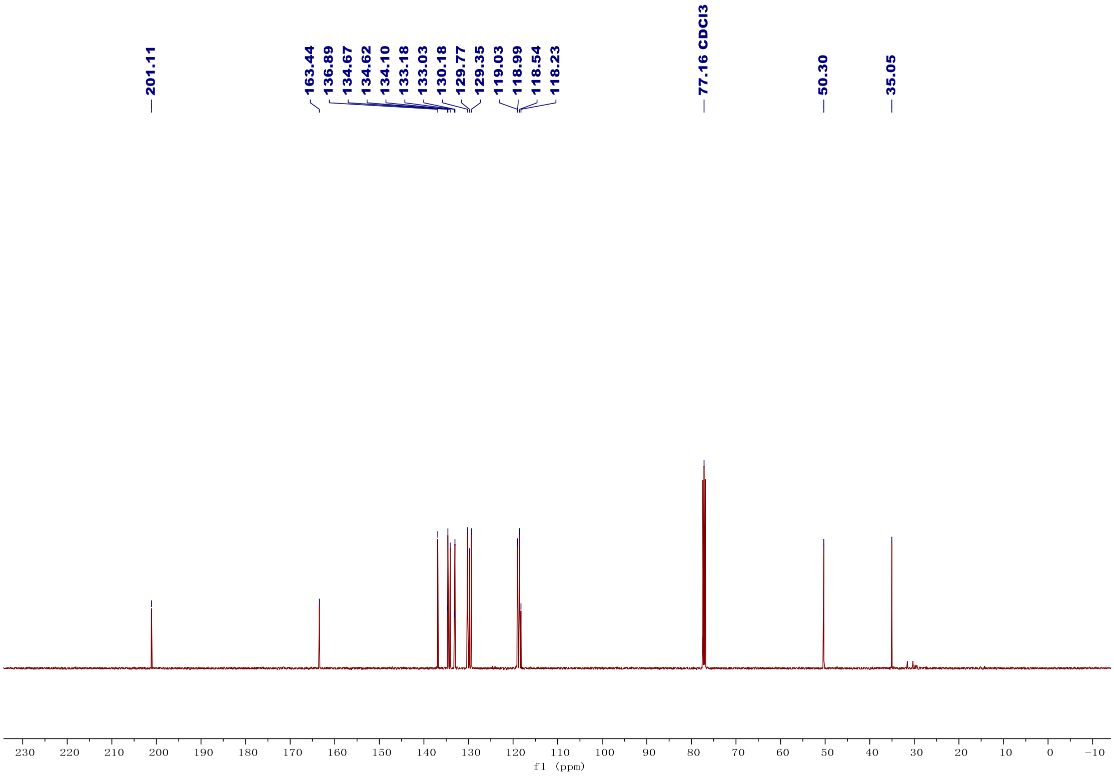

^{13}C NMR spectrum for product **3o**

1-(2-hydroxyphenyl)-2-((4-methoxyphenyl)thio)pent-4-en-1-one (3p)

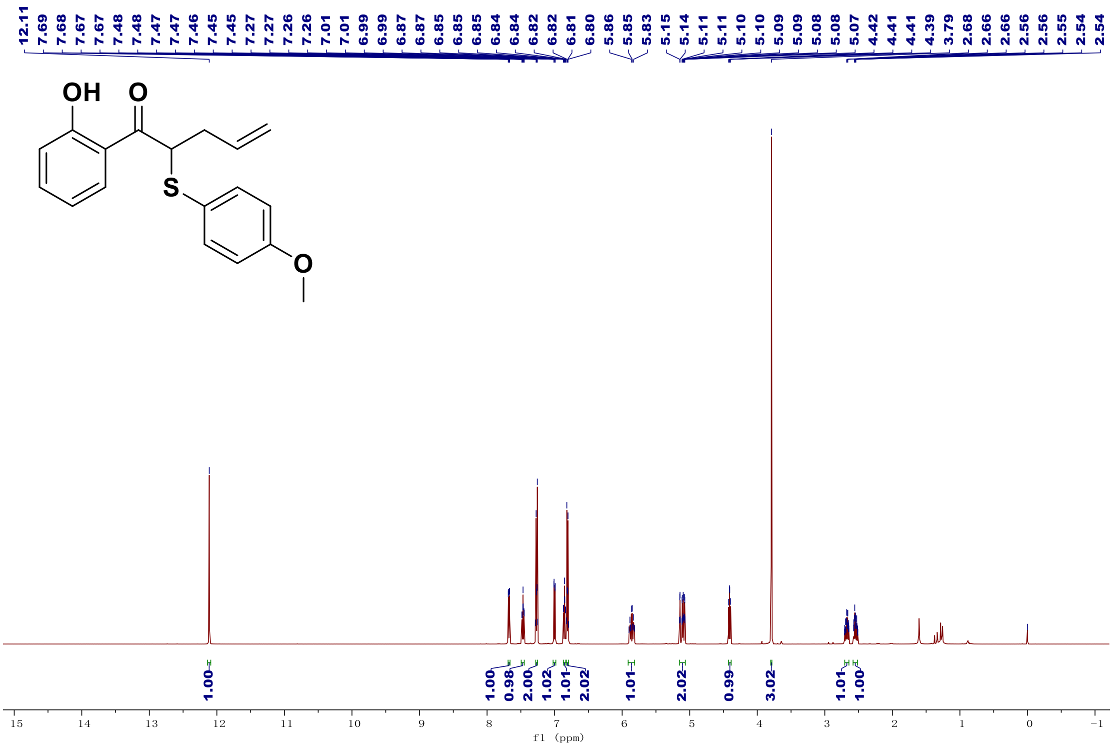

^{1}H NMR spectrum for product **3p**

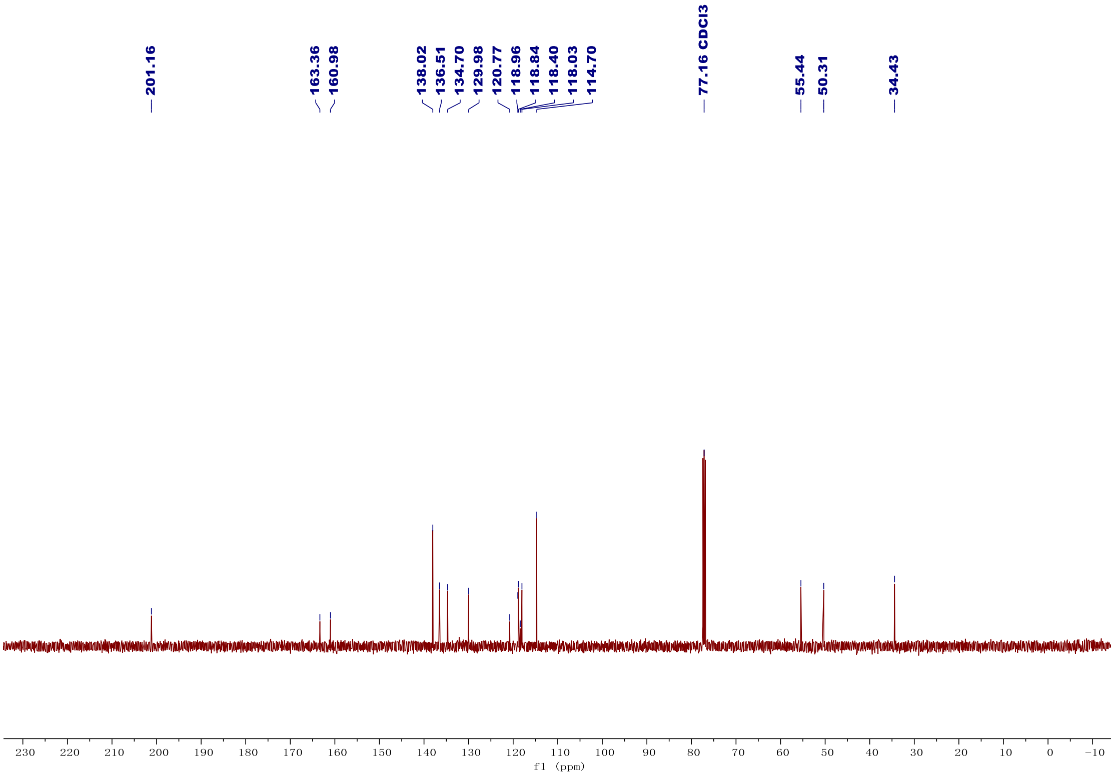

^{13}C NMR spectrum for product **3p**

1-(2-hydroxyphenyl)-2-((4-isopropylphenyl)thio)pent-4-en-1-one (3q)

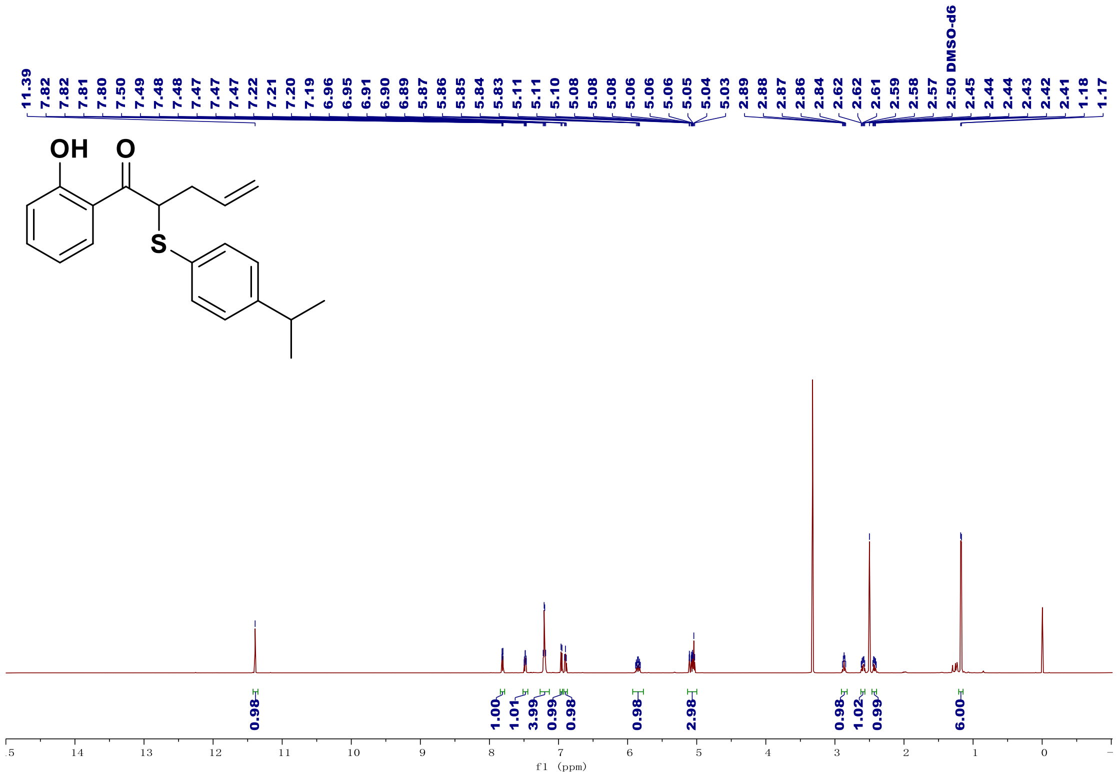

^{1}H NMR spectrum for product **3q**

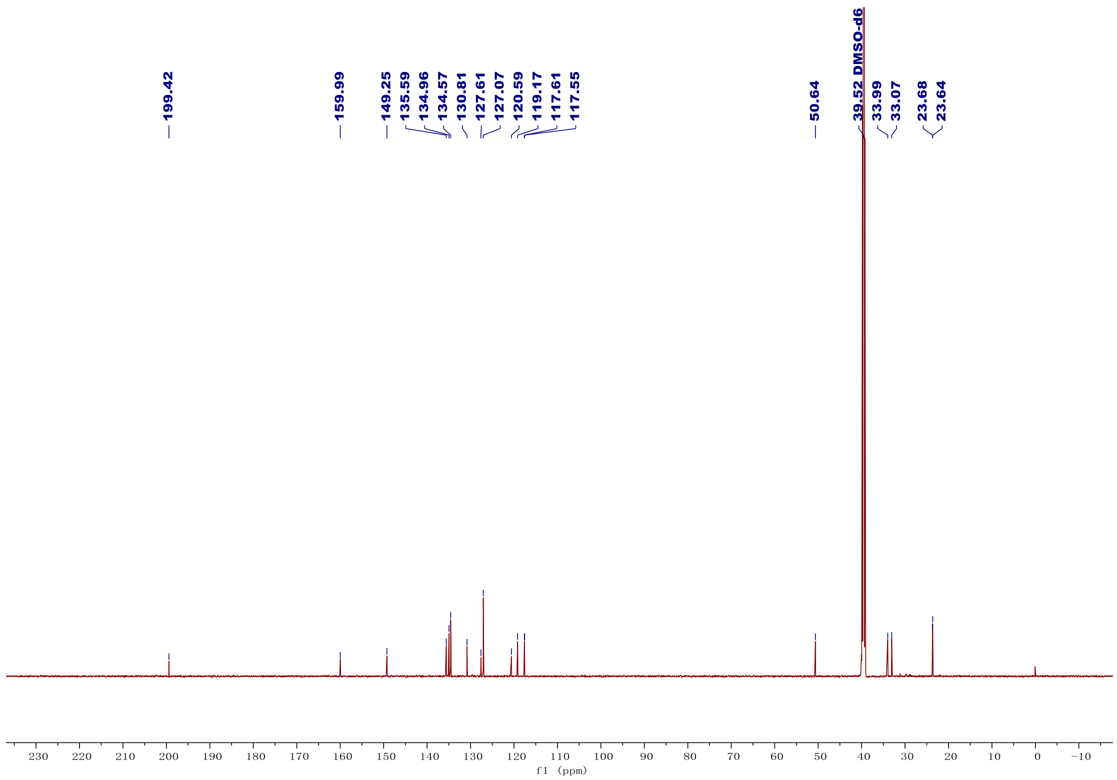

^{13}C NMR spectrum for product **3q**

2-((4-fluorophenyl)thio)-1-(2-hydroxyphenyl)pent-4-en-1-one (3r)

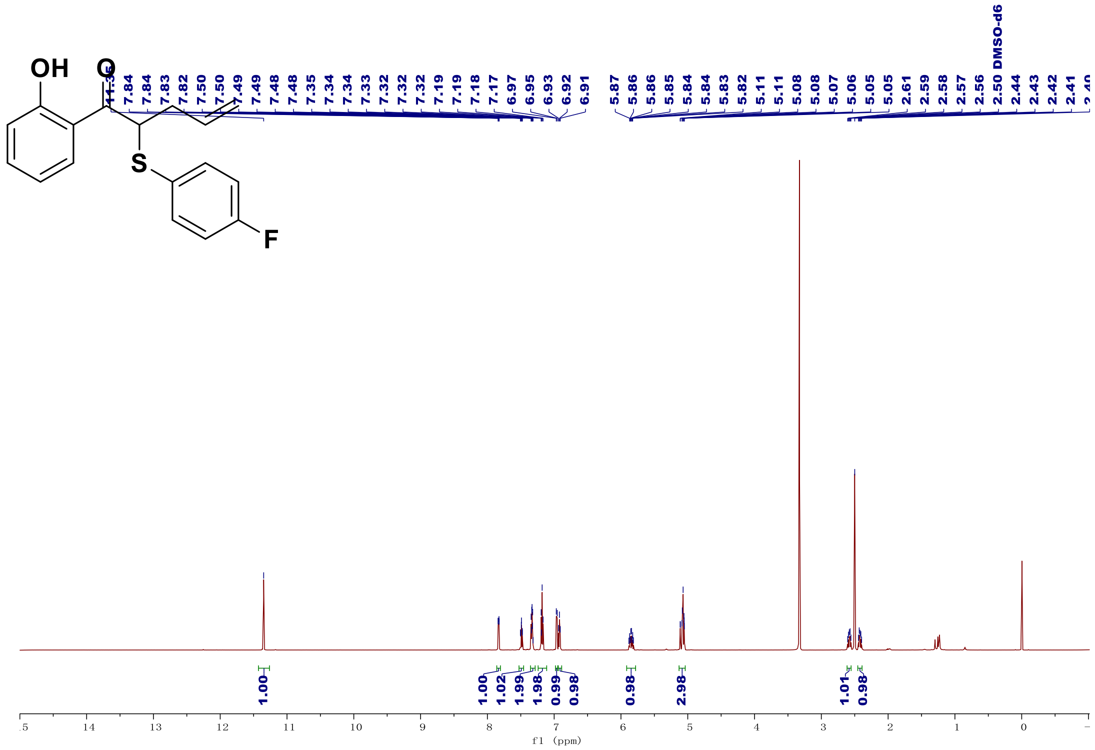

^{1}H NMR spectrum for product **3r**

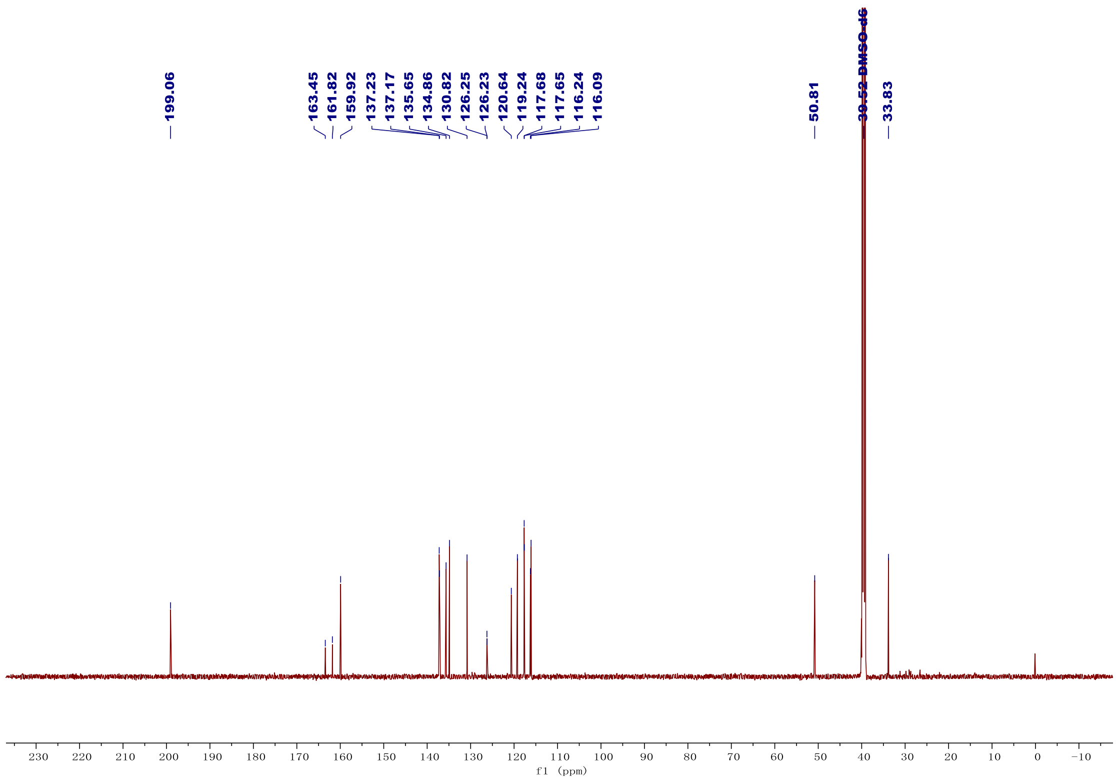

^{13}C NMR spectrum for product **3r**

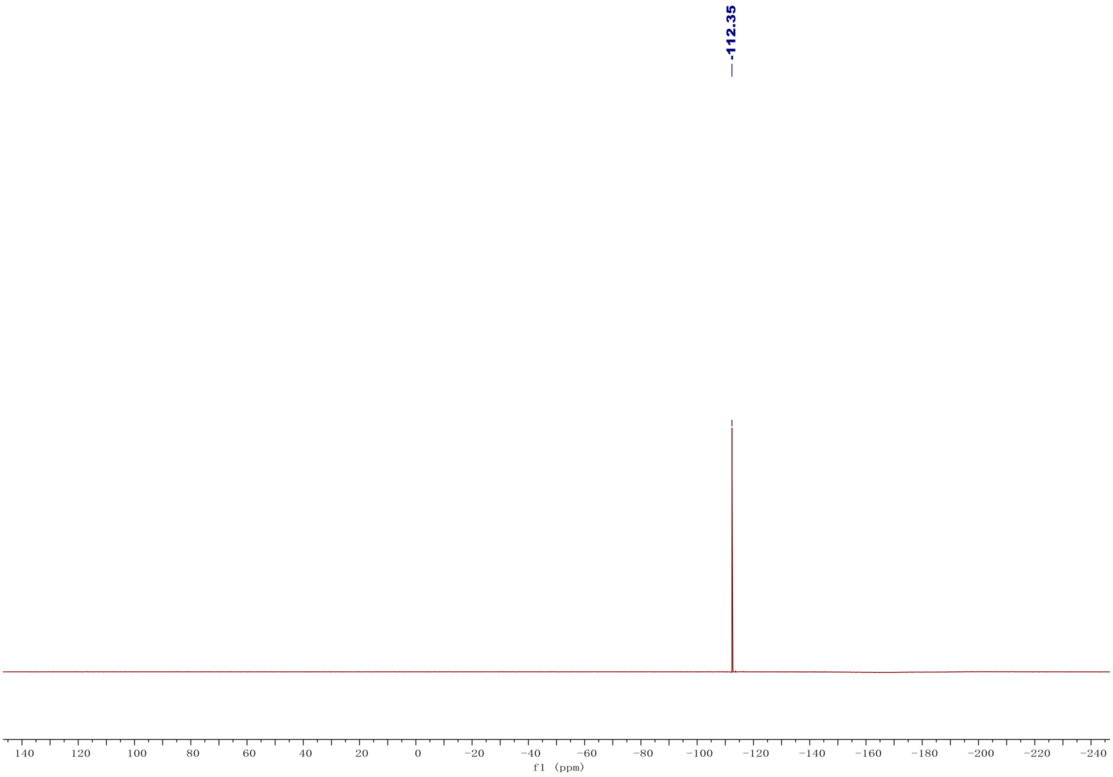

^{19}F NMR spectrum for product **3r**

2-((4-bromophenyl)thio)-1-(2-hydroxyphenyl)pent-4-en-1-one (3s)

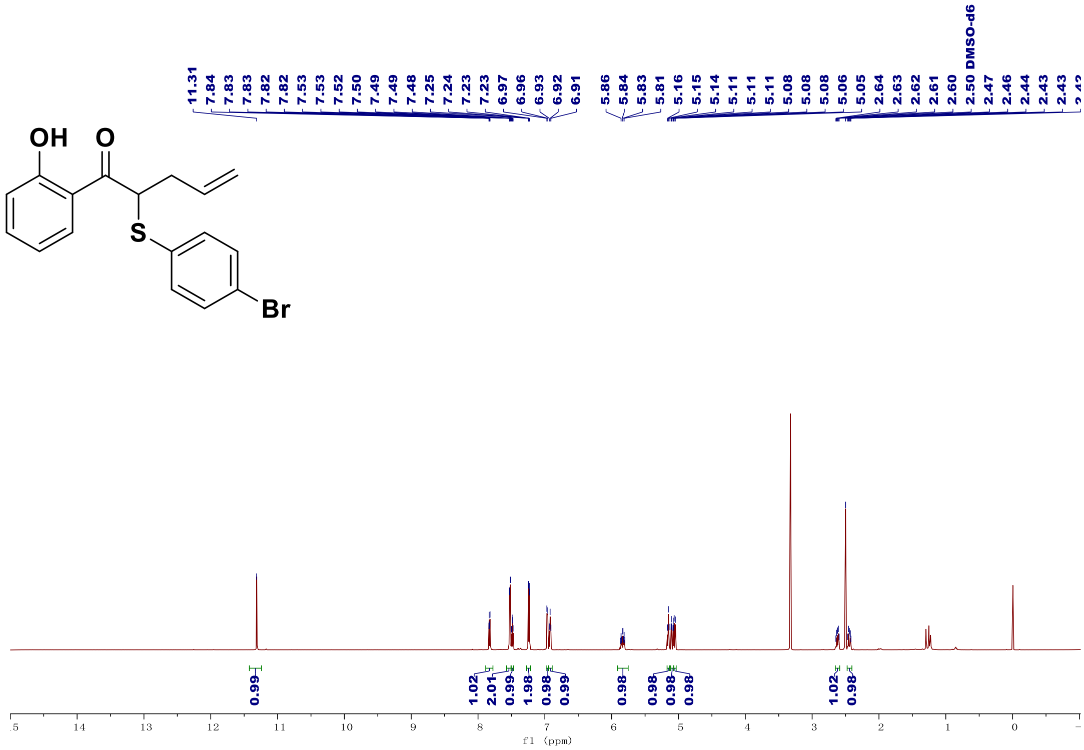

^{1}H NMR spectrum for product **3s**

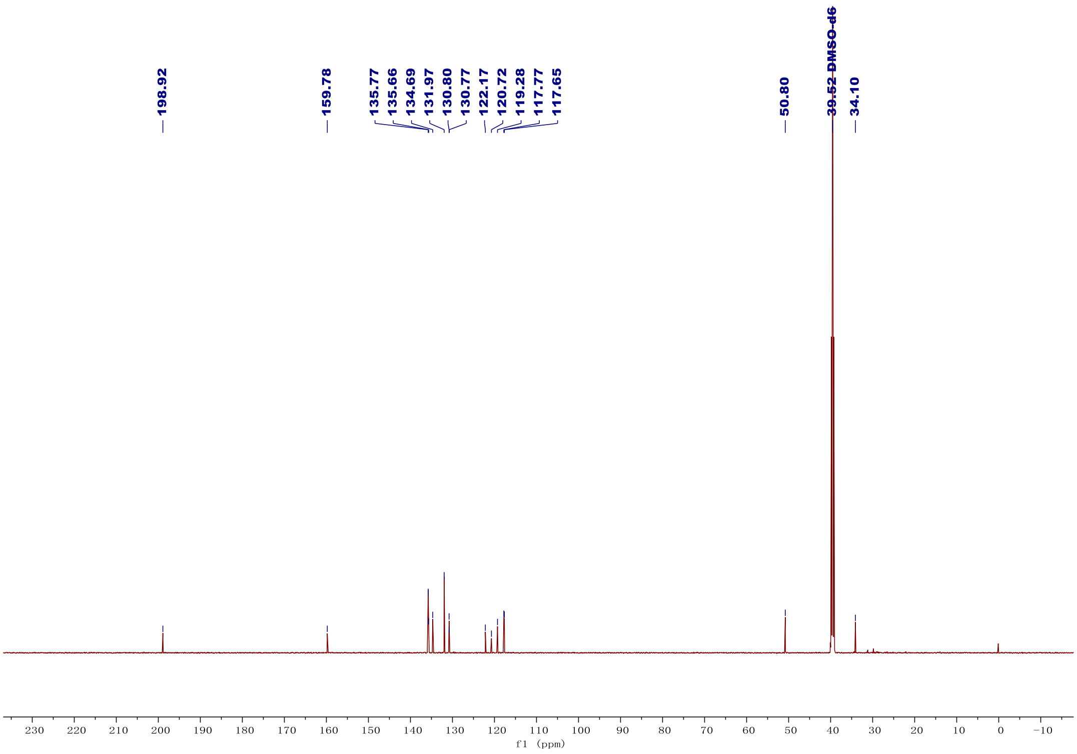

^{13}C NMR spectrum for product **3s**

1-(2-hydroxyphenyl)-2-((4-nitrophenyl)thio)pent-4-en-1-one (3t)

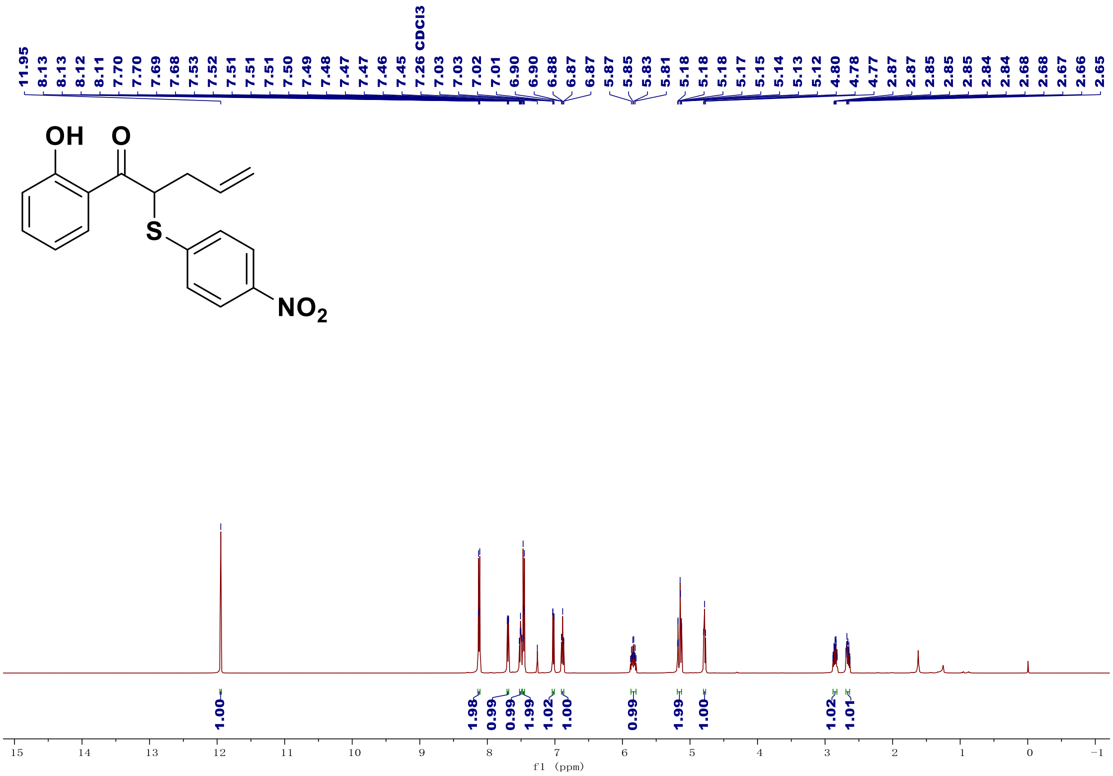

^{1}H NMR spectrum for product **3t**

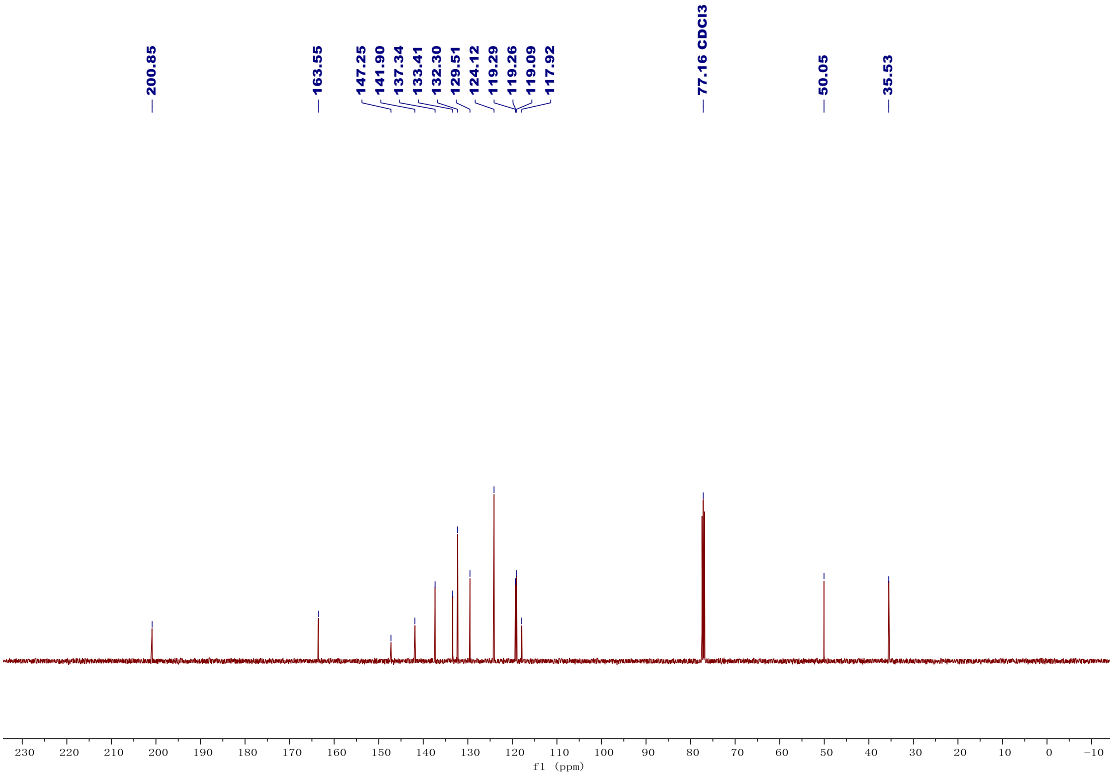

^{13}C NMR spectrum for product **3t**

1-(2-hydroxyphenyl)-2-(naphthalen-2-ylthio)pent-4-en-1-one (3u)

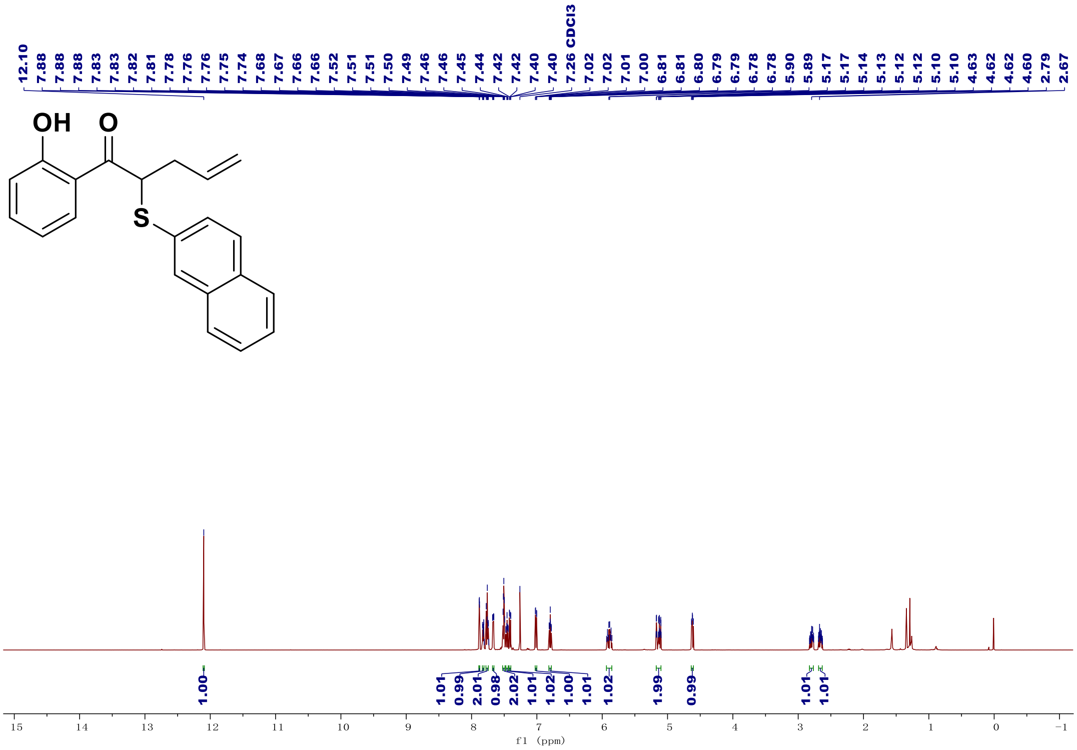

^{1}H NMR spectrum for product **3u**

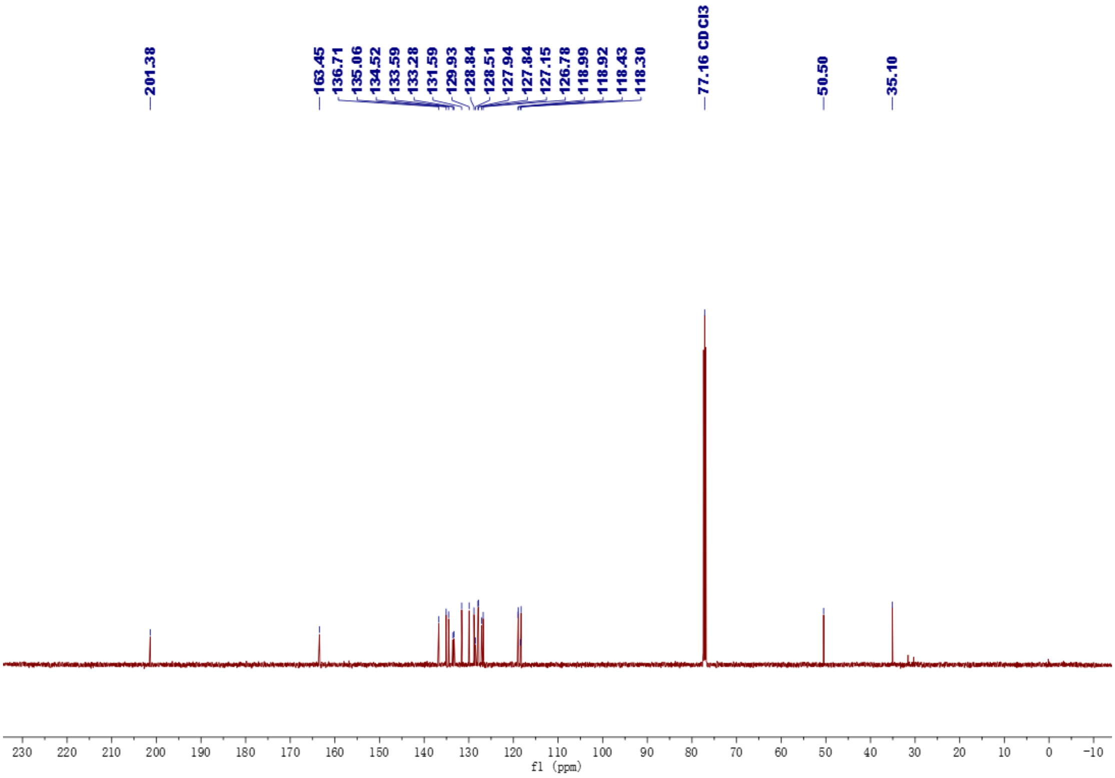

^{13}C NMR spectrum for product **3u**

1-(2-hydroxyphenyl)-2-(pyridin-2-ylthio)pent-4-en-1-one (3v)

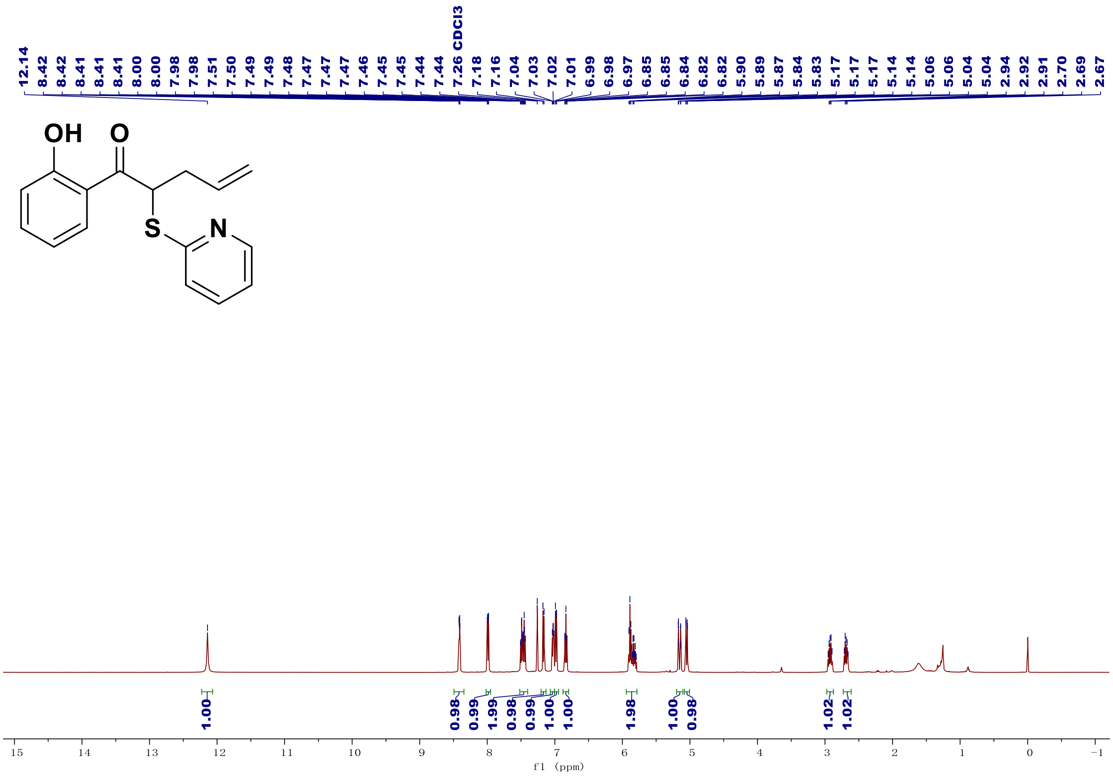

^{1}H NMR spectrum for product **3v**

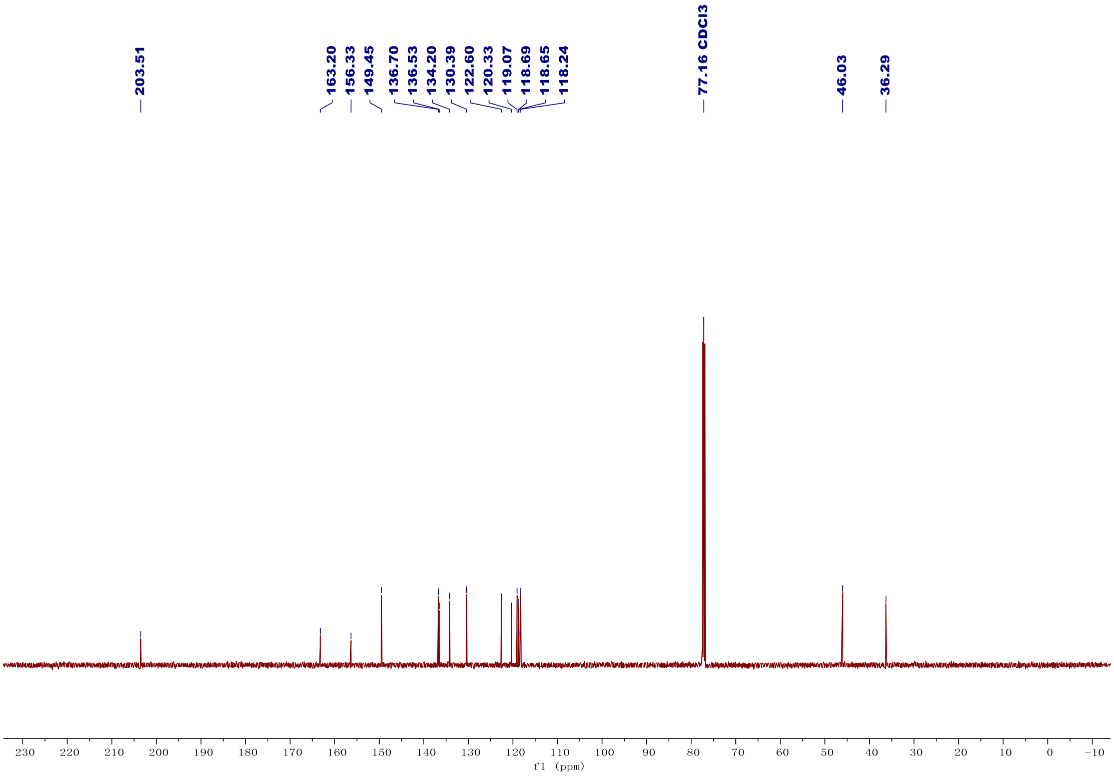

^{13}C NMR spectrum for product **3v**

2-(benzylthio)-1-(2-hydroxyphenyl)pent-4-en-1-one (3w)

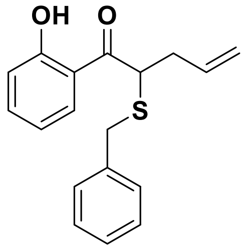

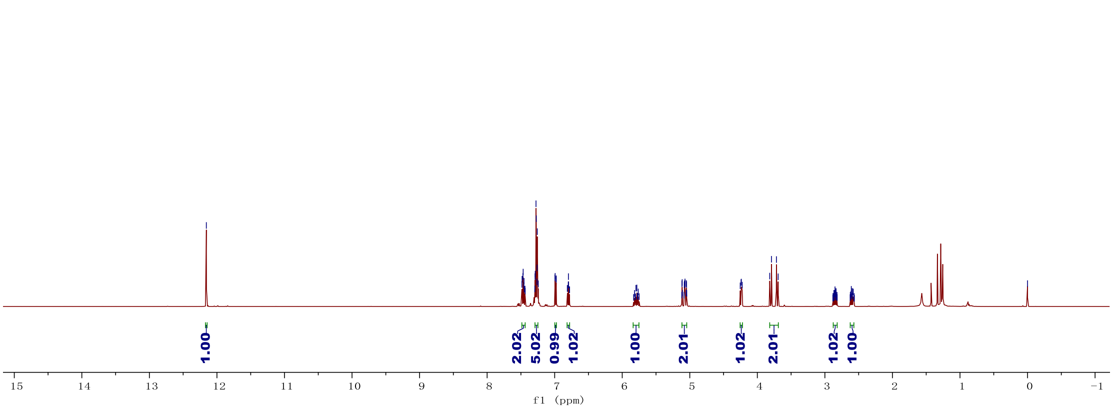

^{1}H NMR spectrum for product **3w**

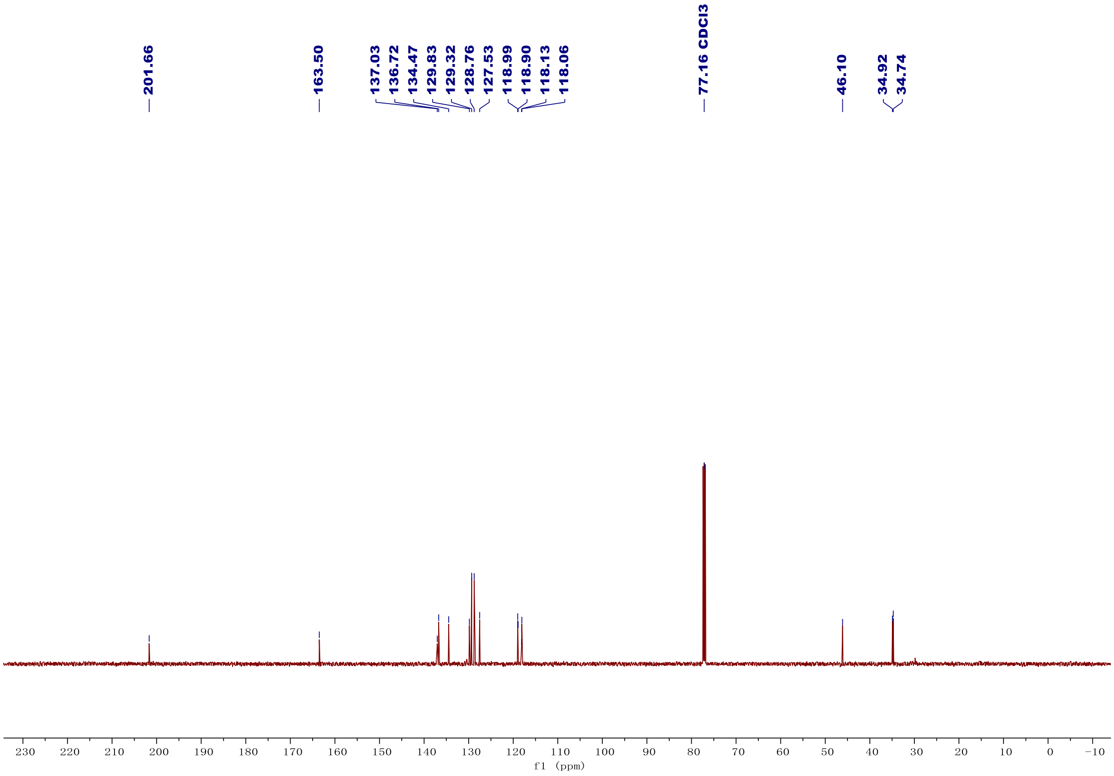

^{13}C NMR spectrum for product **3w**

2-(cyclohexylthio)-1-(2-hydroxyphenyl)pent-4-en-1-one (3x)

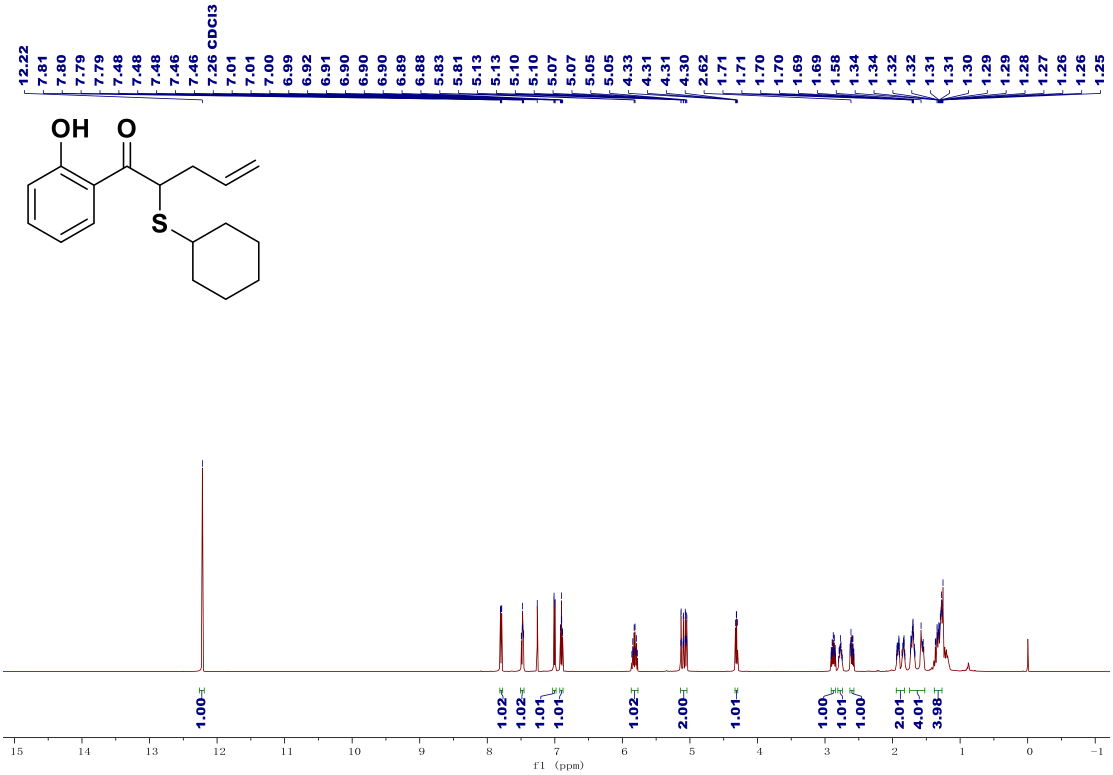

^{1}H NMR spectrum for product **3x**

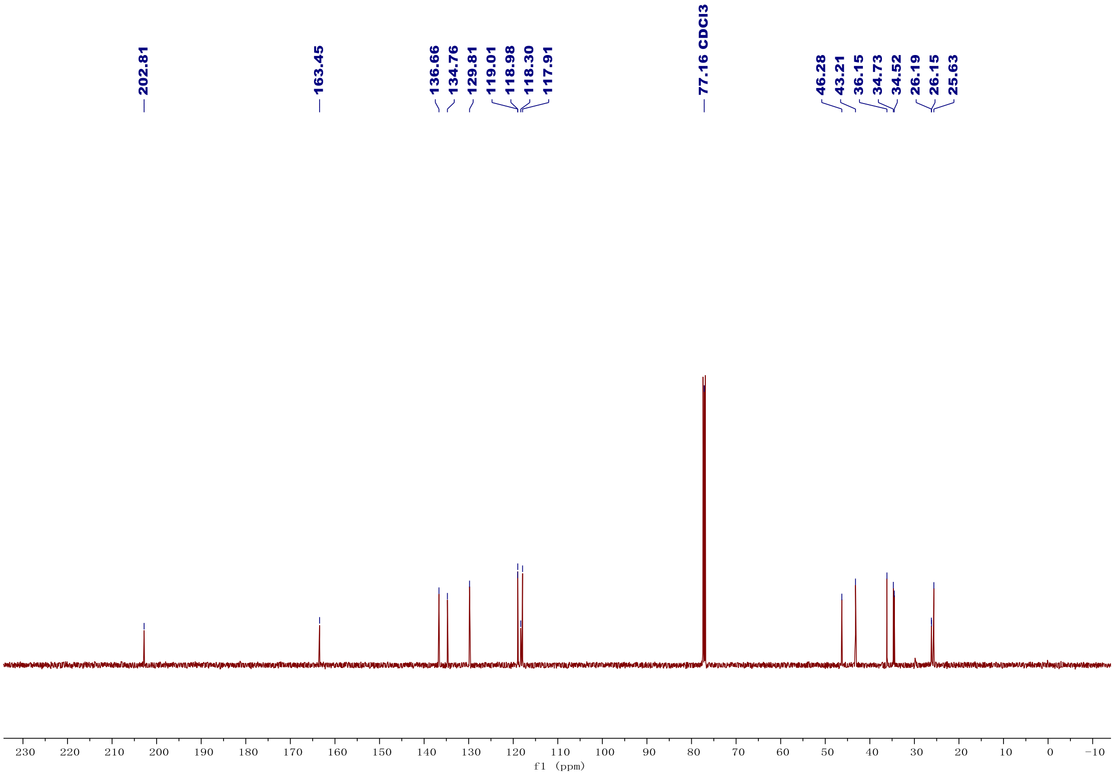

^{13}C NMR spectrum for product **3x**

2-(allylthio)-1-(2-hydroxyphenyl)pent-4-en-1-one (3y)

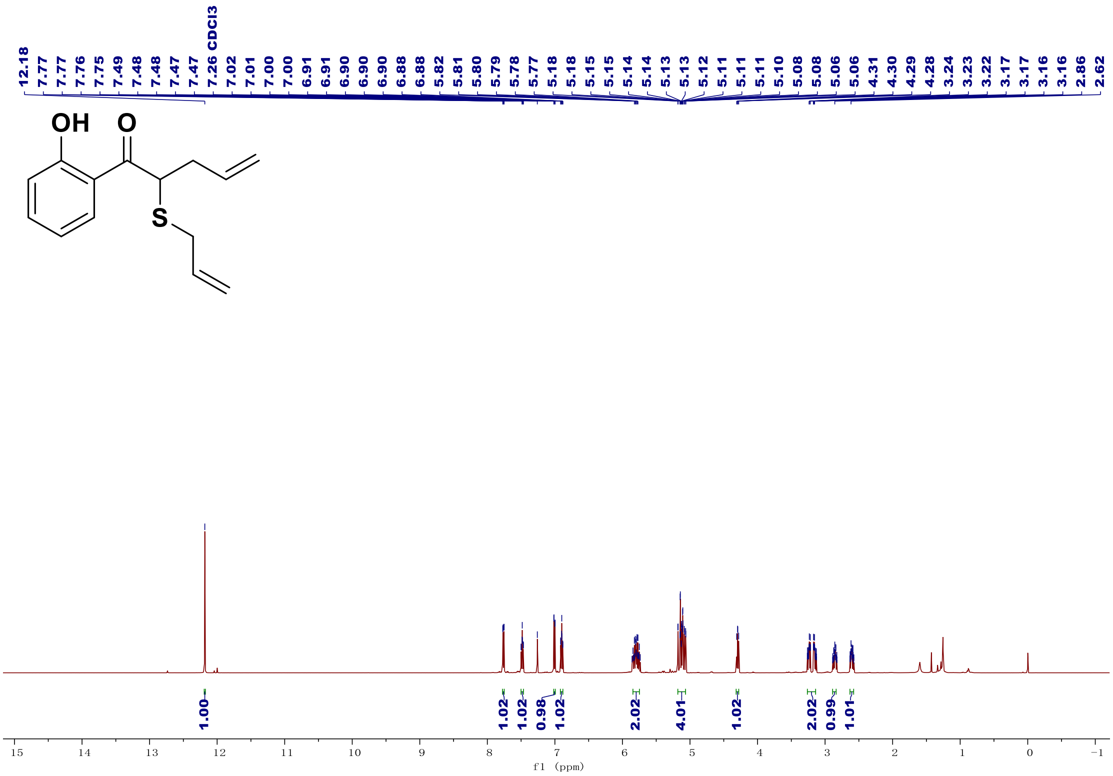

^{1}H NMR spectrum for product **3y**

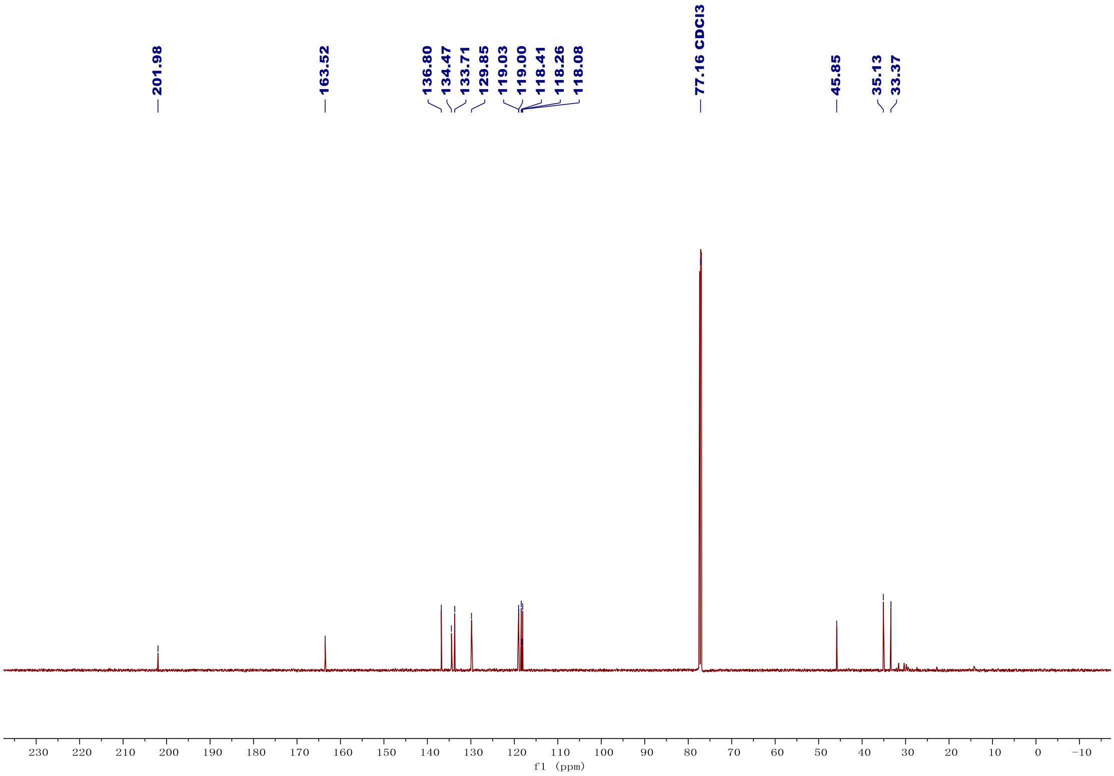

^{13}C NMR spectrum for product **3y**

1-(2-hydroxyphenyl)-2-(propylthio)pent-4-en-1-one (3z)

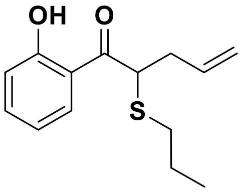

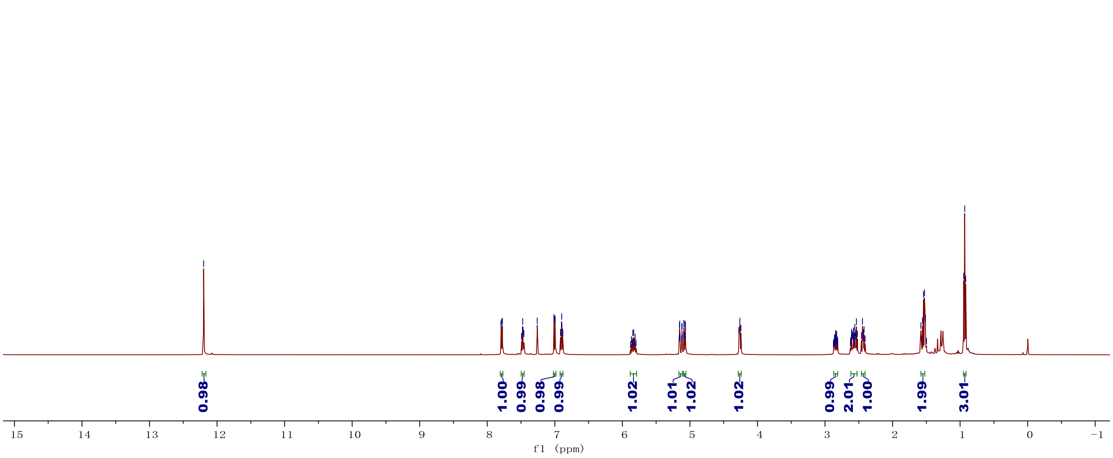

^{1}H NMR spectrum for product **3z**

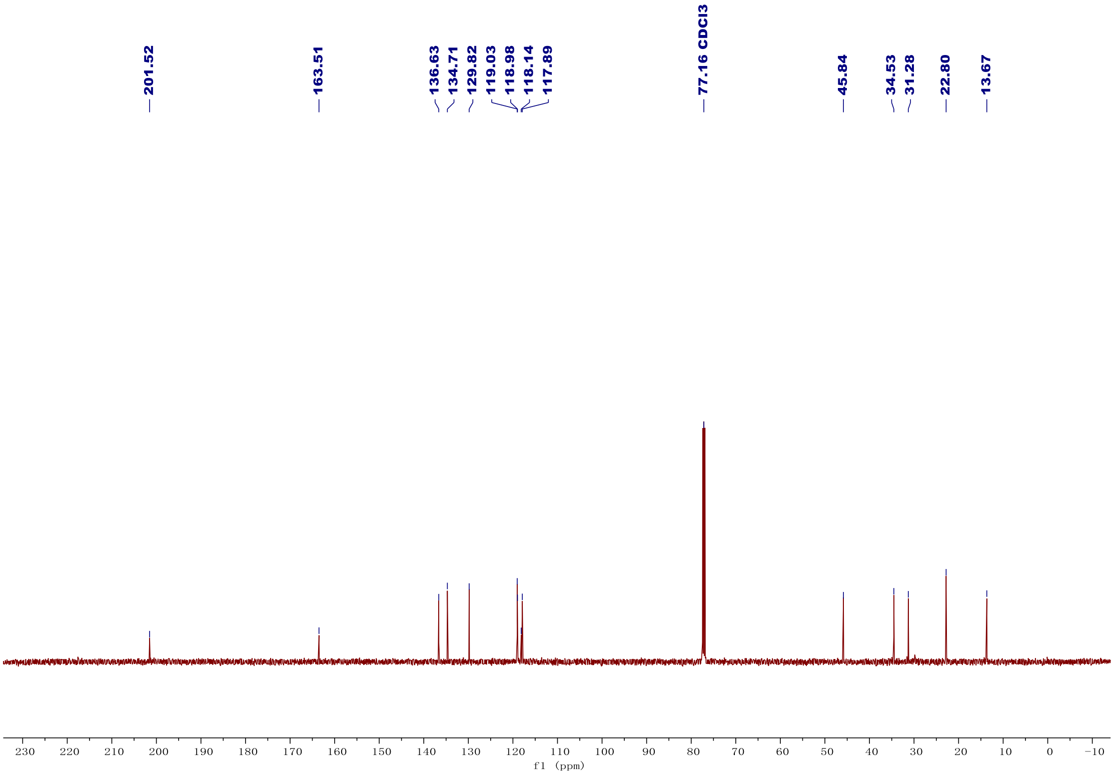

^{13}C NMR spectrum for product **3z**

1-(2-hydroxyphenyl)-2-(methylthio)pent-4-en-1-one (3za)

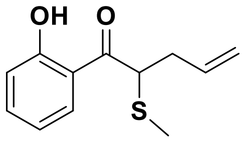

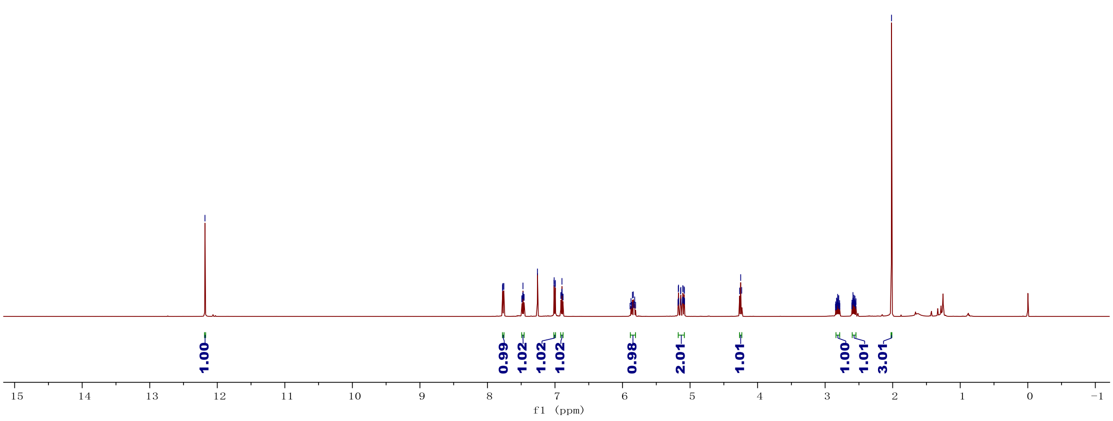

^{1}H NMR spectrum for product **3za**

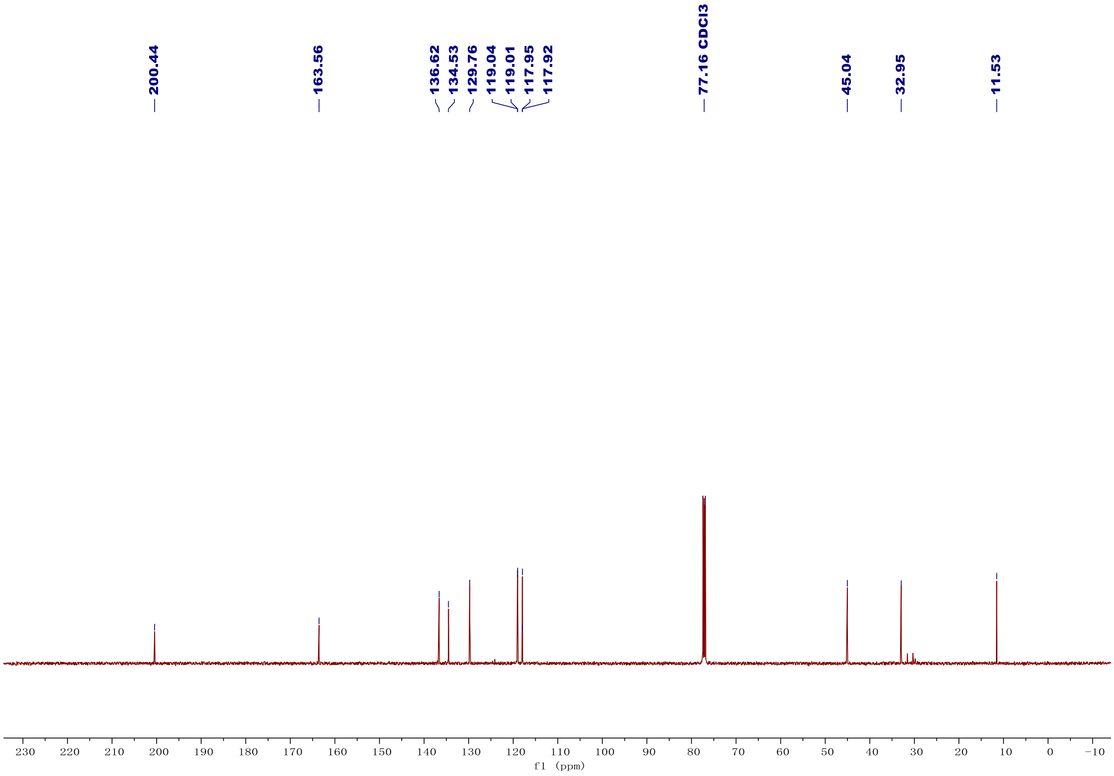

^{13}C NMR spectrum for product **3za**

2-((3-chlorophenyl)thio)-1-(2-hydroxyphenyl)-3-methylpent-4-en-1-one (3zb)

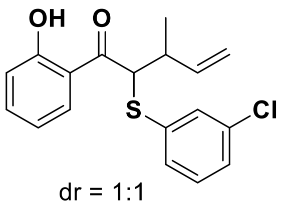

dr = 1:1

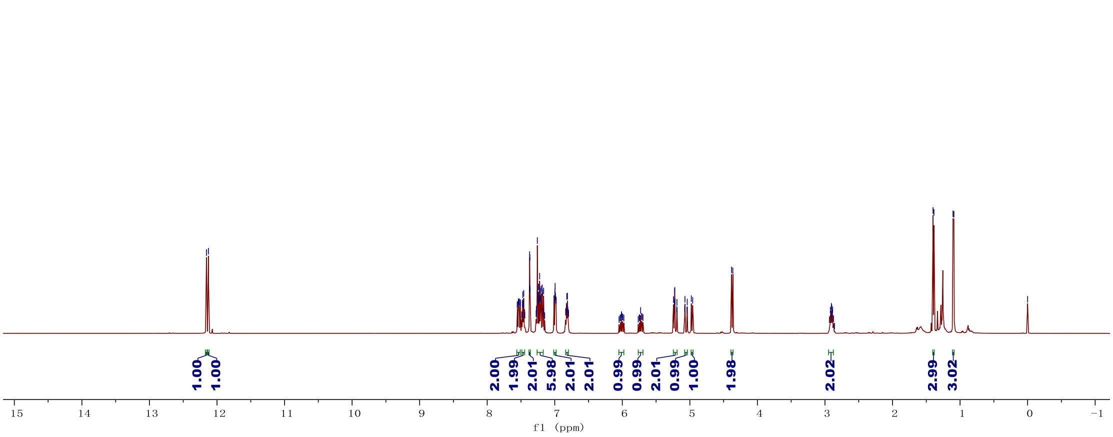

^{1}H NMR spectrum for product **3zb**

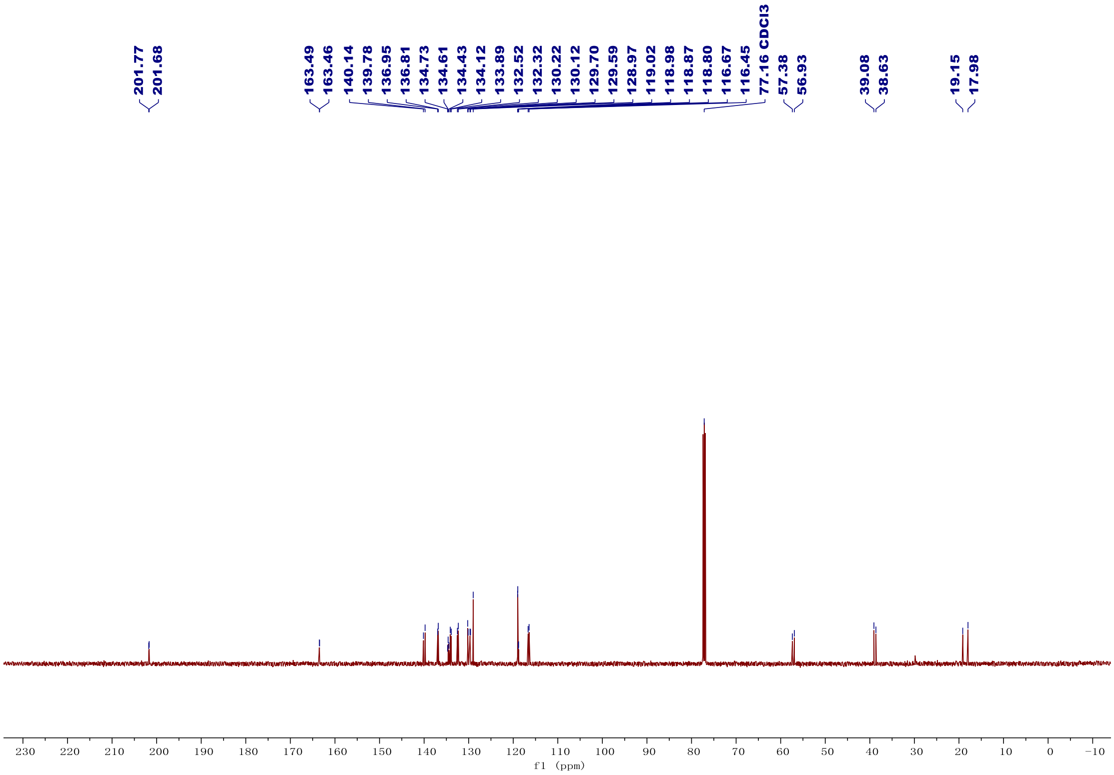

^{13}C NMR spectrum for product **3zb**

3-allyl-3-(phenylthio)quinoline-2,4(1H,3H)-dione (5a)

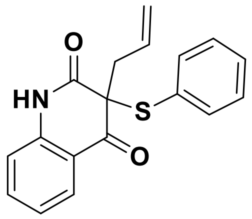

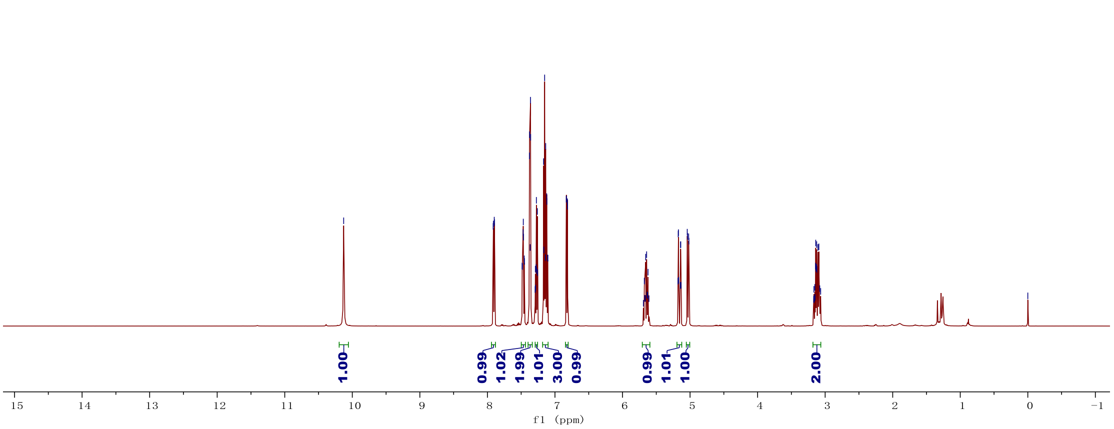

^{1}H NMR spectrum for product **5a**

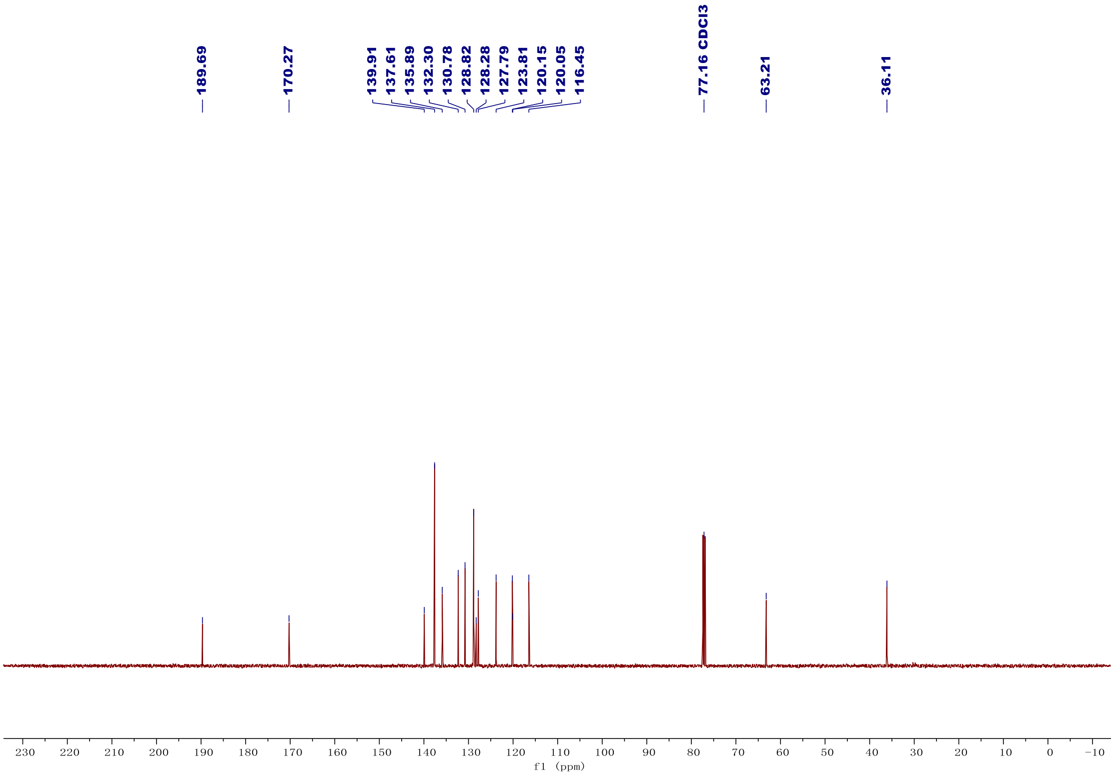

^{13}C NMR spectrum for product **5a**

methyl 2-((3-allyl-2,4-dioxo-1,2,3,4-tetrahydroquinolin-3-yl)thio)benzoate (5b)

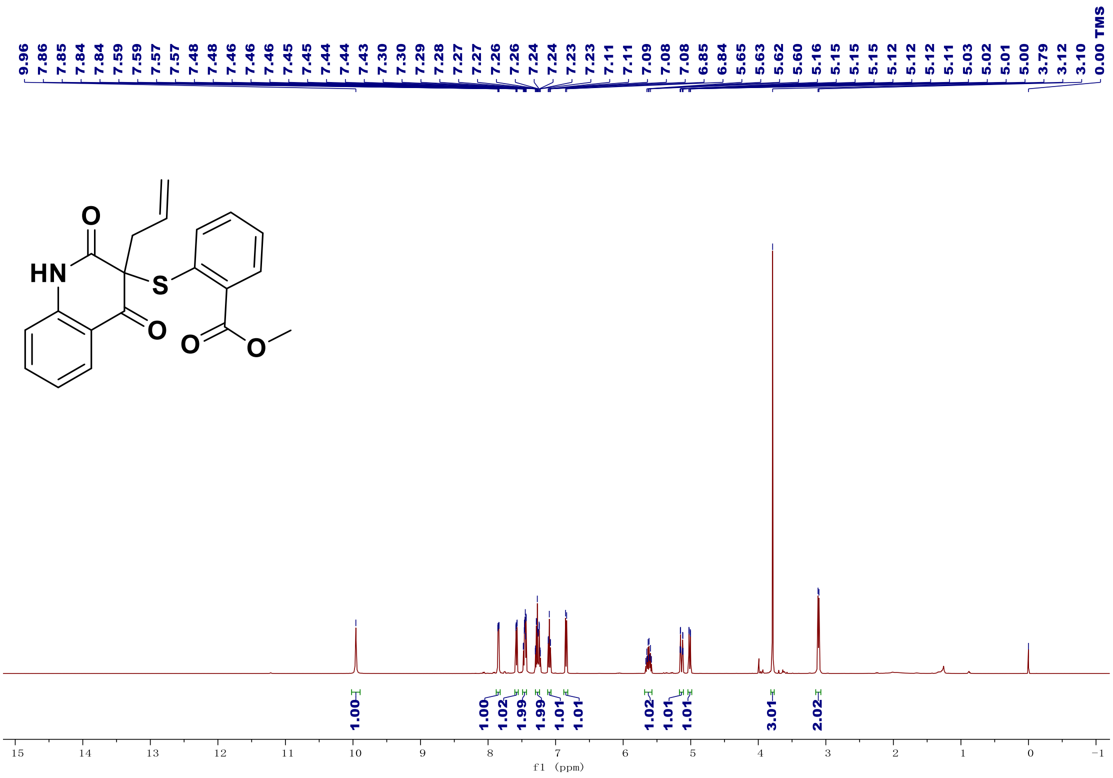

^{1}H NMR spectrum for product **5b**

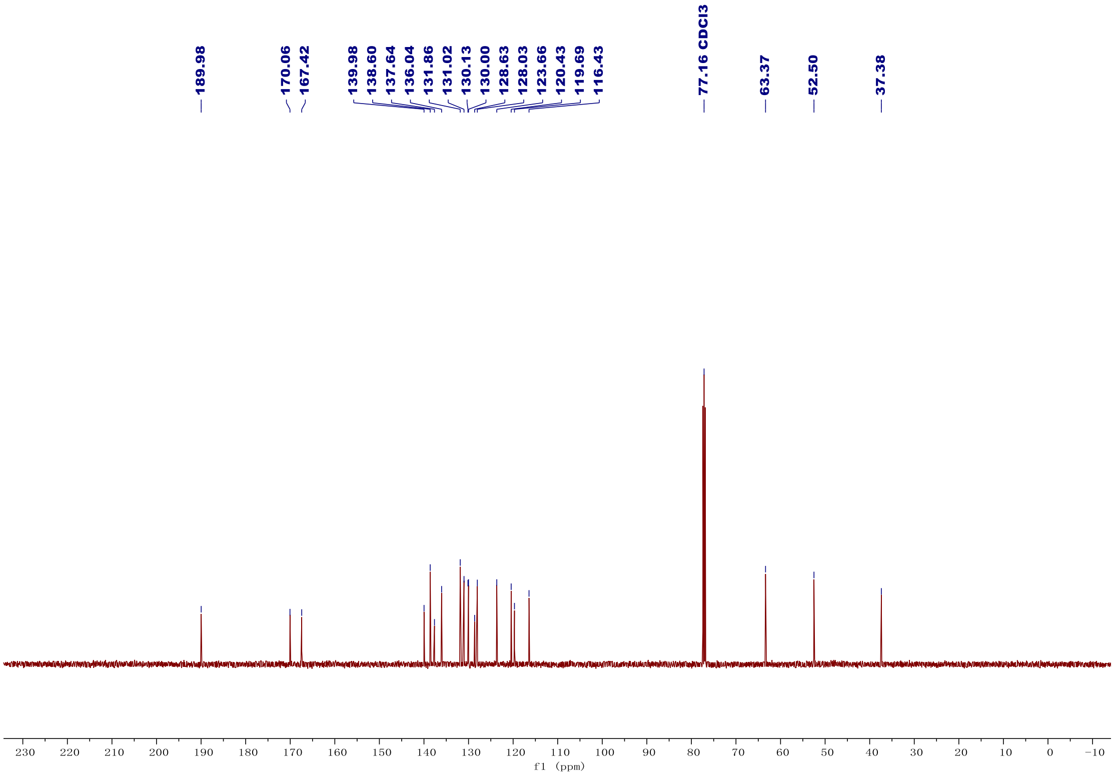

^{13}C NMR spectrum for product **5b**

3-allyl-3-((4-(tert-butyl)phenyl)thio)quinoline-2,4(1H,3H)-dione (5c)

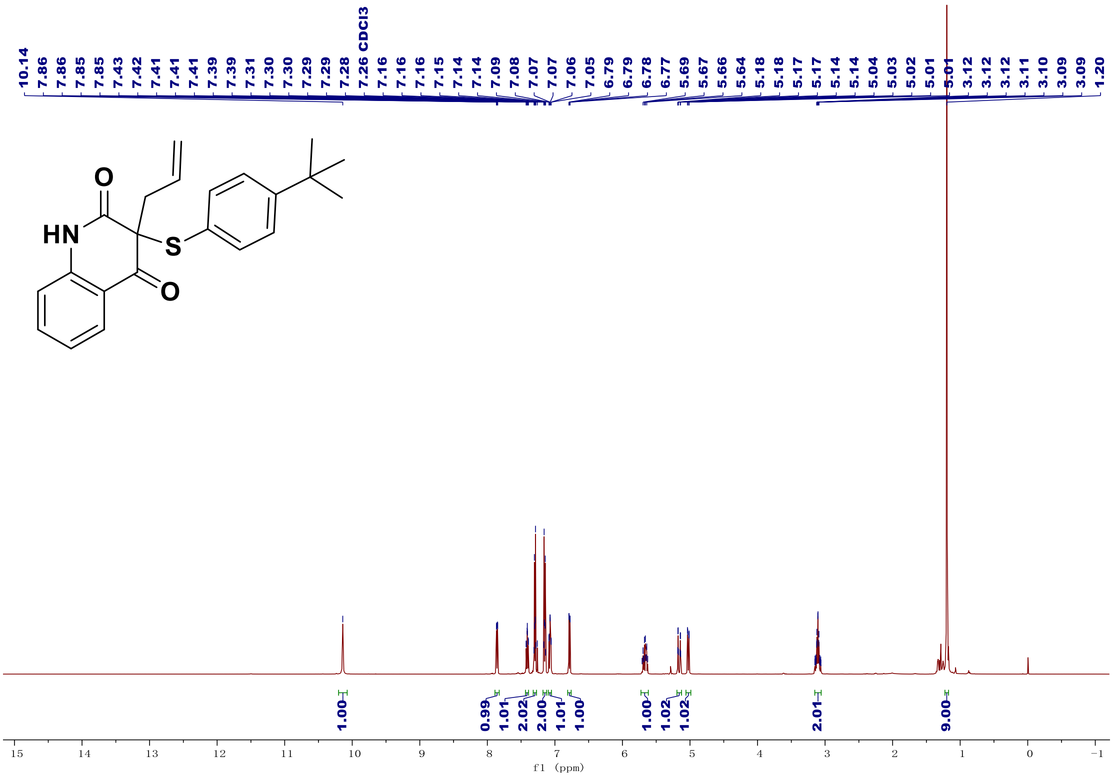

^{1}H NMR spectrum for product **5c**

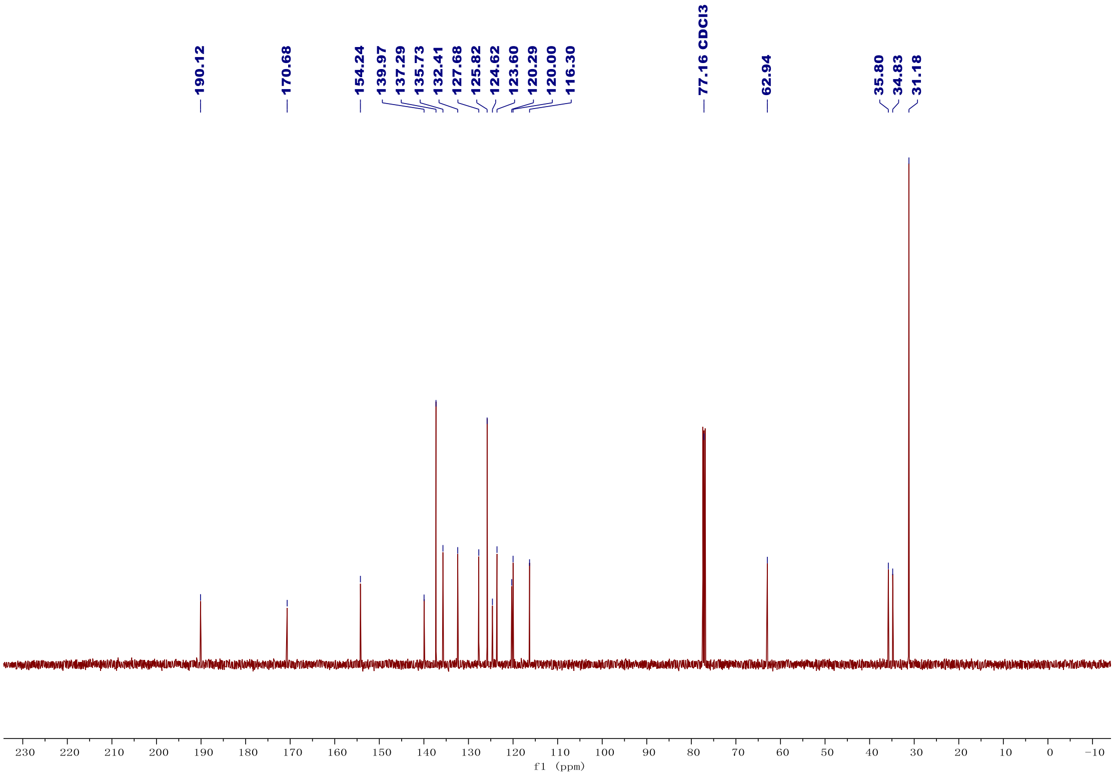

^{13}C NMR spectrum for product **5c**

1-methoxy-6,6-dimethyl-3-phenyl-7,8-dihydroquinoline-2,5(1H,6H)-dione (5d)

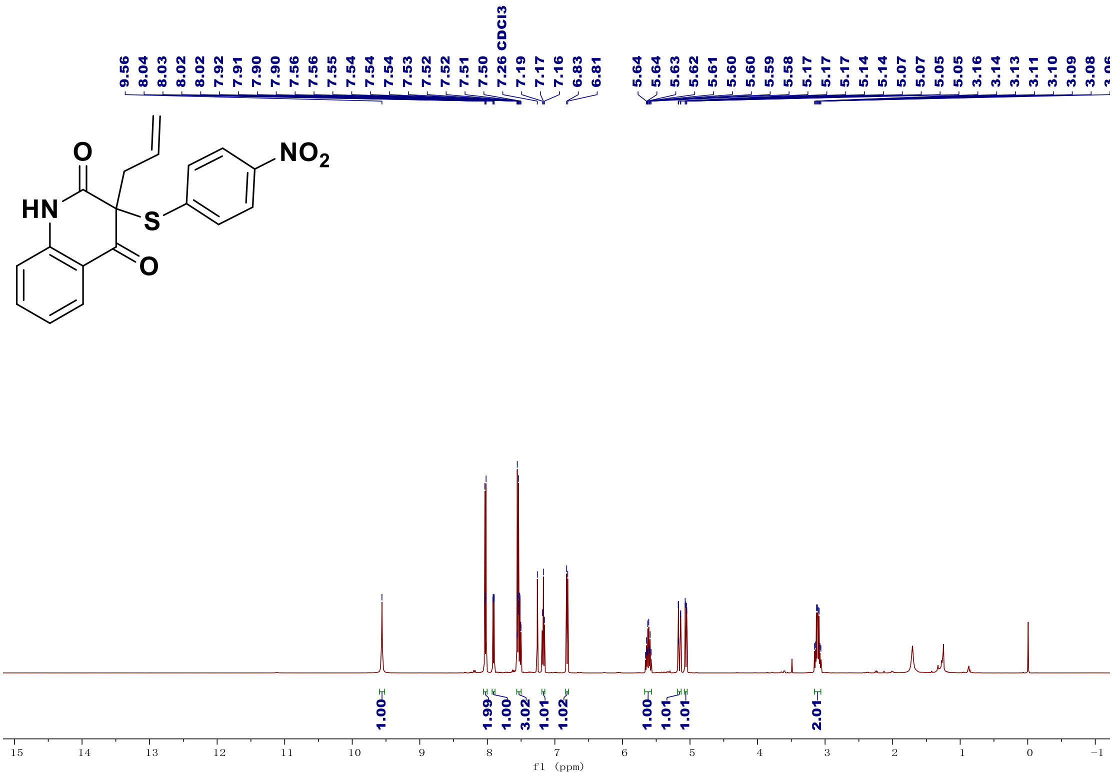

^{1}H NMR spectrum for product **5d**

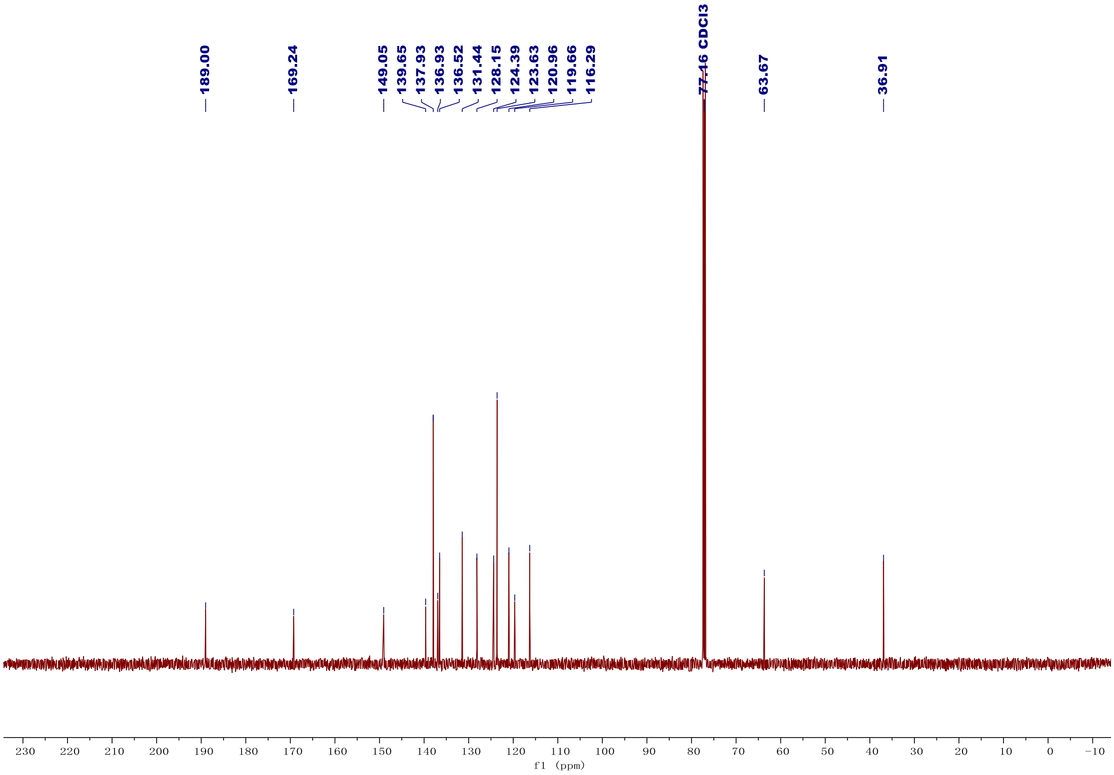

^{13}C NMR spectrum for product **5d**

3-allyl-3-(naphthalen-2-ylthio)quinoline-2,4(1H,3H)-dione (5e)

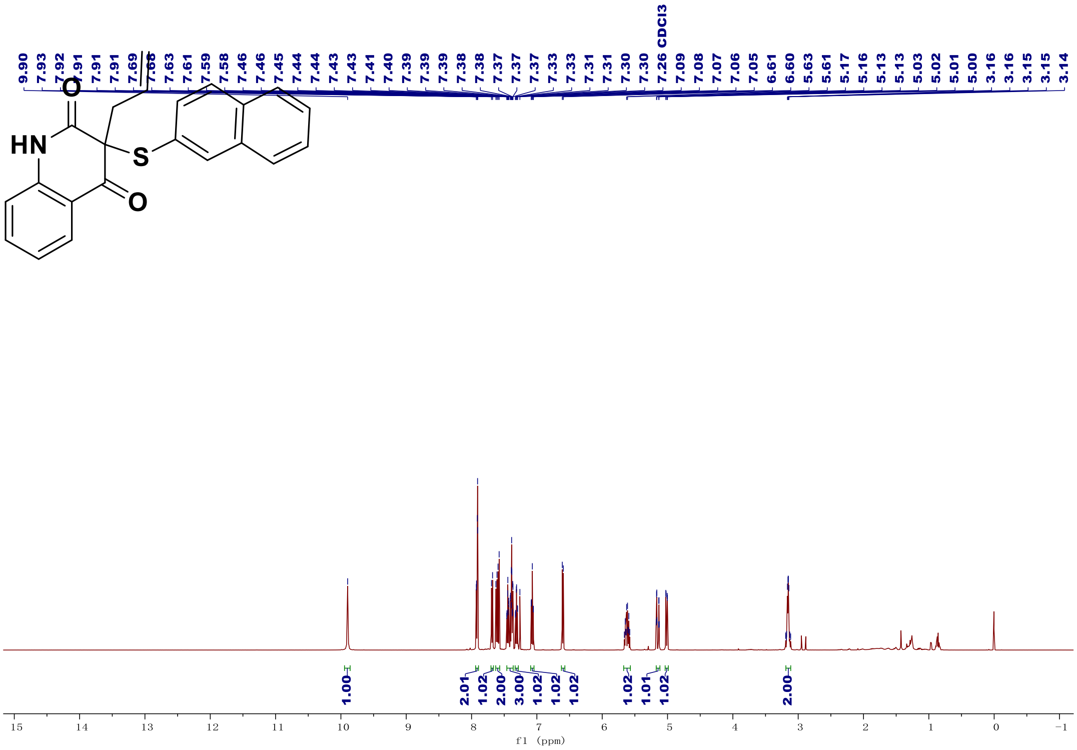

^{1}H NMR spectrum for product **5e**

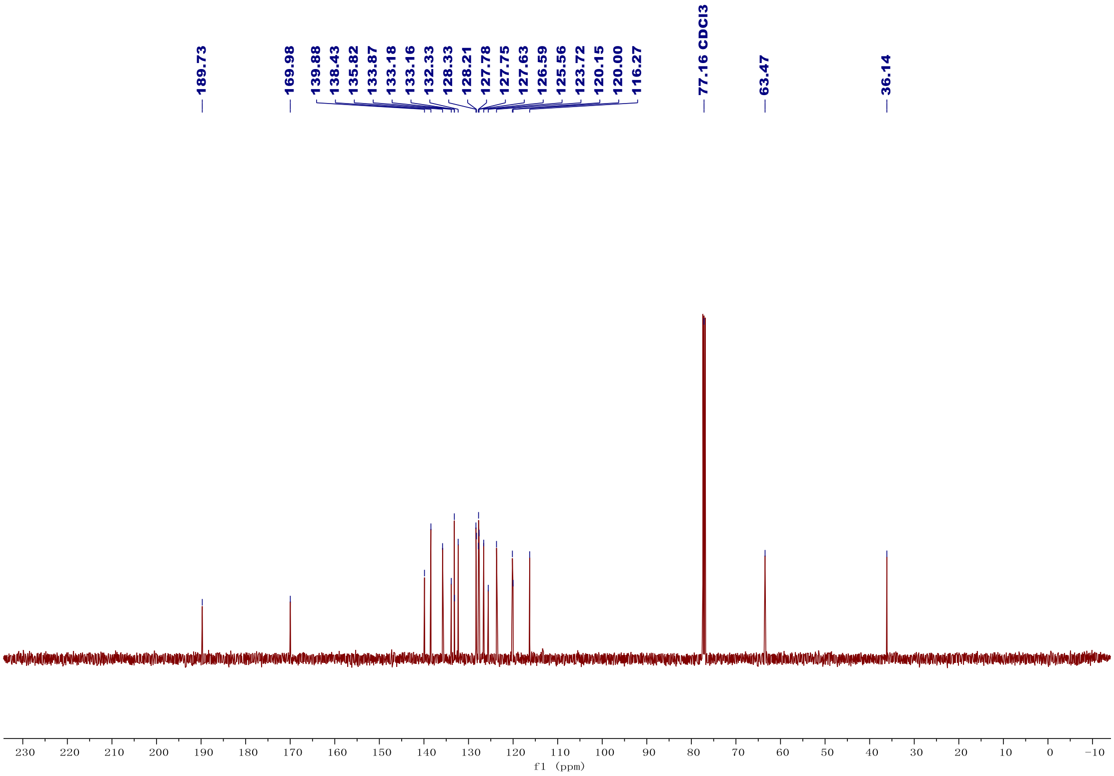

^{13}C NMR spectrum for product **5e**

3-allyl-3-(phenylthio)quinoline-2,4(1H,3H)-dione (5f)

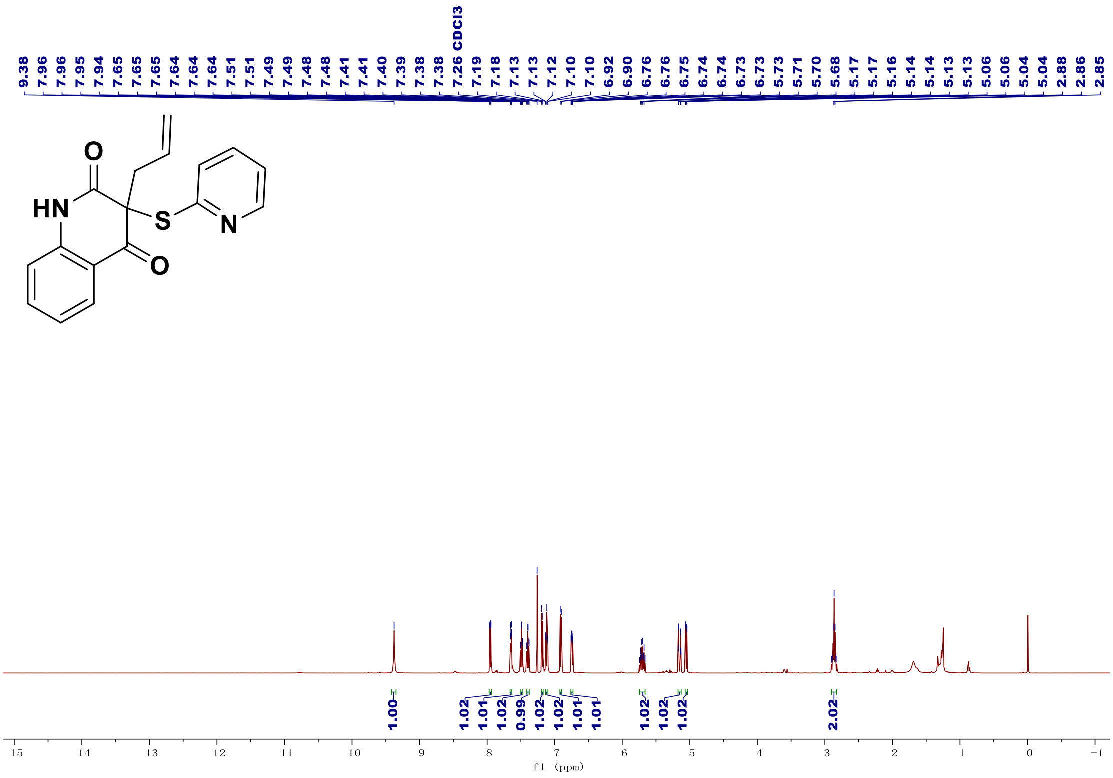

^{1}H NMR spectrum for product **5f**

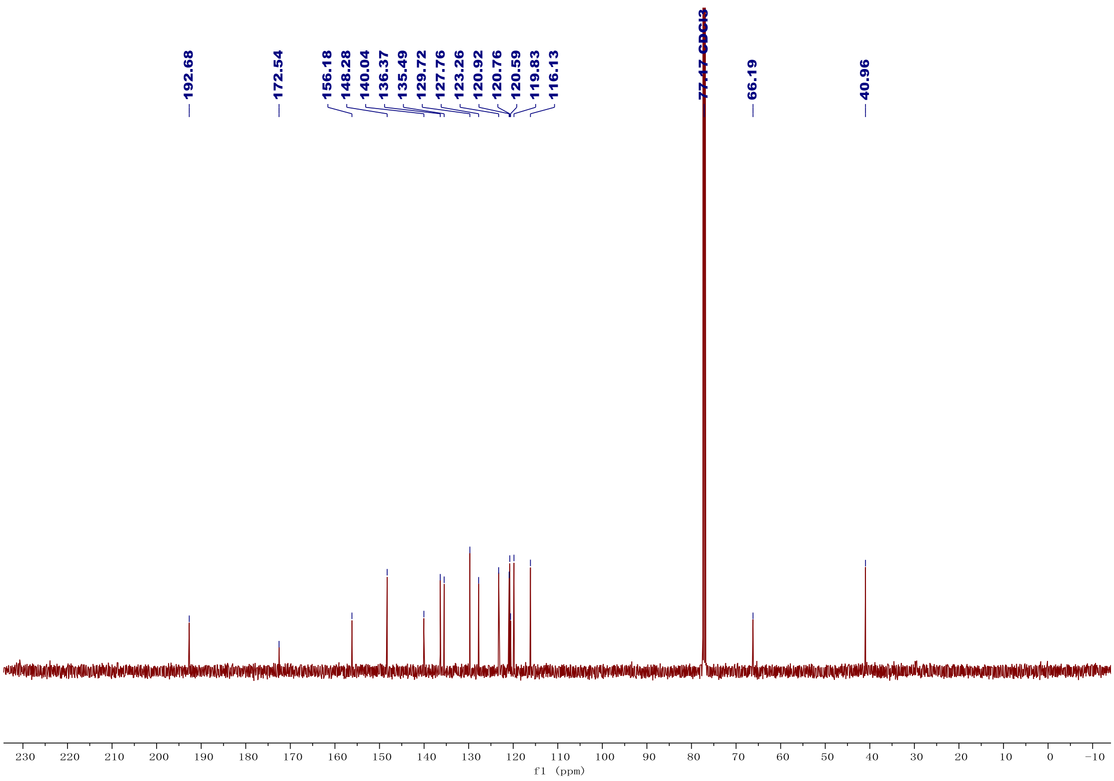

^{13}C NMR spectrum for product **5f**

3-allyl-3-(phenylthio)quinoline-2,4(1H,3H)-dione (5g)

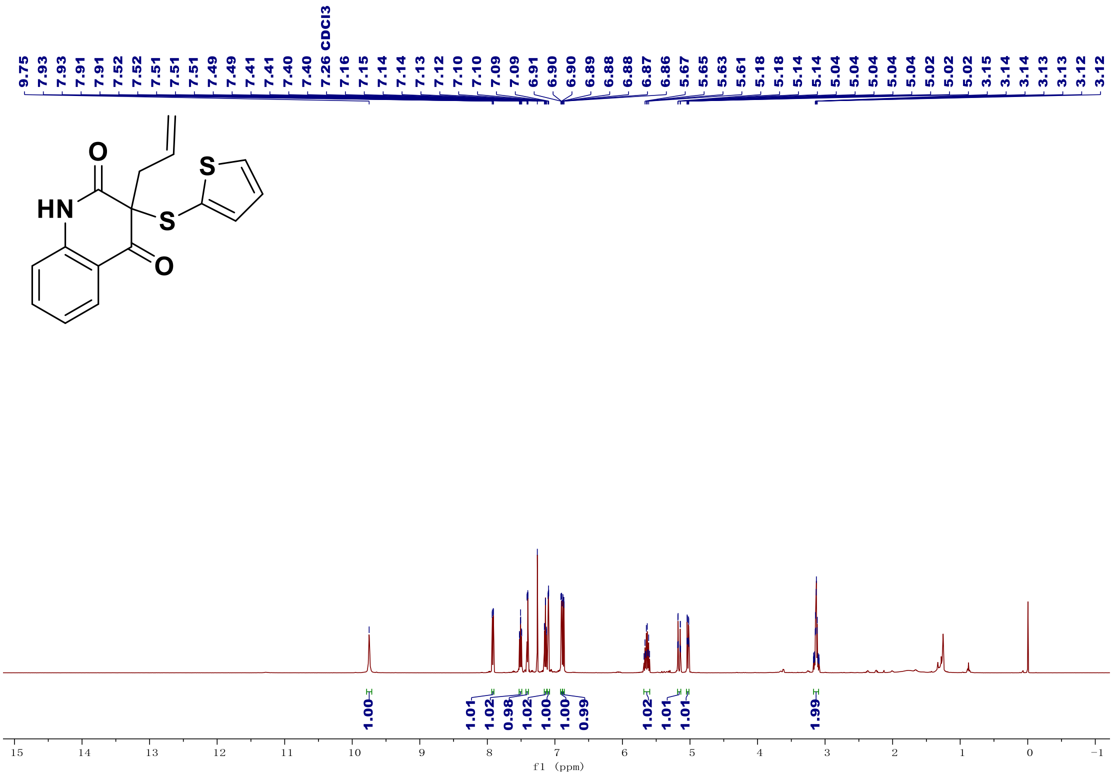

^{1}H NMR spectrum for product **5g**

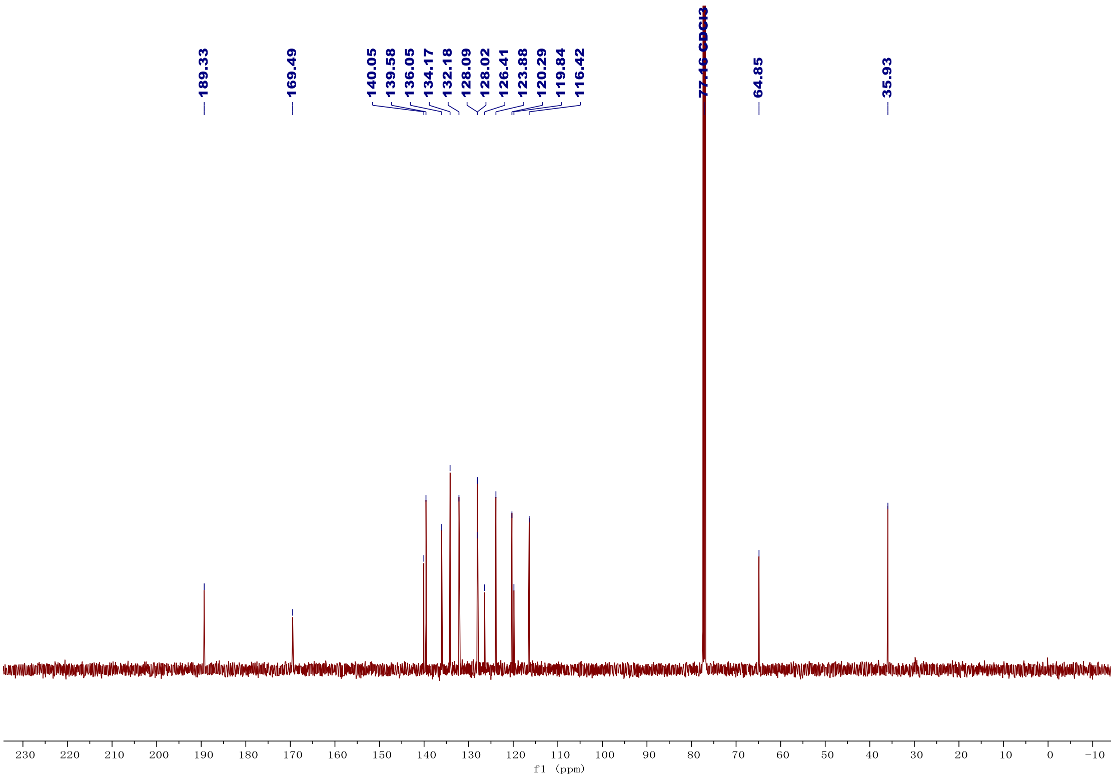

^{13}C NMR spectrum for product **5g**

3-allyl-3-(benzylthio)quinoline-2,4(1H,3H)-dione (5h)

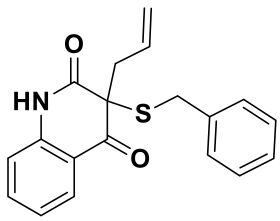

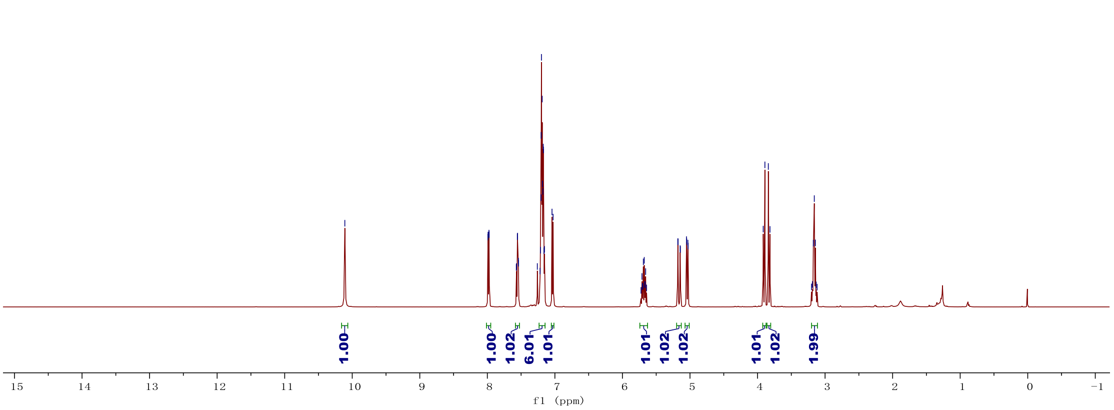

^{1}H NMR spectrum for product **5h**

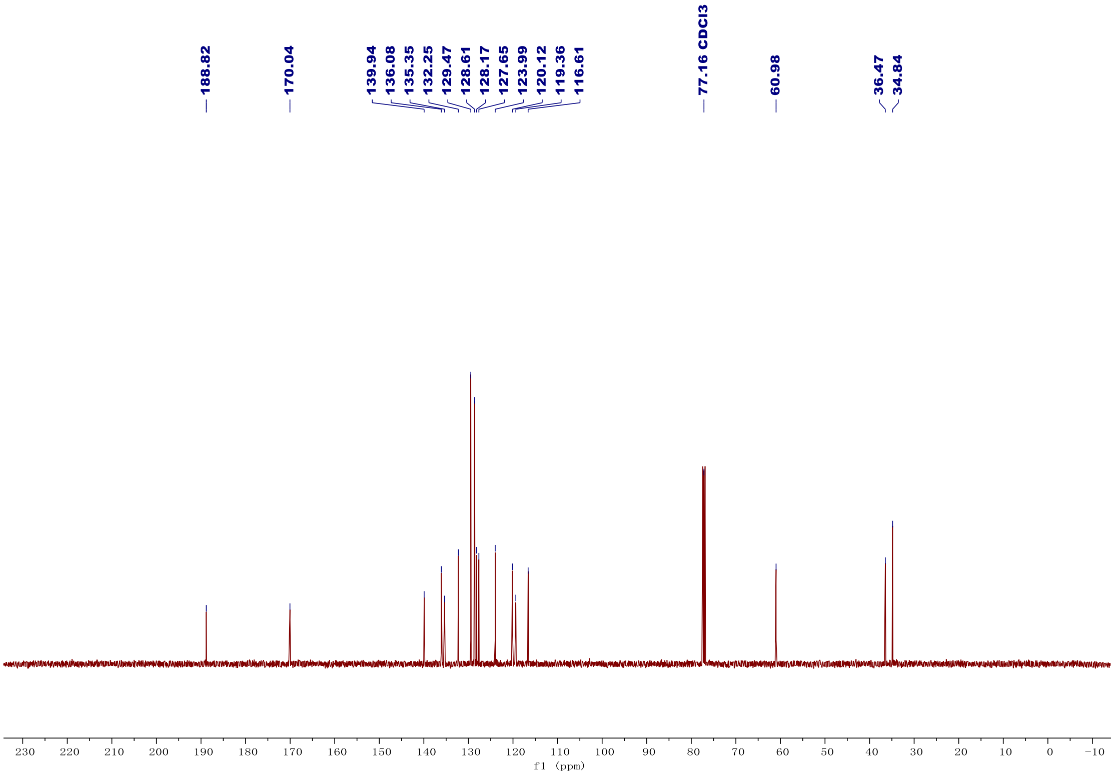

^{13}C NMR spectrum for product **5h**

3-allyl-3-(phenylthio)quinoline-2,4(1H,3H)-dione (5i)

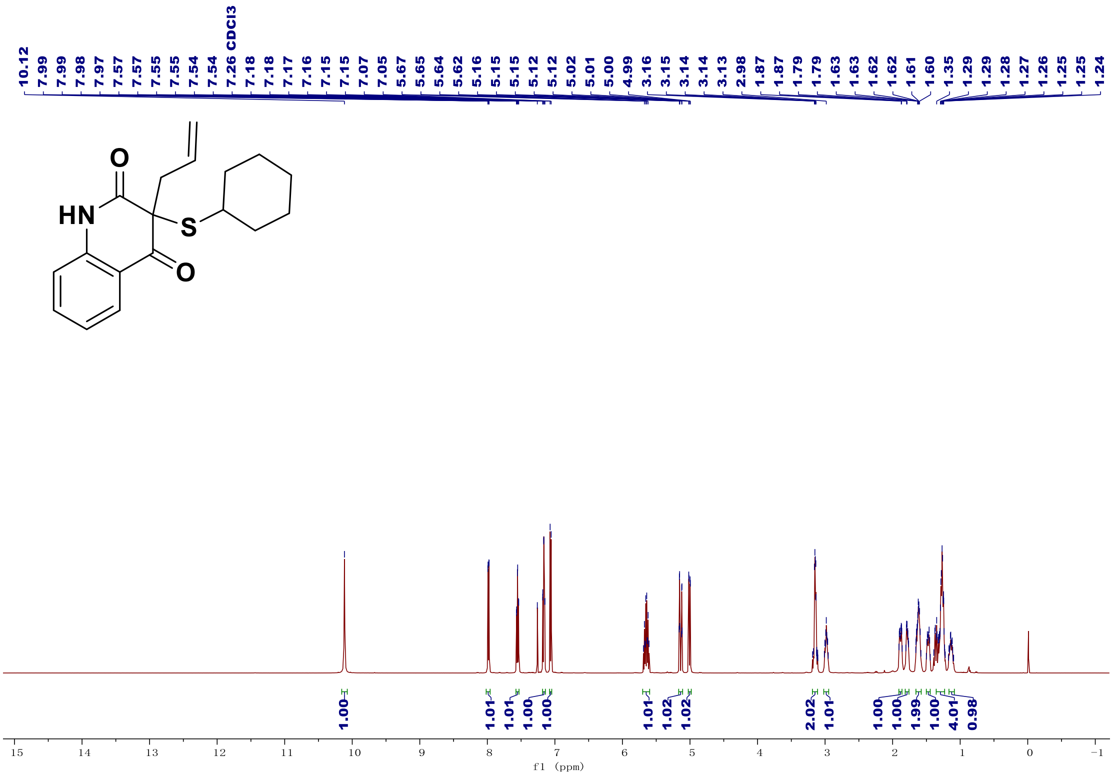

^{1}H NMR spectrum for product **5i**

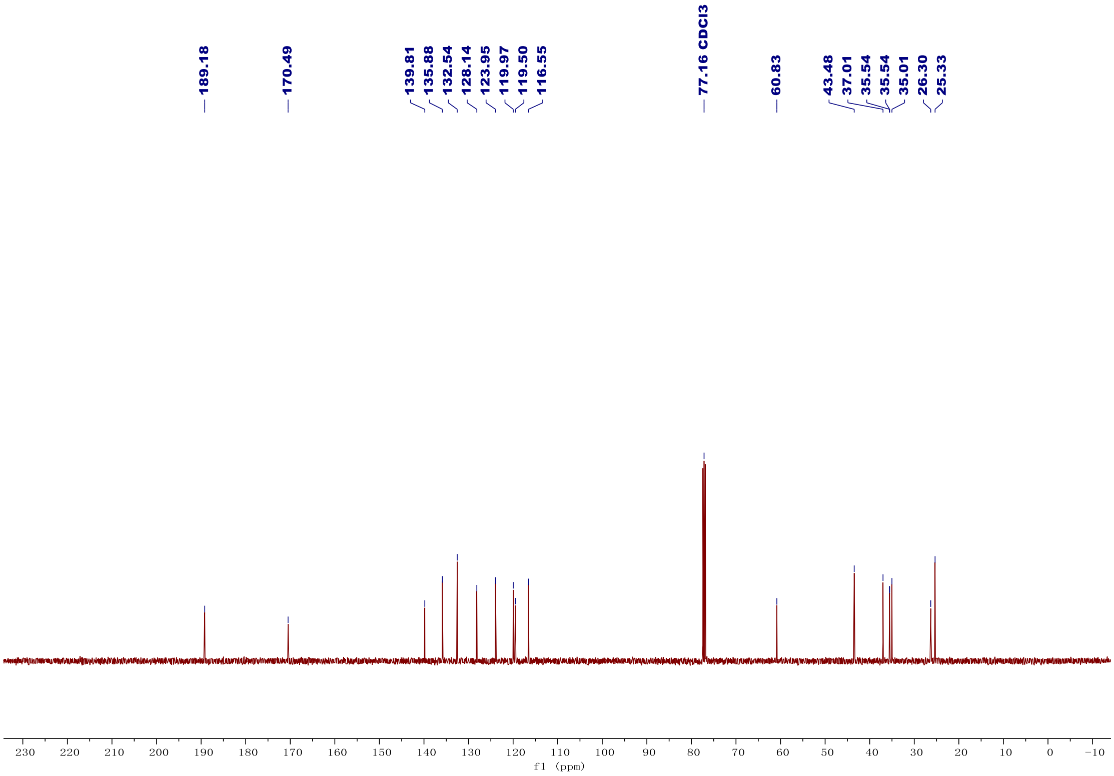

^{13}C NMR spectrum for product **5i**

3-allyl-3-(allylthio)-1-methylquinoline-2,4(1H,3H)-dione (5j)

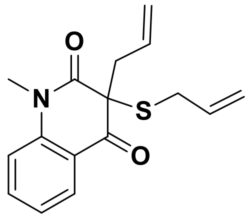

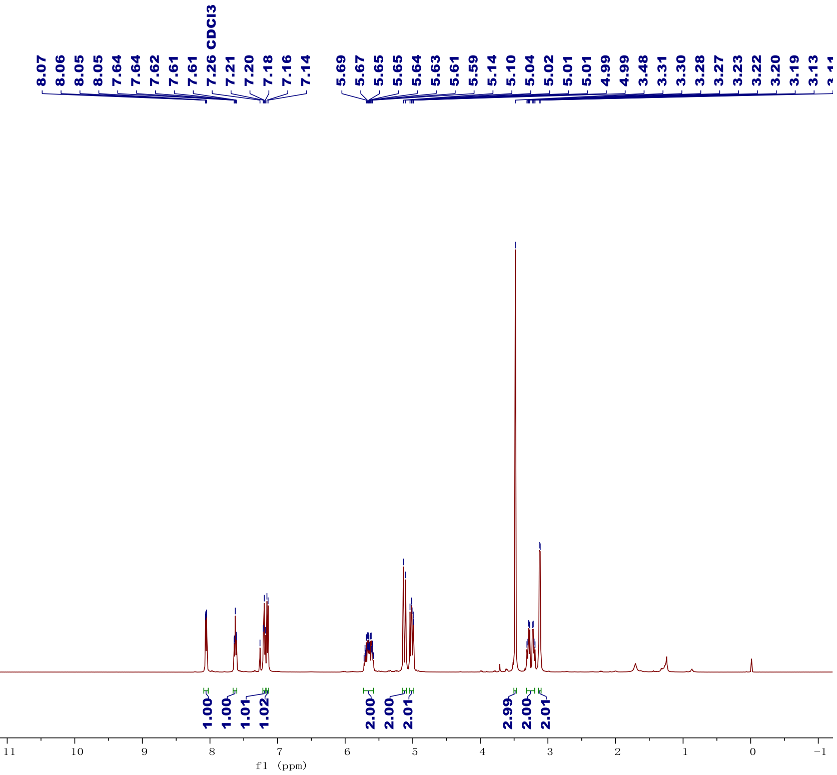

^{1}H NMR spectrum for product **5j**

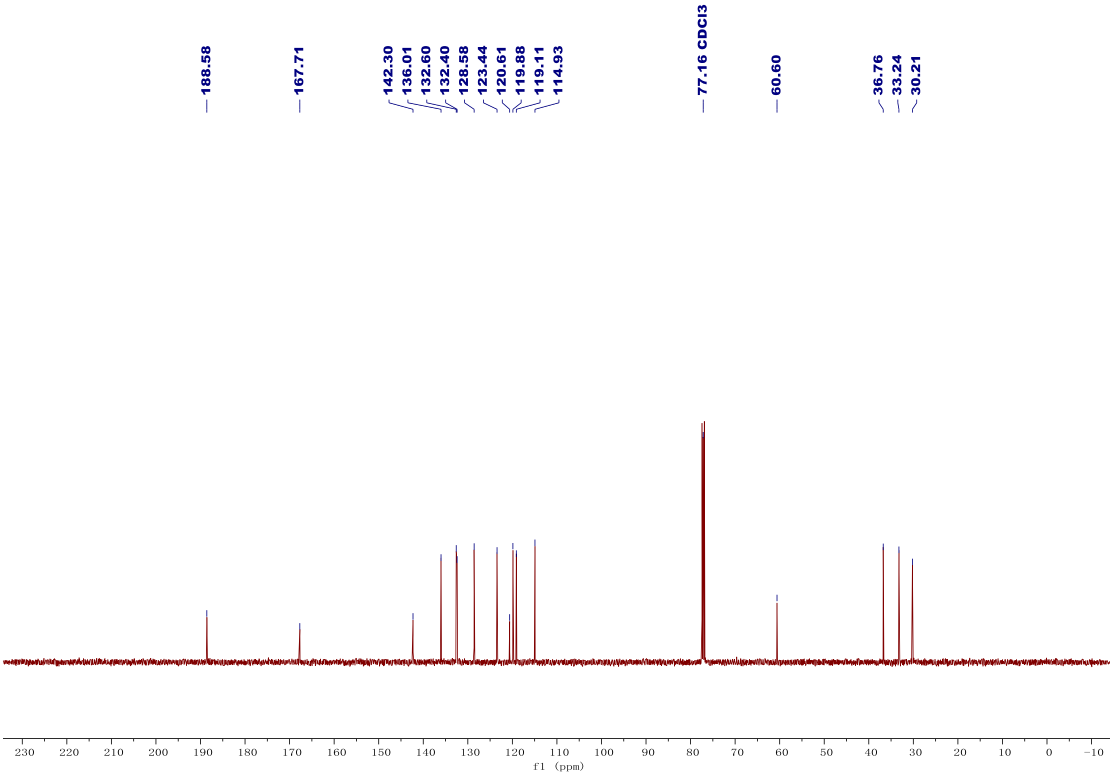

^{13}C NMR spectrum for product **5j**

3-allyl-3-(phenylthio)quinoline-2,4(1H,3H)-dione (5k)

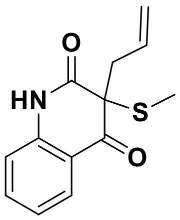

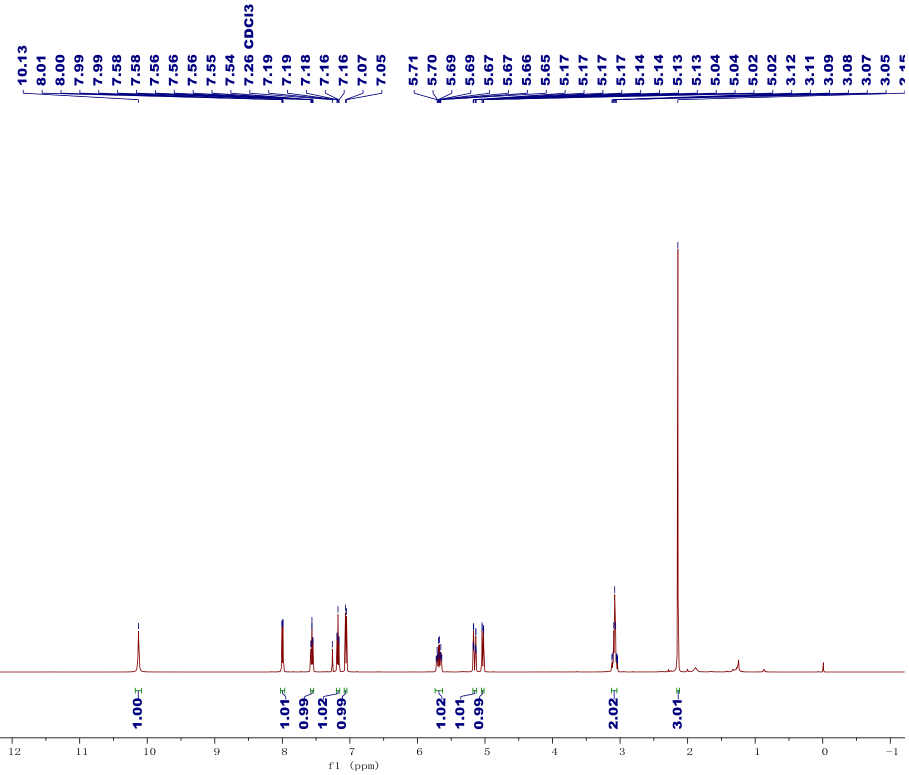

^{1}H NMR spectrum for product **5k**

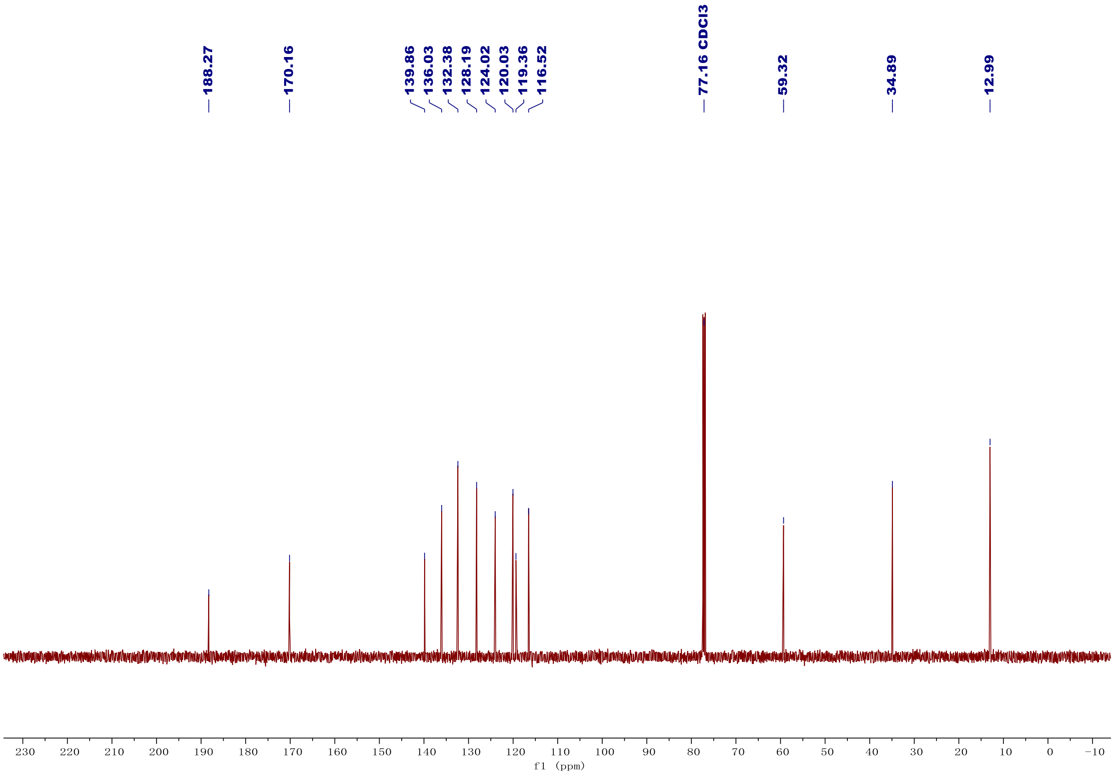

^{13}C NMR spectrum for product **5k**

3-(but-3-en-2-yl)-3-((3-chlorophenyl)thio)quinoline-2,4(1H,3H)-dione (5l)

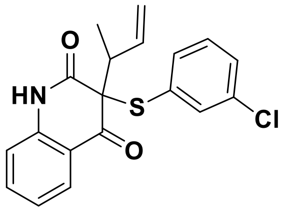

dr = 1:1

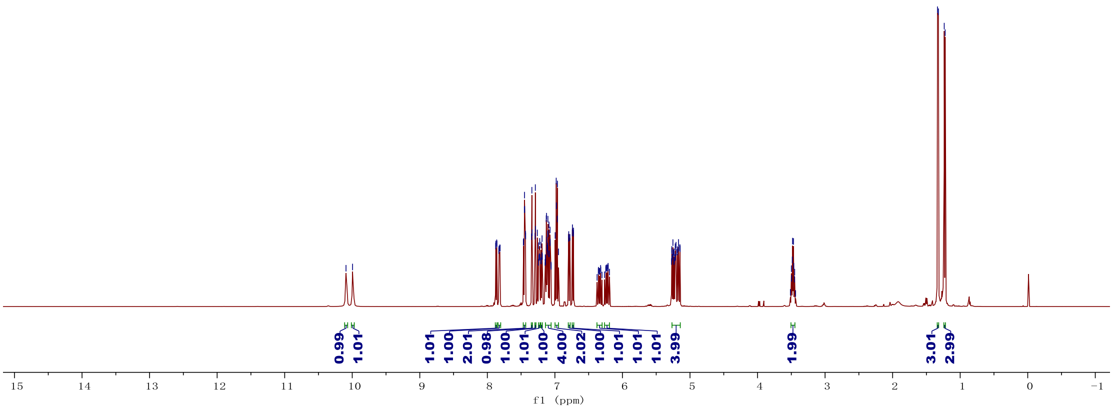

^{1}H NMR spectrum for product **5l**

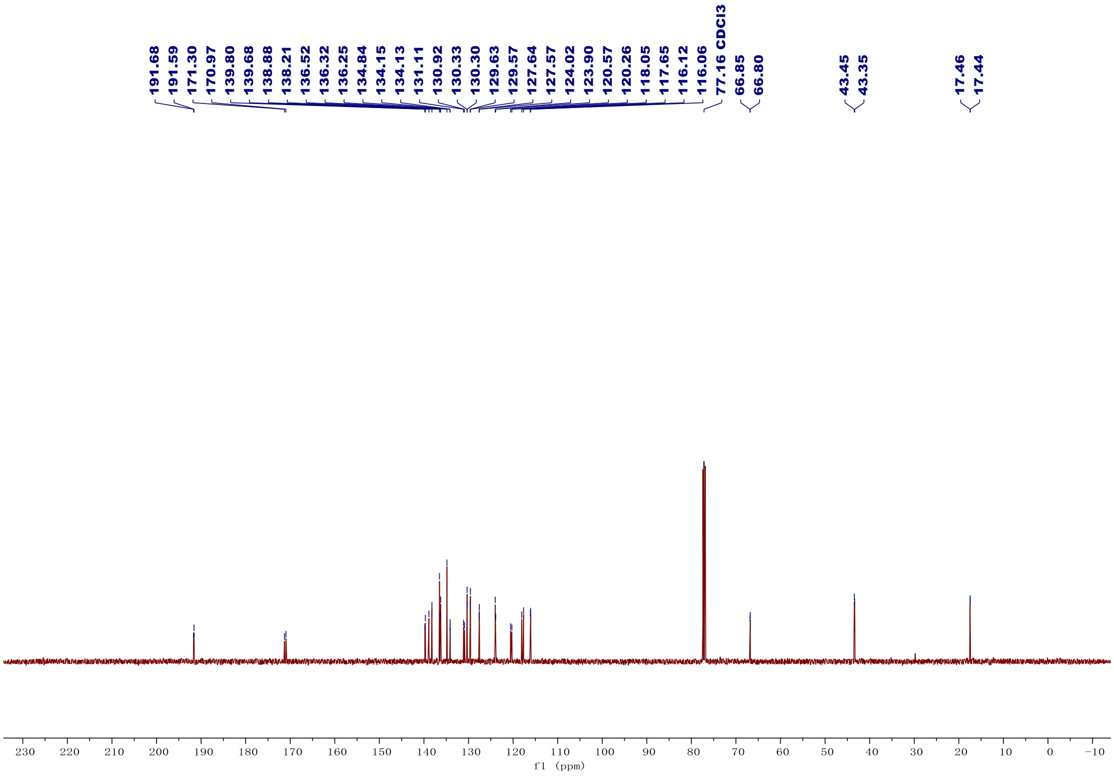

^{13}C NMR spectrum for product **5l**

3-allyl-1-methyl-3-(phenylthio)quinoline-2,4(1H,3H)-dione (5m)

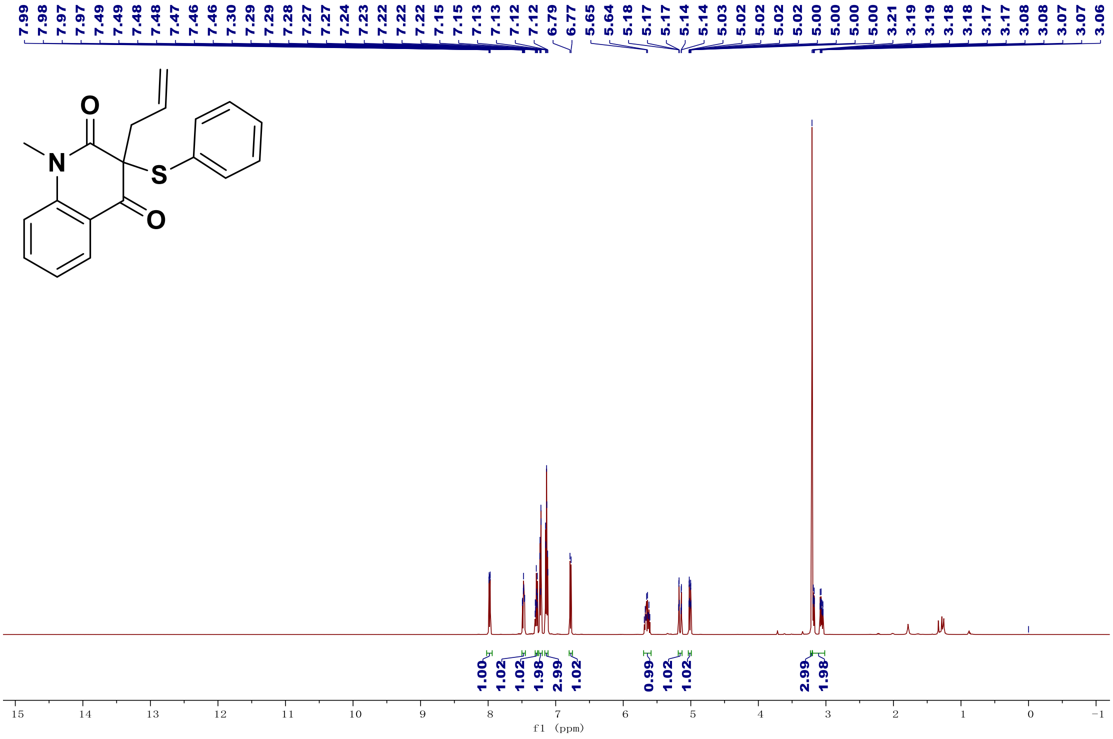

^{1}H NMR spectrum for product **5m**

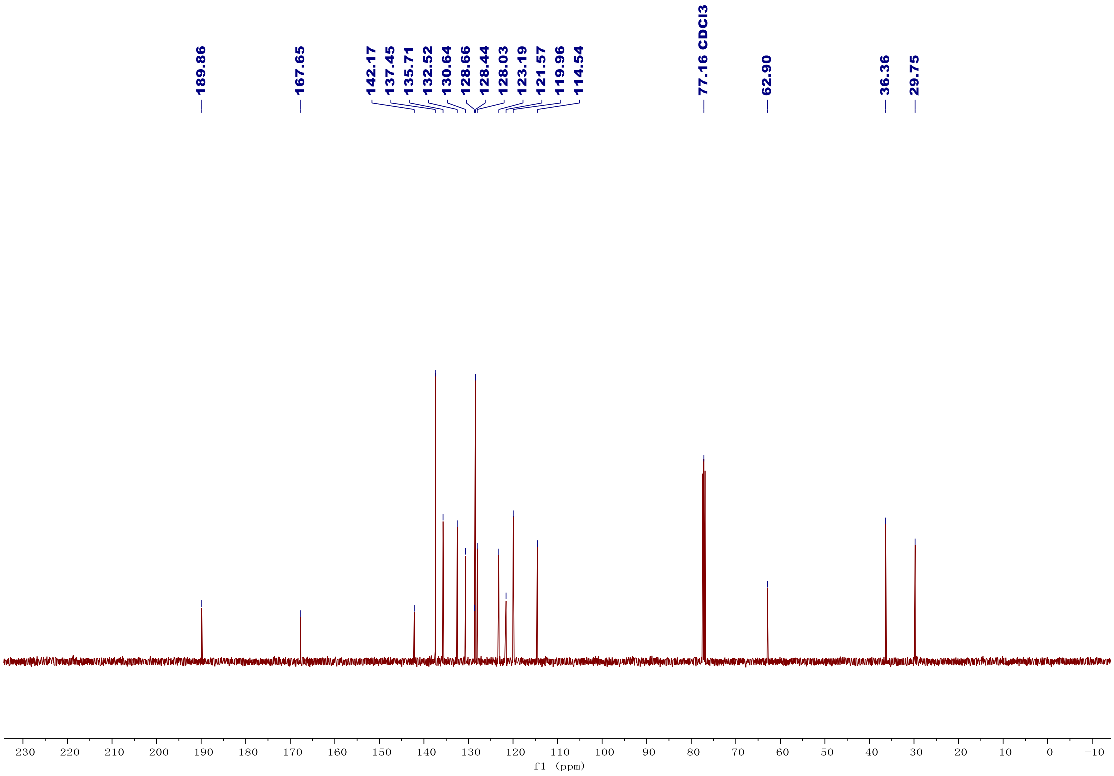

^{13}C NMR spectrum for product **5m**

3-allyl-3-(phenylthio)pyridine-2,4(1H,3H)-dione (5n)

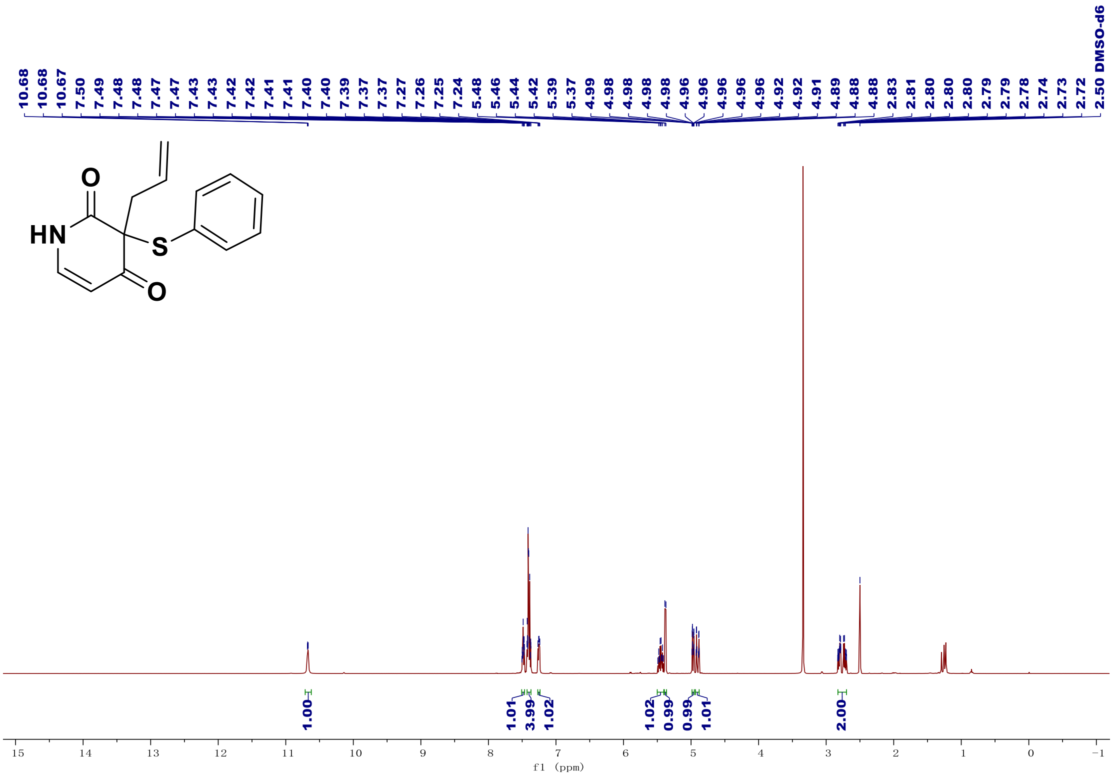

^{1}H NMR spectrum for product **5n**

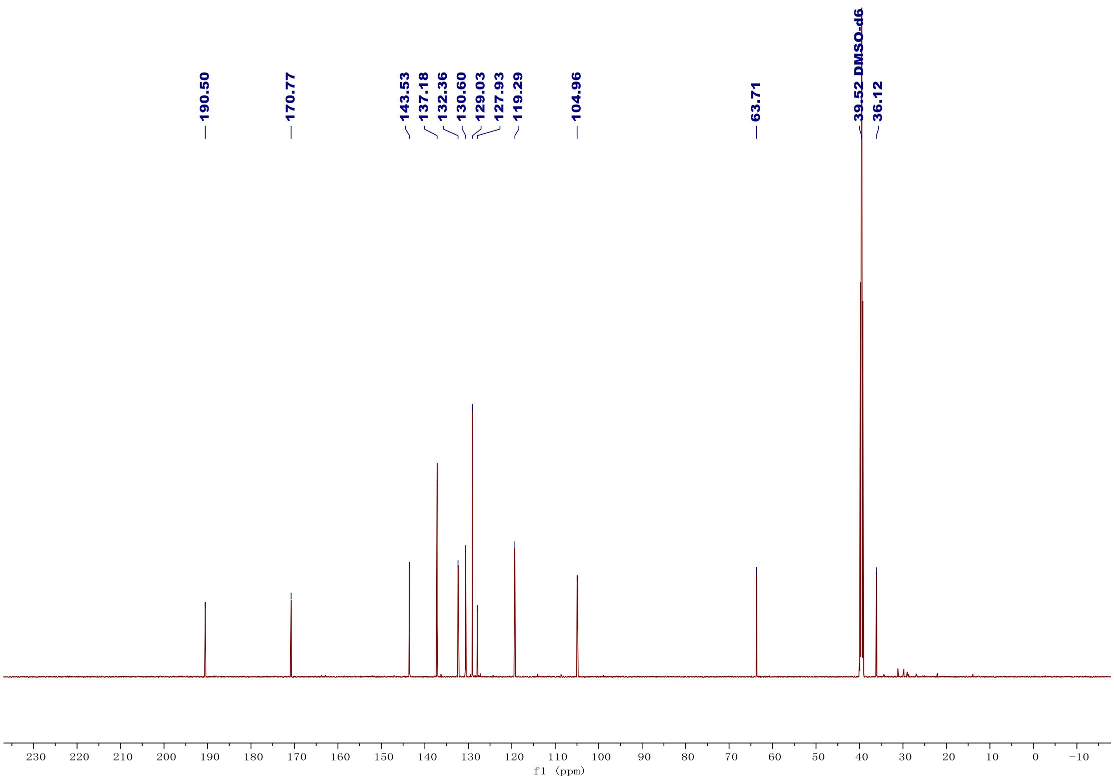

^{13}C NMR spectrum for product **5n**

3-allyl-6-methyl-3-(phenylthio)pyridine-2,4(1H,3H)-dione (5o)

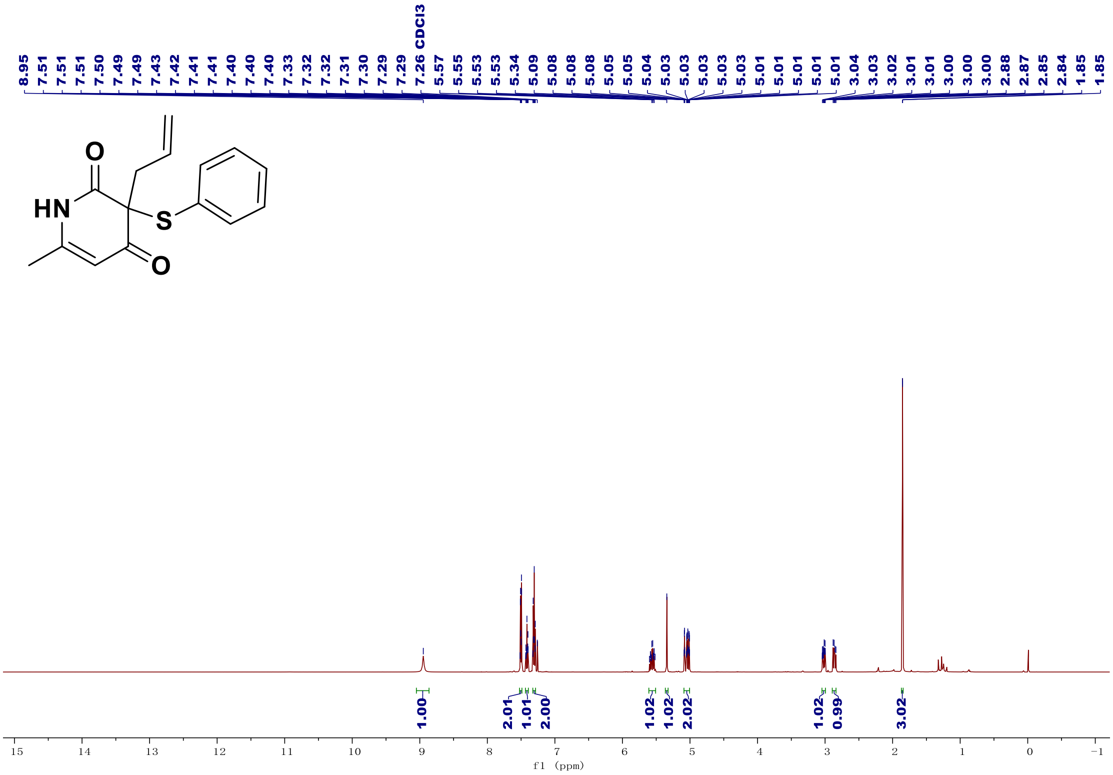

^{1}H NMR spectrum for product **5o**

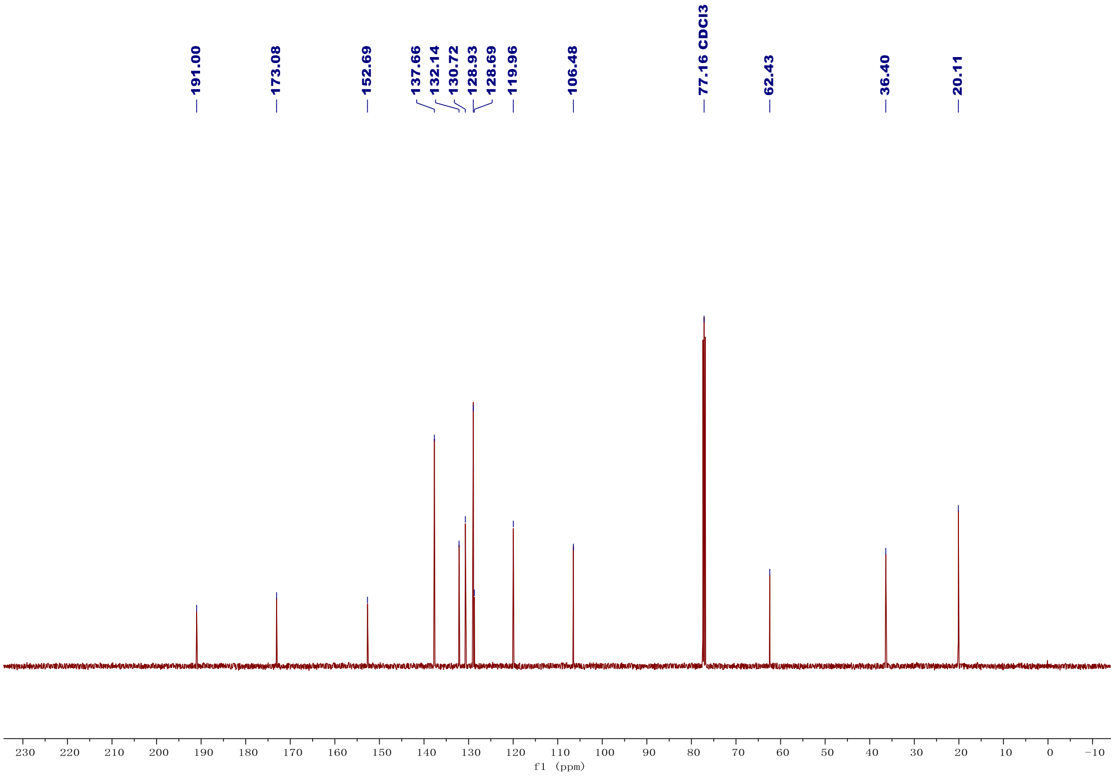

^{13}C NMR spectrum for product **5o**

2-allyl-2-(phenylthio)cyclohexane-1,3-dione (5p)

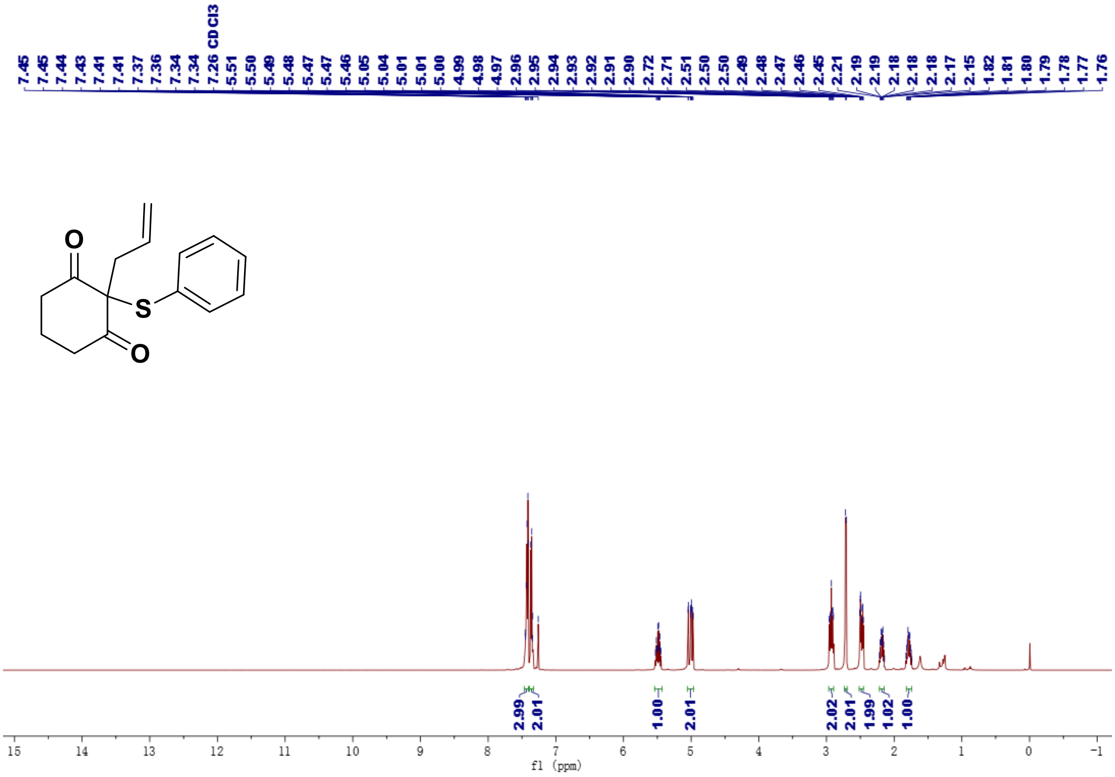

^{1}H NMR spectrum for product **5p**

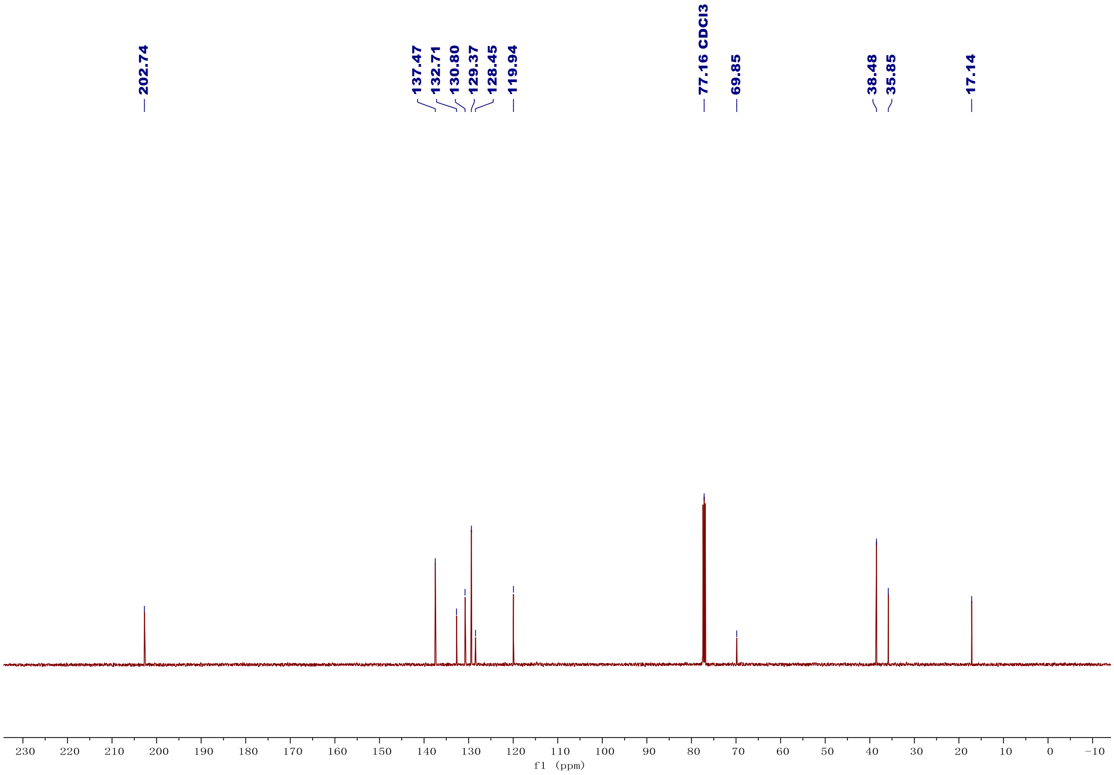

^{13}C NMR spectrum for product **5p**

2-allyl-5,5-dimethyl-2-(phenylthio)cyclohexane-1,3-dione (5q)

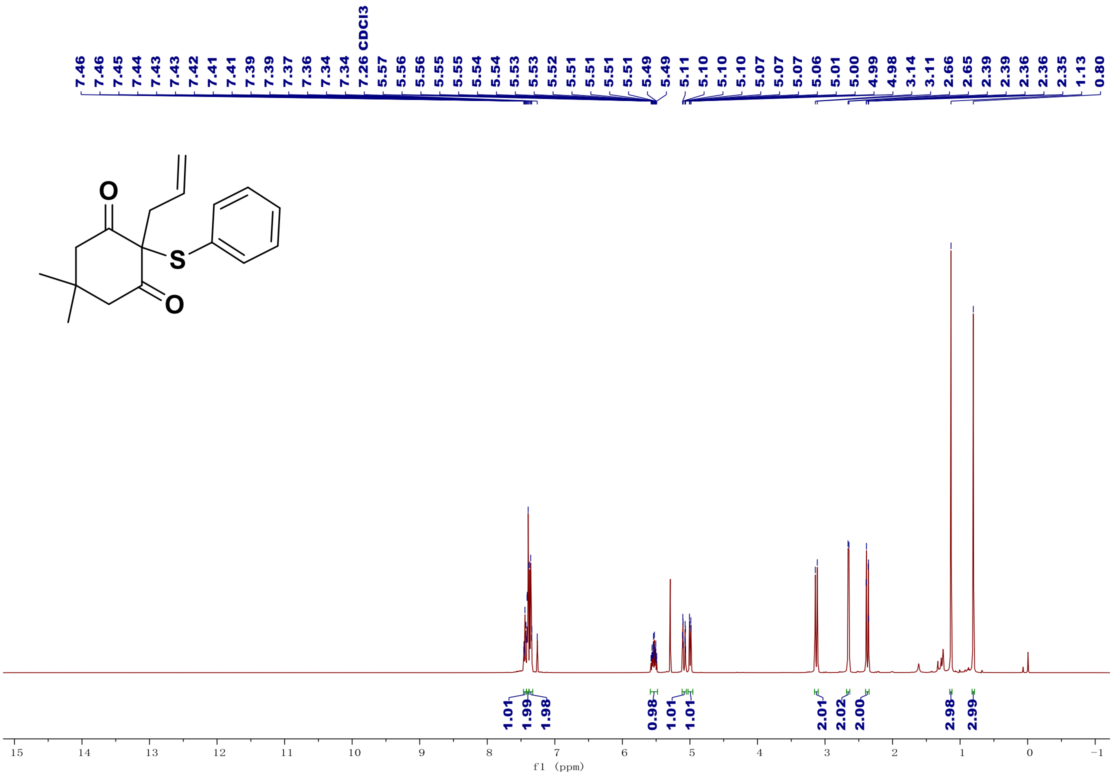

^{1}H NMR spectrum for product **5q**

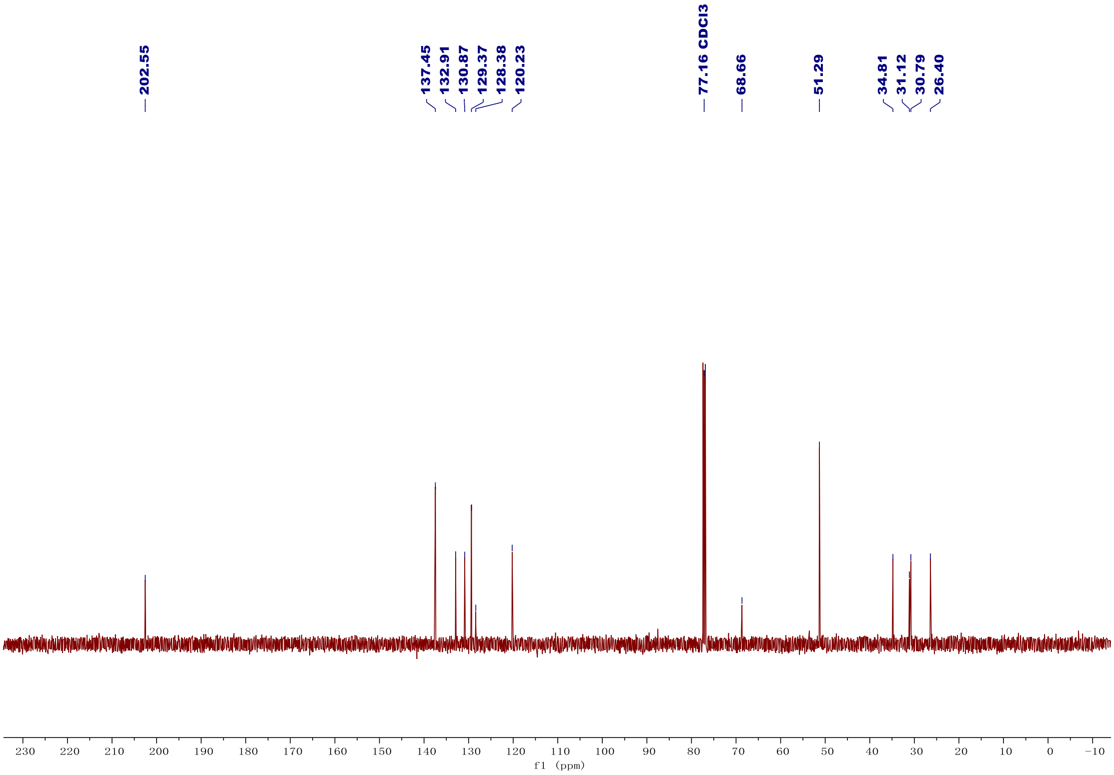

^{13}C NMR spectrum for product **5q**

7-allyl-7-(phenylthio)spiro[3.5]nonane-6,8-dione (5r)

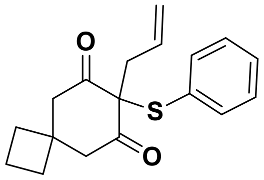

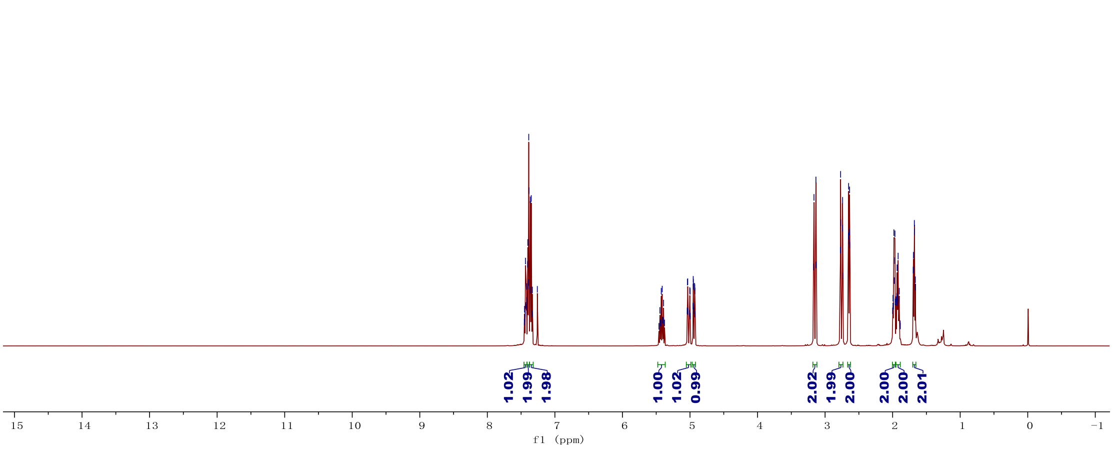

^{1}H NMR spectrum for product **5r**

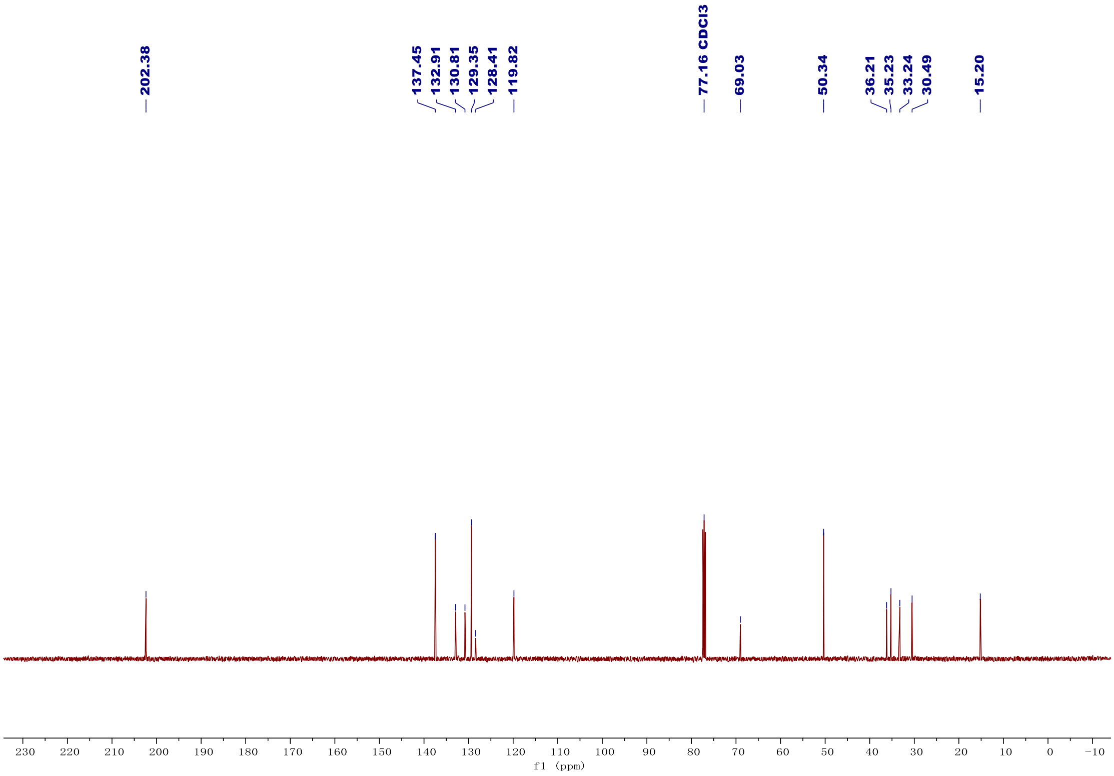

^{13}C NMR spectrum for product **5r**

2-allyl-2-(phenylthio)cyclopentane-1,3-dione (5s)

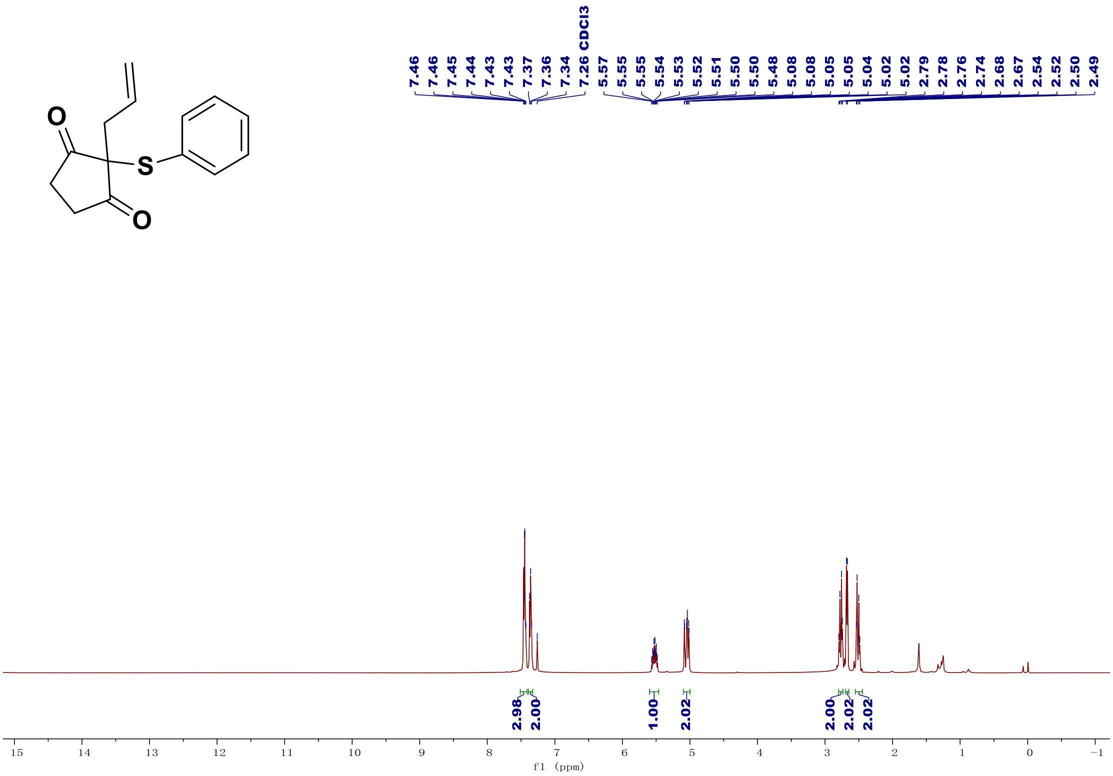

^{1}H NMR spectrum for product **5s**

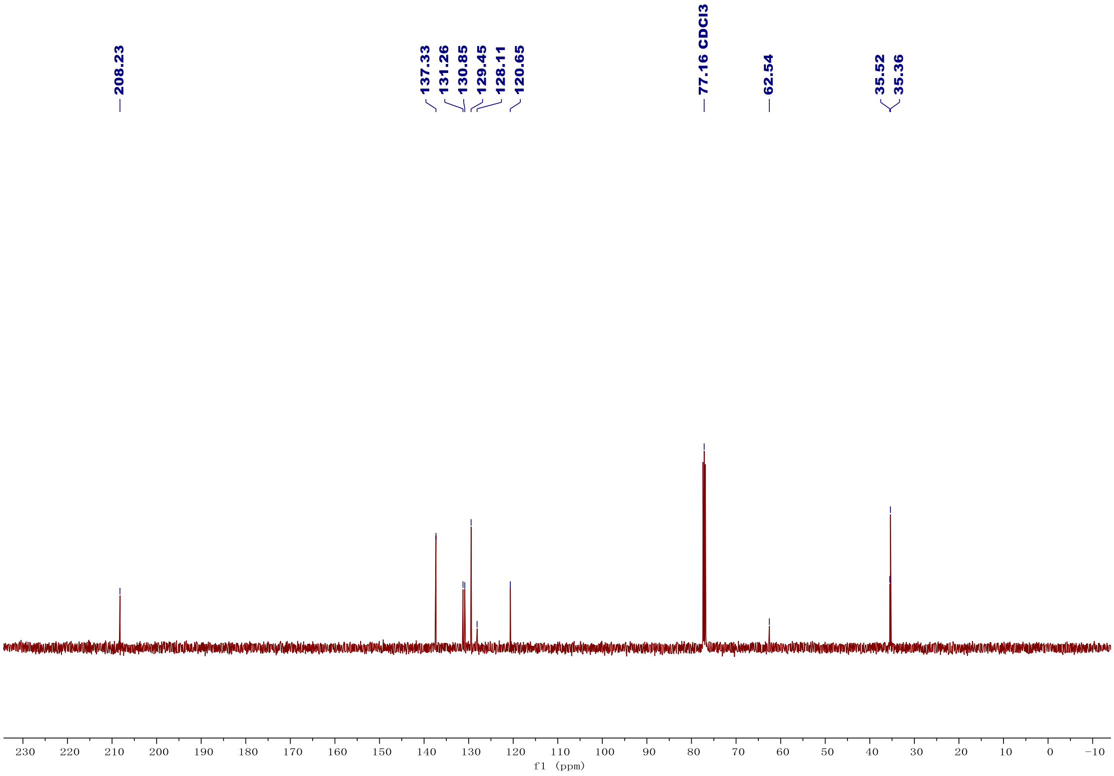

^{13}C NMR spectrum for product **5s**

dimethyl 2-allyl-2-(phenylthio)malonate (5t)

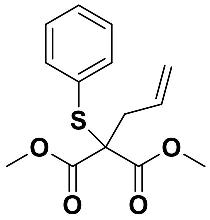

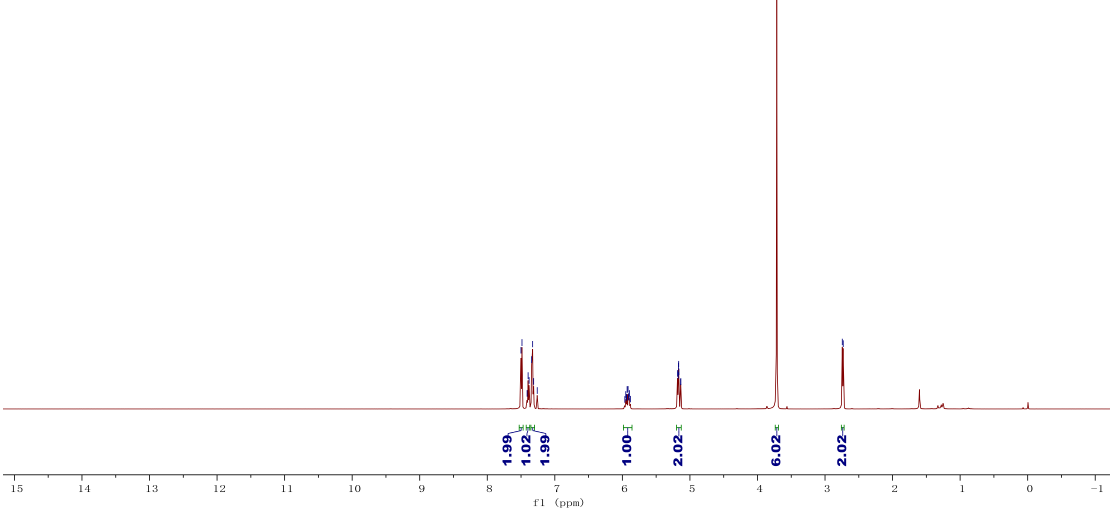

^{1}H NMR spectrum for product **5t**

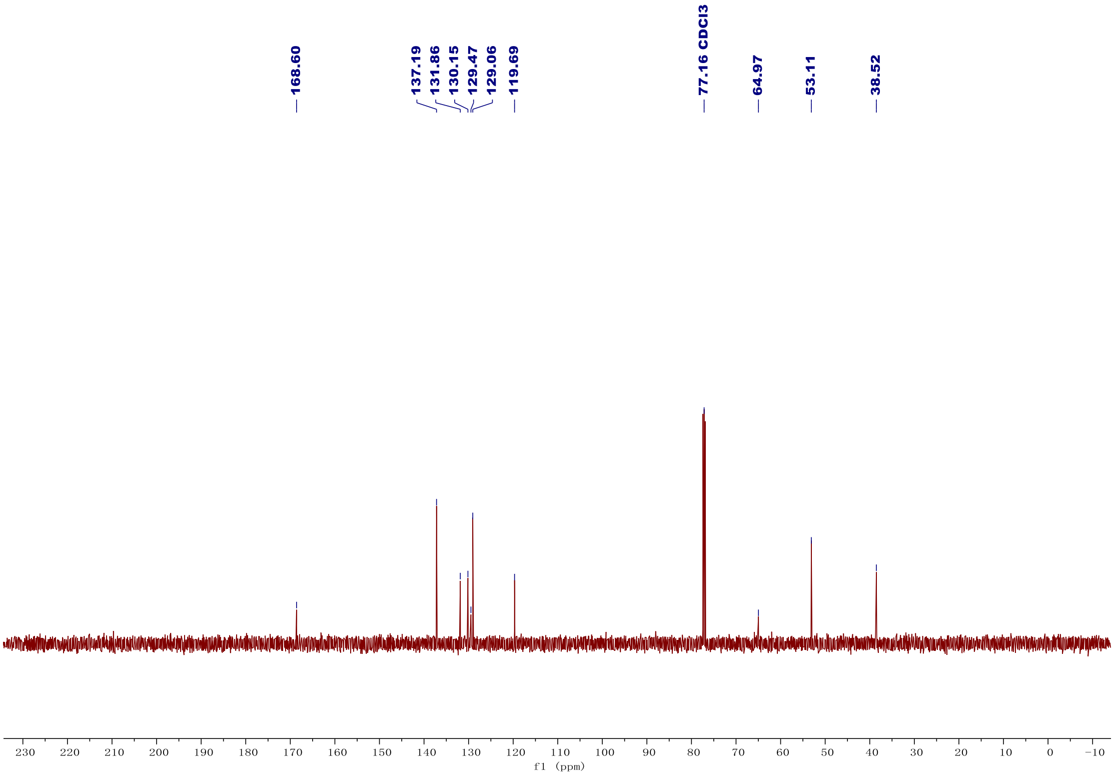

^{13}C NMR spectrum for product **5t**

2-allyl-1,3-diphenyl-2-(phenylthio)propane-1,3-dione (5u)

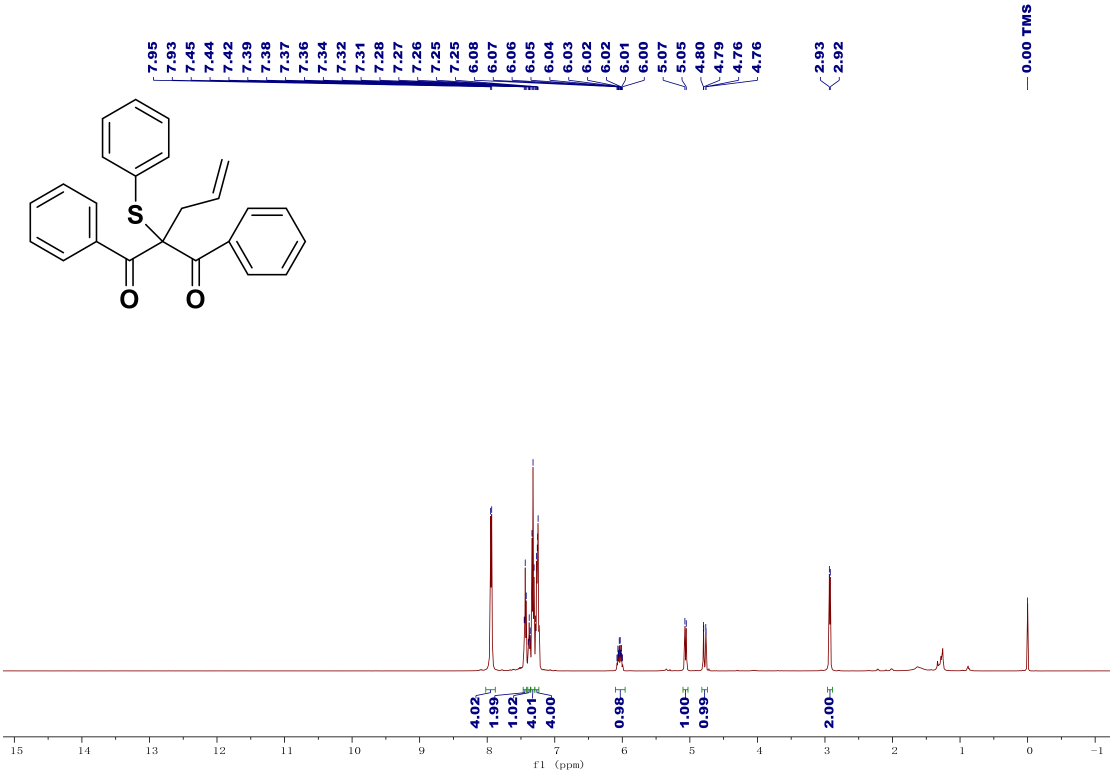

^{1}H NMR spectrum for product **5u**

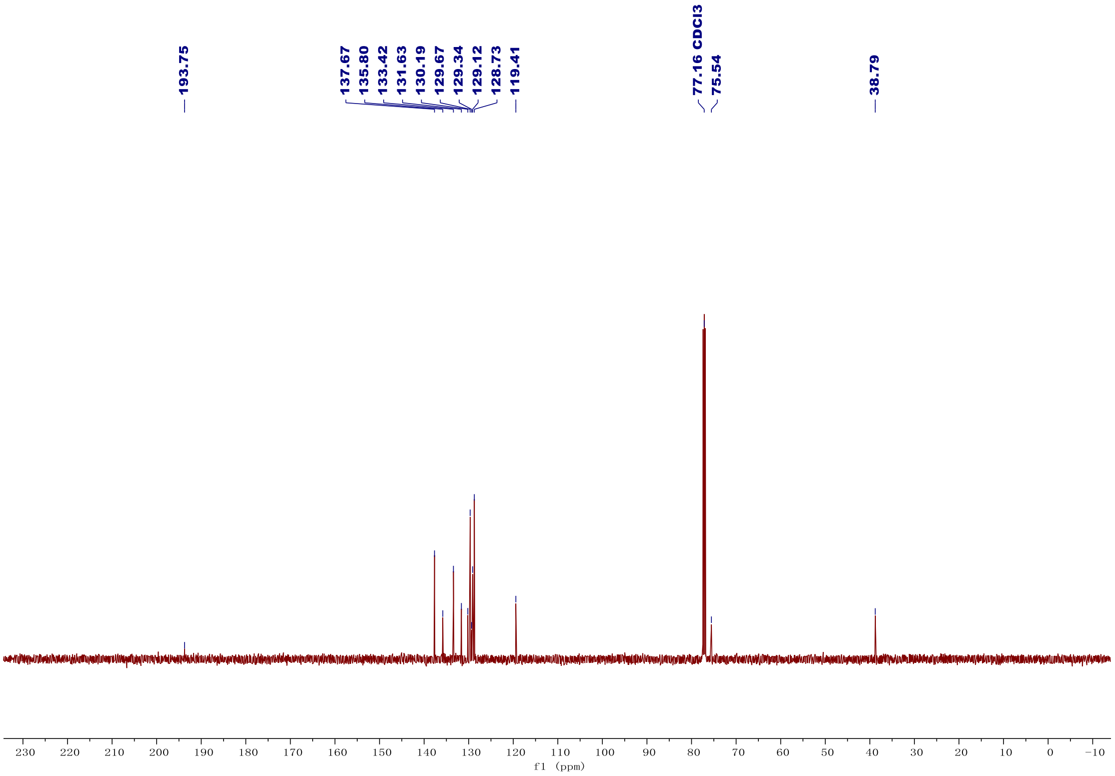

^{13}C NMR spectrum for product **5u**

2-allyl-1,3-diphenyl-2-(phenylselanyl)propane-1,3-dione (5v)

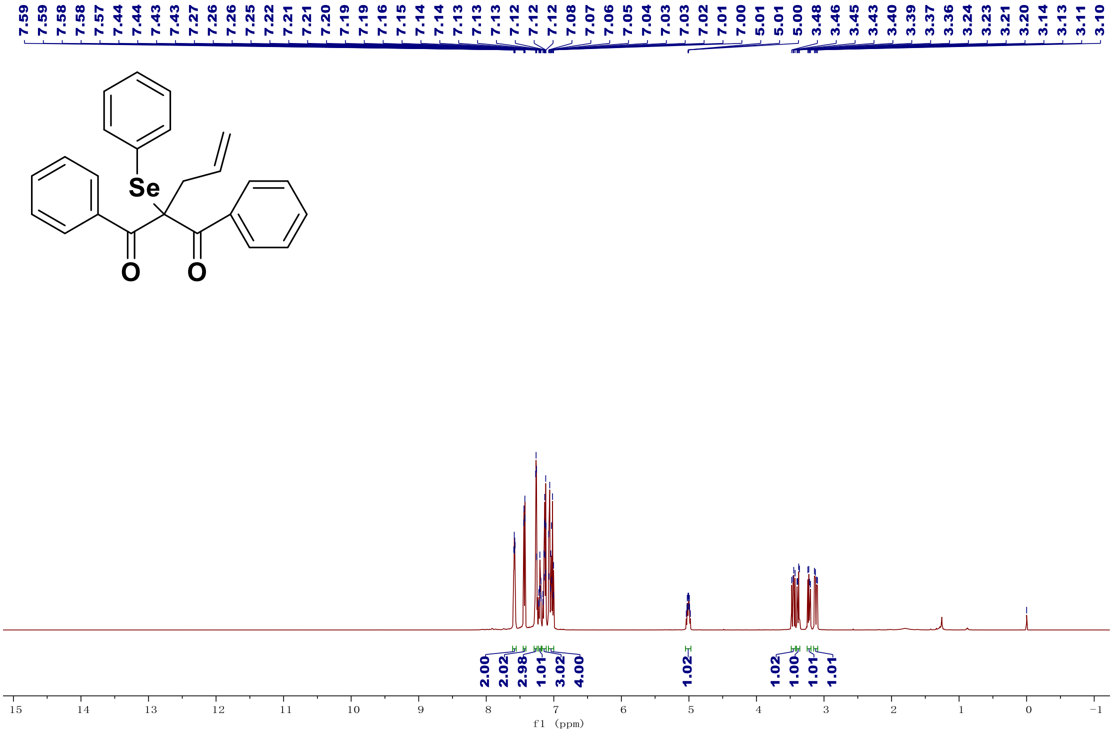

^{1}H NMR spectrum for product **5v**

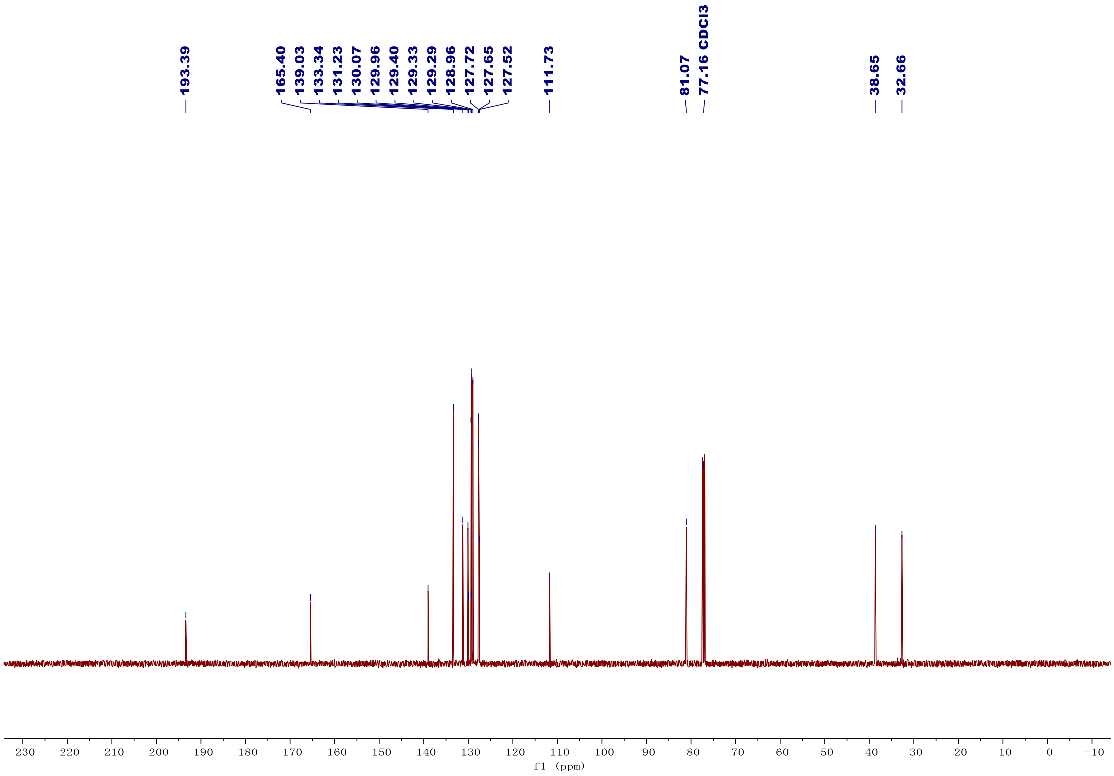

^{13}C NMR spectrum for product **5v**

1,3-diphenyl-2-(phenylthio)-2-(propa-1,2-dien-1-yl)propane-1,3-dione (5w)

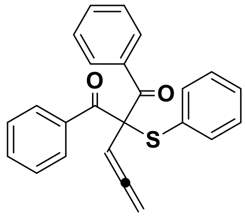

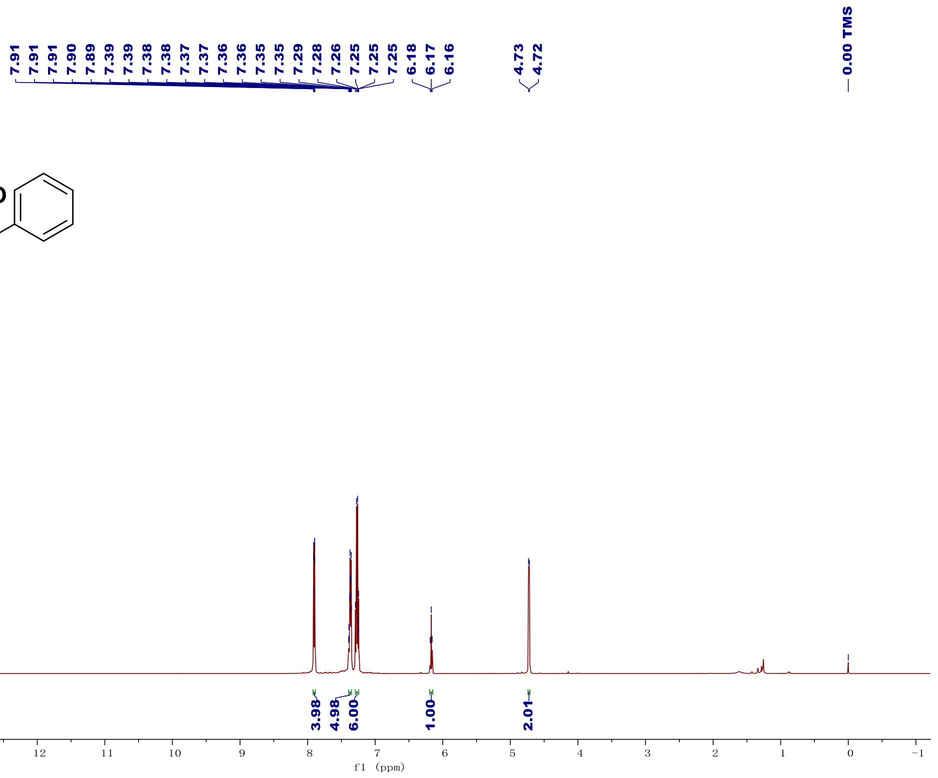

^{1}H NMR spectrum for product **5w**

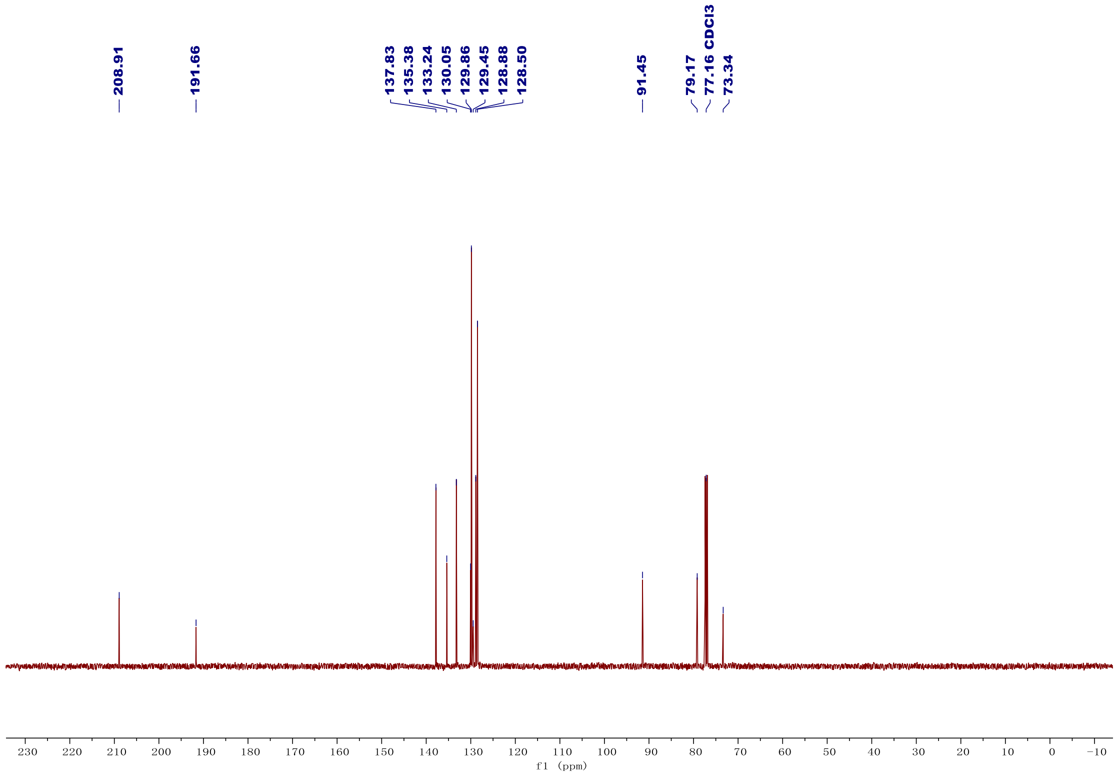

^{13}C NMR spectrum for product **5w**

(*Z*)-*N*-((2,4-dioxochroman-3-ylidene)(4-methoxyphenyl)-λ⁴-sulfaneyl)pivalamide (7a)

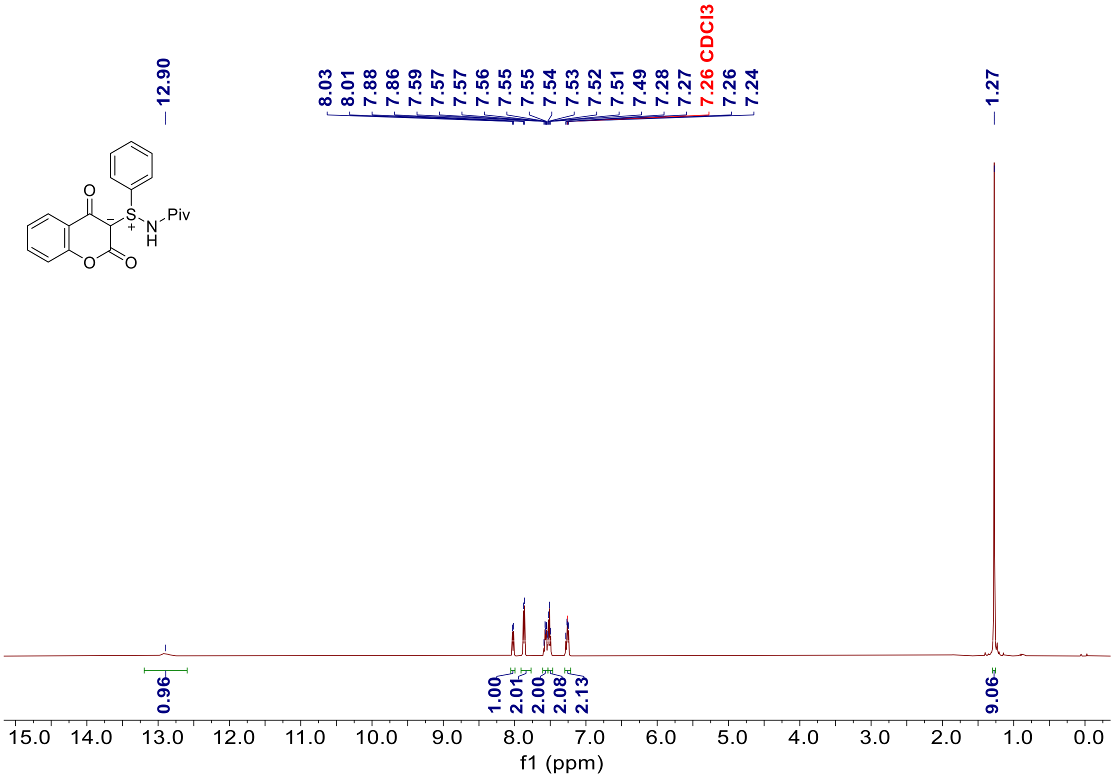

^{1}H NMR spectrum for product **7a**

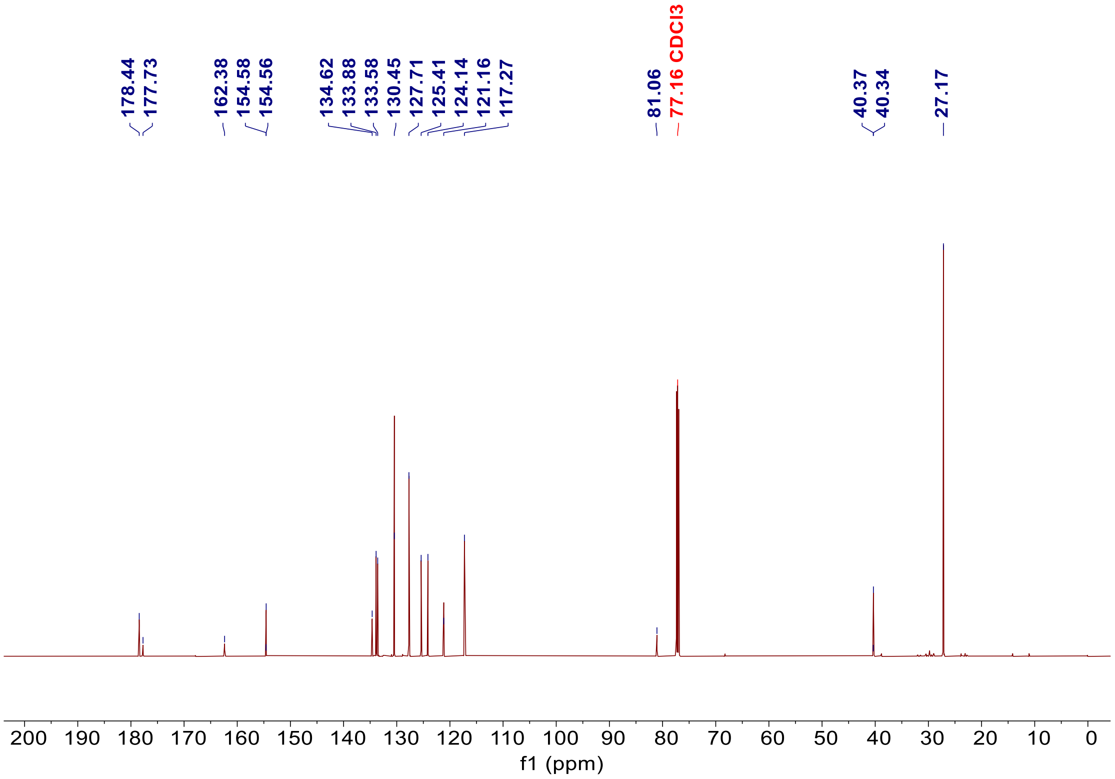

^{13}C NMR spectrum for product **7a**

(*Z*)-*N*-(phenyl(1,3,4-trioxo-3,4-dihydronaphthalen-2(1*H*)-ylidene)-λ^4-sulfaneyl)pivalamide (7b)

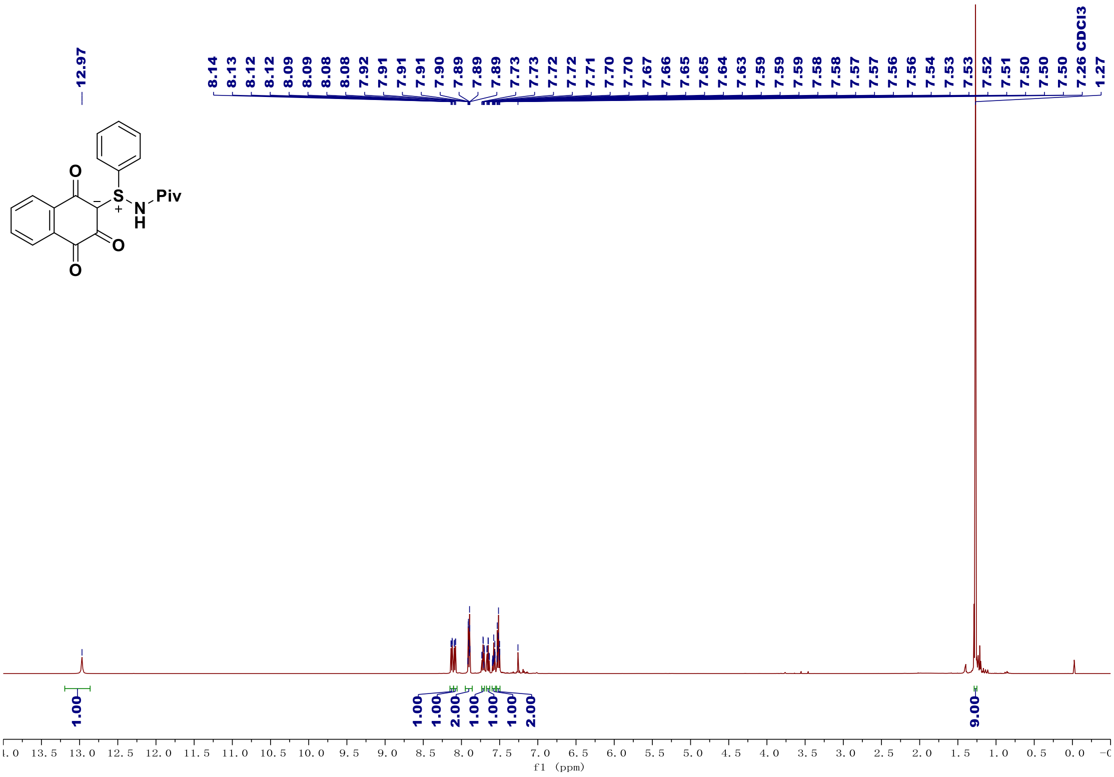

^{1}H NMR spectrum for product **7b**

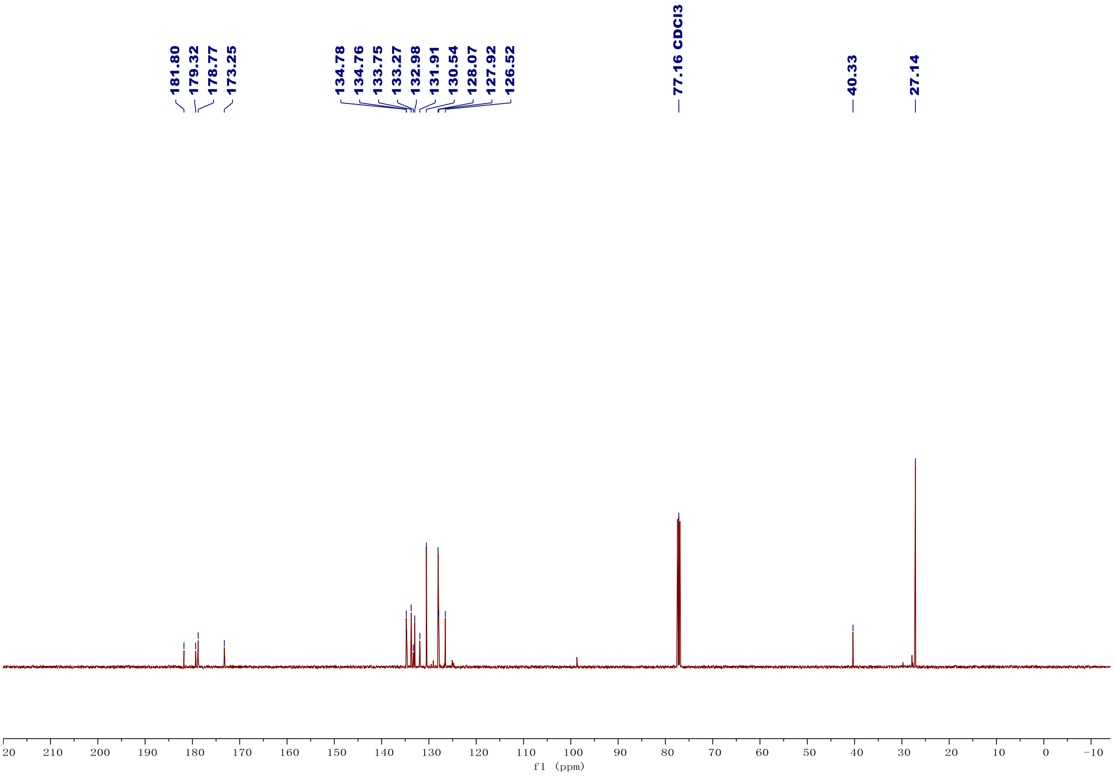

^{13}C NMR spectrum for product **7b**

(*Z*)-*N*-((1-methyl-2,4-dioxo-1,4-dihydroquinolin-3(2*H*)-ylidene)(phenyl)-λ[4]-sulfaneyl)pivalamide (7c)

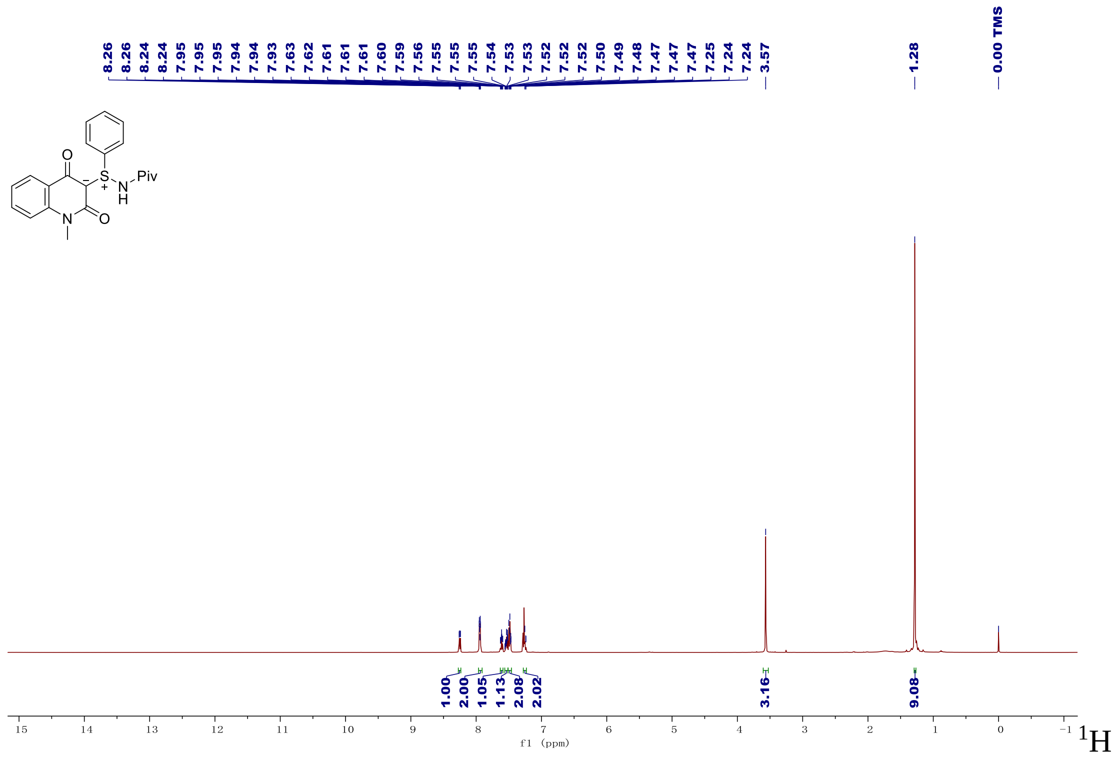

NMR spectrum for product **7c**

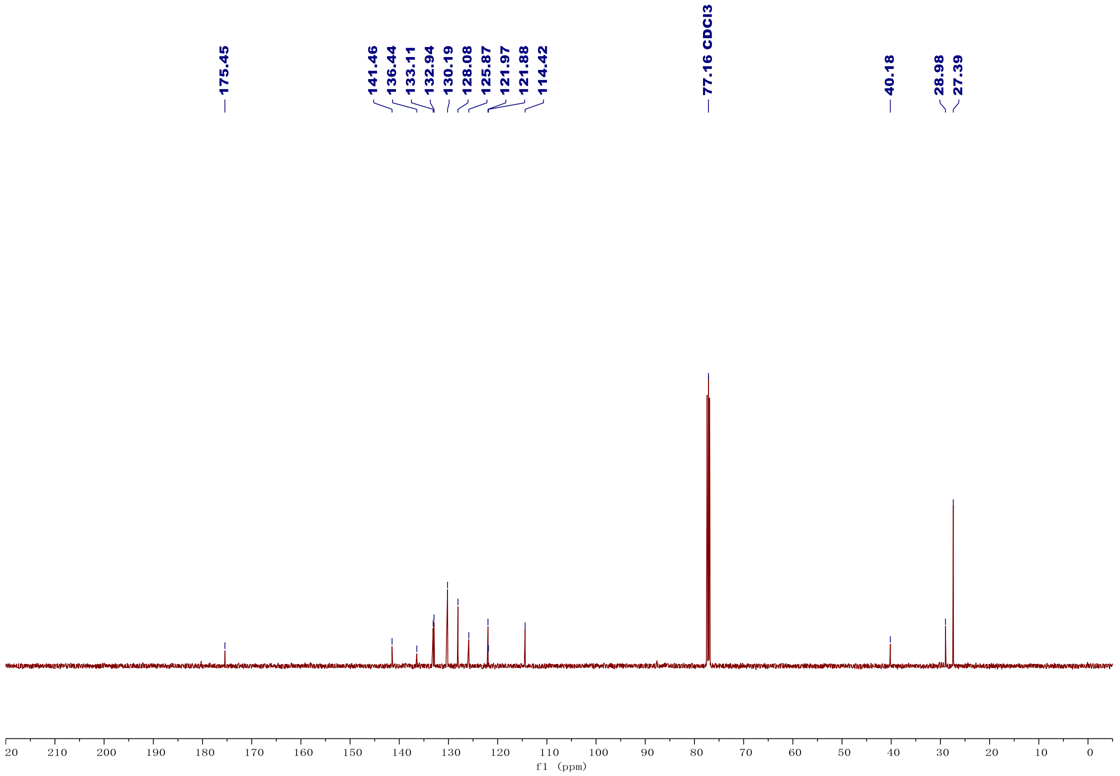

^{13}C NMR spectrum for product **7c**

(*E*)-*N*-((2,4-dioxo-2H-pyrido[1,2-a]pyrimidin-3(4*H*)-ylidene)(phenyl)-λ⁴-sulfaneyl)pivalamide (7d)

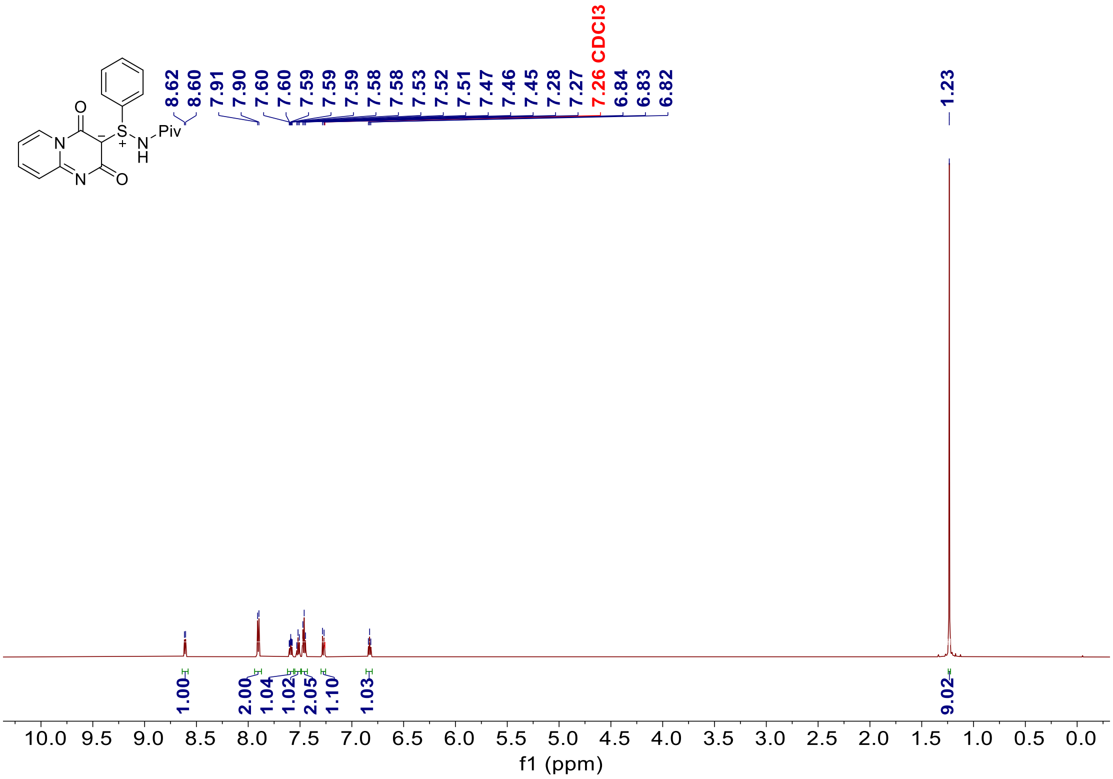

^{1}H NMR spectrum for product **7d**

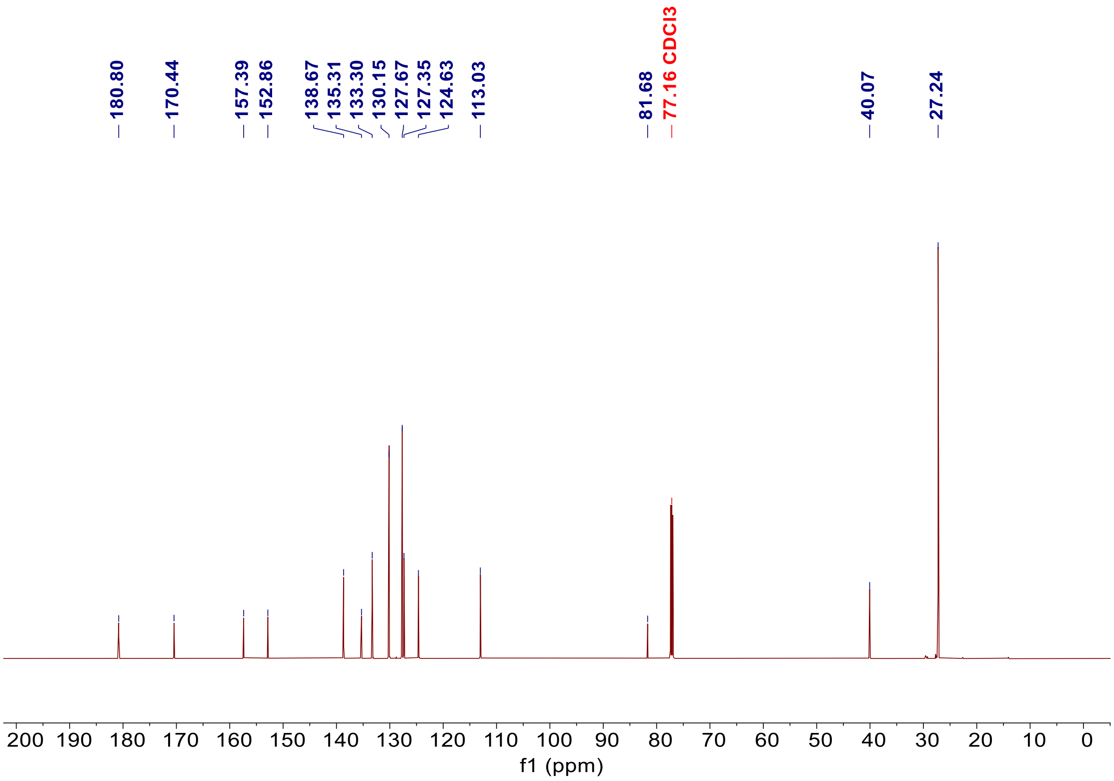

^{13}C NMR spectrum for product **7d**

(*Z*)-*N*-((6-methyl-2,4-dioxo-2H-pyran-3(4*H*)-ylidene)(phenyl)-λ⁴-sulfaneyl)pivalamide (7e)

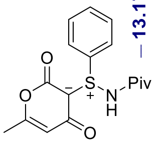

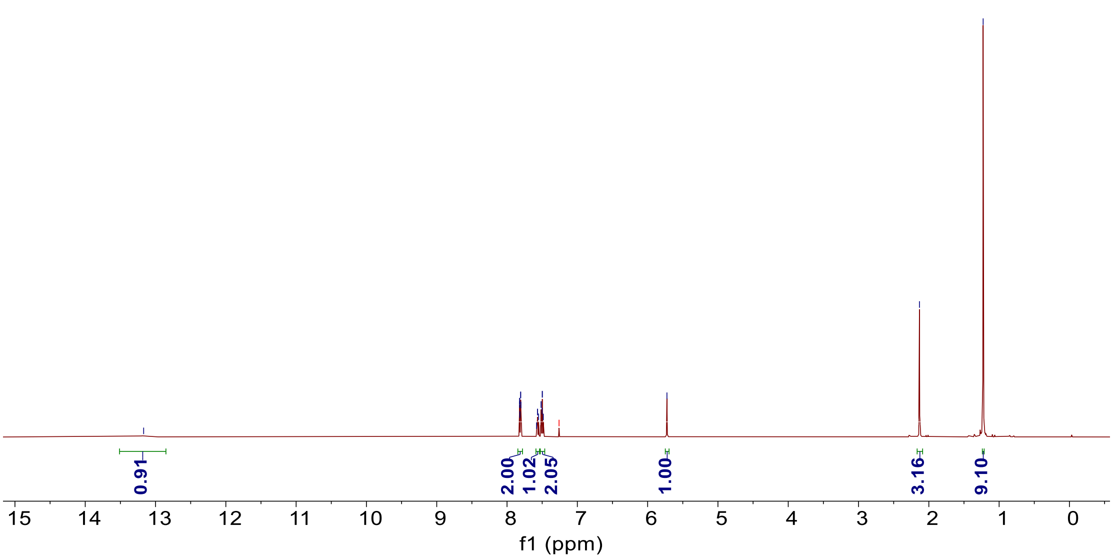

^{1}H NMR spectrum for product **7e**

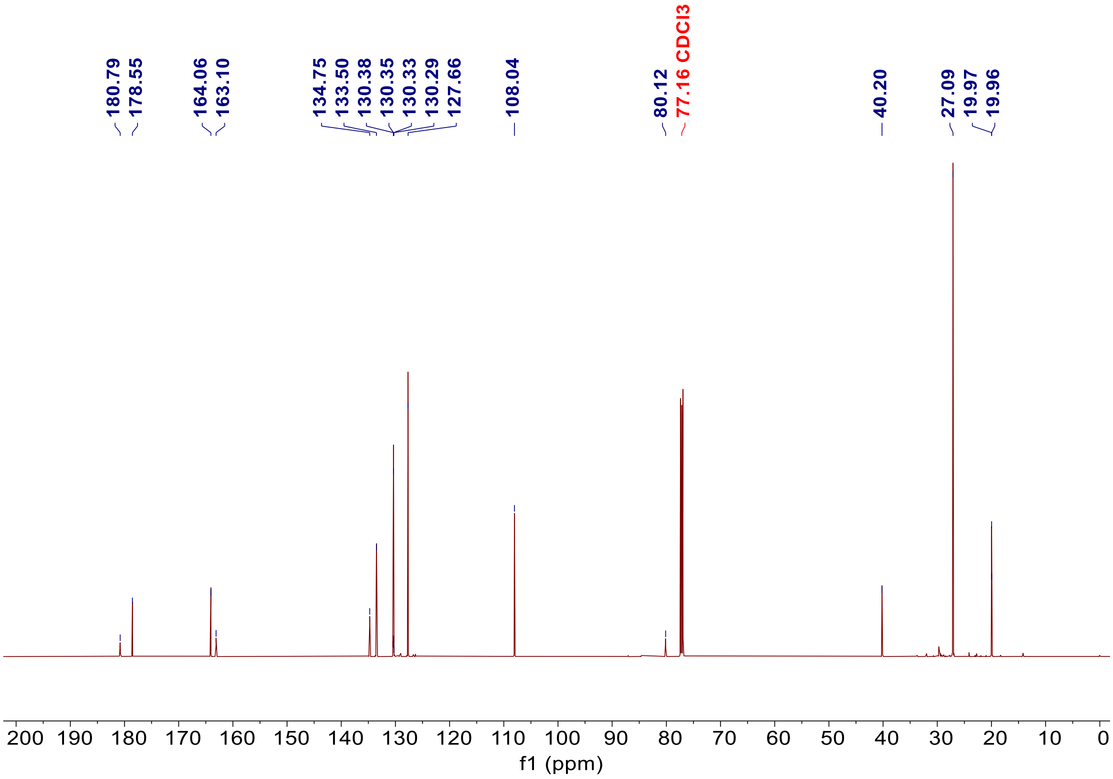

^{13}C NMR spectrum for product **7e**

(*E*)-*N*-((6-methyl-2,4-dioxo-1,4-dihydropyridin-3(2*H*)-ylidene)(phenyl)-λ[4]-sulfaneyl)pivalamide (7f)

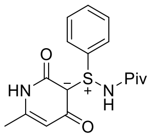

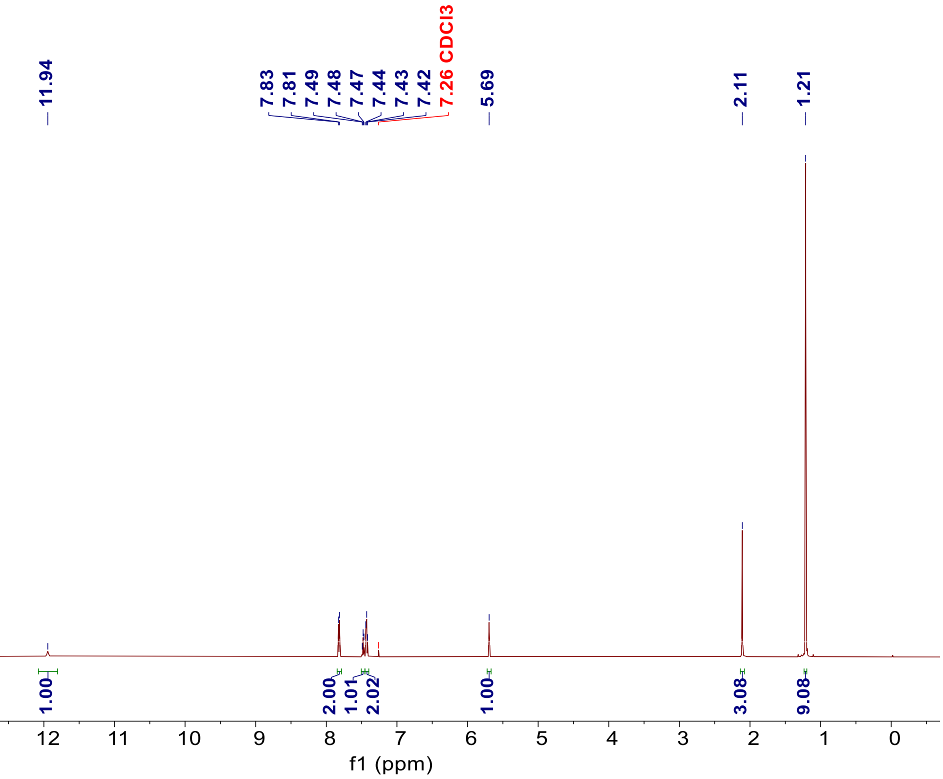

^{1}H NMR spectrum for product **7f**

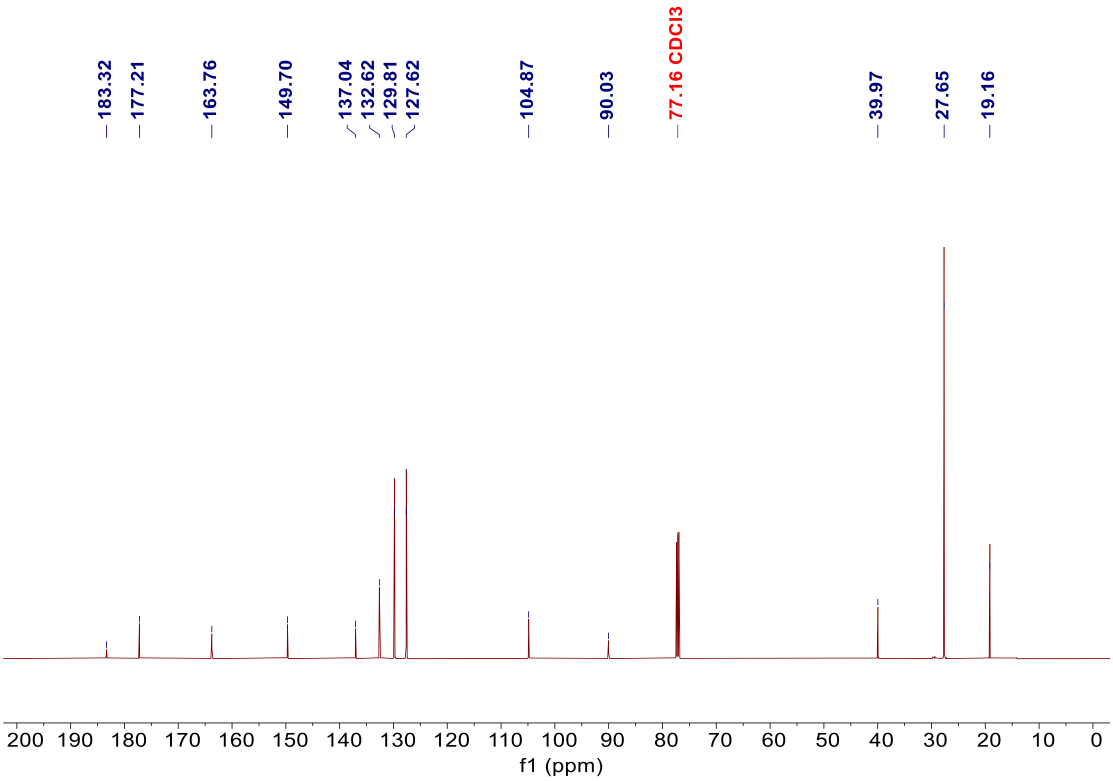

^{13}C NMR spectrum for product **7f**

N-((2,6-dioxo-4-phenylcyclohexylidene)(phenyl)-λ⁴-sulfaneyl)pivalamide (7g)

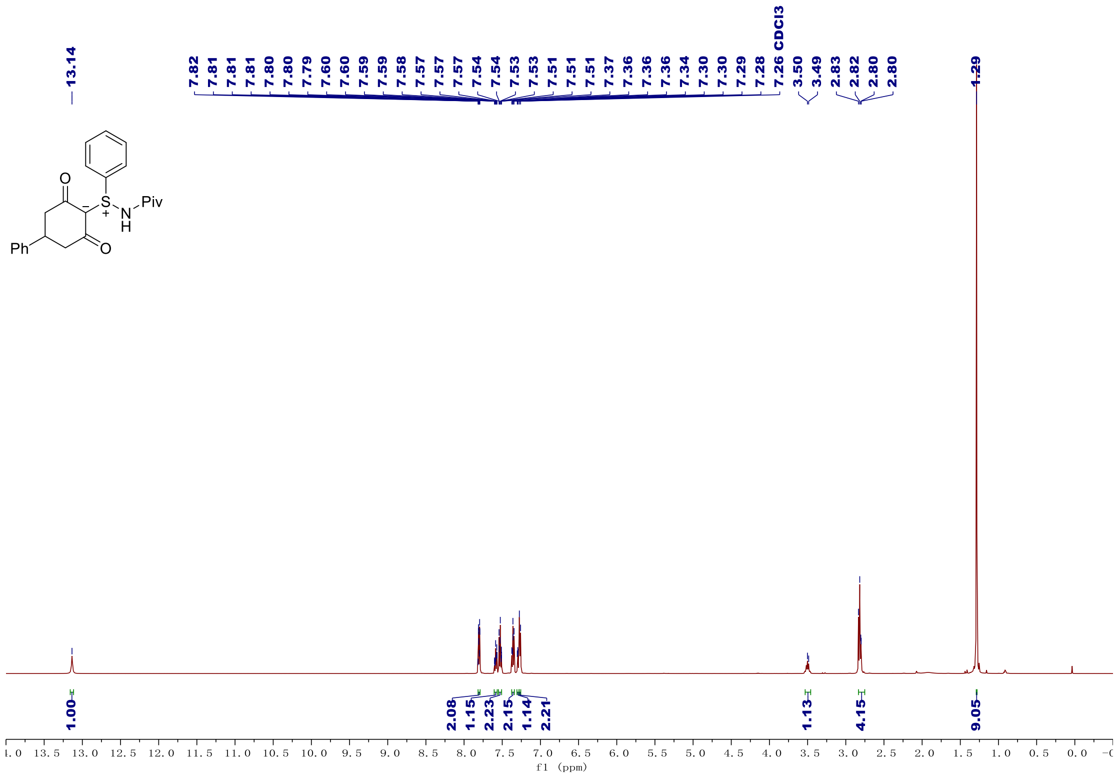

^{1}H NMR spectrum for product **7g**

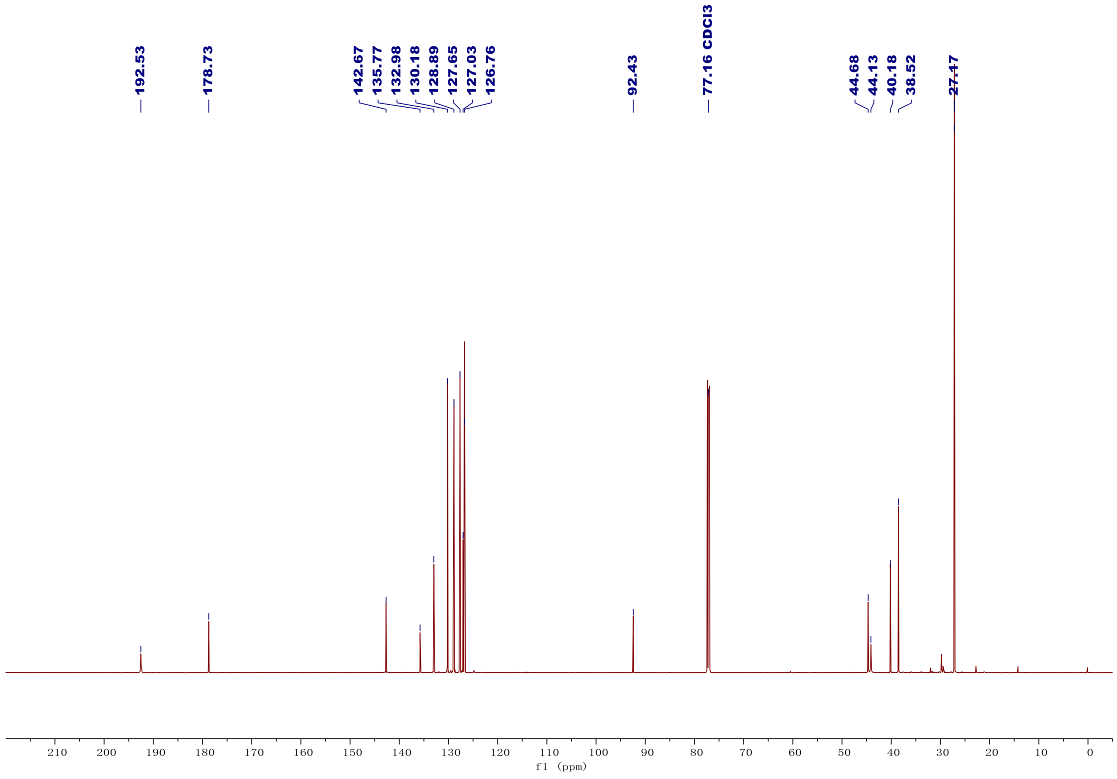

^{13}C NMR spectrum for product **7g**

tert-butyl

***3,5-dioxo-4-(phenyl(pivalamido)-λ⁴-sulfaneylidene)piperidine-1-carboxylate* (7h)**

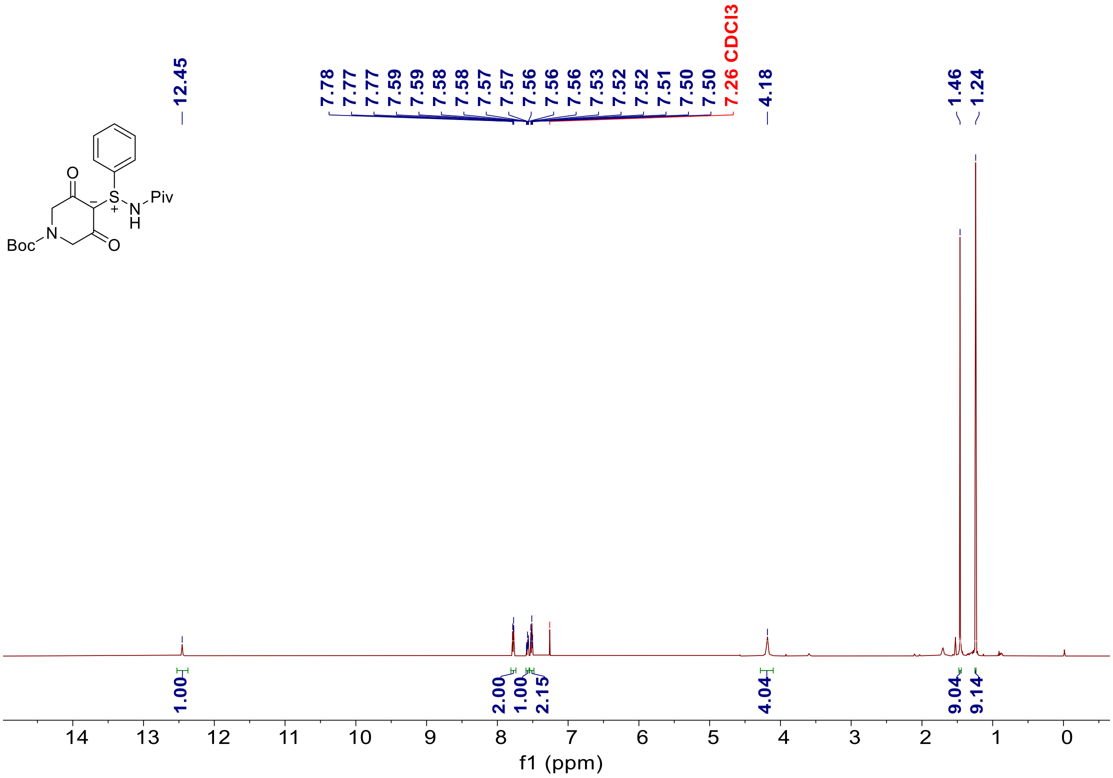

^{1}H NMR spectrum for product **7h**

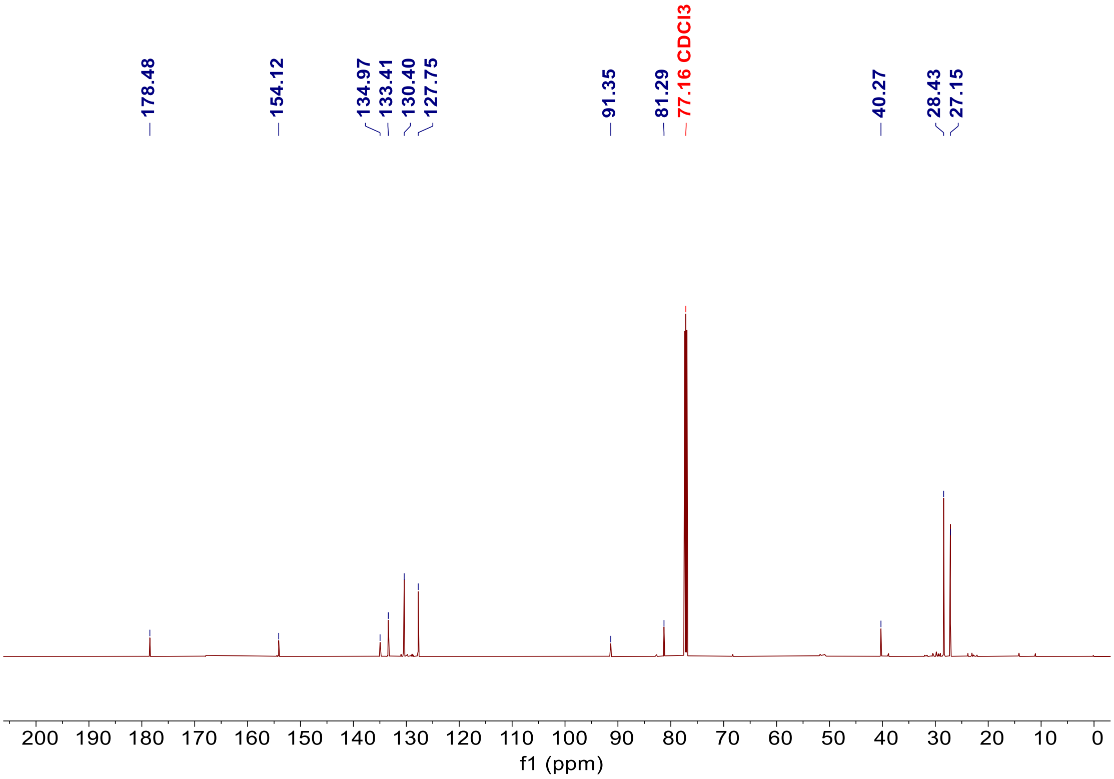

^{13}C NMR spectrum for product **7h**

N-((2,5-dioxocyclopentylidene)(phenyl)-λ⁴-sulfaneyl)pivalamide (7i)

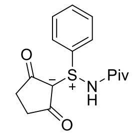

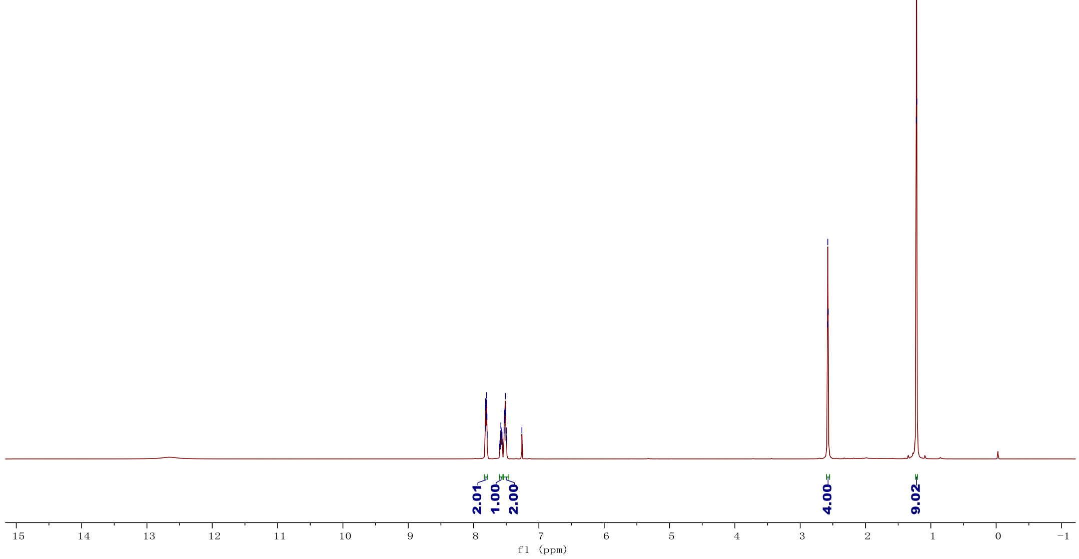

^{1}H NMR spectrum for product **7i**

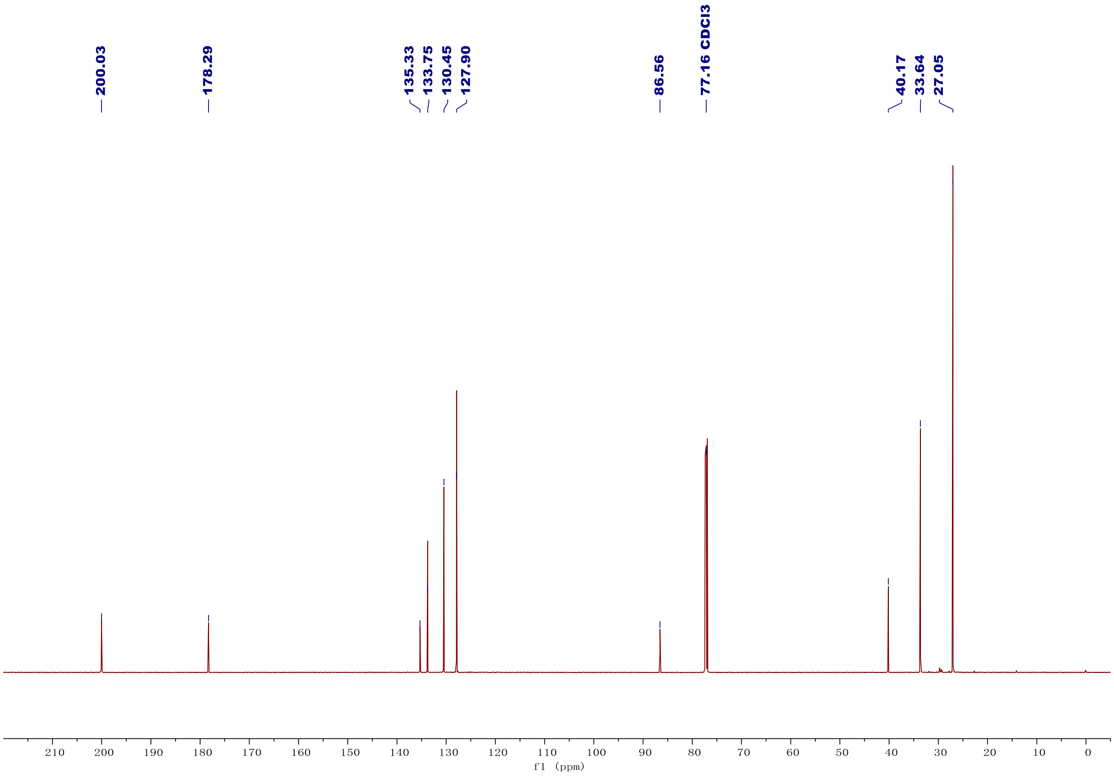

^{13}C NMR spectrum for product **7i**

N-((2,6-dioxocyclohexylidene)(phenyl)- λ⁴-sulfaneyl)pivalamide **(7j)**

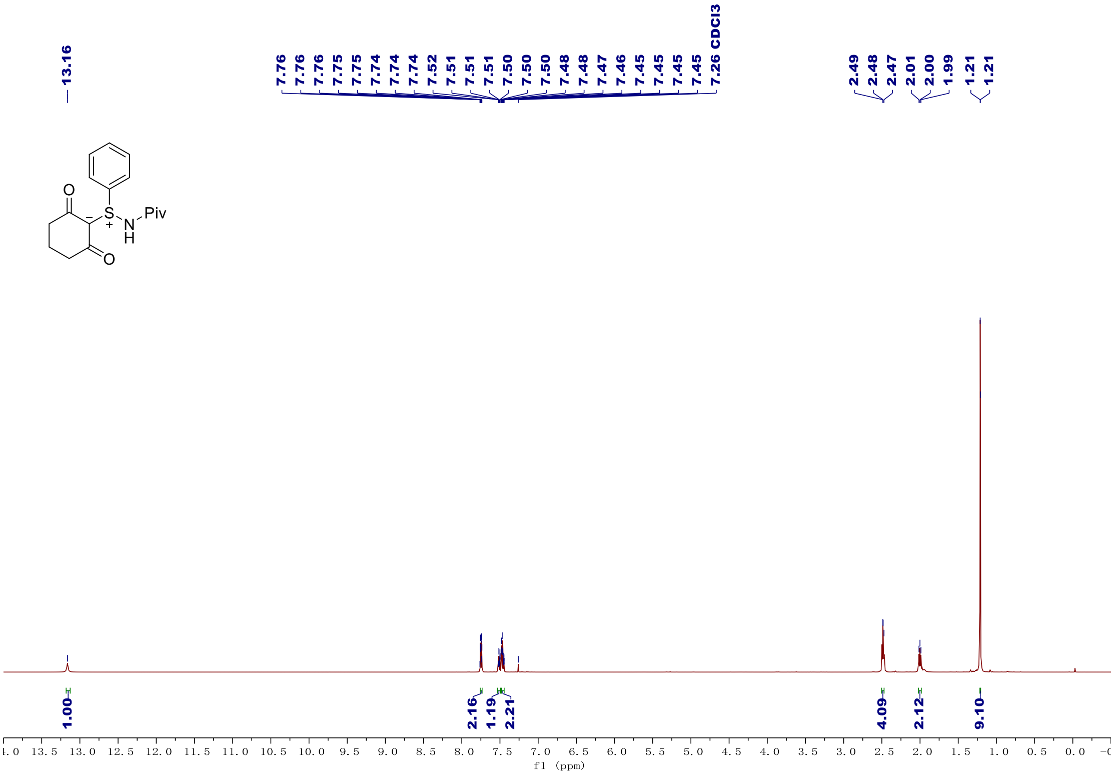

^{1}H NMR spectrum for product **7j**

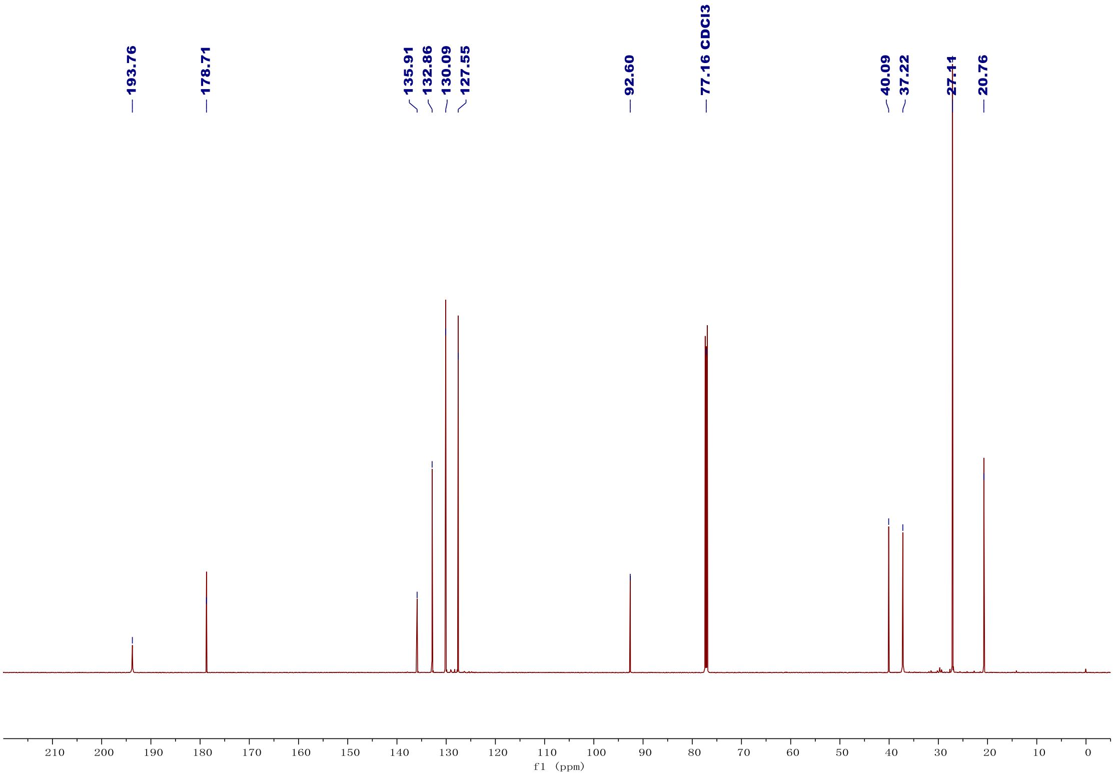

^{13}C NMR spectrum for product **7j**

***(E)-N-((5-hydroxy-3-oxo-3,6-dihydro-2H-pyran-4-yl)(phenyl)-l4-sulfaneylidene)pivalamide* (7k)**

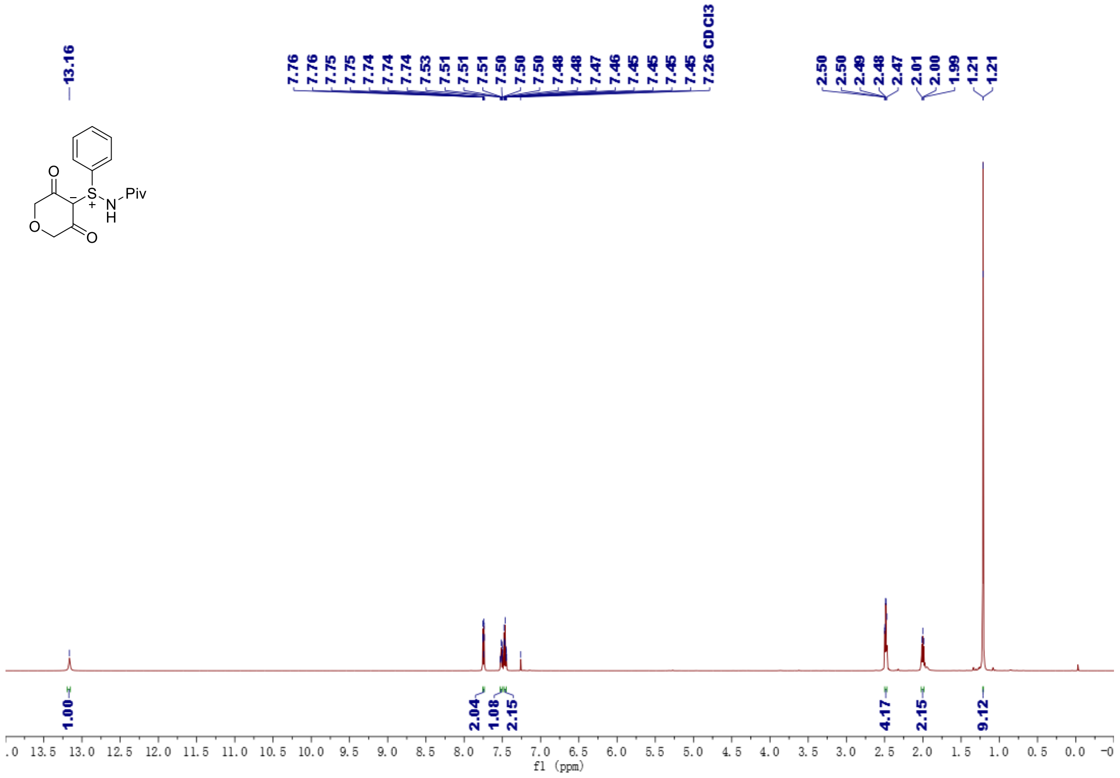

^{1}H NMR spectrum for product **7k**

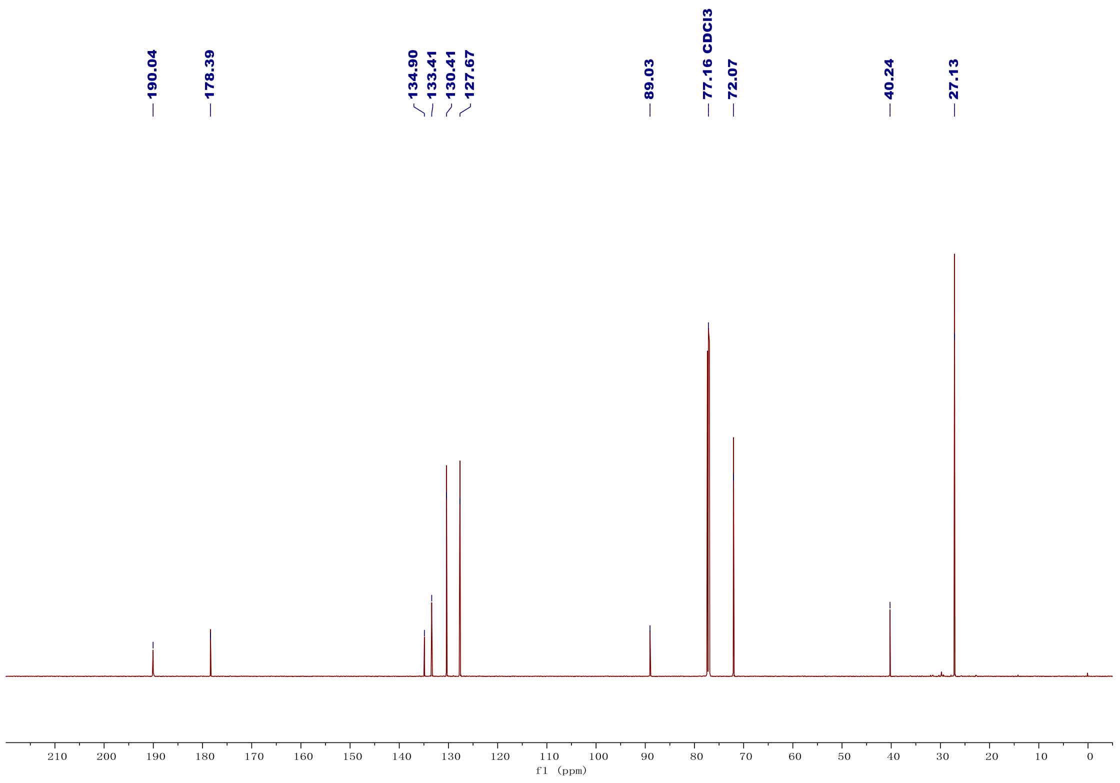

^{13}C NMR spectrum for product **7k**

***dimethyl 2-(phenyl(pivalamido)-λ⁴-sulfaneylidene)malonate* (7l)**

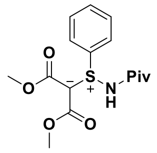

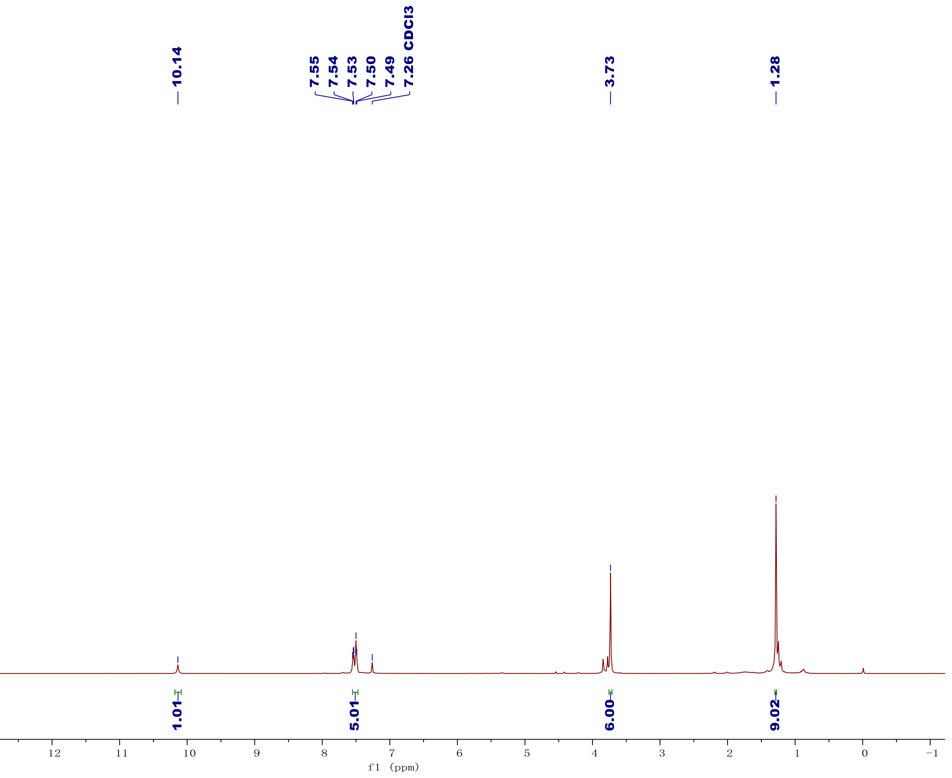

^{1}H NMR spectrum for product **7l**

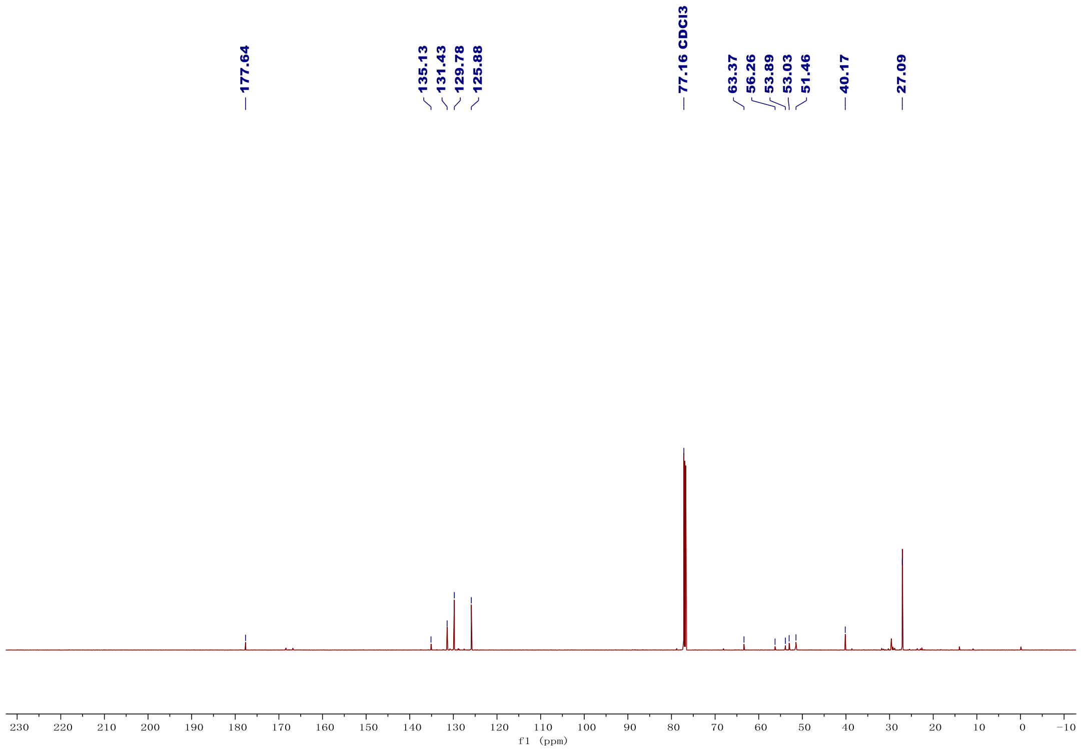

^{13}C NMR spectrum for product **7l**

(Z)-N-((2,4-dioxochroman-3-ylidene)(4-(trifluoromethyl)phenyl)-λ⁴-sulfaneyl)pivalamide **(7m)**

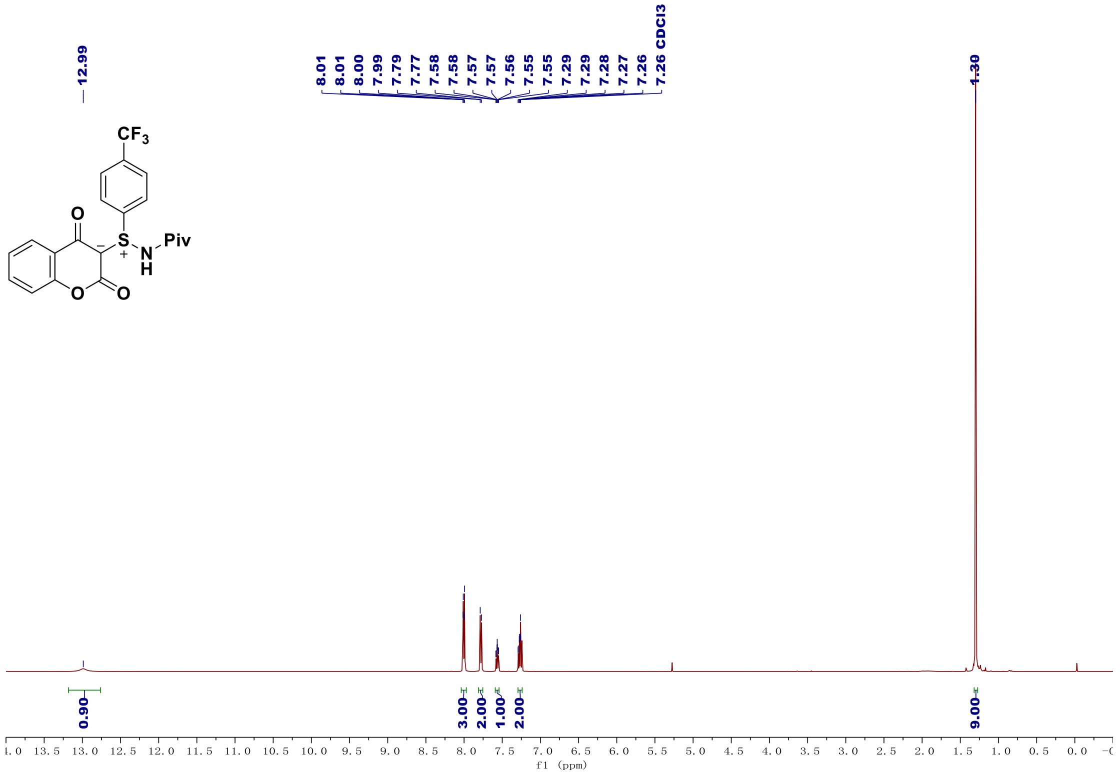

^{1}H NMR spectrum for product **7m**

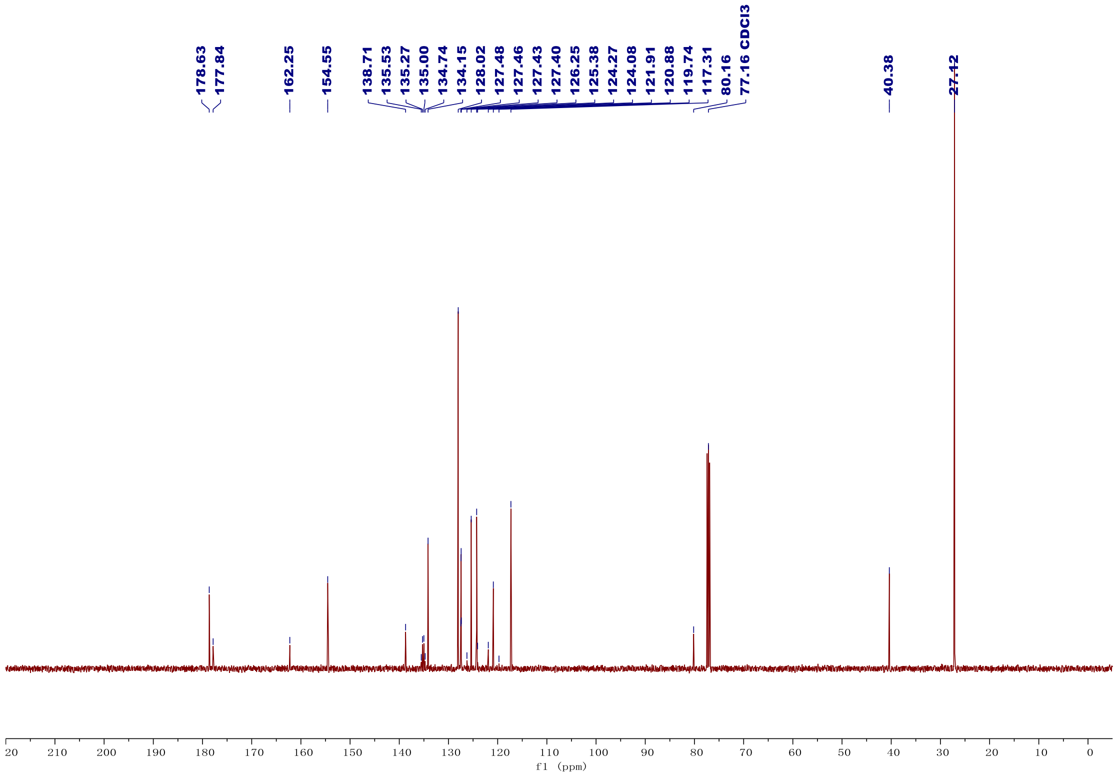

^{13}C NMR spectrum for product **7m**

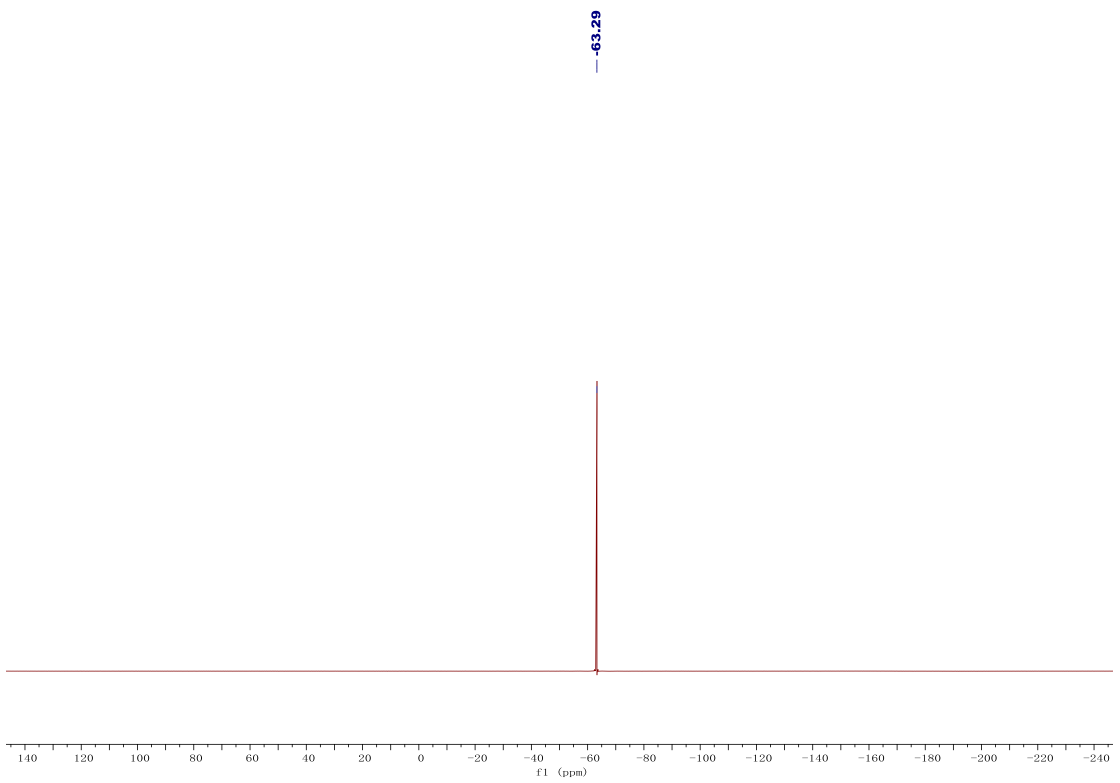

^{19}F NMR spectrum for product **7m**

(Z)-N-((4-chlorophenyl)(2,4-dioxochroman-3-ylidene)-λ⁴-sulfaneyl)pivalamide **(7n)**

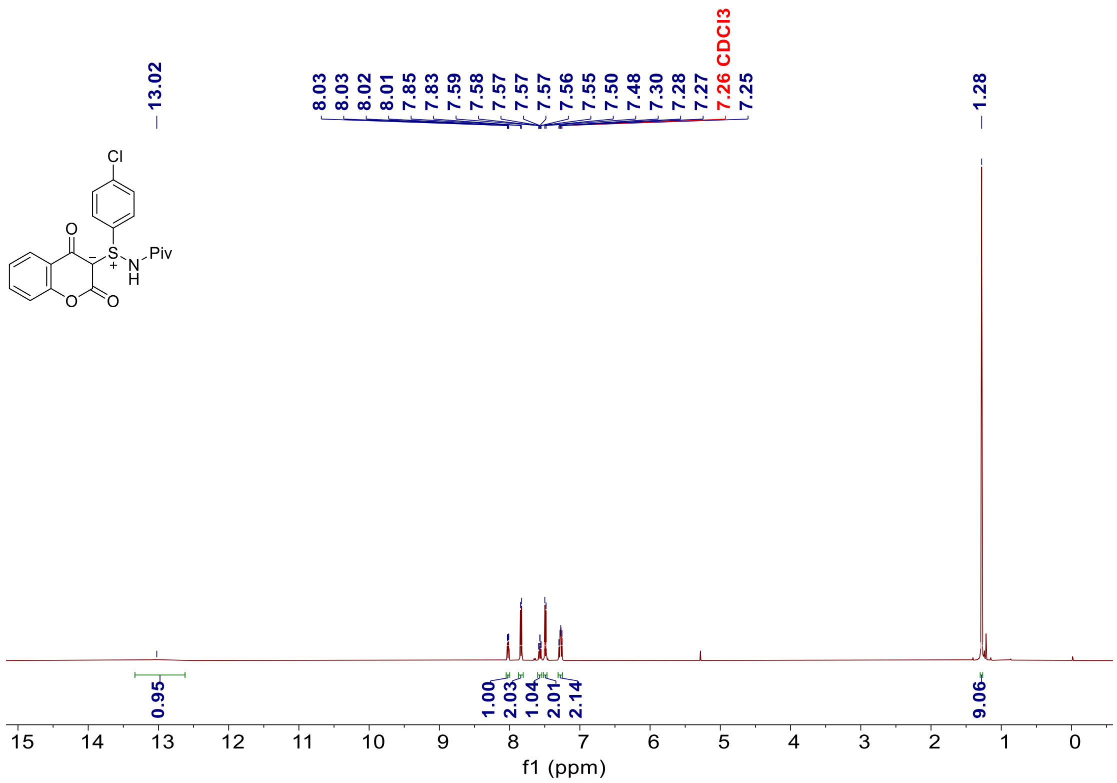

^{1}H NMR spectrum for product **7n**

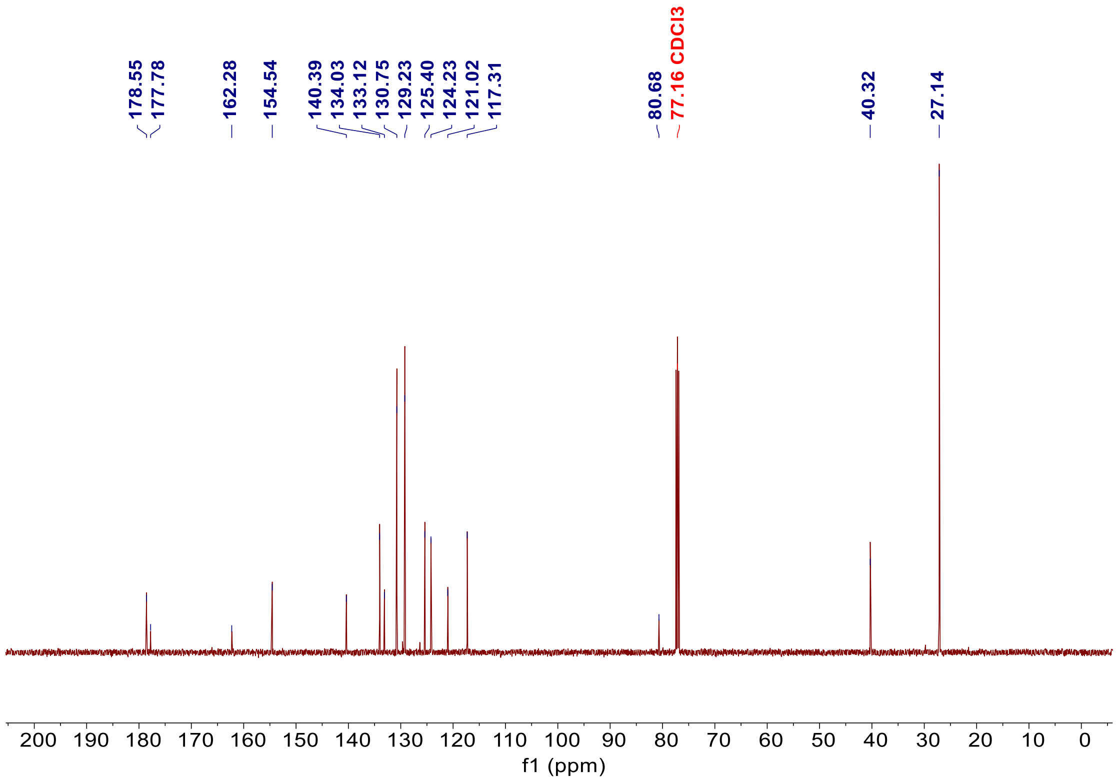

^{13}C NMR spectrum for product **7n**

***(Z)-N-((2,4-dioxochroman-3-ylidene)(4-methoxyphenyl)-λ^4-sulfaneyl)pivalamide* (7o)**

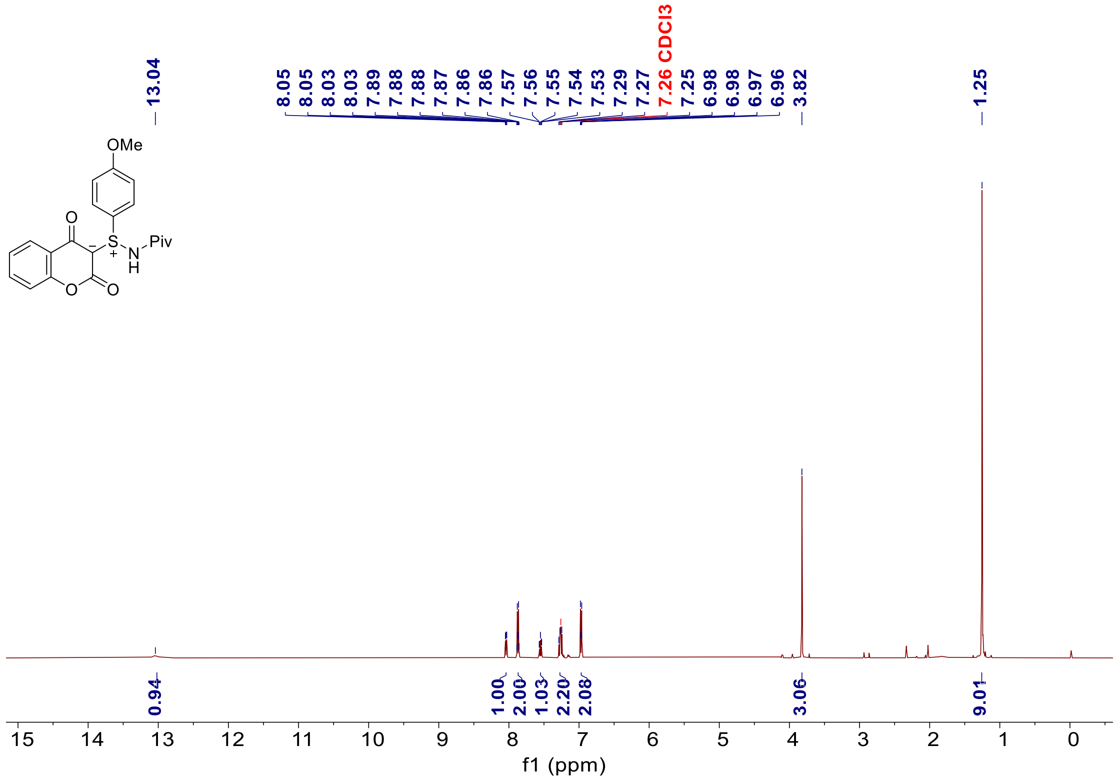

^{1}H NMR spectrum for product **7o**

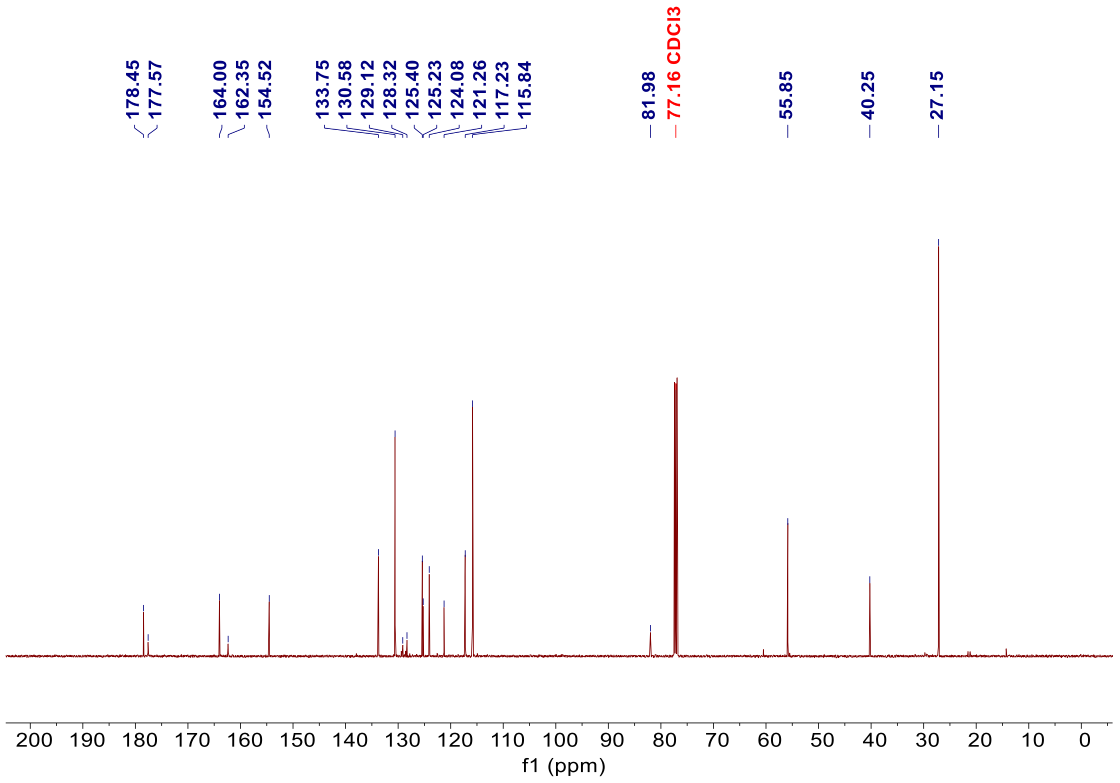

^{13}C NMR spectrum for product **7o**

***(Z)-N-((2,4-dioxochroman-3-ylidene)(thiophen-2-yl)-λ⁴-sulfaneyl)pivalamide* (7p)**

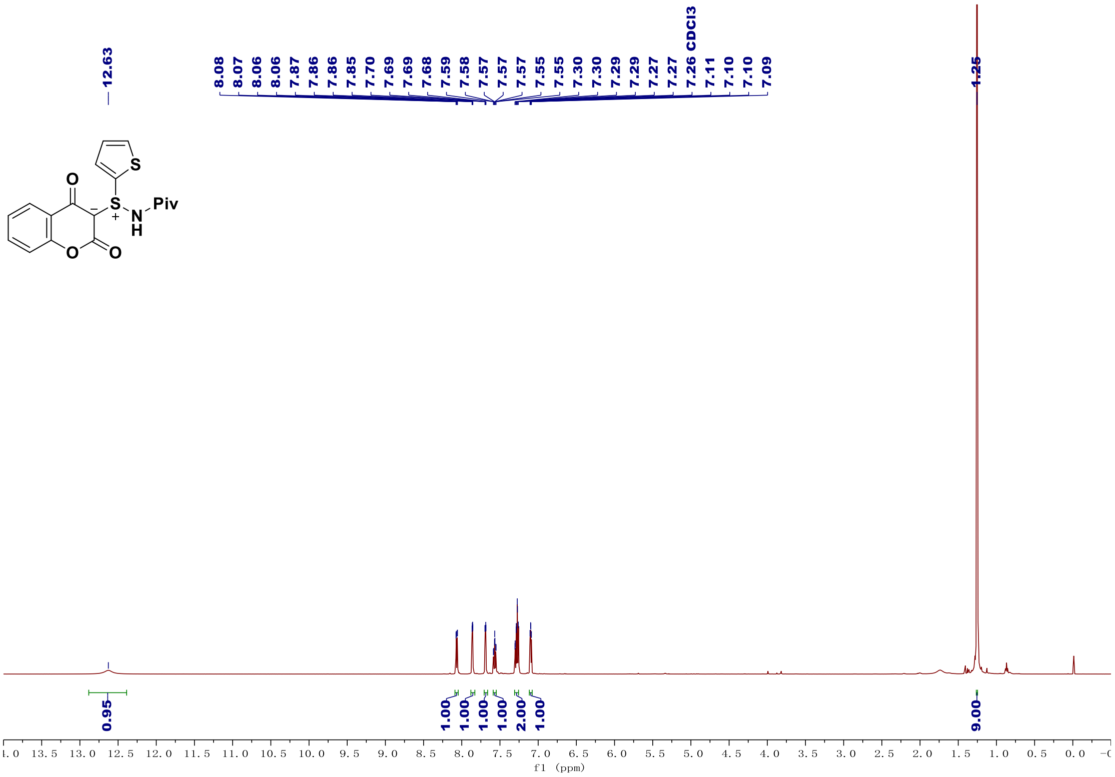

^{1}H NMR spectrum for product **7p**

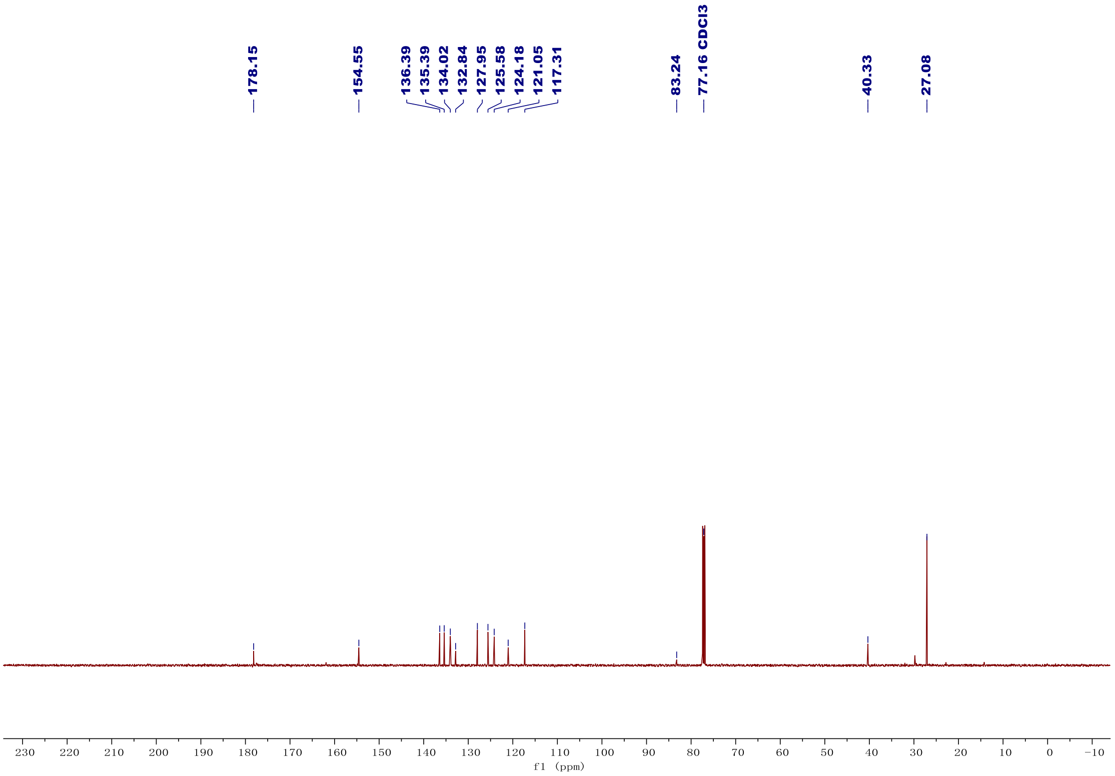

^{13}C NMR spectrum for product **7p**

(Z)-N-((2,4-dioxochroman-3-ylidene)(phenyl)-λ⁴-sulfaneyl)acetamide **(7q)**

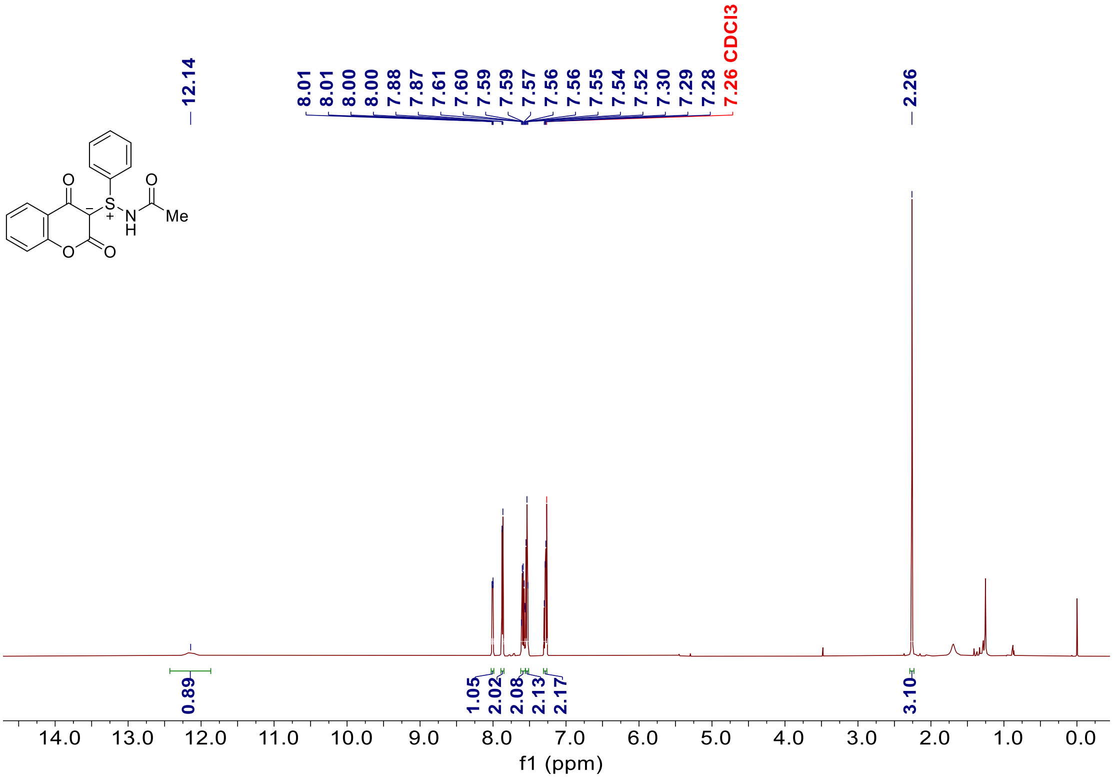

^{1}H NMR spectrum for product **7q**

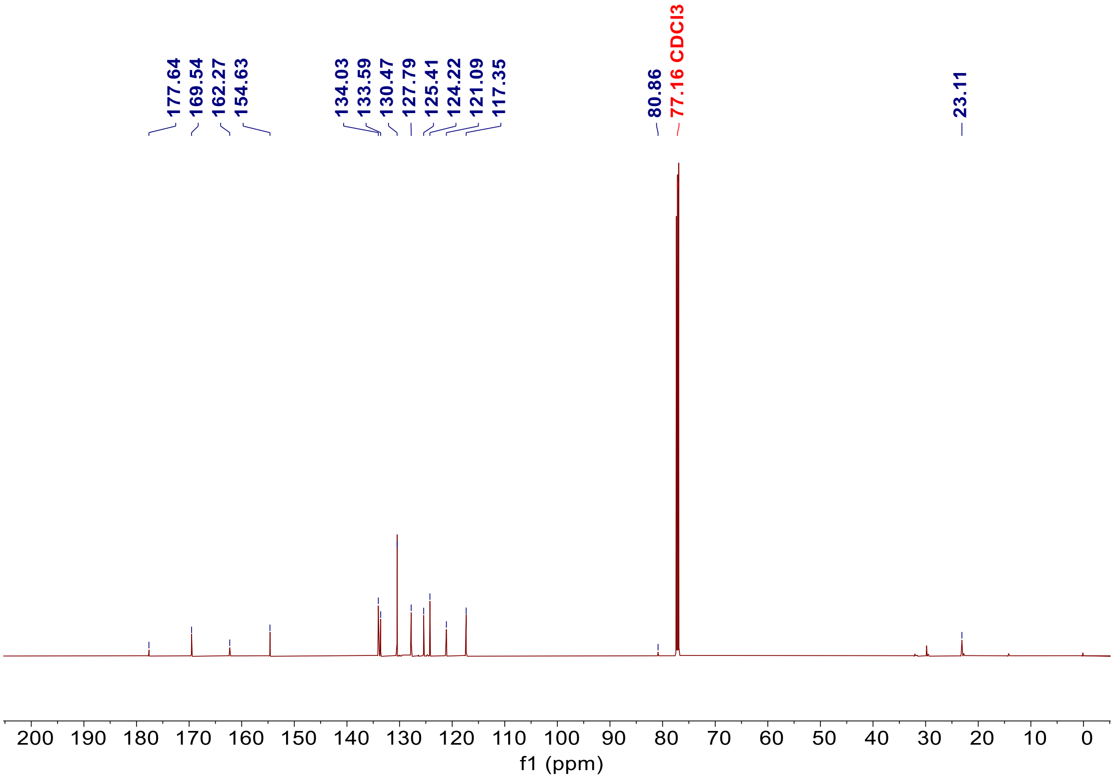

^{13}C NMR spectrum for product **7q**

***(Z)-N-((2,4-dioxochroman-3-ylidene)(phenyl)-λ⁴-sulfaneyl)benzamide* (7r)**

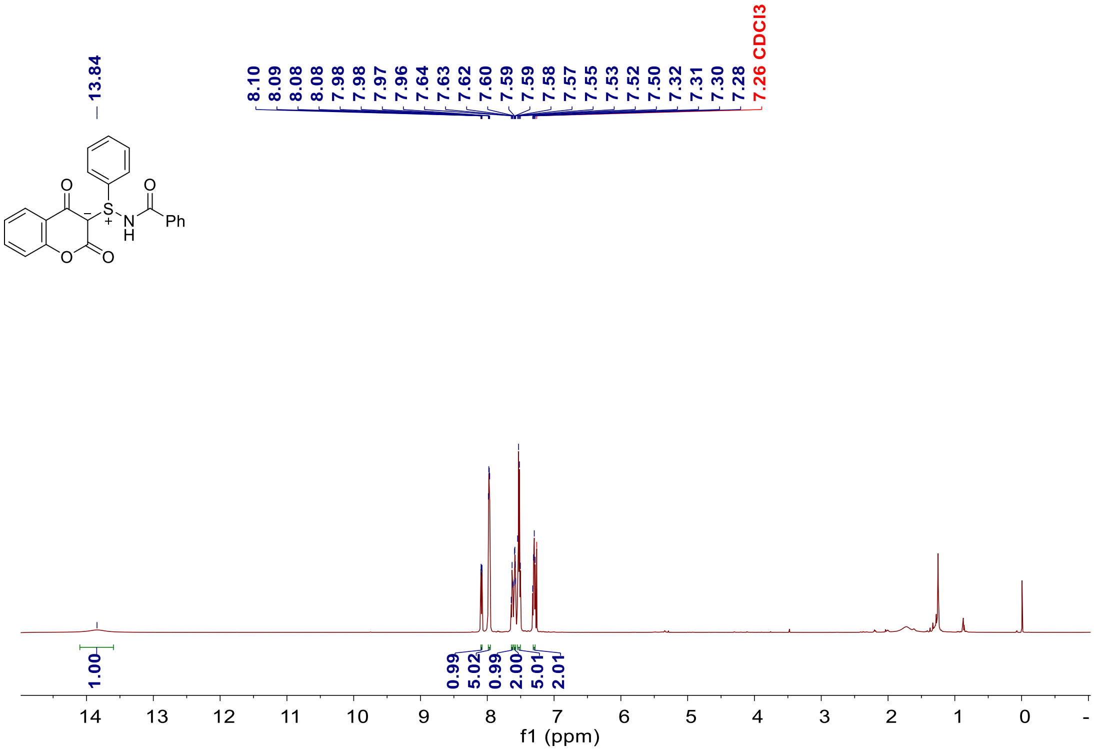

^{1}H NMR spectrum for product **7r**

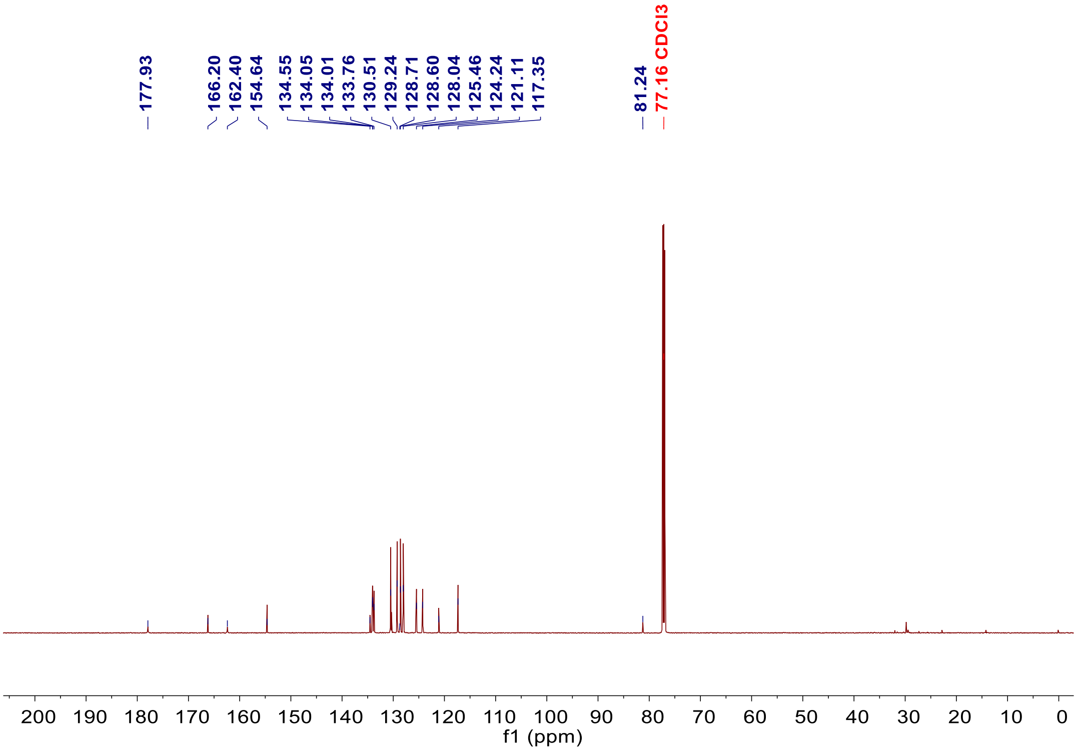

^{13}C NMR spectrum for product **7r**

2-(2-(phenylthio)pent-4-enoyl)phenyl acetate (8a)

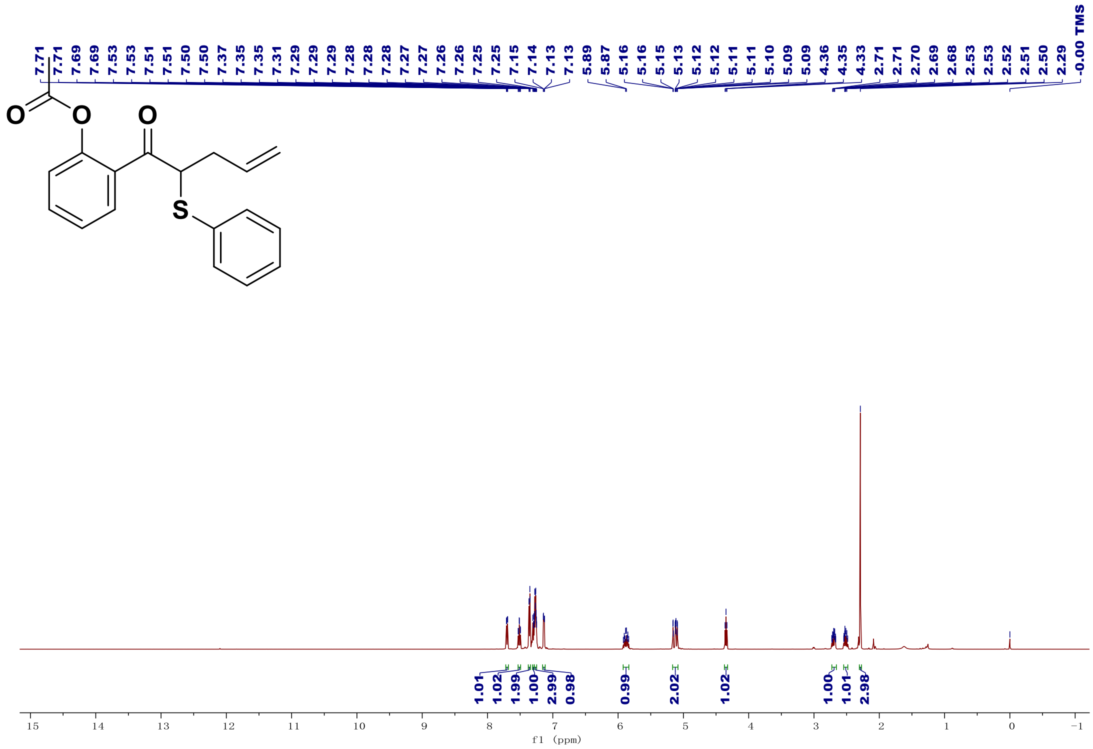

^{1}H NMR spectrum for product **8a**

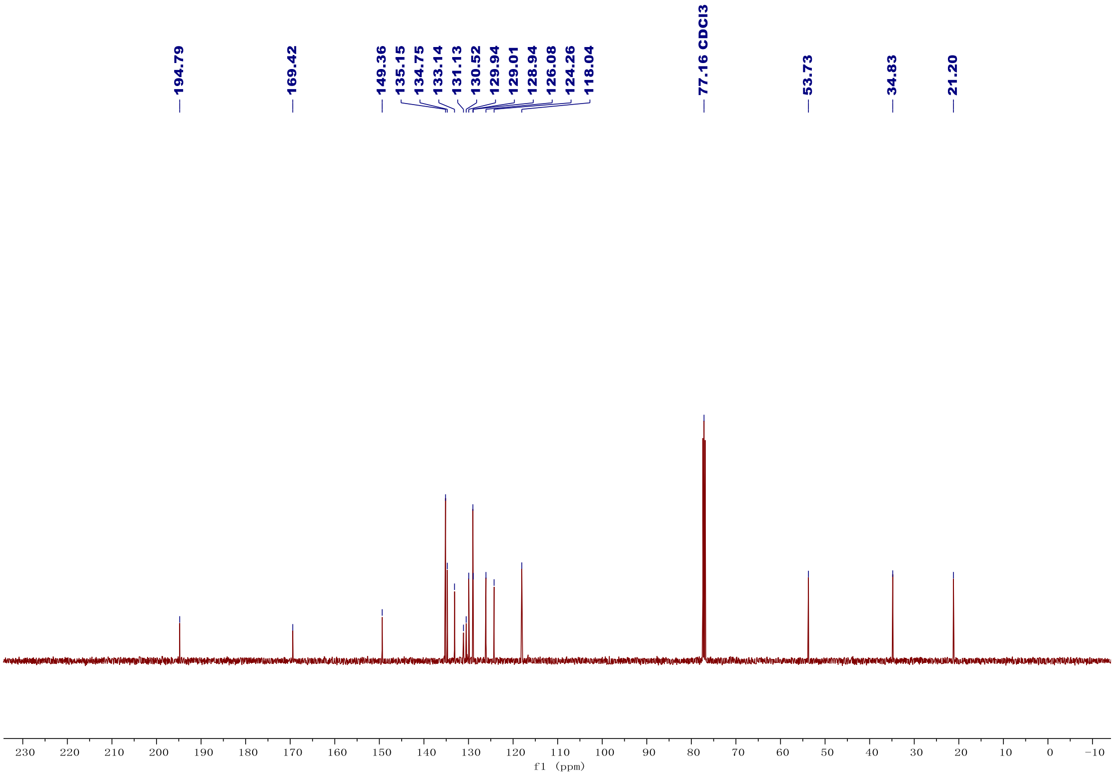

^{13}C NMR spectrum for product **8a**

1-(2-(cyclopentyloxy)phenyl)-2-(phenylthio)pent-4-en-1-one (8b)

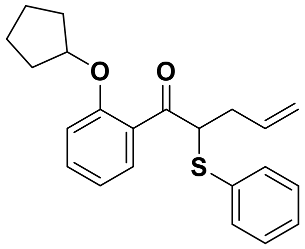

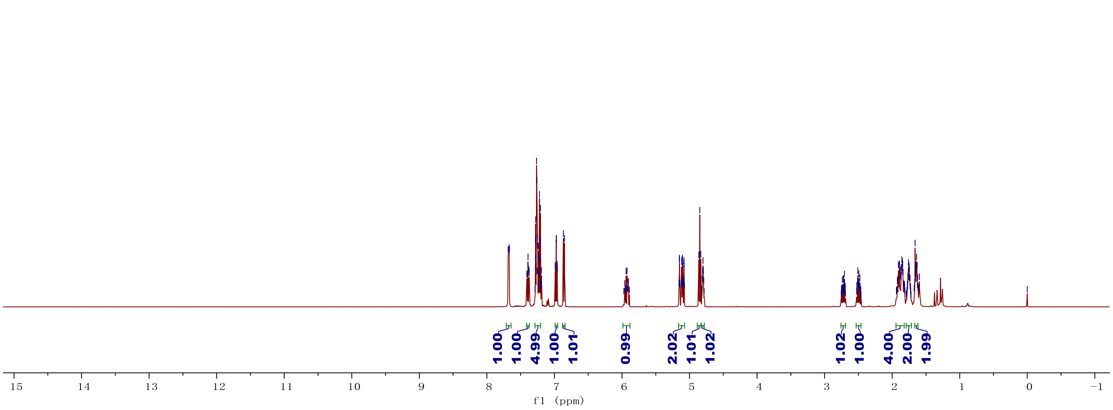

^{1}H NMR spectrum for product **8b**

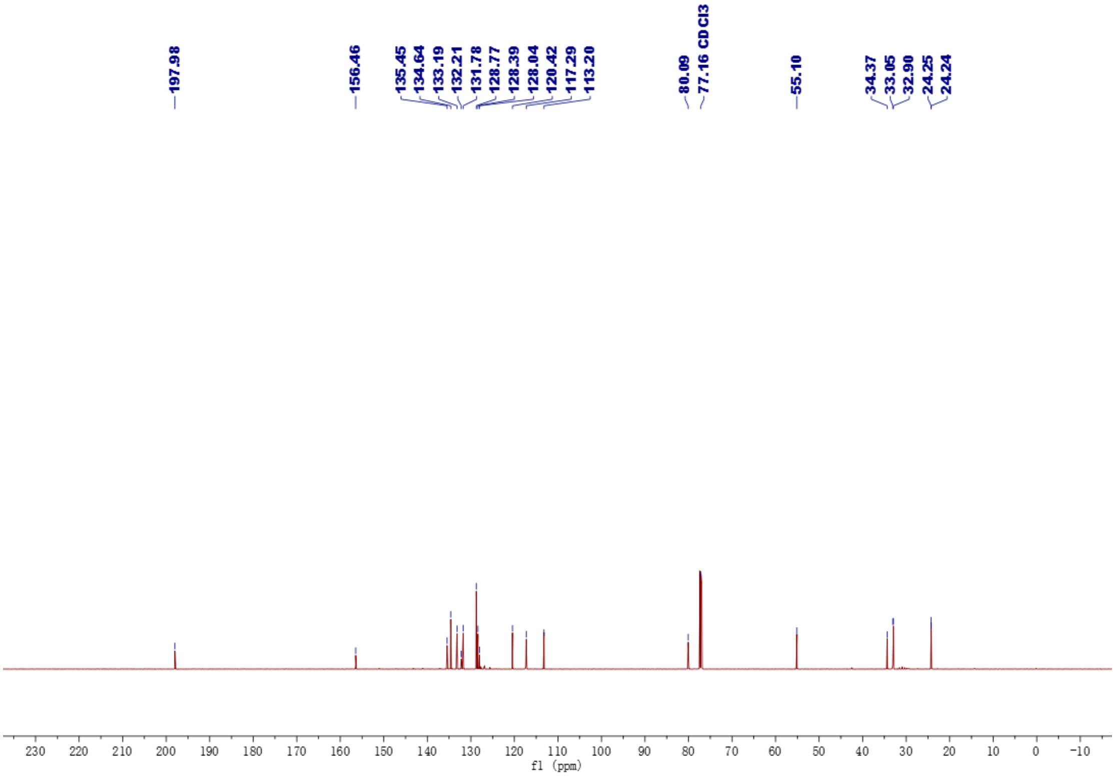

^{13}C NMR spectrum for product **8b**

1-(2-hydroxyphenyl)-2-(phenylsulfonyl)pent-4-en-1-one (8c)

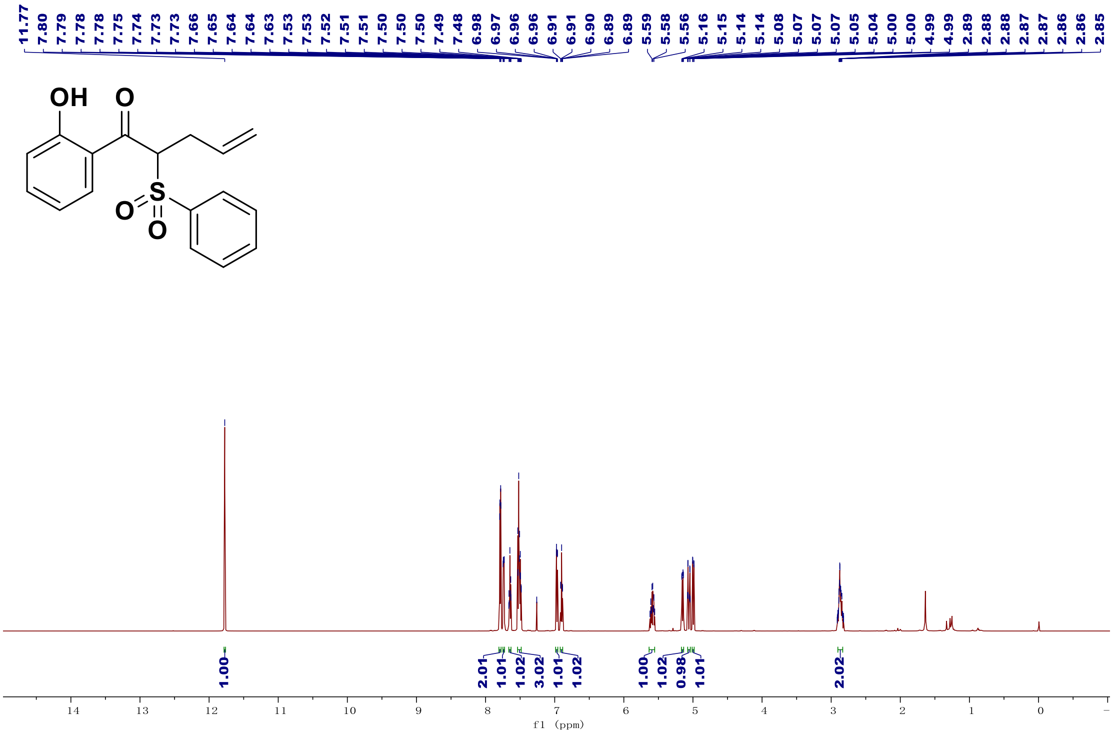

^{1}H NMR spectrum for product **8c**

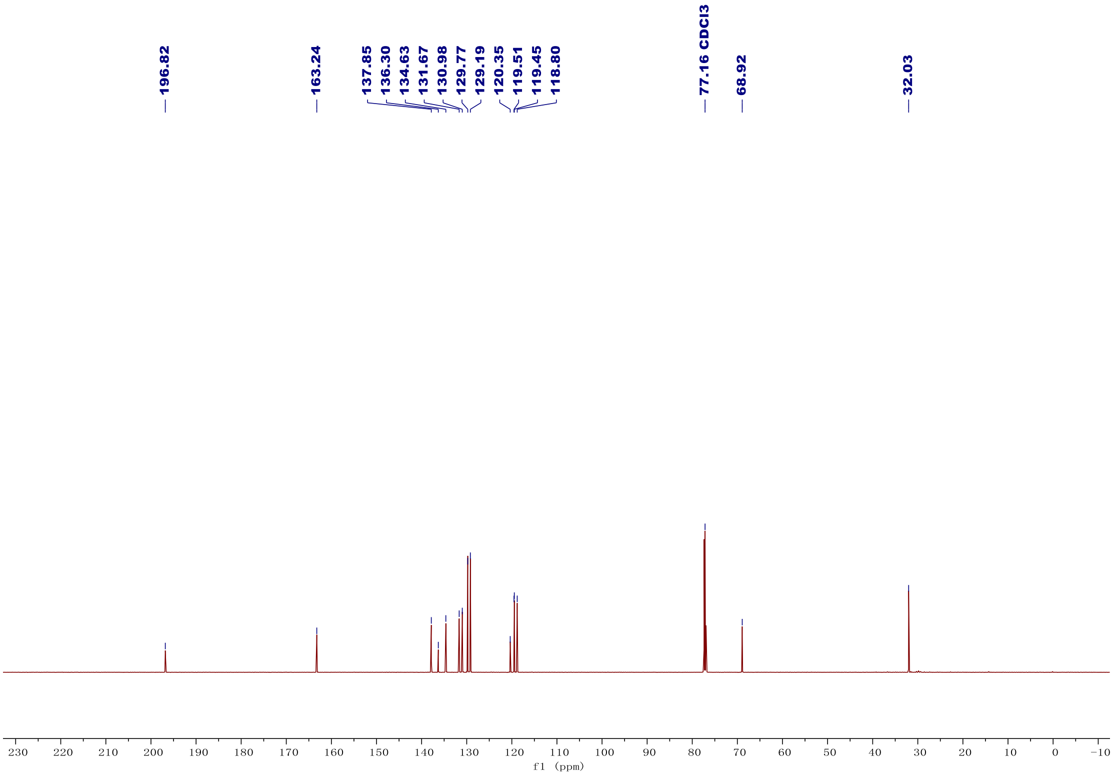

^{13}C NMR spectrum for product **8c**

1-(2-hydroxyphenyl)-3-(oxiran-2-yl)-2-(phenylsulfonyl)propan-1-one (8d)

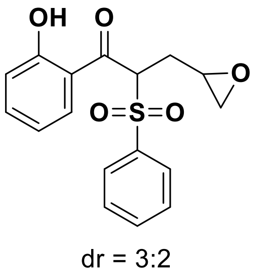

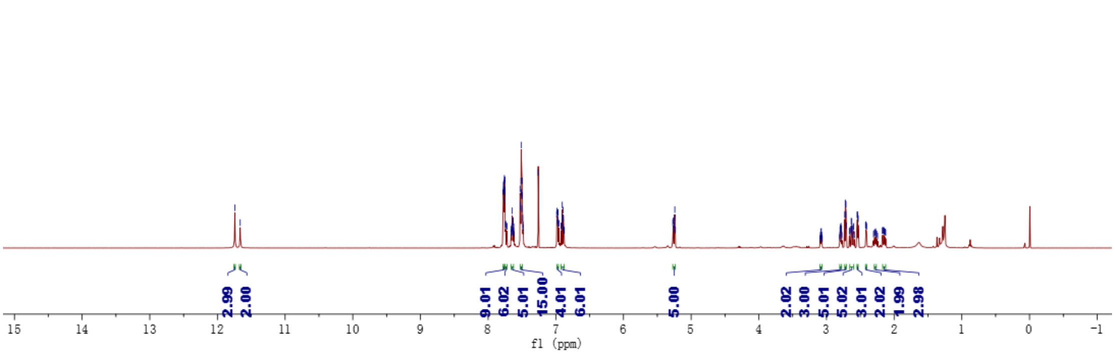

^{1}H NMR spectrum for product **8d**

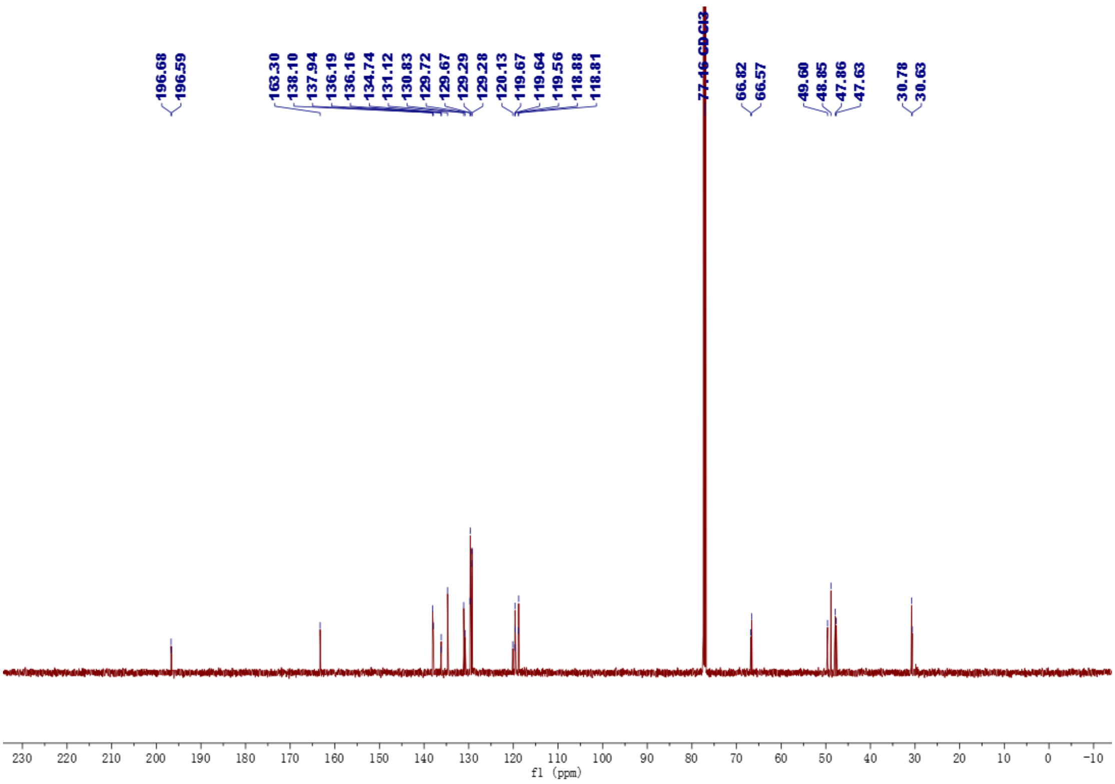

^{13}C NMR spectrum for product **8d**

Copies of HPLC Spectra for selected Compounds

HPLC analysis was conducted using MeOH/H_2O = 90/10 as eluent. The retention time is expressed in min at UV detection of 254 nm. HPLC analysis was performed on an XBridge®-C18 (4.6 × 50 mm, 3.5 μm) at 30 °C. Flow rate: 0.5 mL / min.

1-(2-hydroxy-4-methylphenyl)-2-(phenylthio)pent-4-en-1-one (**3c**)

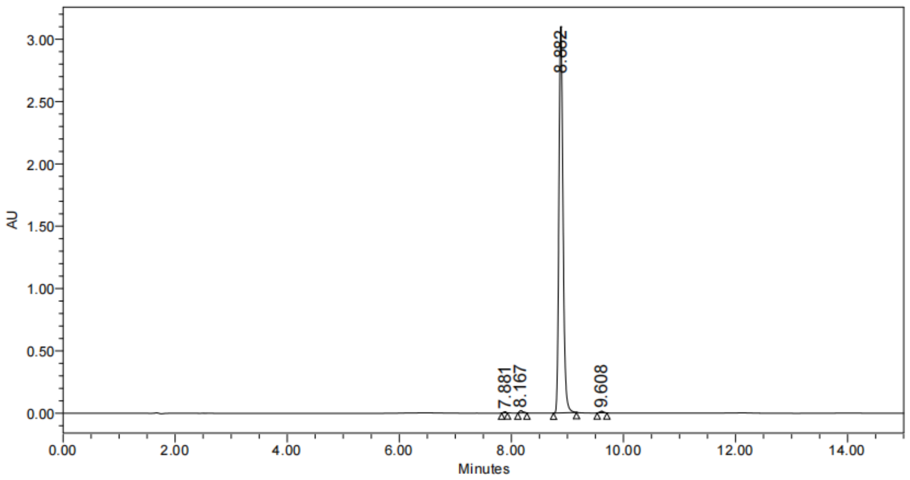

	RT	Area	% Area	Height
1	7.881	26104	0.16	8048
2	8.167	83436	0.52	18327
3	8.882	15836862	98.92	3097672
4	9.608	63103	0.39	12380

1-(4-fluoro-2-hydroxyphenyl)-2-(phenylthio)pent-4-en-1-one (**3e**)

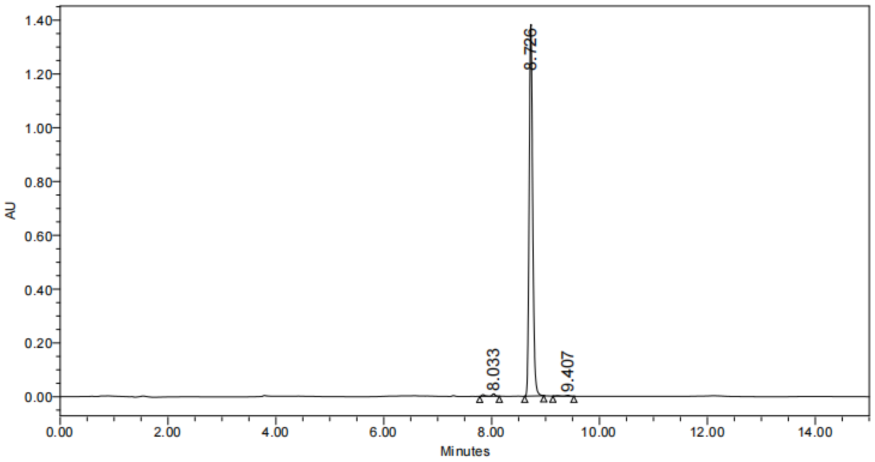

	RT	Area	% Area	Height
1	8.033	59361	0.93	8680
2	8.726	6275975	98.69	1381458
3	9.407	23683	0.37	2893

2-((2-chlorophenyl)thio)-1-(2-hydroxyphenyl)pent-4-en-1-one (**3m)**

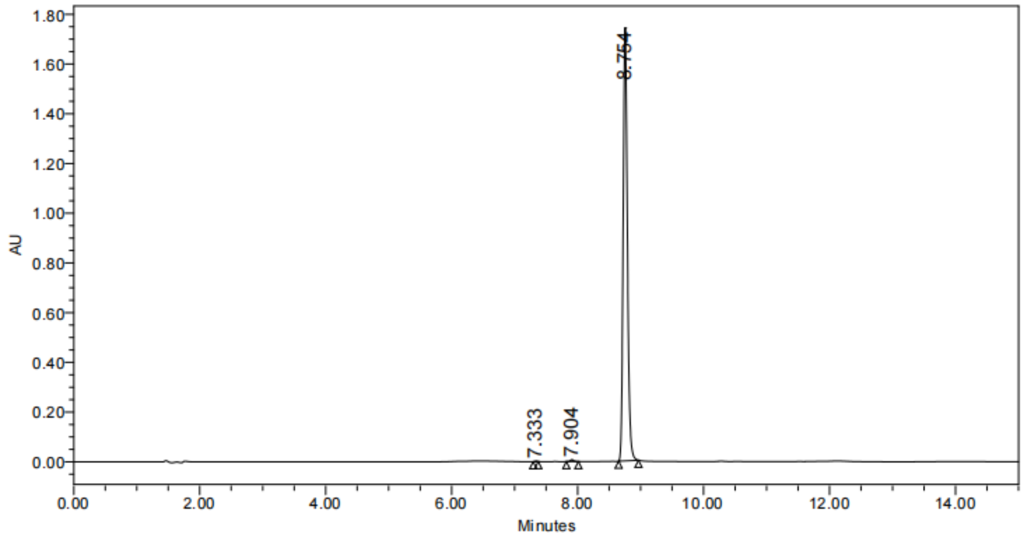

	RT	Area	% Area	Height
1	7.333	3793	0.05	1422
2	7.904	28087	0.34	6308
3	8.754	8128388	99.61	1742610

1-(2-hydroxyphenyl)-2-(m-tolylthio)pent-4-en-1-one (**3n)**

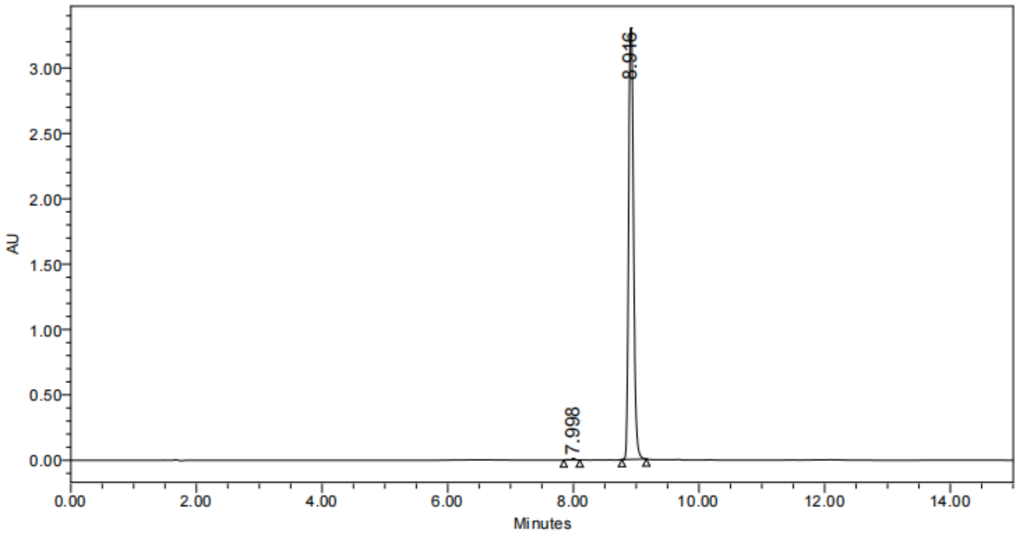

	RT	Area	% Area	Height
1	7.998	58782	0.33	11651
2	8.916	18009064	99.67	3300720

2-((4-bromophenyl)thio)-1-(2-hydroxyphenyl)pent-4-en-1-one (**3s)**

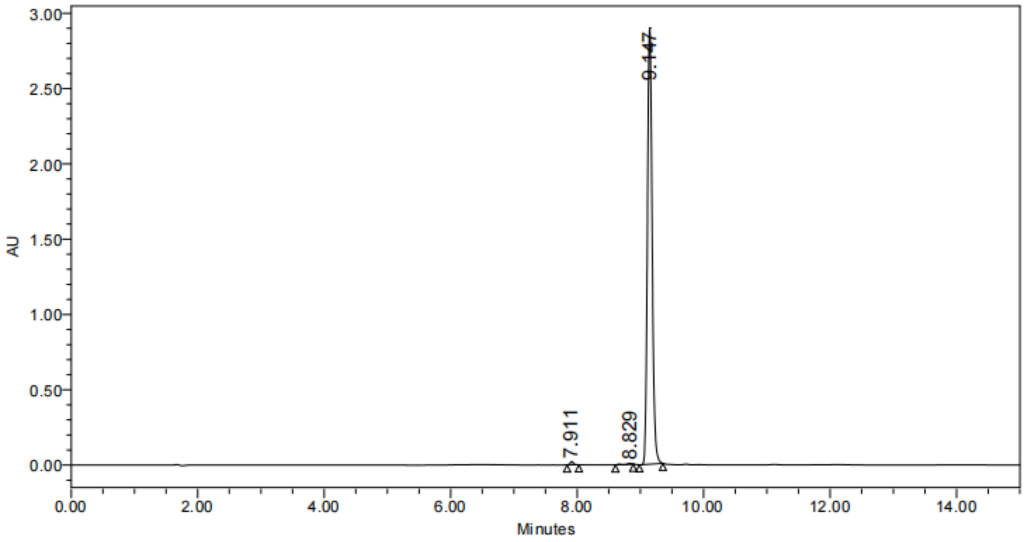

	RT	Area	% Area	Height
1	7.911	78436	0.52	20650
2	8.829	36681	0.24	5373
3	9.147	15030568	99.24	2896312

1-(2-hydroxyphenyl)-2-(naphthalen-2-ylthio)pent-4-en-1-one (**3u)**

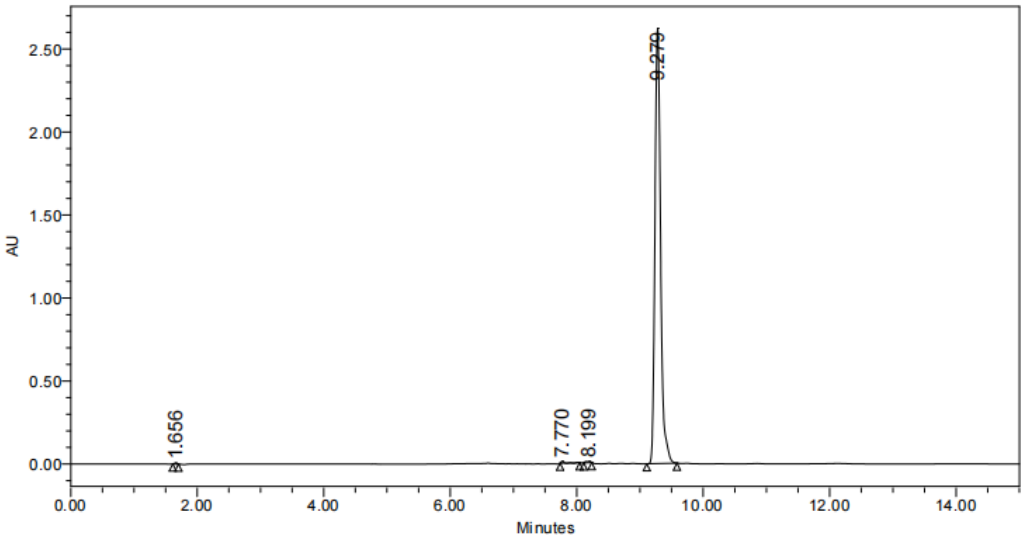

	RT	Area	% Area	Height
1	1.656	19940	0.12	6364
2	7.770	46144	0.28	10882
3	8.199	42153	0.26	7687
4	9.279	16311714	99.34	2621843

2-(benzylthio)-1-(2-hydroxyphenyl)pent-4-en-1-one (**3w**)

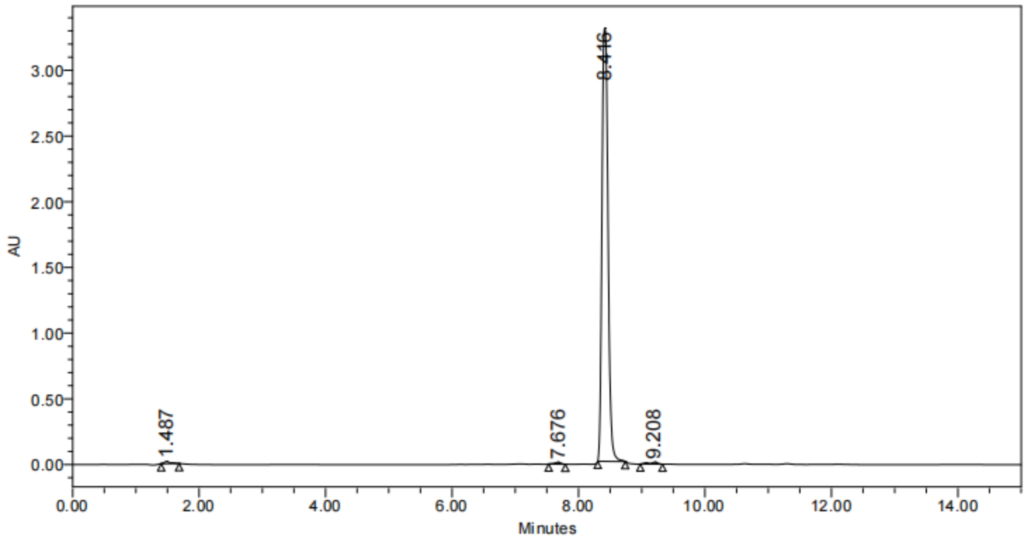

	RT	Area	% Area	Height
1	1.487	117709	0.53	16507
2	7.676	99011	0.44	13716
3	8.416	21952547	98.37	3296699
4	9.208	147131	0.66	14551

2-(allylthio)-1-(2-hydroxyphenyl)pent-4-en-1-one (**3y**)

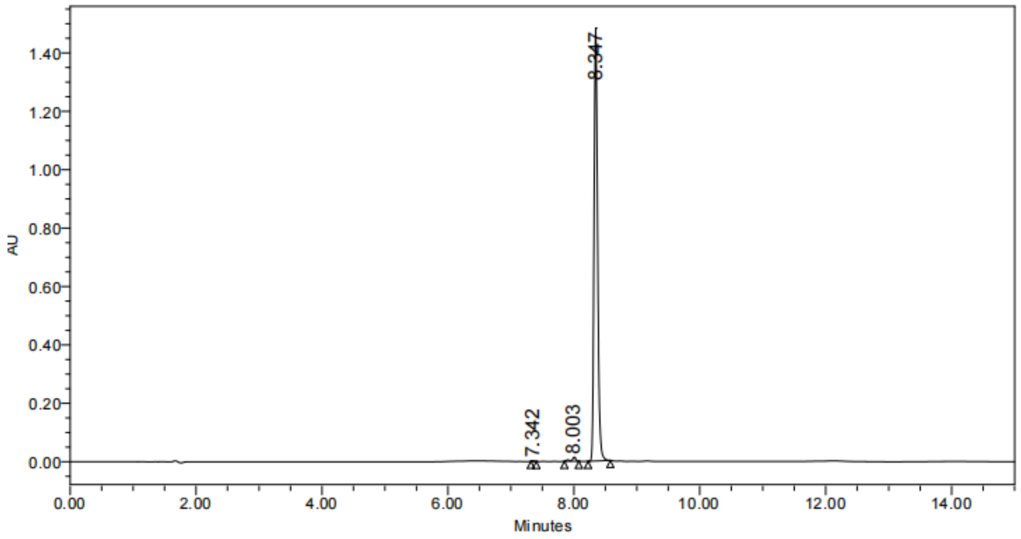

	RT	Area	% Area	Height
1	7.342	3849	0.07	1284
2	8.003	61816	1.05	13152
3	8.347	5824684	98.89	1482410

1-(2-hydroxyphenyl)-2-(methylthio)pent-4-en-1-one (**3za)**

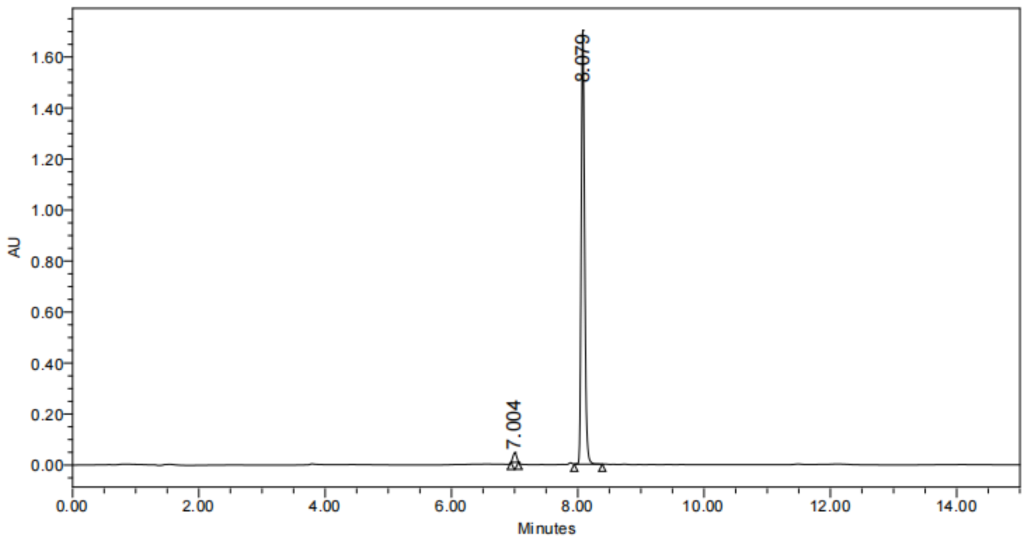

	RT	Area	% Area	Height
1	7.004	152633	2.37	37974
2	8.079	6289848	97.63	1702647

2-((3-chlorophenyl)thio)-1-(2-hydroxyphenyl)-3-methylpent-4-en-1-one (**3zb)**

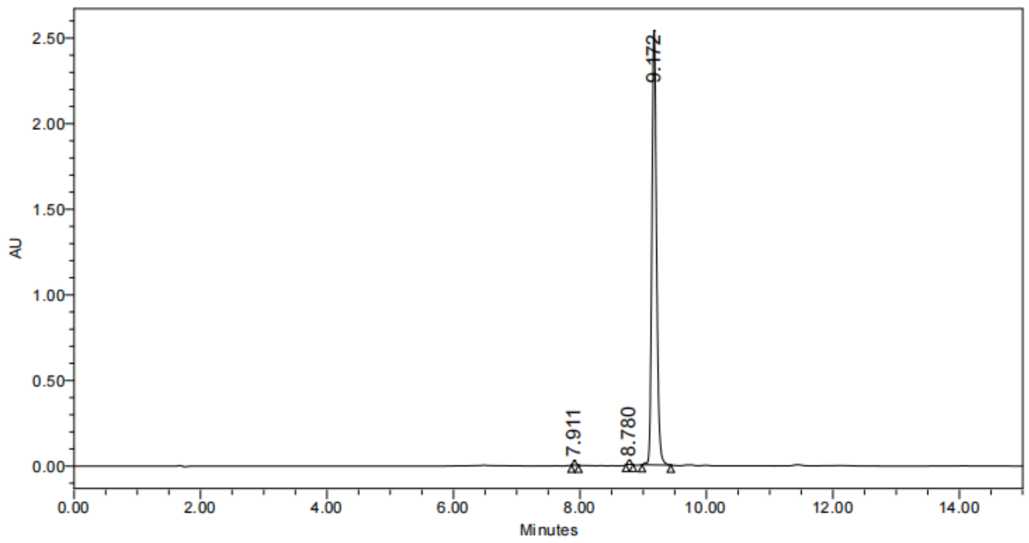

	RT	Area	% Area	Height
1	7.911	90791	0.69	28834
2	8.780	94265	0.72	25449
3	9.172	12948178	98.59	2537136

dimethyl 2-(phenyl(pivalamido)-λ[4]-sulfaneylidene)malonate: (**7l)**

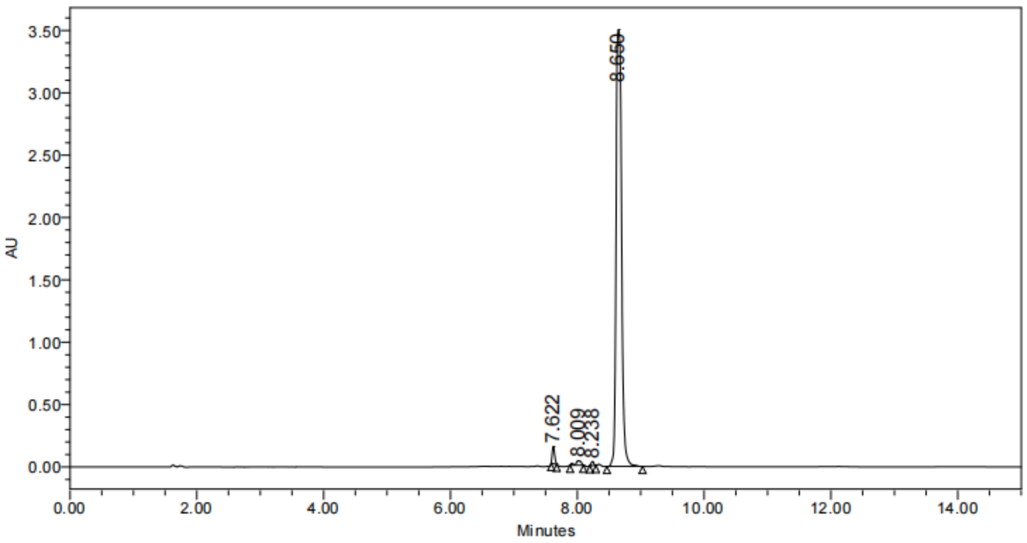

	RT	Area	% Area	Height
1	7.622	358895	1.65	138275
2	8.009	217433	1.00	33608
3	8.238	90378	0.42	30494
4	8.650	21070099	96.93	3502821

(*Z*)-*N*-((2,4-dioxochroman-3-ylidene)(phenyl)-λ[4]-sulfaneyl)benzamide (**7r)**

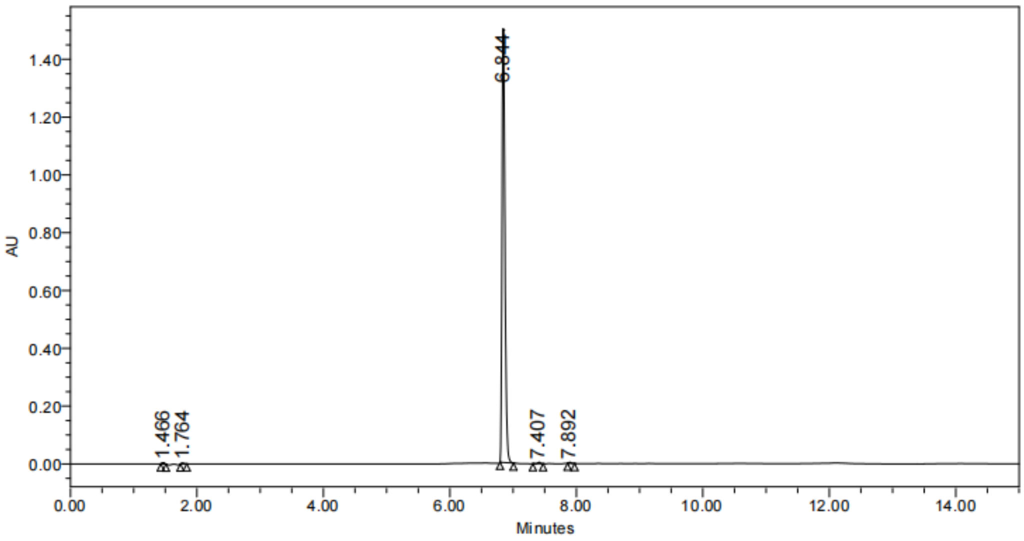

	RT	Area	% Area	Height
1	1.466	13094	0.27	4713
2	1.764	7633	0.16	2235
3	6.844	4808980	99.13	1502165
4	7.407	12979	0.27	4416
5	7.892	8726	0.18	2949
